# Systematic Hydrogen‐Bond Manipulations To Establish Polysaccharide Structure–Property Correlations

**DOI:** 10.1002/anie.201906577

**Published:** 2019-08-19

**Authors:** Yang Yu, Theodore Tyrikos‐Ergas, Yuntao Zhu, Giulio Fittolani, Vittorio Bordoni, Ankush Singhal, Richard J. Fair, Andrea Grafmüller, Peter H. Seeberger, Martina Delbianco

**Affiliations:** ^1^ Department of Biomolecular Systems Max-Planck-Institute of Colloids and Interfaces Am Mühlenberg 1 14476 Potsdam Germany; ^2^ Department of Chemistry and Biochemistry Freie Universität Berlin Arnimallee 22 14195 Berlin Germany; ^3^ Department of Theory Max-Planck-Institute of Colloids and Interfaces Am Mühlenberg 1 14476 Potsdam Germany; ^4^ Current affiliation: X-Chem Pharmaceutical 100 Beaver St. Waltham MA 02453 USA

**Keywords:** automated glycan assembly, carbohydrates, hydrogen bonds, molecular dynamics, structure–property correlations

## Abstract

A dense hydrogen‐bond network is responsible for the mechanical and structural properties of polysaccharides. Random derivatization alters the properties of the bulk material by disrupting the hydrogen bonds, but obstructs detailed structure–function correlations. We have prepared well‐defined unnatural oligosaccharides including methylated, deoxygenated, deoxyfluorinated, as well as carboxymethylated cellulose and chitin analogues with full control over the degree and pattern of substitution. Molecular dynamics simulations and crystallographic analysis show how distinct hydrogen‐bond modifications drastically affect the solubility, aggregation behavior, and crystallinity of carbohydrate materials. This systematic approach to establishing detailed structure–property correlations will guide the synthesis of novel, tailor‐made carbohydrate materials.

## Introduction

**Figure 1:**
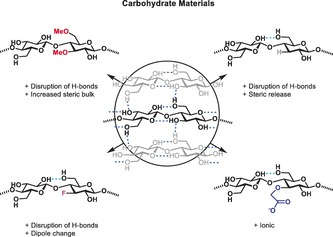
Systematic approach to study structure–property correlations in carbohydrate materials.

Cellulose, the most dominant polysaccharide on earth, with 700 billion tons produced annually, is an important material in the textile, food, paper, and pharmaceutical industries.^1^ The stability, crystallinity, and poor water solubility of cellulose are the result of a dense network of inter‐ and intramolecular hydrogen bonds that create allomorphs with different properties (Figure 1).^2^ The hydrogen bond between OH(3) and O(5) of the ring stabilizes the cellobiose repeating unit, with additional stabilization gained from intra‐ and intermolecular interactions (chain stacking) involving OH(6) and OH(2).^3^ Hydrophobic interactions between the CH‐rich, apolar faces of the glucose units as well as van der Waals forces also play an important role.^4^ Although different levels of cellulose organization have been studied in detail, not all the allomorphs have been described.^5^ Chemical modification alters the organization of cellulose and creates new materials with enhanced water solubility or ionic character.^6^ Non‐regioselective derivatization results in polydisperse materials with respect to the length and modification patterns, which do not allow for proper structure–function correlations.6d The lack of standards and experimental data has hampered in silico modeling studies. Molecular dynamics (MD) simulations capture some structural changes,^7^ but a detailed structural description is often lacking due to the flexibility of the carbohydrates.

The synthesis of carbohydrate materials by polymerization^8^ or enzymatic reactions^9^ provides an attractive alternative to the modification of natural polysaccharides, but has been limited by poor product solubility and narrow substrate scope. Laborious procedures for the synthesis of oligosaccharides have been overcome by automated glycan assembly (AGA), which enables rapid access to synthetic polysaccharides as long as 50‐mers.^10^ Well‐defined natural and unnatural glycans served as useful probes for systematic structural investigations, which revealed that even hexasaccharides adopt distinct secondary structures.^11^

Here, we use tailor‐made cellulose derivatives, designed to selectively disrupt hydrogen‐bond networks and/or alter the electronic properties, to establish a structure–property relationship (Figure 1).^12^ Methylated, deoxygenated, and deoxyfluorinated cellulose, in addition to well‐defined carboxymethyl cellulose (CMC) and chitin analogues, are prepared with full control over the length, pattern, and degree of substitution. MD simulations guided the synthesis, by correlating the disruption of the hydrogen‐bonding network with the increased flexibility of the modified oligosaccharides.

All the unnatural derivatives are highly water soluble as they aggregate less. Analogues with the same degree of substitution, but different substitution patterns, show dramatic differences in the conformation and aggregation. Crystallographic (XRD) and solubility analysis confirmed the in silico prediction, strongly supporting that fine‐tuning the natural polysaccharide backbone greatly influences the macroscopic material properties. These insights will guide the development of novel, high‐performance biomaterials.

## Results and Discussion

**Figure 2:**
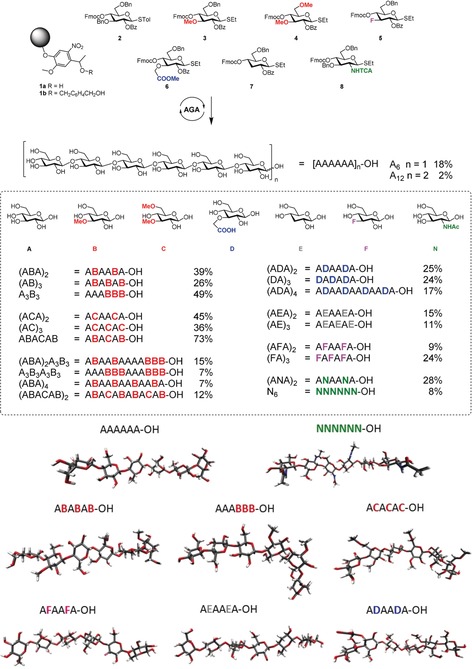
AGA of tailor‐made cellulose oligosaccharides and representative oligosaccharides conformations as obtained by MD simulations. Yields of isolated products after AGA, deprotection, and purification are reported.

Automated glycan assembly (AGA) increases the efficiency of oligosaccharide synthesis by iteratively combining monosaccharide building blocks (BBs) on a solid support, thus replacing laborious purification processes with simple washing steps.10a, 13 Each BB is equipped with a reactive thioglycoside leaving group and a temporary Fmoc protecting group that is easily removed after glycosylation to release a free hydroxy group that serves as the new glycosyl acceptor in the next coupling cycle. Iterative glycosylation and deprotection cycles allow for the step‐wise elongation of polysaccharides and the insertion of specific modifications in defined positions of the chain. The fully protected glycan target with a free reducing end is released from the solid support upon cleavage of the UV‐labile linker **1 a** (Figure 2).^14^ To overcome glycan decomposition during the basic methanolysis of the ester protecting groups in the presence of the free reducing end, a solid‐phase methanolysis was developed. Subsequent photocleavage and hydrogenolysis then afforded the desired oligosaccharides. Alternatively, cleavage of linker **1 b** liberates the desired glycan equipped with the 4‐hydroxymethylbenzyl group at the reducing end, thereby allowing for solution‐phase methanolysis and subsequent cleavage during hydrogenolysis. A collection of well‐defined cellulose derivatives was prepared by AGA (Figure 2). Two natural cellulose oligomers (hexamer A_6_ and dodecamer A_12_) and one chitin analogue (N_6_) served as standards for the structural analysis. Unnatural analogues with defined substitution patterns were prepared to tune the conformation and properties of the material. Regioselective functionalization was achieved with five “unnatural” monosaccharide BBs **3**–**7** (Figure 2). Global deprotection afforded oligosaccharide derivatives with complete control over the length, pattern, and degree of functionalization.

Methylation effectively alters the solubility and gelation properties of cellulose by influencing the intra‐ and intermolecular hydrogen bonding. Methylcellulose is widely used in the food and pharmaceutical industries.^15^ Six hexamers and four dodecamers with different methylation patterns were synthesized using BBs **3** and **4**, which contain 3‐methyl and 3,6‐dimethyl motifs, respectively (Figure 2). The position of the substituents was chosen to selectively disrupt hydrogen bonds that play a fundamental role in the rigidity of the cellulose. Methylation of OH(3) impedes the hydrogen bond between O(5) and OH(3), while 6‐methylation hinders the inter‐ and intrachain stabilization offered by OH(6). Structures with a regular methylation pattern (e.g. (AB)_3_), diblock analogues (e.g. A_3_B_3_), as well as irregularly functionalized structures (e.g. (ABA)_2_) were assembled to assess the effect of methylation patterns on the overall cellulose conformation. 3‐Deoxygenated BB **7** prevents the formation of hydrogen bonds between O(5) and OH(3), while 3‐deoxyfluorinated BB **5** not only prevents hydrogen‐bond formation, but is expected to affect the overall dipole of the oligomer as a result of the strong electron‐withdrawing nature of fluorine.12b, 16 Three CMC derivatives were prepared using BB **6** to evaluate the effect of negative charges on the resulting material. Lastly, one hybrid cellulose‐chitin derivative (ANA)_2_ was assembled.

The synthesis of A_6_ was low yielding (18 %) due to the low solubility of the oligosaccharide product. Methylation and carboxymethylation drastically improved the product solubility, which is reflected in higher yields for the unnatural hexasaccharide analogues (26–73 % overall yield). Similar results were observed for the 12‐mer syntheses (Figure 2) with the insertion of BBs **3**, **4**, and **6** with higher yields observed than for A_12_ (2 % overall yield). 3‐Deoxyfluorination and 3‐deoxygenation also improved the product solubility, but the reduced reactivity of BBs **5** and **7** as a glycosyl acceptor resulted in moderate yields (9–25 % overall).

**Figure 3:**
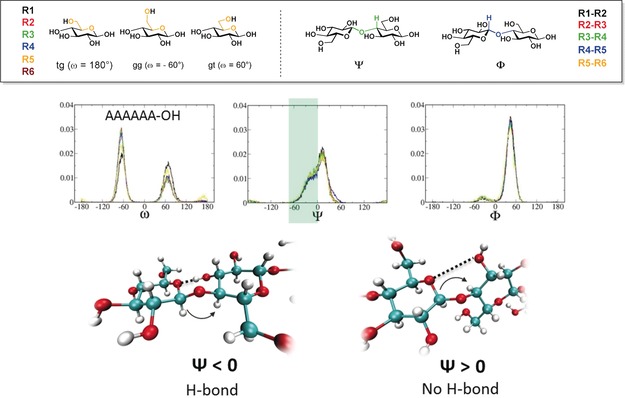
Analysis of the dihedral distributions obtained by MD simulations for A_6_. Negative degrees of **Ψ** (green box) are stabilized by the hydrogen bond between OH(3) and O(5), whereas the increased distance between these two residues is reflected by a positive **Ψ**. The residues are numbered from the nonreducing end (R1) to the reducing end (R6).

**Figure 4:**
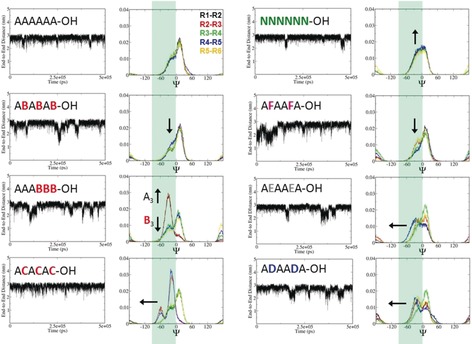
Analysis of end‐to‐end distances as a function of MD time and **Ψ** distribution obtained by MD simulations. The end‐to‐end distance was monitored over 500 ns. Large fluctuations are observed for all modified analogues, indicating that these molecules are more flexible. Changes in the population of **Ψ** at negative degrees (green box) are correlated to changes in hydrogen bonding between OH(3) and O(5). Changes in intensity are correlated with a decreased (↓) or increased (↑) rigidity, compared to A_6_; shifts (←) indicate that new geometries become accessible.

The perturbation of the 3D shape of the oligosaccharides as a result of single‐site substitutions was modeled using MD simulations, employing a modified version of the GLYCAM06 carbohydrate force field.^17^ The effect of substitution of the neighboring monomer on the torsion angles (**ω**, **Ψ**, **Φ**) was monitored and compared with the unsubstituted analogue A_6_ (Figure 3). Particular attention was paid to the changes in the population of **Ψ**, which is directly related to the presence of a hydrogen bond. To monitor the overall conformation of the hexamers, the end‐to‐end distance (Figure 4) and the radius of gyration (RoG; Figure S4) were calculated as a function of time. Cellulose A_6_ and chitin N_6_ oligomers showed a fairly rigid backbone core with low conformational variability (average end‐to‐end distance 2.76±0.17 nm for A_6_; Figure 4). Both structures tend to adopt an extended helical conformation (Figure 2). To examine how specific modifications affect such organized structures, the series of methylated analogues was studied (Figure 4).

A preliminary analysis of Cremer–Pople parameters^18^ showed surprisingly frequent ^4^C_1_‐to‐^1^C_4_ interconversions for all methylated analogues (**B** and **C**) during the simulation time (even at the monomer level). However, this tendency was disproved by NMR analysis of the ^1^ *J*_C1H1_ and ^3^ *J*_H1H2_ values (see the Supporting Information), and thus dihedral restraints were applied to these monomers (**B** and **C**) in the simulations to prevent the “flipping‐chair” artifact.

A regular alternating substitution pattern, as in the case of (AB)_3_, revealed a moderate, but important, decrease in the population of **Ψ** at negative degrees (−27°). This results from the increased distance between OMe(3) and O(5) because of the decreased tendency to form hydrogen bonds and the increased steric bulk (Figure 4). The same degree of methylation with a block distribution A_3_B_3_ resulted in dramatic changes. A significantly more flexible bent shape (Figure 2) with an end‐to‐end distance of 2.65±0.26 nm was observed for most of the simulation time. Surprisingly, the OH(3)⋅⋅⋅O(5) hydrogen bond between the first two glucose monomers was detected for most of the simulation time, thus suggesting the coexistence of a rigid rod block (A_3_) and a very flexible counterpart (B_3_; Figure 4).

Methylation at the 3‐ and 6‐positions (**C**), aimed to reduce inter‐ and intramolecular hydrogen bonds, disrupts the “standard” dihedral values, thereby resulting in a completely new geometry (Figure 4). Ramachandran plots of the dodecamers (Figure S16) confirmed that increased length enhances the resistance to deformation, since the cooperativity of intramolecular hydrogen‐bonding interactions stabilizes the overall structure. Nevertheless, a noticeable deviation from the main population of A_12_ was observed for all the substituted analogues. An irregular substitution pattern appears to be important to drastically change the cellulose conformation (e.g. (ABA)_2_A_3_B_3_). A regular substitution pattern such as (ABA)_4_ maintains more cellulose character while improving the water solubility.

Similar to methylation, deoxyfluorination and deoxygenation prevent the formation of hydrogen bonds between O(5) and OH(3). In addition, these substitutions influence the electron density along the chain (electronegative F) and the steric hindrance (deoxygenation). Since dipoles are key to the stability of cellulose, the replacement of OH(3) by the isosteric electron‐withdrawing F is expected to greatly influence the conformation of the resulting material. The calculated mean RoG for (AFA)_2_ shows a large dispersion and the average end‐to‐end distance is among the lowest (2.61±0.34 nm), indicative of a very flexible system (Figure 4) with a lower population at negative degrees of **Ψ_1_** and **Ψ_4_**. This effect extends beyond the single AF glycosidic bond, with significant variation of **Ψ_3_** (Figure 4). 3‐Deoxygenation had an even bigger effect on the **Ψ** distribution for (AEA)_2_, as reduced steric hindrance allows for more conformational freedom. The insertion of a carboxylic group (e.g. (ADA)_2_) resulted in a highly flexible, mostly linear conformation (Figure 2). Moreover, the carboxylate can engage in additional hydrogen bonds, as observed between COO^−^ and OH(2) of the same residue, as well as between COO^−^ and OH(6) of the adjacent previous sugar residue (OH(6)⋅⋅⋅COO^−^⋅⋅⋅OH(2)).

**Figure 5:**
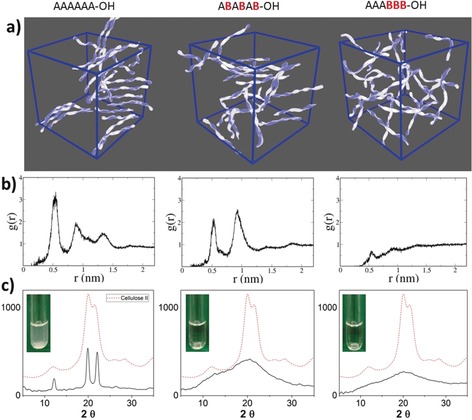
a) Representative snapshots of MD simulations of concentrated solutions, b) RDFs, and c) XRD patterns; inset: solubility test.

The behavior of the oligosaccharides in a crowded environment was studied and correlated to the crystallinity and solubility of the materials. Long MD simulations (1 μs production run) of concentrated experiments (see the Supporting Information) aimed to elucidate the molecular interactions. Radial distribution functions (RDFs) were used to characterize the spatial correlations in the systems (Figure 5). The RDF for A_6_ shows three sharp signals at small distances and remains large for distances up to 1.5 nm, which indicates high aggregation tendencies of such oligosaccharides. The more soluble methylated analogue (AB)_3_ shows some tendency to aggregate at high concentrations. However, a significantly decreased signal at 0.5 nm indicates the lower probability of finding two chains in proximity, compared to cellulose oligomers. RDF peaks are only found at shorter distances, thus revealing a lower tendency for cluster formation and a less organized structure, with a homogeneous distribution of molecules beyond the nearest neighbors. No aggregation was detected for A_3_B_3_, as expected from the high flexibility of such compounds, which should prevent chain stacking (Figure 5).

**Figure 6:**
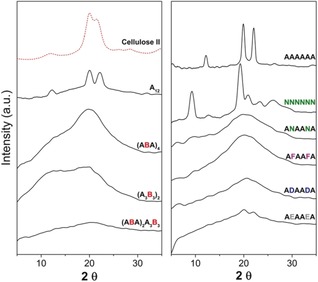
Powder XRD analysis of natural oligosaccharides (A_6_, A_12_, and N_6_) and all the modified analogues.

X‐ray diffraction and solubility data (Figure 5) support the calculations. As anticipated, A_6_ and A_12_ are very poorly soluble in water (less than 1 mg mL^−1^), due to the formation of cellulose‐like aggregates. Powder XRD measurements of both A_6_ and A_12_ gave sharp peaks (Figures 5 and 6) that are distinctive for cellulose II, thus indicating that short oligomers adopt the same aggregation pattern and the same hydrogen‐bonding arrangement as cellulose. The flat XRD profile of the diblock analogue A_3_B_3_ indicates the absence of any structural organization, as predicted by the theoretical model (Figure 5). The alternating methylation pattern of (AB)_3_ renders the material more sensitive to the X‐ray beam angle and, while the XRD peaks are still broad, they resemble the cellulose II structure, as predicted by MD simulations (Figure 5). This trend is confirmed by the longer oligomers, where more intense, yet broad, XRD profiles are observed for the regularly substituted analogues (Figure 6). No cellulose‐like character is detected for randomly functionalized structures. Similar to cellulose, the XRD profile of chitin analogue N_6_ is identical to that of natural chitin (Figures 5 and S2), as it is poorly soluble (13–17 mg mL^−1^) and tends to form gels at higher concentrations. Surprisingly, the hybrid cellulose‐chitin (ANA)_2_, is much more soluble (>50 mg mL^−1^) with no ordered supramolecular structures (Figure 6). All the functionalized cellulose analogues are, in contrast to the natural derivatives, highly water‐soluble (>50 mg mL^−1^) and form amorphous solids (Figure 6). Interestingly, although remaining highly water soluble, the deoxy series (**E**) adopts a cellulose‐like character in the solid state with two broad, but noticeable, peaks in the XRD profile (Figure 6) and a similar peak structure in the RDF (Supplementary Information).

## Conclusion

Tailor‐made cellulose oligosaccharide analogues, prepared by sequential addition of monomeric BBs using AGA, allow for control over the length and substitution patterns. Seven BBs were prepared bearing modifications to disrupt specific hydrogen bonds and tune the three‐dimensional shapes and properties of the materials. Methylation blocked the hydrogen bond between OH(3) and O(5), thereby resulting in an increased flexibility of the chain, as observed by MD simulations. A detailed dihedral analysis depicted how each glyosidic bond is affected by the modifications, and the consequences for the overall structure, such as fluctuation of the end‐to‐end distance during the simulation time. Compounds with the same degree of methylation, but different substitution patterns, behave drastically different. Regular substitution patterns result in quasilinear structures, whereas more bent geometries are observed with a block arrangement. These structural features control the aggregation process, which is expressed by high crystallinity for the natural compound and amorphous organization for irregular or block‐substituted analogues. A more significant disruption of the “standard” dihedral values was observed with methylation at the OH(3) and OH(6) positions, as well as for the deoxy derivatives (**E**). Interestingly, upon drying, the highly water‐soluble deoxy derivatives show a tendency for cellulose‐like packing. Replacement of the OH(3) group by the isosteric electron‐withdrawing F atom resulted in compact analogues (shortest end‐to‐end distance) and amorphous organization. Carboxylates (**D**) or amides (**N**) derivatives made new conformations accessible thanks to the formation of additional hydrogen bonds. All the unnatural analogues are drastically more soluble, due to the more flexible backbone. Novel biomaterials with tuned properties that could be engineered depending on the nature and pattern of the substituents can be envisioned. The collection of unnatural compounds will be available to evaluate enzymatic degradation and substrate specificity.

## Experimental Section

AGA was performed on a home‐built synthesizer developed at the Max‐Planck‐Institute of Colloids and Interfaces. MD simulations were performed on a modified version of the GLYCAM06 carbohydrate force field.^17^ After equilibration (NVT and NPT ensemble), single molecules were simulated for 500 ns using Gromacs 5.1.2.^19^

## Conflict of interest

The authors declare no conflict of interest.

## Supporting information

As a service to our authors and readers, this journal provides supporting information supplied by the authors. Such materials are peer reviewed and may be re‐organized for online delivery, but are not copy‐edited or typeset. Technical support issues arising from supporting information (other than missing files) should be addressed to the authors.

Supplementary
