# Supplementary material for: Systematic Hydrogen‐Bond Manipulations To Establish Polysaccharide Structure–Property Correlations
Source: Angew Chem Int Ed Engl. 2019 Aug 19;58(37):13127–32. doi: 10.1002/anie.201906577 (PMC6772130; doi:10.1002/anie.201906577)
Supplement: Supplementary file 1 — Supplementary [file ANIE-58-13127-s001.pdf]

Angewandte Chemie
Eine Zeitschrift der Gesellschaft Deutscher Chemiker

Supporting Information

Systematic Hydrogen-Bond Manipulations To Establish Polysaccharide Structure–Property Correlations

Yang Yu[+], Theodore Tyrikos-Ergas[+], Yuntao Zhu, Giulio Fittolani, Vittorio Bordoni, Ankush Singhal, Richard J. Fair, Andrea Grafmüller, Peter H. Seeberger, and Martina Delbianco**

anie_201906577_sm_miscellaneous_information.pdf

Table of Contents

Table of Contents 2

1. General Materials and Methods 3

2. Synthesis of Building Blocks 3

   2.1. Synthesis of 3 3

   2.2. Synthesis of 5 6

   2.3. Synthesis of 6 21

   2.4. Synthesis of 7 28

3. Automated Glycan Assembly 37

   3.1. General materials and methods 37

   3.2. Preparation of stock solutions 38

   3.3. Modules for automated synthesis 38

   3.4. Post-synthesizer manipulations 40

   3.5. Oligosaccharides synthesis 43

      3.5.1. Synthesis of hexamers 43

      3.5.2. Synthesis of 12-mers 92

4. Synthesis and NMR analysis of 3-*O*-methyl-D-glucopyranoside S1 112

5. Solubility Measurement 116

6. XRD Analysis 117

7. Molecular Dynamics Simulations 119

8. References 162

1. General Materials and Methods

All chemicals used were reagent grade and used as supplied unless otherwise noted. The automated syntheses were performed on a home-built synthesizer developed at the Max Planck Institute of Colloids and Interfaces. Analytical thin-layer chromatography (TLC) was performed on Merck silica gel 60 F254 plates (0.25 mm). Compounds were visualized by UV irradiation or dipping the plate in a p-anisaldehyde (PAA) solution. Flash column chromatography was carried out by using forced flow of the indicated solvent on Fluka Kieselgel 60 M (0.04 – 0.063 mm). Analysis and purification by normal and reverse phase HPLC was performed by using an Agilent 1200 series. Products were lyophilized using a Christ Alpha 2-4 LD plus freeze dryer. ^{1}H, ^{13}C and HSQC NMR spectra were recorded on a Varian 400-MR (400 MHz), Varian 600-MR (600 MHz), or Bruker Biospin AVANCE700 (700 MHz) spectrometer. Spectra were recorded in $CDCl_3$ by using the solvent residual peak chemical shift as the internal standard ($CDCl_3$: 7.26 ppm ^{1}H, 77.0 ppm ^{13}C) or in D_2O using the solvent as the internal standard in ^{1}H NMR (D_2O: 4.79 ppm ^{1}H). High resolution mass spectra were obtained using a 6210 ESI-TOF mass spectrometer (Agilent) and a MALDI-TOF autoflex™ (Bruker). MALDI and ESI mass spectra were run on IonSpec Ultima instruments. IR spectra were recorded on a Perkin-Elmer 1600 FTIR spectrometer. Optical rotations were measured by using a Perkin-Elmer 241 and Unipol L1000 polarimeter. For XRD measurements, a Bruker D8 Advanced X-ray diffractometer with Cu Kα radiation was used. Cellulose II was prepared following to previous literature.[1]

2. Synthesis of Building Blocks

2.1. Synthesis of 3

Ethyl 2-*O*-benzoyl-6-*O*-benzyl-4-*O*-fluorenylcarboxymethyl-3-*O*-methyl-thio-β-D-glucopyranoside, 3

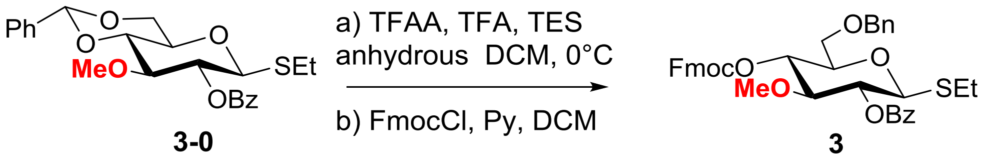

3-0 **3**

Compound **3-0** was prepared according to previous literature.[2]

Ethyl 2-*O*-benzoyl-4,6-*O*-benzylidene-3-*O*-methyl-thio-β-D-glucopyranoside **3-0** (9.7 g, 22.5 mmol) was dissolved in anhydrous dichloromethane (DCM) (125 mL). Triethylsilane (TES) (21.6 mL, 135 mmol) was added and the solution was cooled to 0°C. Trifluoroacetic acid (TFA) (8.7 mL, 113 mmol) and trifluoroacetic anhydride (TFAA) (1.6 mL, 11.3 mmol) were added sequentially. The solution was stirred at 0°C for 3 hours. The reaction was diluted with DCM (150 mL) and washed with saturated aq. $NaHCO_3$ (2 x 50 mL) and H_2O (1 x 50 mL). The organics were dried over $MgSO_4$, filtered, and evaporated. The remaining pale yellow oil was dissolved in pyridine and the solvent was removed under reduced pressure. The crude compound was dissolved in DCM (75 mL). Pyridine (Py) (6.8 mL, 67.6 mmol) was added followed by Fmoc-Cl (11.7 g, 45.1 mmol). The yellow solution was stirred at room temperature under inert Ar atmosphere until completion (3 h). The reaction was diluted with DCM (100 mL) and washed with 1 M HCl (1 x 50 mL), saturated aq. $NaHCO_3$ (1 x 50 mL), and H_2O (1 x 50 mL). The organics were dried over $MgSO_4$, filtered, and evaporated. The crude product was purified by column chromatography (Hexane : EtOAc = 4:1) to give **3** as a pale yellow solid (9.7 g,

66%). ^{1}H NMR (400 MHz, $CDCl_3$) δ 8.09 (d, *J* = 8.0 Hz, 2H), 7.77 (d, *J* = 7.5 Hz, 2H), 7.63 – 7.55 (m, 3H), 7.47 (t, *J* = 7.7 Hz, 2H), 7.41 (t, *J* = 7.5 Hz, 2H), 7.36 – 7.27 (m, 7H), 5.27 (t, *J* = 9.6 Hz, 1H), 4.92 (t, *J* = 9.5 Hz, 1H), 4.58 (d, *J* = 10.0 Hz, 1H), 4.55 (s, 2H), 4.47 (dd, *J* = 10.4, 7.4 Hz, 1H), 4.36 (dd, *J* = 10.4, 6.9 Hz, 1H), 4.21 (t, *J* = 7.1 Hz, 1H), 3.75 (dt, *J* = 11.9, 3.3 Hz, 1H), 3.73 – 3.64 (m, 3H), 3.41 (s, 3H), 2.80 – 2.67 (m, 2H), 1.25 (t, *J* = 7.4 Hz, 3H); ^{13}C NMR (101 MHz, $CDCl_3$) δ 165.14, 154.44, 143.40, 143.26, 141.46, 141.42, 138.01, 133.40, 129.98, 129.84, 128.60, 128.45, 128.03, 127.74, 127.29, 127.27, 125.17, 125.13, 120.20, 83.82, 83.28, 77.36, 75.00, 73.73, 71.89, 70.10, 69.77, 60.17, 46.88, 24.25, 15.02; R_f = 0.7 (Silica, Hexane : EtOAc = 3:1); $[\alpha]_D^{20}$ +4.60 (c 1, $CHCl_3$); IR (neat) ν_{max} = 1754, 1729 cm^{-1}; m/z (HRMS+) 677.2191 $[M + Na]^+$ ($C_{38}H_{38}O_8NaS$ requires 677.2180).

^{1}H NMR of 3 (400 MHz, $CDCl_3$)

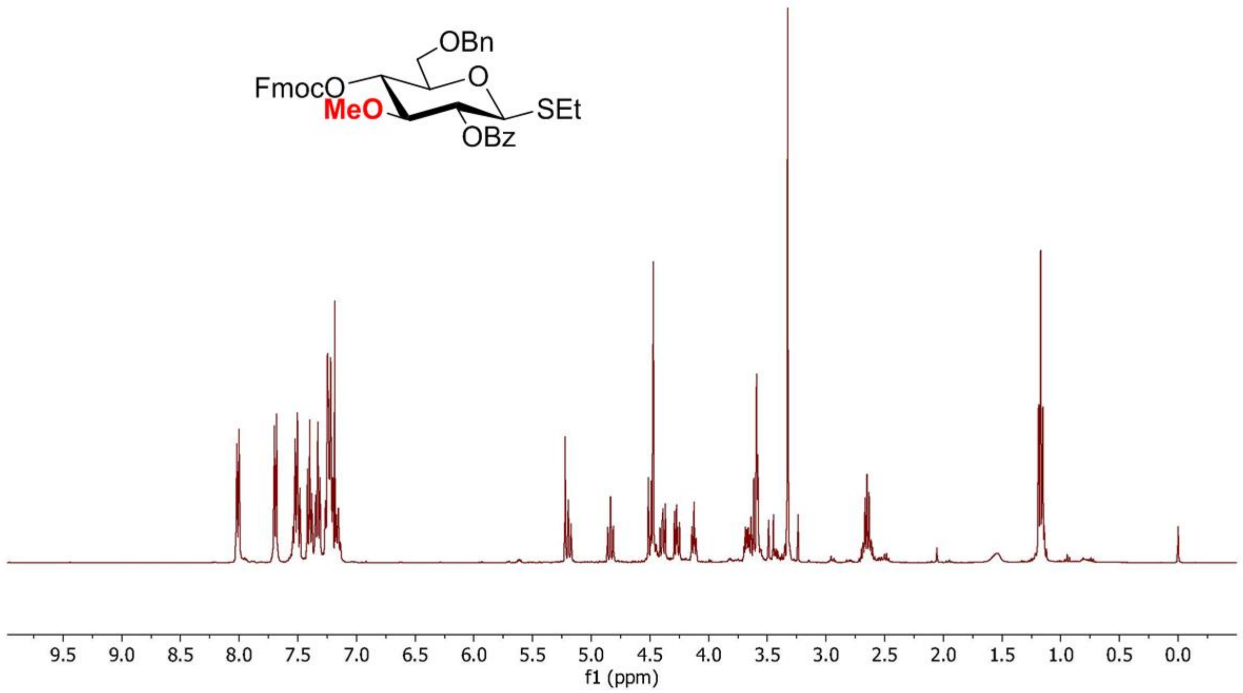

^{13}C NMR of 3 (101 MHz, $CDCl_3$)

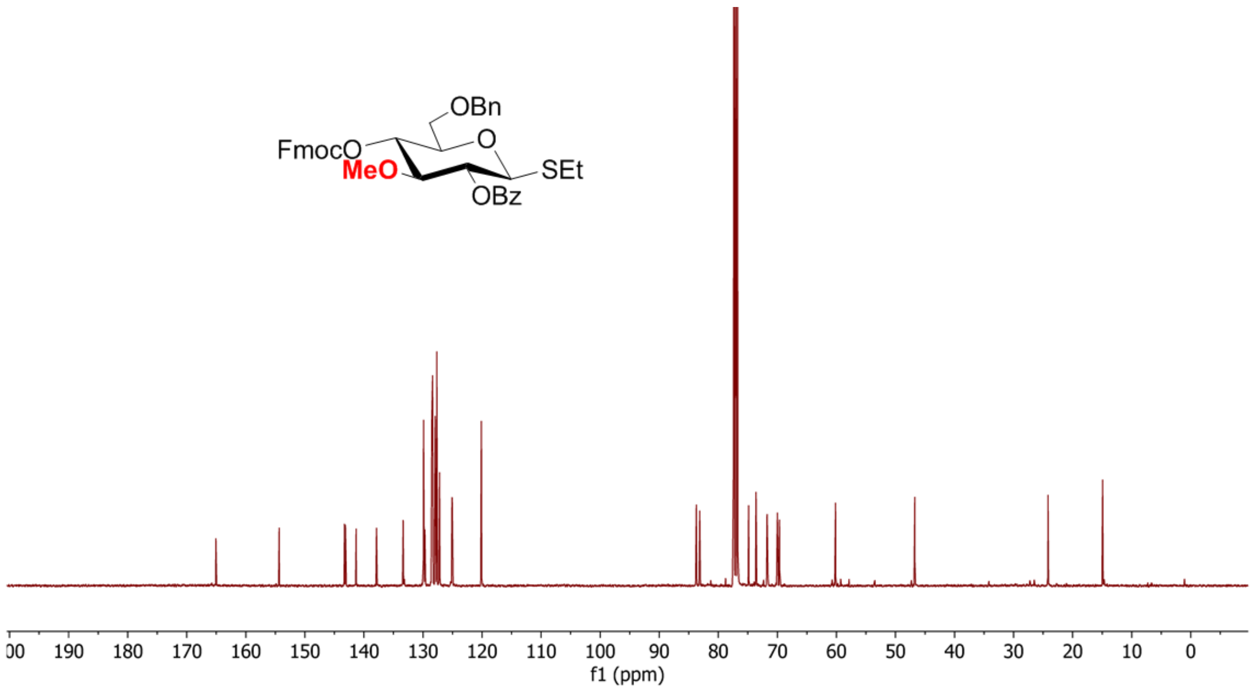

HSQC NMR of 3 ($CDCl_3$)

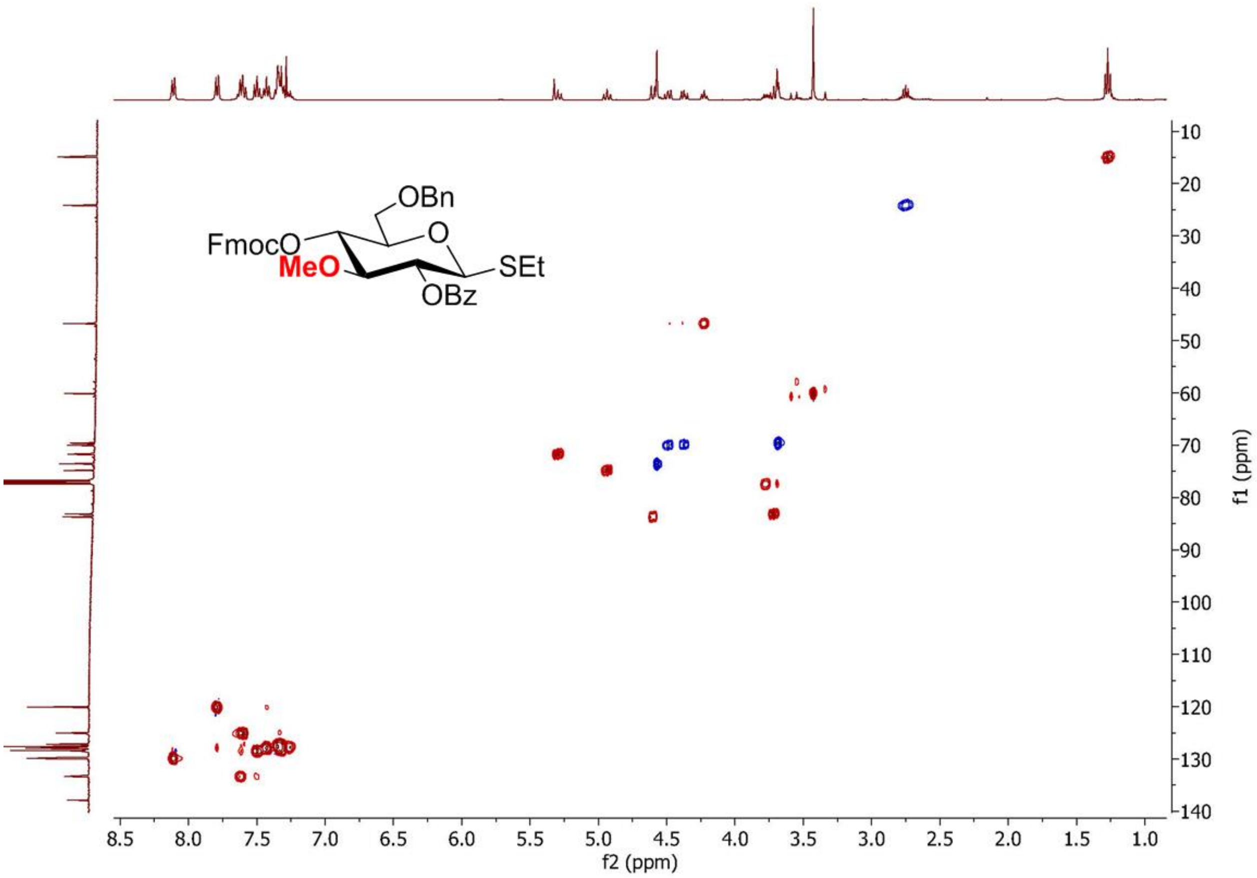

2.2. Synthesis of 5

Ethyl 2,4,6-tri-*O*-acetyl-3-deoxy-3-fluoro-1-thio-β-D-glucopyranoside, 5-1

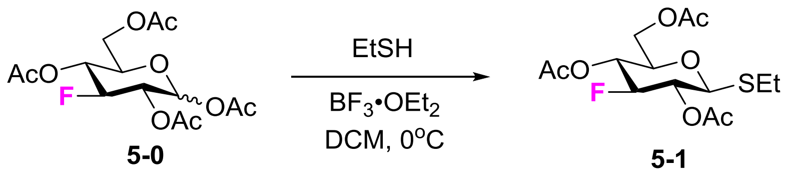

Compound **5-0** was prepared according to previous literature.[3]

BF_3·EtO_2 complex (3.5 mL, 27.6 mmol) was slowly added to a stirred solution of 3-deoxy-3-fluoro-1,2,4,6-tetra-*O*-acetyl-α/β-D-glucopyranose **5-0** (6.46 g, 18.4 mmol) with EtSH (3.4 mL, 46.0 mmol) in DCM (40 mL) at 0°C under nitrogen atmosphere. After 3h at 0°C the reaction was quenched with sat. aq. solution of $NaHCO_3$. The mixture was diluted with DCM and washed three times with saturated aqueous solution of $NaHCO_3$ and one time with brine. The organic phase was dried over Na_2SO_4 and concentrated under reduced pressure. The crude product was purified by flash column chromatography (Hexane : EtOAc = 4:1→2:1) to yield **5-1** as colorless thick oil (2.36 g, 36%). ^{1}H NMR (400 MHz, $CDCl_3$) δ 5.25 – 5.07 (m, 2H), 4.55 (dt, *J* = 52.3, 9.0 Hz, 1H), 4.40 (d, *J* = 10.1 Hz, 1H), 4.22 (dd, *J* = 12.4, 5.0 Hz, 1H), 4.13 (ddd, *J* = 12.7, 2.6, 1.4 Hz, 1H), 3.61 (dddd, *J* = 9.8, 4.6, 2.2, 1.0 Hz, 1H), 2.78 – 2.60 (m, 2H), 2.12 (s, 3H), 2.09 (s, 3H), 2.07 (s, 3H), 1.25 (t, *J* = 7.4 Hz, 3H); ^{13}C NMR (101 MHz, $CDCl_3$) δ 170.80 (s), 169.34 (s), 169.33 (s), 92.67 (d, *J* = 192.6 Hz), 83.01 (d, *J* = 7.9 Hz), 75.33 (d, *J* = 7.3 Hz), 69.75 (d, *J* = 18.1 Hz), 68.29 (d, *J* = 18.4 Hz), 62.09 (d, *J* = 1.8 Hz), 24.18, 20.94, 20.87, 20.80, 14.84;^{19}F NMR (376 MHz, $CDCl_3$) δ -192.44 (dt, *J* = 52.2, 12.3 Hz); $[\alpha]_D^{20}$ -13.71 (*c* 0.56 g/100 mL, $CHCl_3$); IR (neat) ν_{max} = 2926, 1744, 1373, 1212, 1033 cm^{-1}; (ESI-HRMS) m/z 375.0890 $[M+Na]^+$ ($C_{14}H_{21}FO_7SNa$ requires 375.0890).

^{1}H NMR of 5-1 (400 MHz, $CDCl_3$)

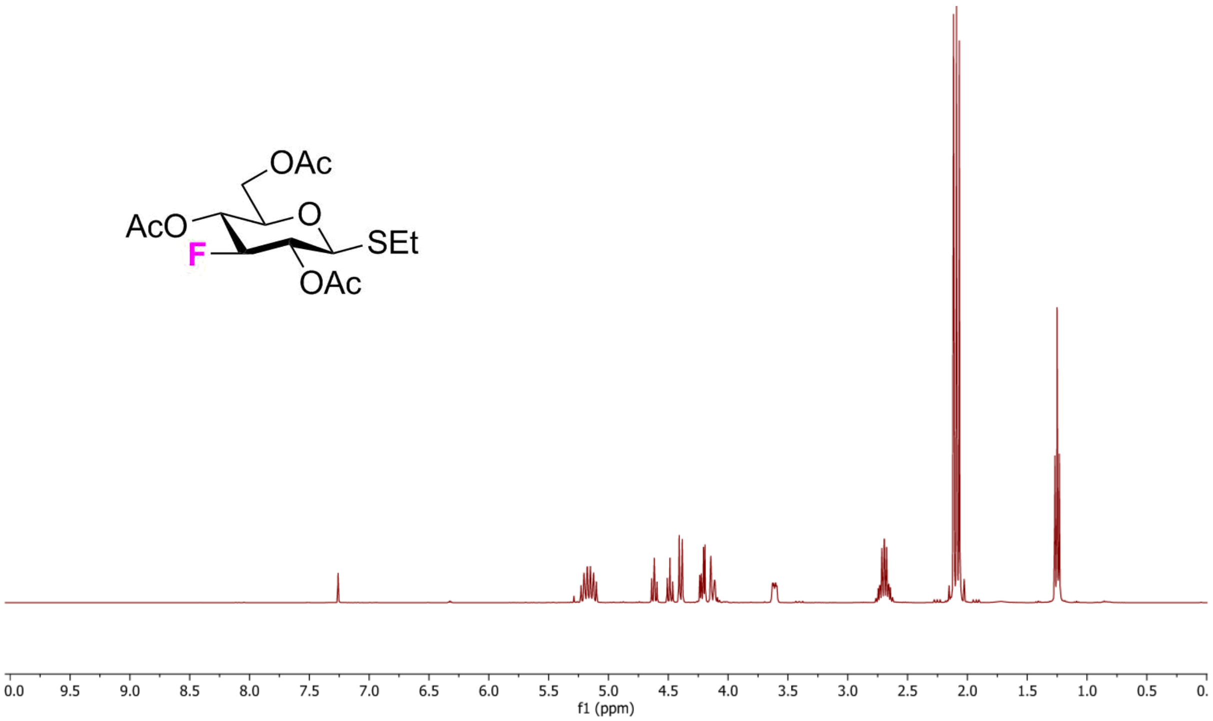

^{13}C NMR of 5-1 (101 MHz, $CDCl_3$)

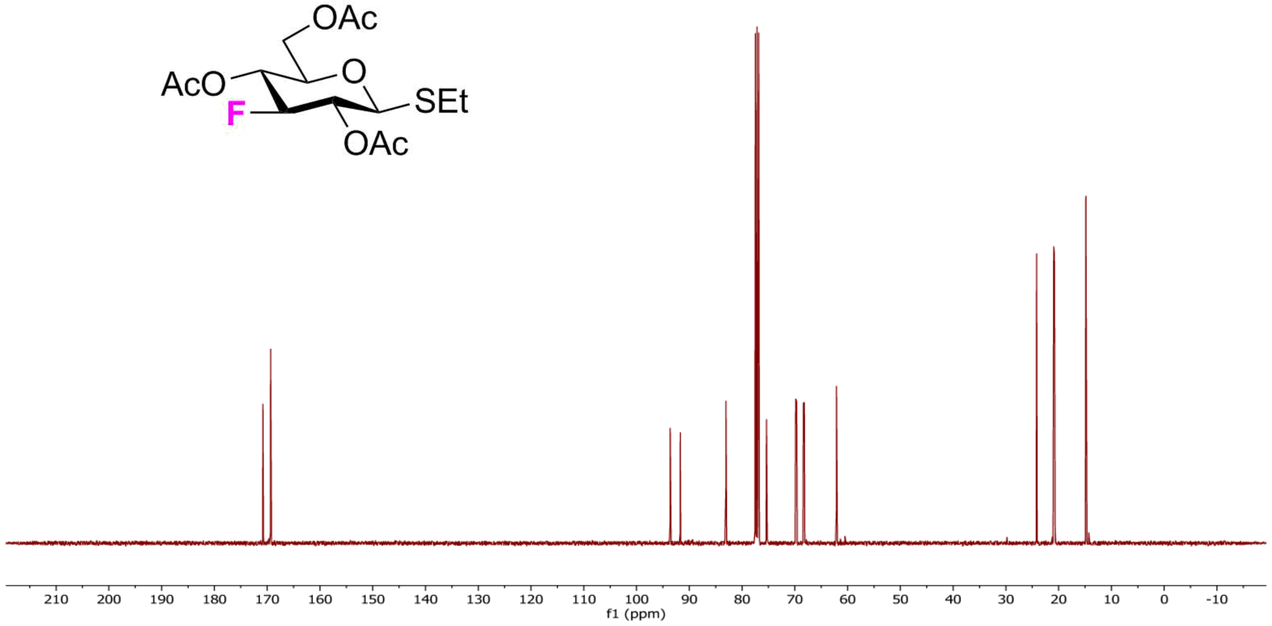

^{19}F NMR of 5-1 (376 MHz, $CDCl_3$)

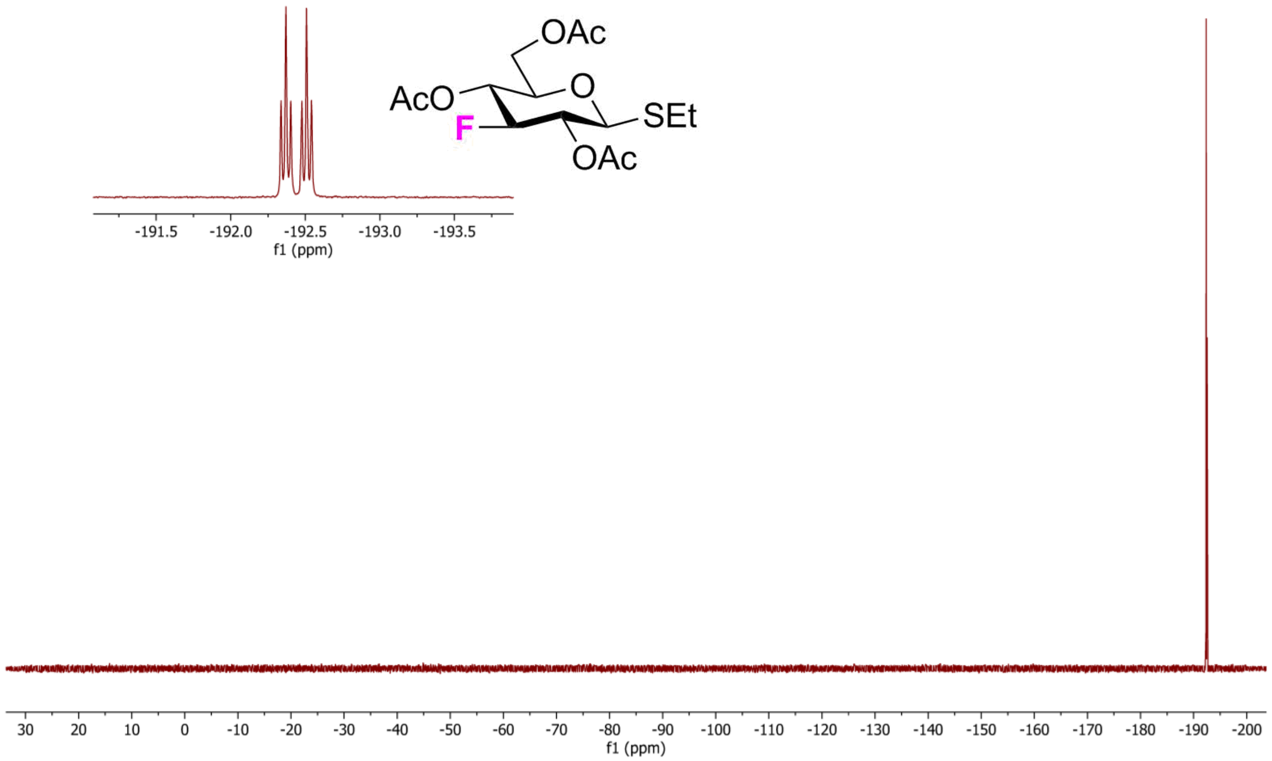

HSQC NMR of 5-1 ($CDCl_3$)

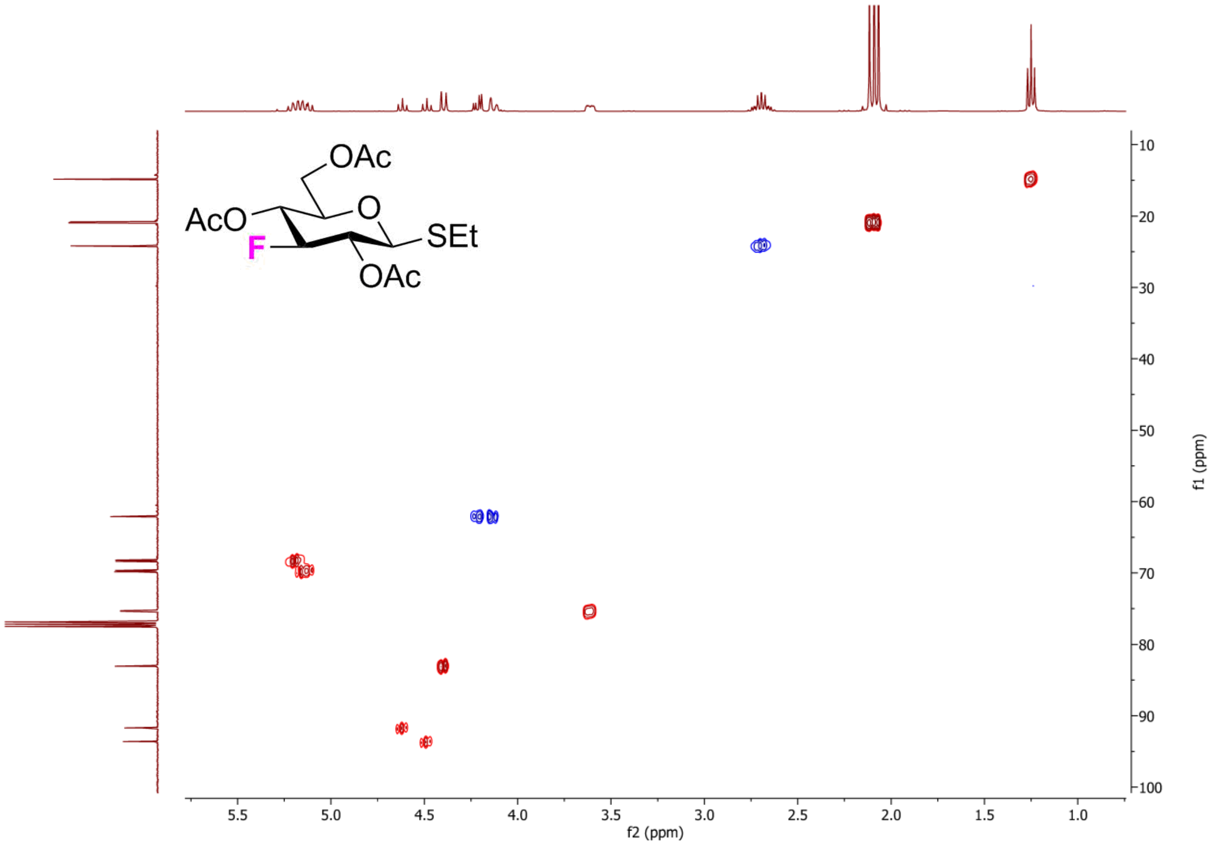

Ethyl 3-deoxy-3-fluoro-1-thio-β-D-glucopyranoside, 5-2

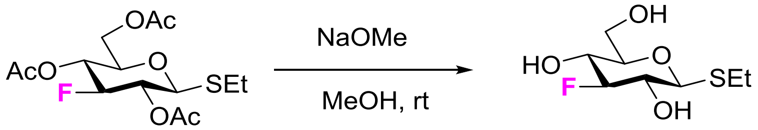

Ethyl 3-deoxy-3-fluoro-2,4,6-*O*-tri-acetyl-1-thio-β-D-glucopyranoside **5-1** (1.23 g, 3.5 mmol) was dissolved in anhydrous MeOH (30 mL) and a 0.5 M solution of MeONa in MeOH (7.0 mL, 3.5 mmol) was slowly added at room temperature. The mixture was stirred at room temperature for 1h. The reaction was neutralized with Amberlite IR-120 (H^+ form), filtered, and concentrated under reduced pressure to yield **5-2** as a sticky oil. The product was used in the next step without any further purification assuming quantitative conversion (0.79 g, quantitative). ^{1}H NMR (400 MHz, $CDCl_3$) δ 4.55 – 4.32 (m, 2H), 3.96 – 3.81 (m, 3H), 3.60 (ddd, *J* = 12.7, 9.8, 8.6 Hz, 1H), 3.46 – 3.37 (m, 1H), 2.76 (q, *J* = 7.4 Hz, 1H), 1.33 (t, *J* = 7.4 Hz, 1H); ^{13}C NMR (101 MHz, $CDCl_3$) δ 97.59 (d, *J* = 184.5 Hz), 85.73 (d, *J* = 8.9 Hz), 78.50 (d, *J* = 7.1 Hz), 70.91 (d, *J* = 17.5 Hz), 68.97 (d, *J* = 18.4 Hz), 62.08, 24.97, 15.35; ^{19}F NMR (376 MHz, $CDCl_3$) δ -192.59 (dt, *J* = 53.1, 13.1 Hz); $[\alpha]_D^{20}$ -36.47 (*c* 0.43 g/100 mL, MeOH); IR (neat) ν_{max} = 3375, 1073, 1034 cm^{-1}; (ESI-HRMS+) m/z 249.0584 $[M+Na]^+$ ($C_8H_{15}FO_4SNa$ requires 249.0573).

^{1}H NMR of 5-2 (400 MHz, $CDCl_3$)

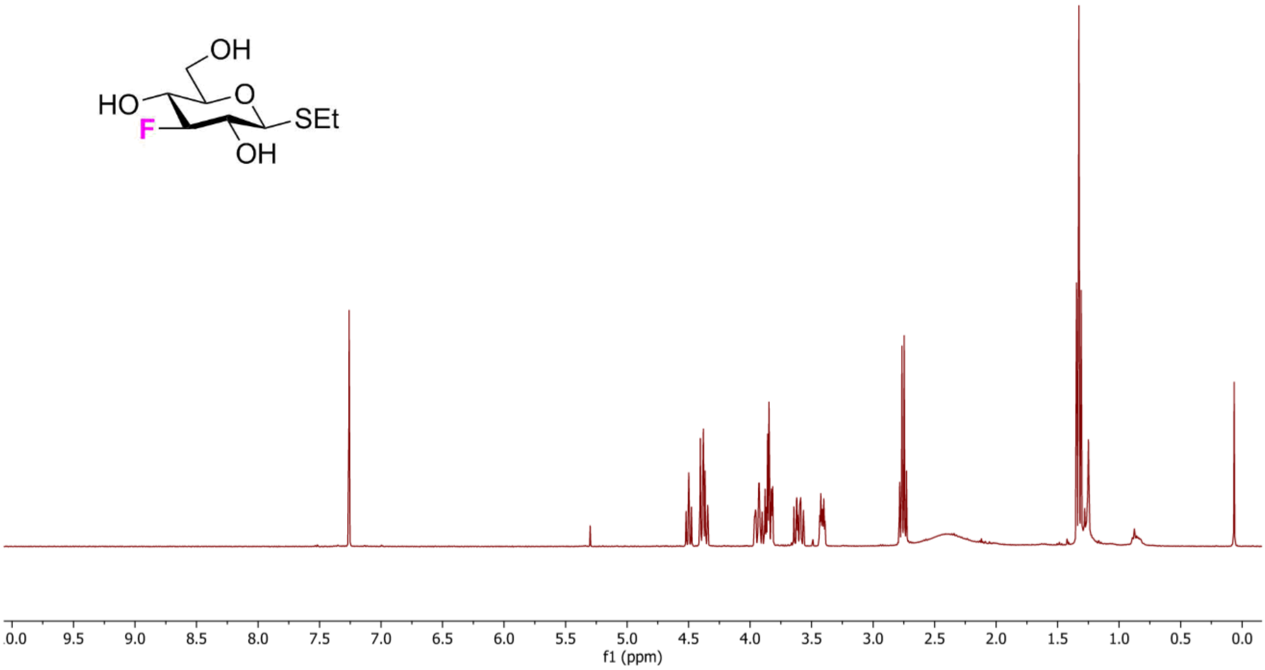

^{13}C NMR of 5-2 (101 MHz, $CDCl_3$)

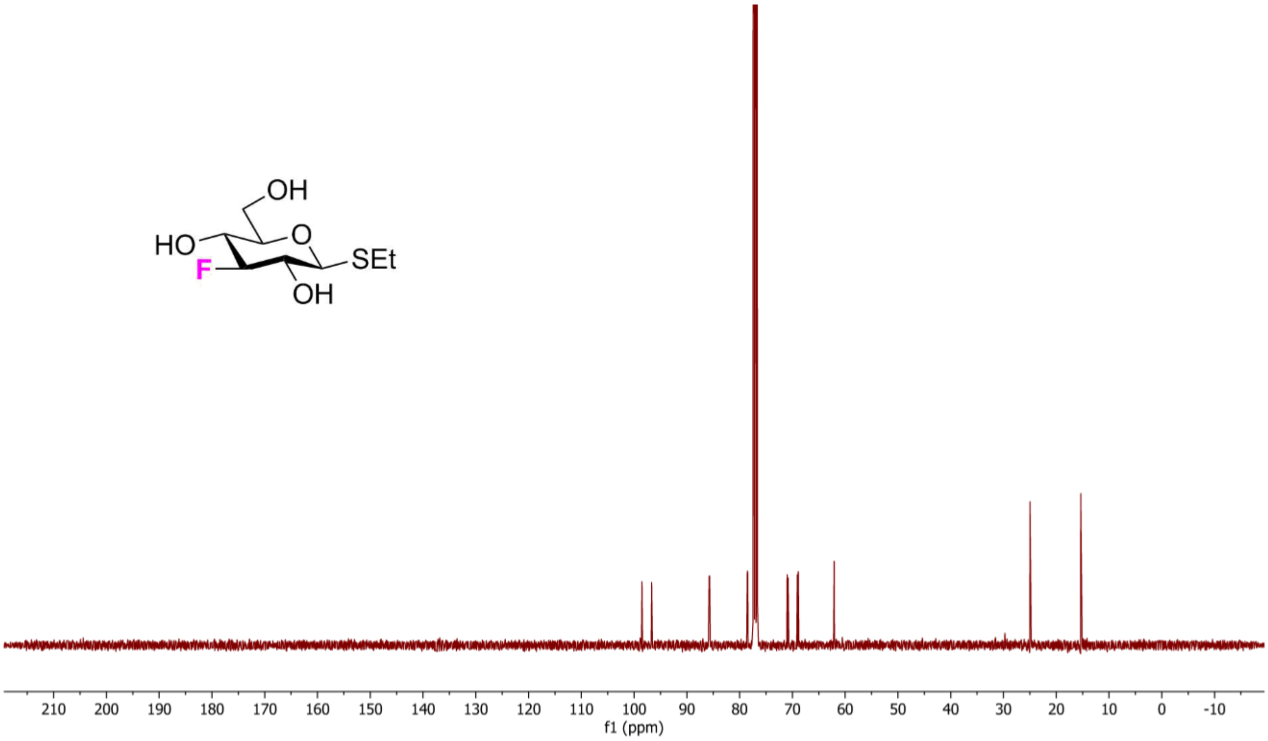

^{19}F NMR of 5-2 (376 MHz, $CDCl_3$)

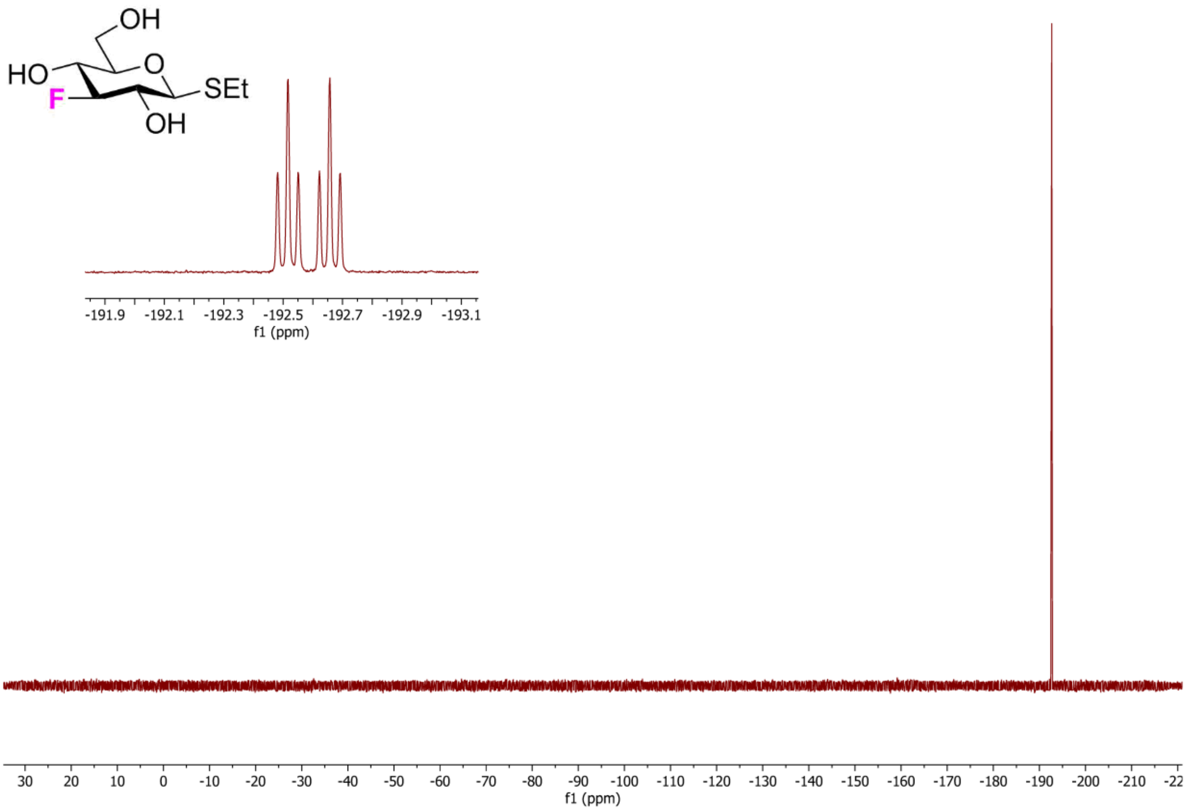

HSQC NMR of 5-2 ($CDCl_3$)

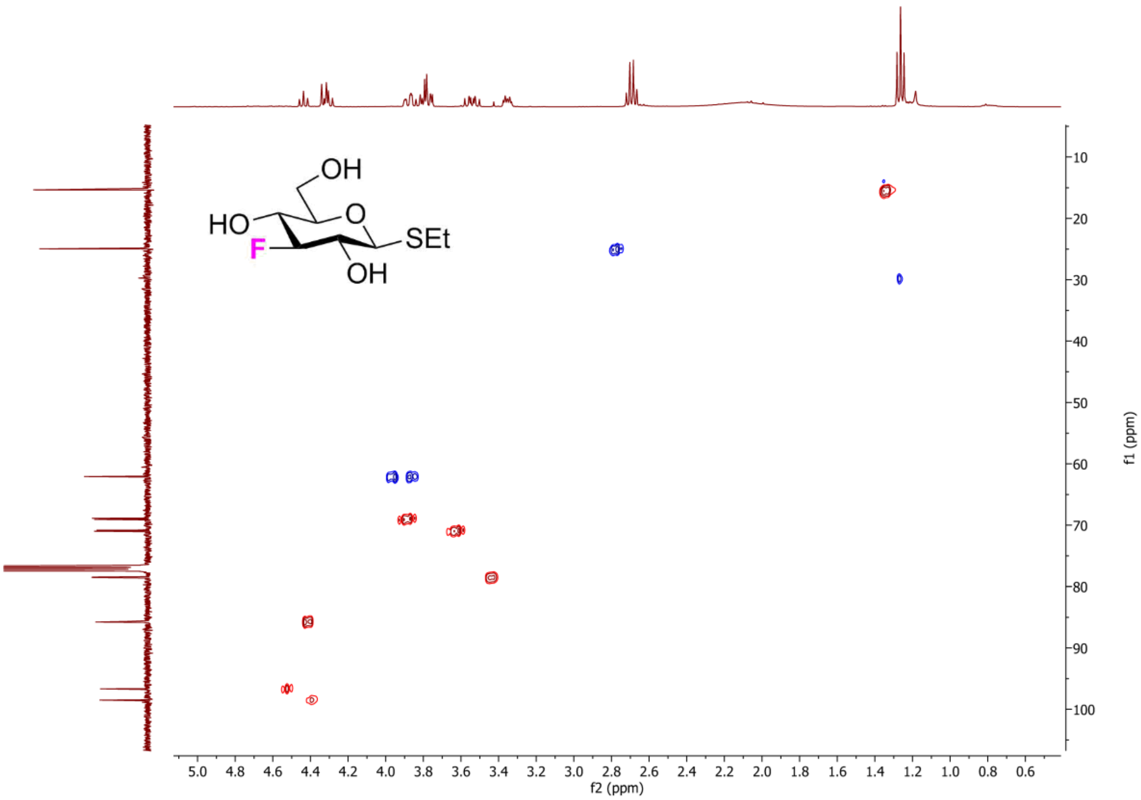

Ethyl 4,6-*O*-benzylidene-3-deoxy-3-fluoro-1-thio-β-D-glucopyranoside, 5-3

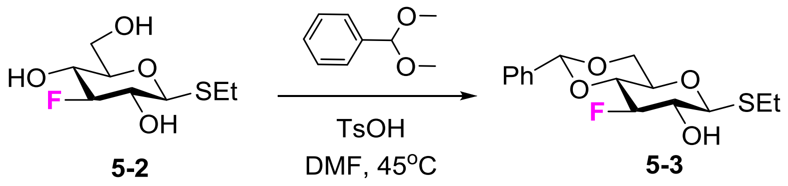

3-deoxy-3-fluoro-1-thio-β-D-glucopyranoside **5-2** (0.79 g, 3.5 mmol) was dissolved in dimethylformamide (DMF) and a catalytic amount of *p*-toluensulfonic acid monohydrate (100 mg, 0.5 mmol) was added to the mixture. Benzaldehyde dimethyl acetal (1.1 mL, 7.3 mmol) was then added dropwise at RT to the stirred solution. The reaction was heated to 45°C for 3h, after which time it was quenched with triethylamine (1 mL) at 0°C. The reaction mixture was diluted with EtOAc and washed three times with $NaHCO_3$ sat. aq. solution and once with brine. The organic layer was dried over Na_2SO_4 and concentrated under reduced pressure. The sticky oil was recrystallized at 0°C using a mixture of hexane and DCM to yield **5-3** as a white solid (0.87 g, 79%). ^{1}H NMR (400 MHz, $CDCl_3$) δ 7.55 – 7.46 (m, 2H), 7.42 -7.33 (m, 3H), 5.57 (s, 1H), 4.65 (dt, *J* = 53.5, 8.7 Hz, 1H), 4.47 (d, *J* = 9.8 Hz, 1H), 4.39 (ddd, *J* = 10.6, 5.0, 2.1 Hz, 1H), 3.84 – 3.68 (m, 3H), 3.54 – 3.47 (m, 1H), 2.84 – 2.72 (m, 2H), 1.34 (t, *J* = 7.4 Hz, 3H); ^{13}C NMR (101 MHz, $CDCl_3$) δ 136.69, 129.44, 128.49, 126.31, 101.77, 93.28 (d, *J* = 189.8 Hz), 86.57 (d, *J* = 7.9 Hz), 78.87 (d, *J* = 17.1 Hz), 72.07 (d, *J* = 18.0 Hz, C-2), 69.93 (d, *J* = 7.8 Hz, C-5), 68.56 (d, *J* = 1.4 Hz, C-6), 25.14, 15.43; ^{19}F NMR (564 MHz, $CDCl_3$) δ -192.99 (dt, *J* = 53.4, 12.5 Hz); R_f= 0.30 (Silica, Hexane : EtOAc = 3:1); $[\alpha]_D^{20}$ -32.92 (*c* 0.56 g/100 mL, $CHCl_3$); IR (neat) ν_{max} = 3467, 2928, 1075, 1110, 1020, 700 cm^{-1}; (ESI-HRMS+) m/z 337.0893 $[M+Na]^+$ ($C_{15}H_{19}FO_4SNa$ requires 337.0886).

^{1}H NMR of 5-3 (400 MHz, $CDCl_3$)

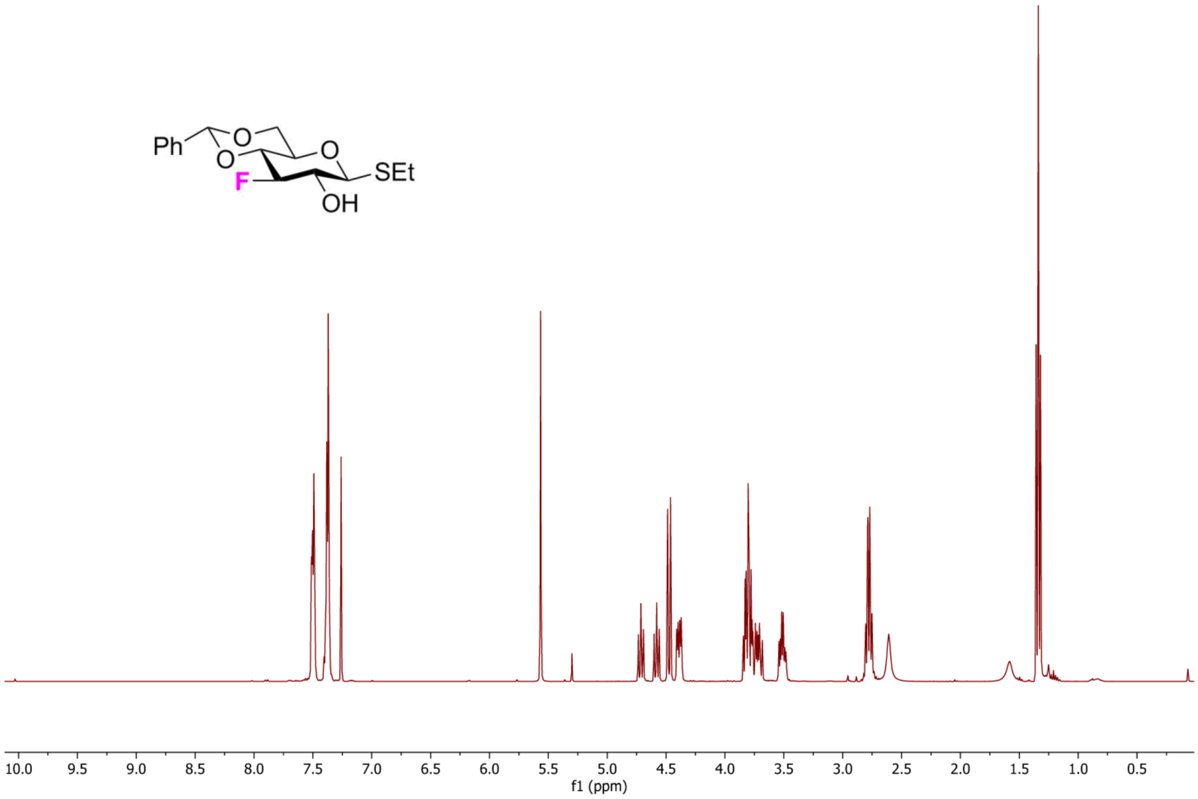

^{13}C NMR of 5-3 (101 MHz, $CDCl_3$)

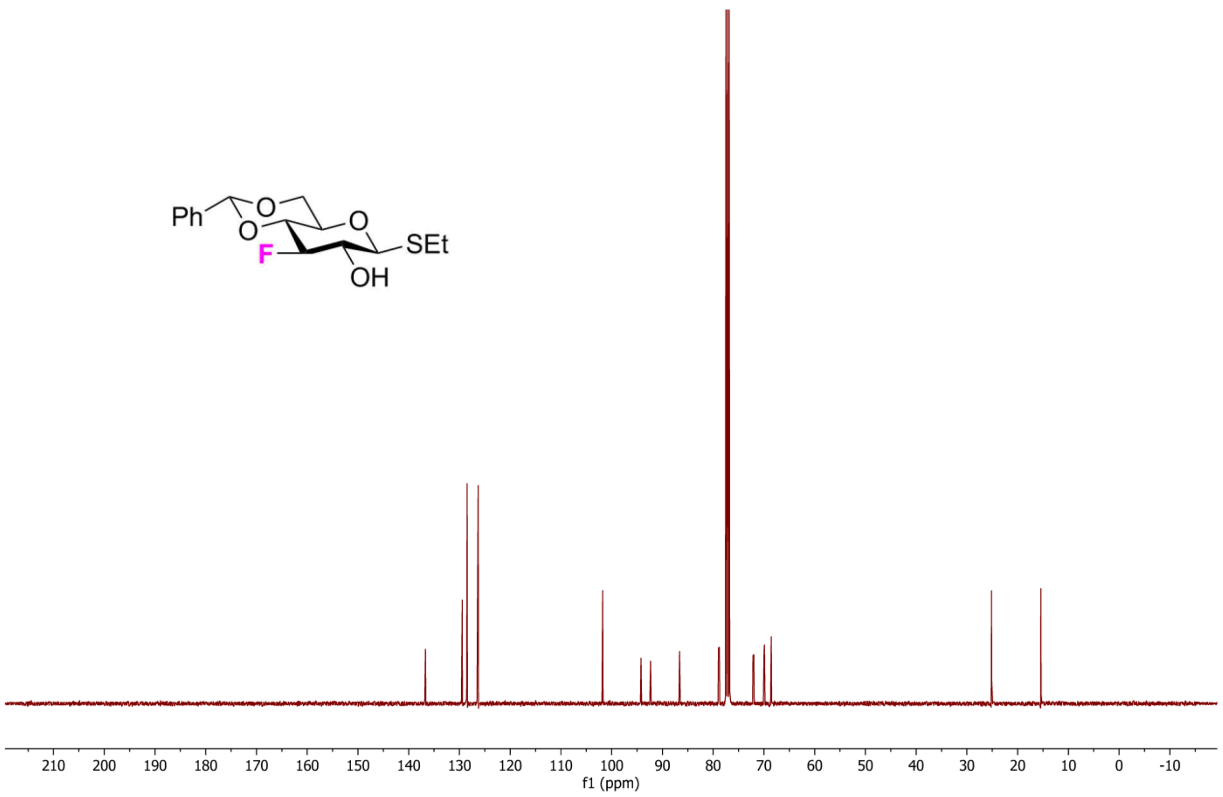

^{19}F NMR of 5-3 (564 MHz, $CDCl_3$)

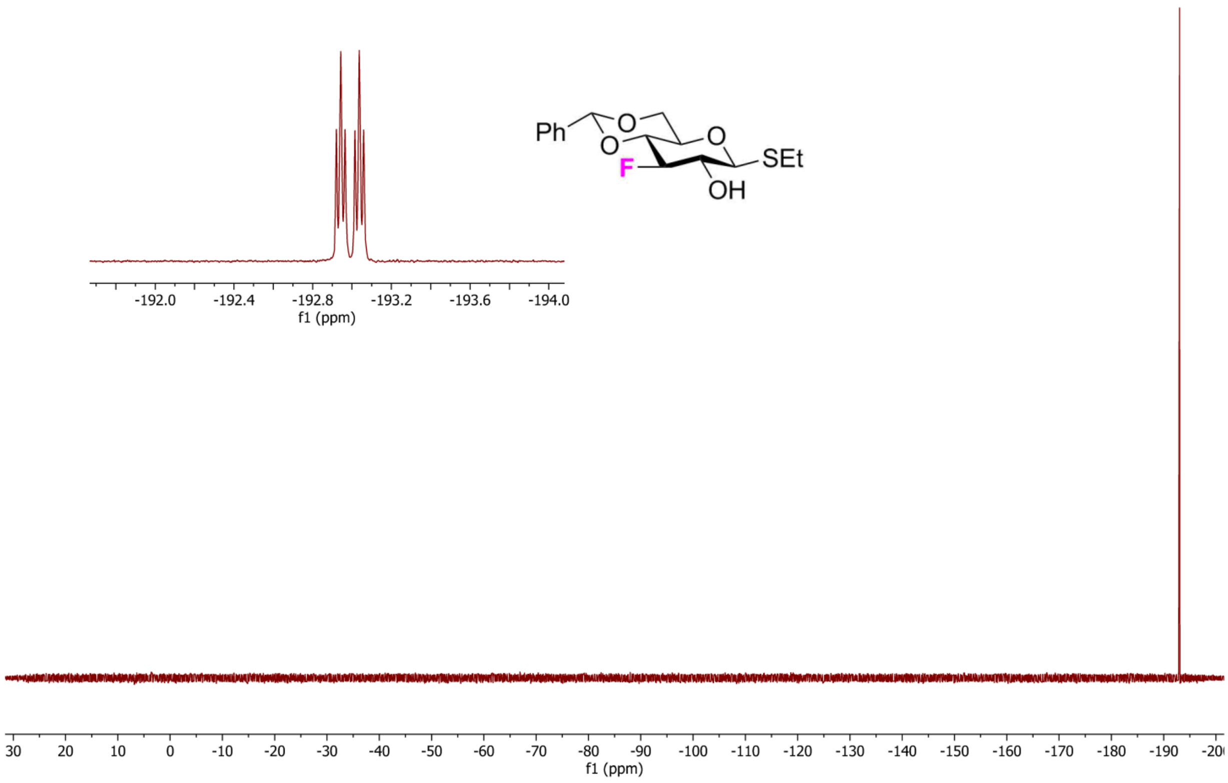

HSQC NMR of 5-3 ($CDCl_3$)

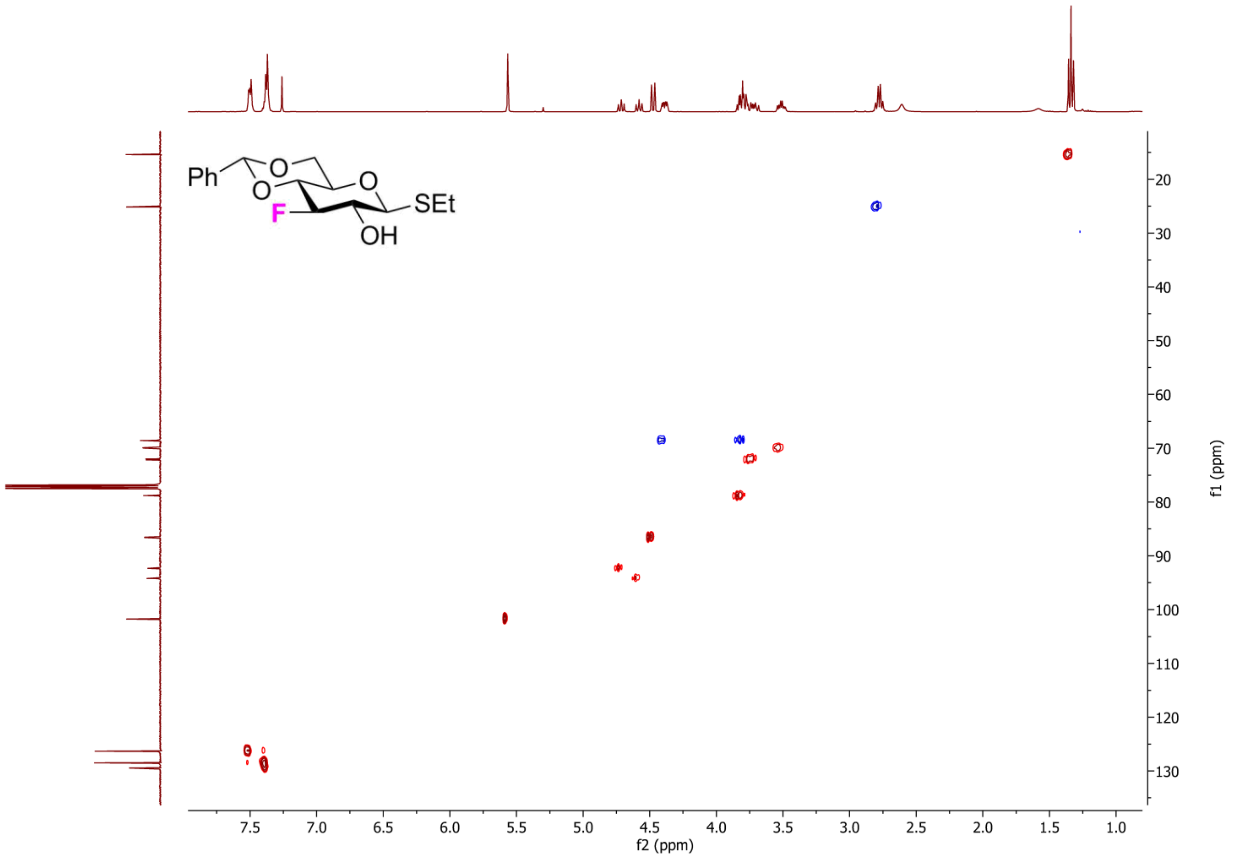

Ethyl 2-*O*-benzoyl-4,6-*O*-benzylidene-3-deoxy-3-fluoro-1-thio-β-D-glucopyranoside, 5-4

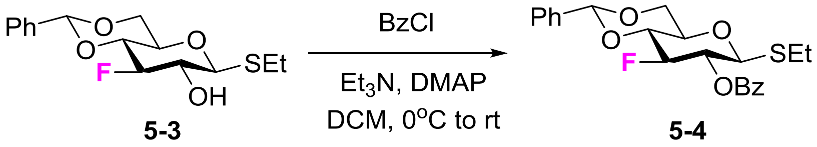

Ethyl 4,6-*O*-benzylidene-3-deoxy-3-fluoro-1-thio-β-D-glucopyranoside **5-3** (0.93 g, 3.0 mmol) was dissolved in anhydrous DCM (20 mL). Triethylamine (1.2 mL, 8.6 mmol) and 4-dimethylaminopyridine (DMAP) (112 mg, 0.9 mmol) were added to the solution, while stirring. Benzoyl chloride (520 µL, 4.5 mmol) was slowly added at 0°C and the reaction allowed to RT. Upon completion (18h), the reaction was quenched with sat. aq. solution of $NaHCO_3$. The mixture was washed three times with sat. aq. solution of $NaHCO_3$ and one time with brine. The organic layer was dried over Na_2SO_4 and concentration under reduced pressure. The crude product was purified using flash chromatography (Hexane : EtOAc = 5:1→4:1) followed by recrystallization from Hexane : EtOAc to yield **5-4** as a white solid (1.06 g, 85%). ^{1}H NMR (600 MHz, $CDCl_3$) δ 8.12 – 8.05 (m, 2H), 7.63 – 7.57 (m, 1H), 7.55 – 7.45 (m, 4H), 7.43 – 7.34 (m, 3H), 5.61 (s, 1H), 5.46 (ddd, *J* = 13.3, 10.1, 8.5 Hz, 1H), 4.86 (dt, *J* = 53.4, 8.9 Hz, 1H), 4.68 (d, *J* = 10.1 Hz, 1H), 4.44 (ddd, *J* = 10.6, 5.0, 1.9 Hz, 1H), 3.92 (dt, *J* = 11.1, 9.4 Hz, 1H), 3.86 (t, *J* = 10.3 Hz, 1H), 3.61 -3.57 (m, 1H), 2.81 – 2.68 (m, 2H), 1.25 (t, *J* = 7.4 Hz, 3H); ^{13}C NMR (151 MHz, $CDCl_3$) δ 165.19, 136.73, 133.60, 130.10, 129.48, 129.44, 128.61, 128.48, 126.33, 101.77, 91.63 (d, *J* = 193.5 Hz), 84.02 (d, *J* = 7.4 Hz), 79.05 (d, *J* = 17.2 Hz), 71.10 (d, *J* = 18.9 Hz), 70.05 (d, *J* = 7.6 Hz), 68.59, 24.37, 14.92; ^{19}F NMR (564 MHz, $CDCl_3$) δ -192.98 (dt, *J* = 53.5, 12.2 Hz); $[\alpha]_D^{20}$ -12.02 (*c* 1.17

g/100 mL, $CHCl_3$); IR (neat) ν_{max} = 1729, 1267, 1093, 990, 709 cm^{-1}; (ESI-HRMS+) m/z 441.1142 $[M+Na]^+$ ($C_{22}H_{23}FO_5SNa$ requires 441.1148).

^{1}H NMR of 5-4 (600 MHz, $CDCl_3$)

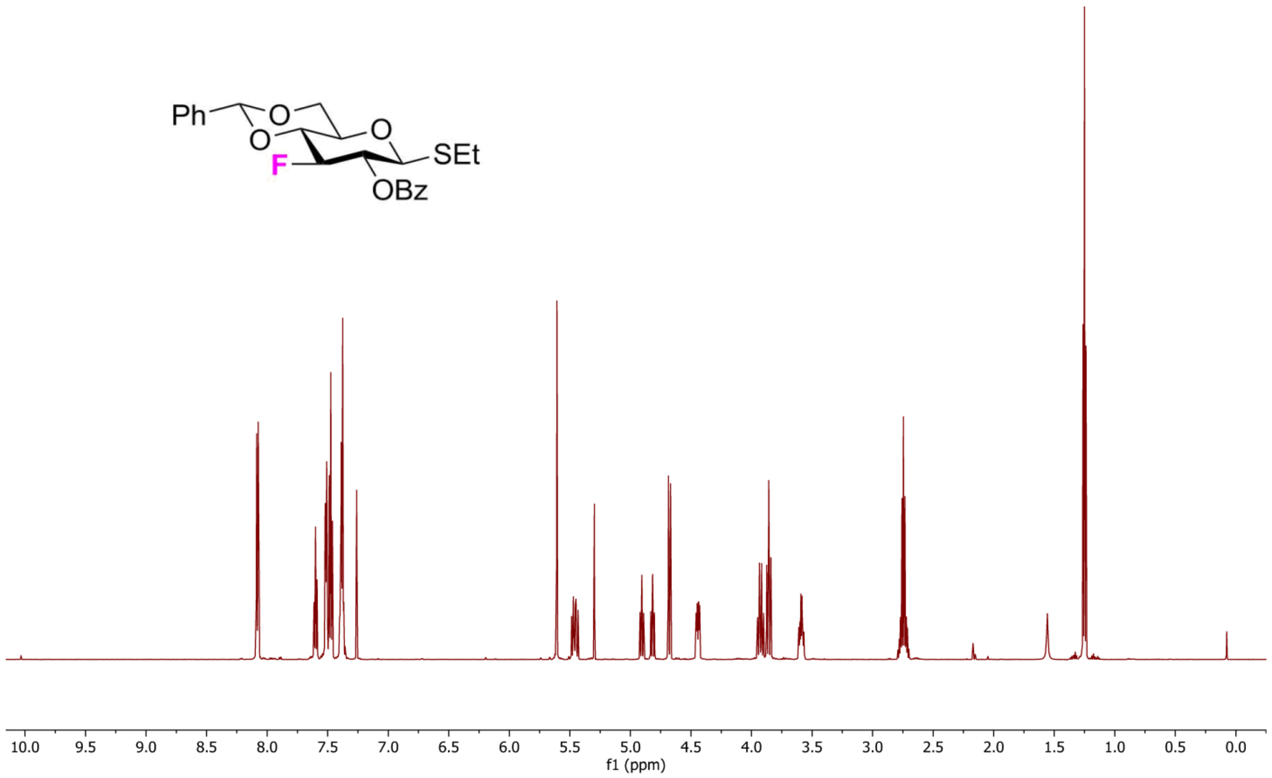

^{13}C NMR of 5-4 (151 MHz, $CDCl_3$)

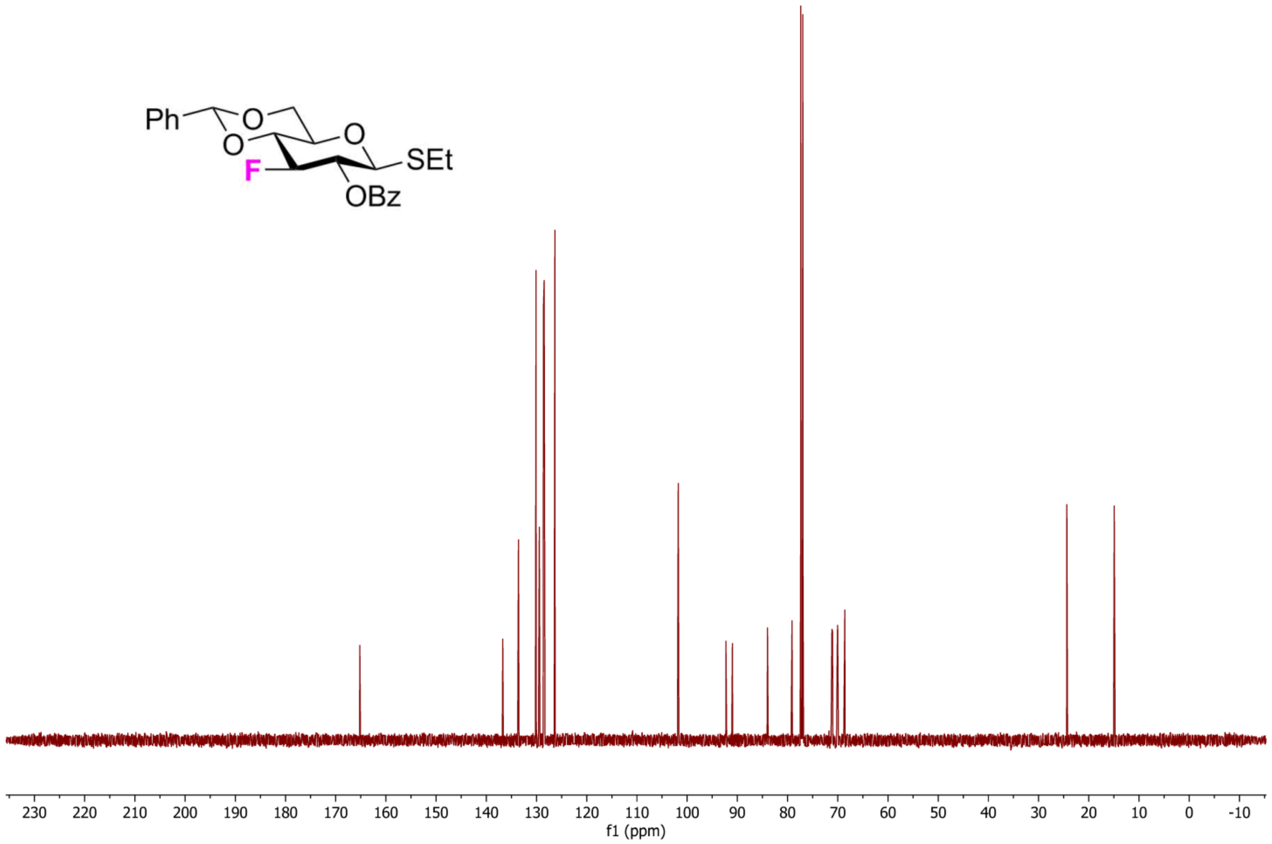

^{19}F NMR of 5-4 (564 MHz, CDCl$_3$)

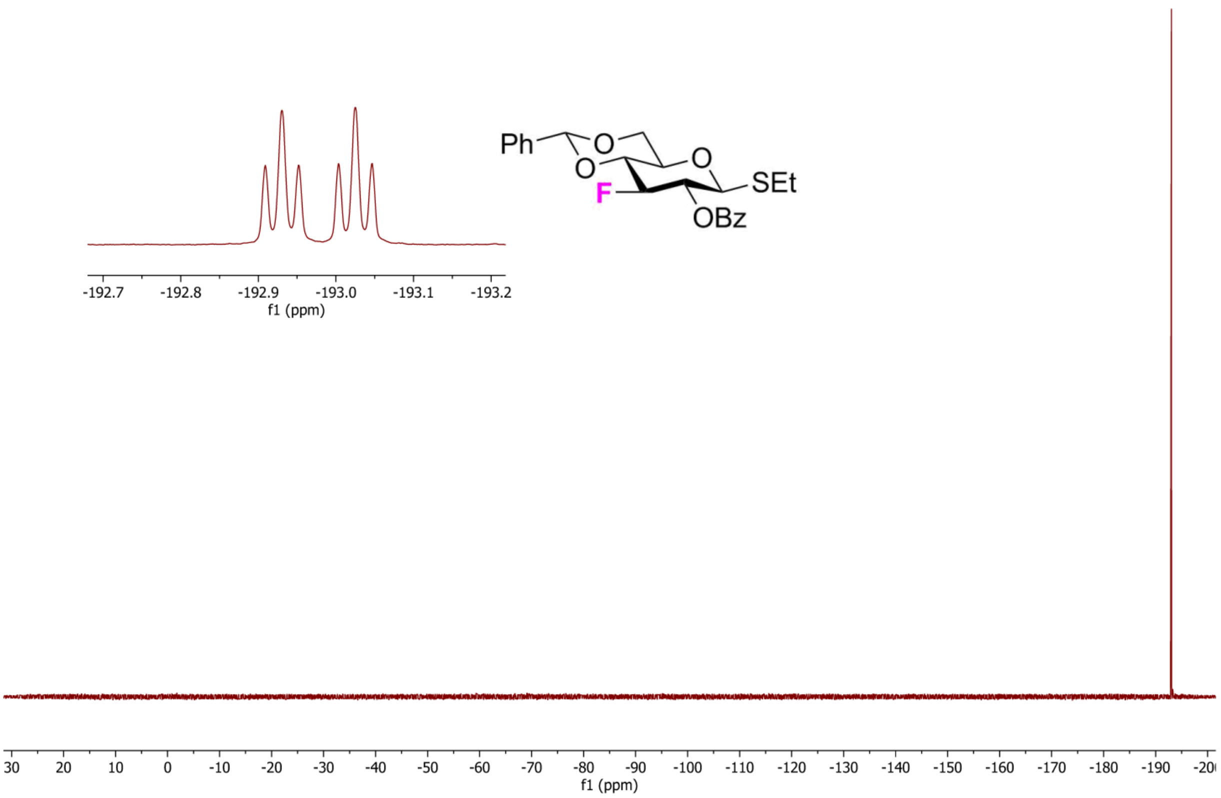

HSQC NMR of 5-4 (CDCl$_3$)

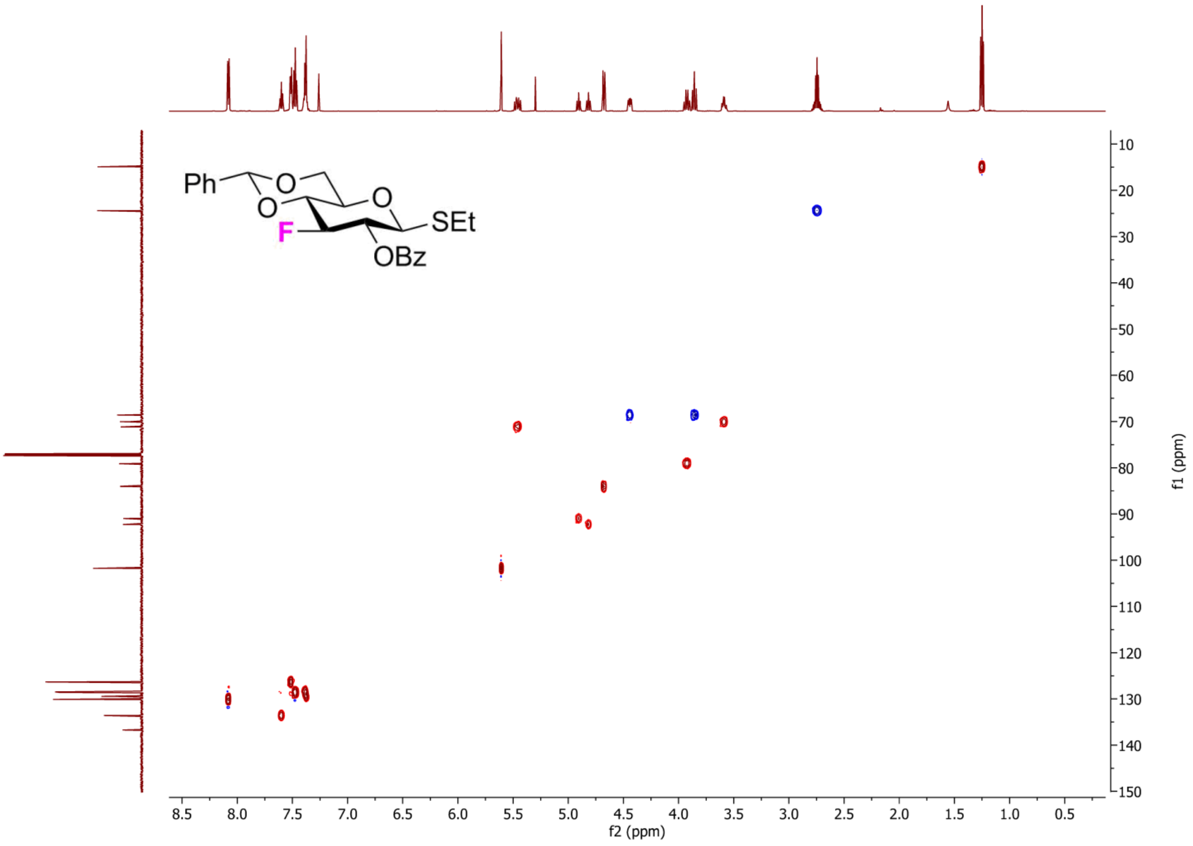

Ethyl 2-*O*-benzoyl-6-*O*-benzyl-3-deoxy-3-fluoro-1-thio-β-D-glucopyranoside, 5-5

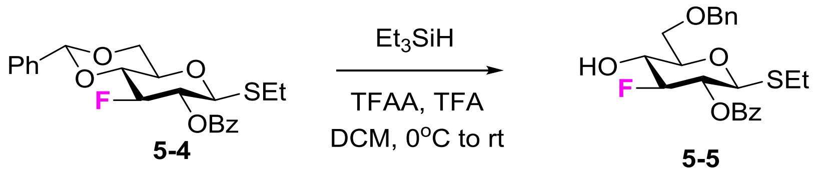

Ethyl 2-*O*-benzoyl-4,6-*O*-benzylidene-3-deoxy-3-fluoro-1-thio-β-D-glucopyranoside **5-4** (0.90 g, 2.1 mmol) was dissolved in DCM (20 mL). $HSiEt_3$ (2.0 mL, 12.5 mmol) and TFAA (300 µL, 2.2 mmol) were sequentially added to the stirred solution at 0°C. After 20 min, TFA (1.0 mL, 13 mmol) was added dropwise at 0°C. The reaction was allowed to rt and quenched with $NaHCO_3$ sat. aq. solution after 1h. The organic layer was washed twice with $NaHCO_3$ sat. aq. solution and once with brine. The crude product was purified by flash column chromatography with silica (Hexane : EtOAc = 5:1→3:1) to give **5-5** as a white solid (0.72 g, 80%). ^{1}H NMR (400 MHz, $CDCl_3$) δ 8.11 – 8.02 (m, 2H), 7.64 – 7.54 (m, 1H), 7.52 – 7.42 (m, 2H), 7.41 – 7.28 (m, 5H), 5.41 – 5.28 (m, 1H), 4.76 – 4.51 (m, 4H), 3.98 (dt, *J* = 13.8, 9.1 Hz, 1H), 3.82 (qd, *J* = 10.4, 4.6 Hz, 2H), 3.62 – 3.53 (m, 1H), 2.80 – 2.62 (m 2H), 1.24 (t, *J* = 7.5 Hz, 3H); ^{13}C NMR (101 MHz, $CDCl_3$) δ 165.21 , 137.44, 133.40, 129.95, 129.43, 128.58 , 128.45, 128.04, 127.86, 95.39 (d, *J* = 188.4 Hz), 82.91 (d, *J* = 8.0 Hz), 77.19 (d, *J* = 7.5 Hz), 73.82, 70.81 (d, *J* = 17.8 Hz), 70.25 (d, *J* = 18.1 Hz), 69.90 (d, *J* = 1.5 Hz), 24.09, 14.85; ^{19}F NMR (564 MHz, $CDCl_3$) δ -192.53 (dt, *J* = 52.7, 12.9 Hz); $[\alpha]_D^{20}$ -11.38 (*c* 0.89 g/100 mL, $CHCl_3$); IR (neat) ν_{max} = 3461, 2928, 1729, 1269, 1069, 710 cm^{-1}; (ESI-HRMS+) m/z 443.1307 $[M+Na]^+$ ($C_{22}H_{25}FO_5SNa$ requires 443.1304).

^{1}H NMR of 5-5 (400 MHz, $CDCl_3$)

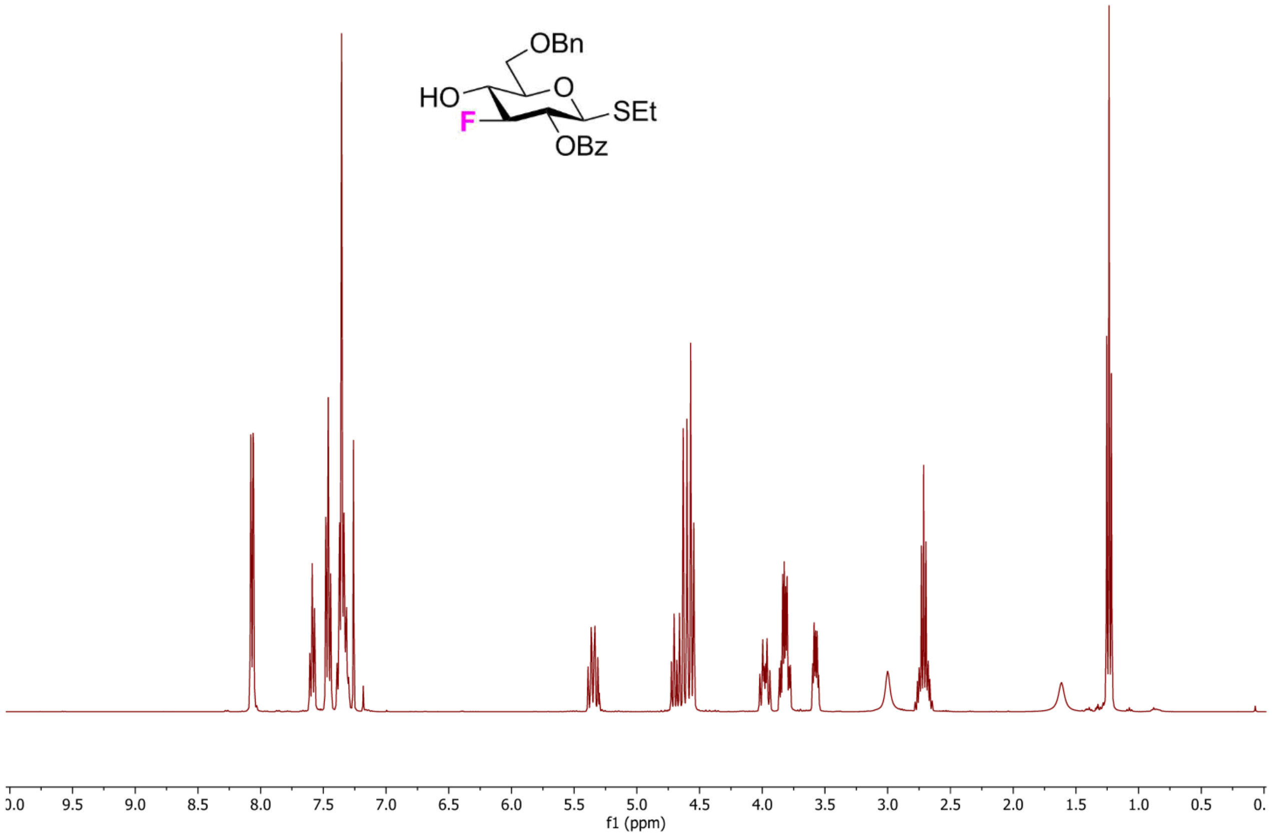

^{13}C NMR of 5-5 (101 MHz, $CDCl_3$)

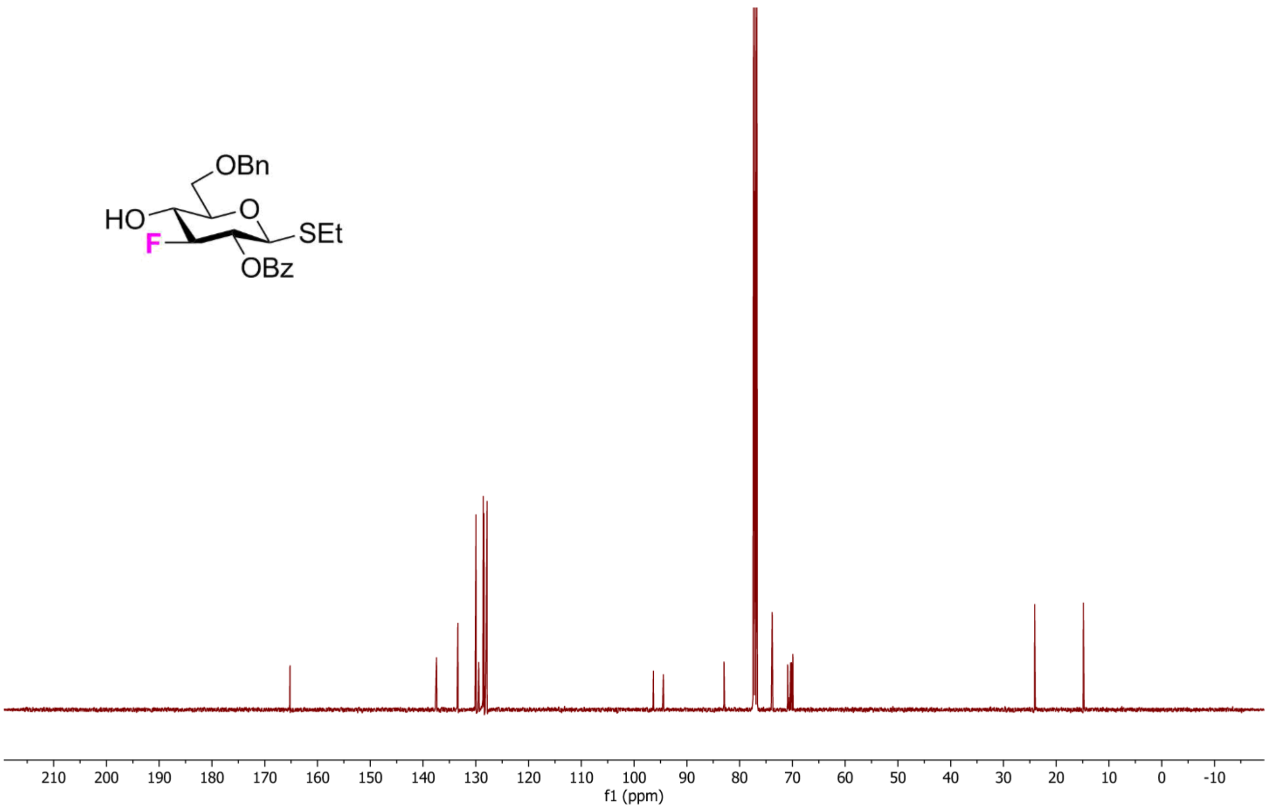

^{19}F NMR of 5-5 (564 MHz, $CDCl_3$)

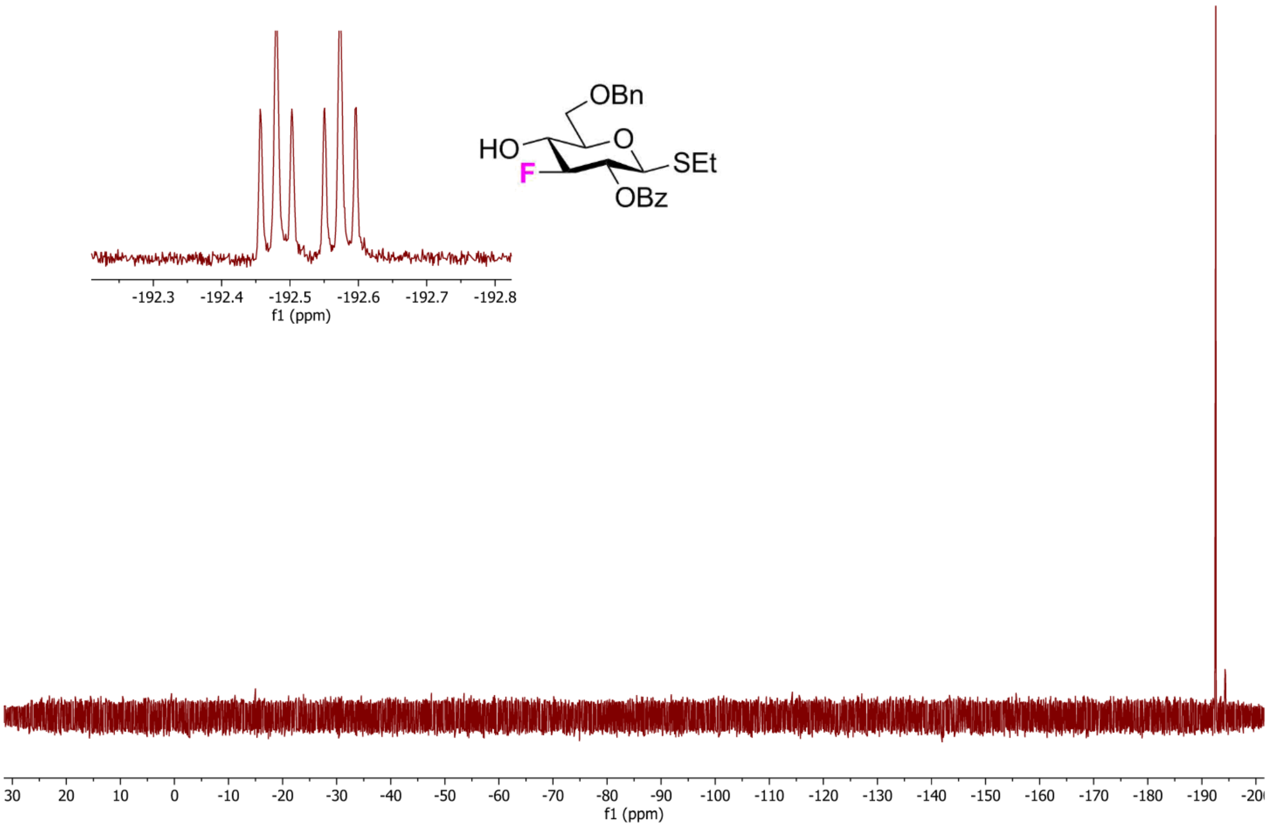

HSQC NMR of 5-5 ($CDCl_3$)

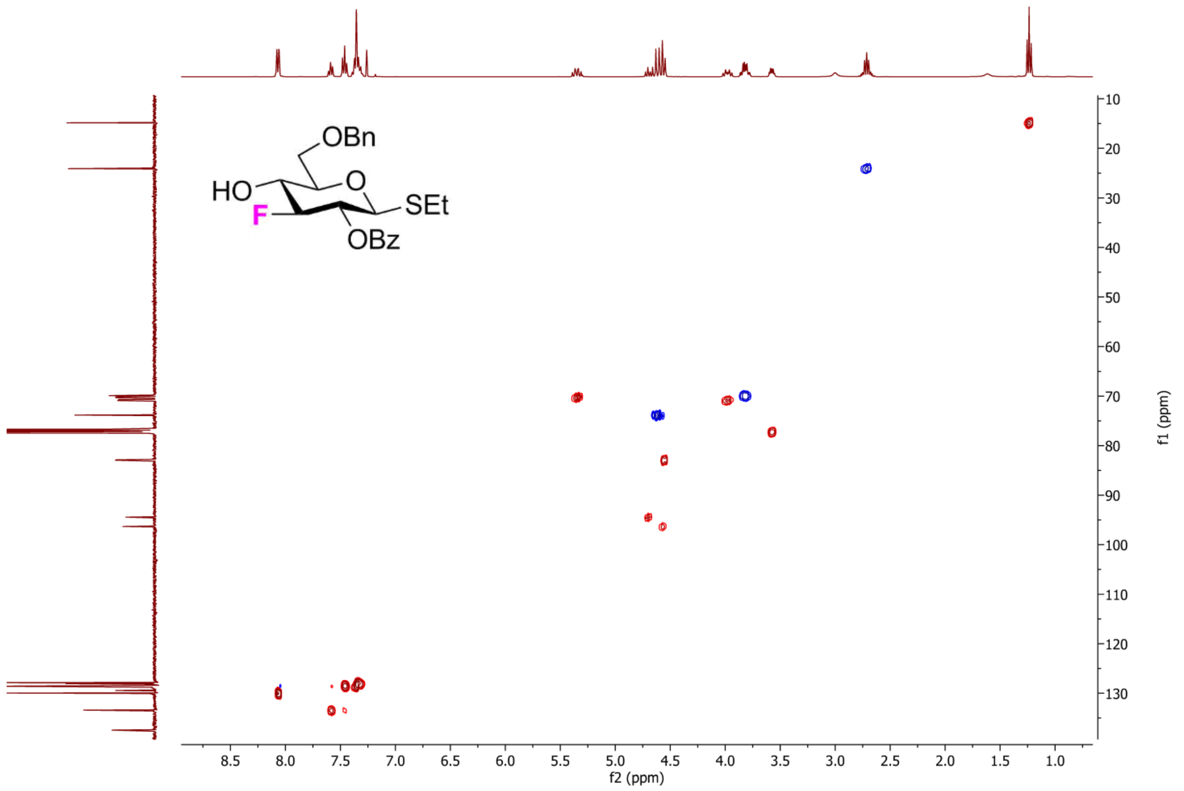

Ethyl 2-*O*-benzoyl-6-*O*-benzyl-4-*O*-(9-fluorenylmethoxycarbonyl)-3-deoxy-3-fluoro-1-thio-β-D-glucopyranoside, 5

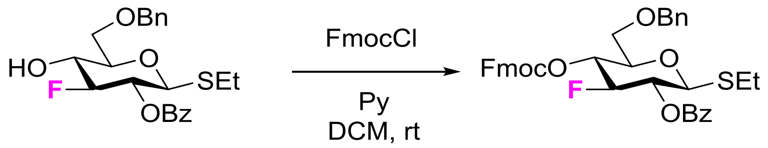

Ethyl 2-*O*-benzoyl-6-*O*-benzyl-3-deoxy-3-fluoro-1-thio-β-D-glucopyranoside **5-5** (0.62 g, 1.5 mmol) was dissolved in DCM (15 mL) and pyridine was added (350 µL, 4.3 mmol). FmocCl (771 mg, 3.0 mmol) was dissolved in DCM (2 mL) and added to the reaction mixture under Ar atmosphere. The yellow solution was stirred for 2h then quenched with a 1M solution of HCl. The organic layer was washed one time with 1M HCl, one time with sat. aq. solution of $NaHCO_3$ and one time with sat. aq. solution of NaCl. The crude compound was purified with flash column chromatography (Toluene : DCM = 4:1→1:1) followed by recrystallization from a mixture of Hexane : DCM to give **5** as a white solid (0.77 g, 81%). ^{1}H NMR (400 MHz, $CDCl_3$) δ 8.08 (d, *J* = 7.0 Hz, 2H), 7.77 (d, *J* = 7.6 Hz, 2H), 7.63 – 7.54 (m, 3H), 7.51 – 7.37 (m, 4H), 7.36 – 7.20 (m, 7H), 5.49 – 5.38 (m, 1H), 5.21 – 5.07 (m, 1H), 4.84 (dt, *J* = 52.3, 8.9 Hz, 1H), 4.65 – 4.50 (m, 3H), 4.44 (dd, *J* = 10.5, 7.5 Hz, 1H), 4.35 (dd, *J* = 10.5, 7.3 Hz, 1H), 4.21 (t, *J* = 7.4 Hz, 1H), 3.79 – 3.72 (m, 1H), 3.72 – 3.65 (m, 2H), 2.83 – 2.66 (m, 2H), 1.26 (t, *J* = 7.4 Hz, 3H); ^{13}C NMR (151 MHz, $CDCl_3$) δ 165.08, 154.17, 143.26, 143.24, 141.45, 141.41, 137.81, 133.57, 130.09, 129.47, 128.59, 128.49, 128.08, 127.86, 127.79, 127.37, 127.32, 120.23, 120.21, 92.79 (d, *J* = 193.1 Hz), 83.09 (d, *J* = 7.7 Hz), 76.77 (d, *J* = 6.4 Hz), 73.79, 73.66 (d, *J* = 18.0 Hz), 70.53

(d, J = 18.2 Hz), 70.50, 69.16 (d, J = 1.1 Hz), 46.78, 24.25, 14.98; ^{19}F NMR (564 MHz, $CDCl_3$) δ -191.98 (dt, J = 52.5, 12.4 Hz); R_f = 0.62 (Silica, Hexane : EtOAc = 3:1); $[\alpha]_D^{20}$ +16.54 (c 1.08 g/100 mL, $CHCl_3$); IR (neat) ν_{max} = 2929, 1757, 1733, 1248, 740, 710 cm^{-1}; (ESI-HRMS+) m/z 665.1992 $[M+Na]^+$ ($C_{37}H_{35}FO_7SNa$ requires 665.1985).

^{1}H NMR of 5 (400 MHz, $CDCl_3$)

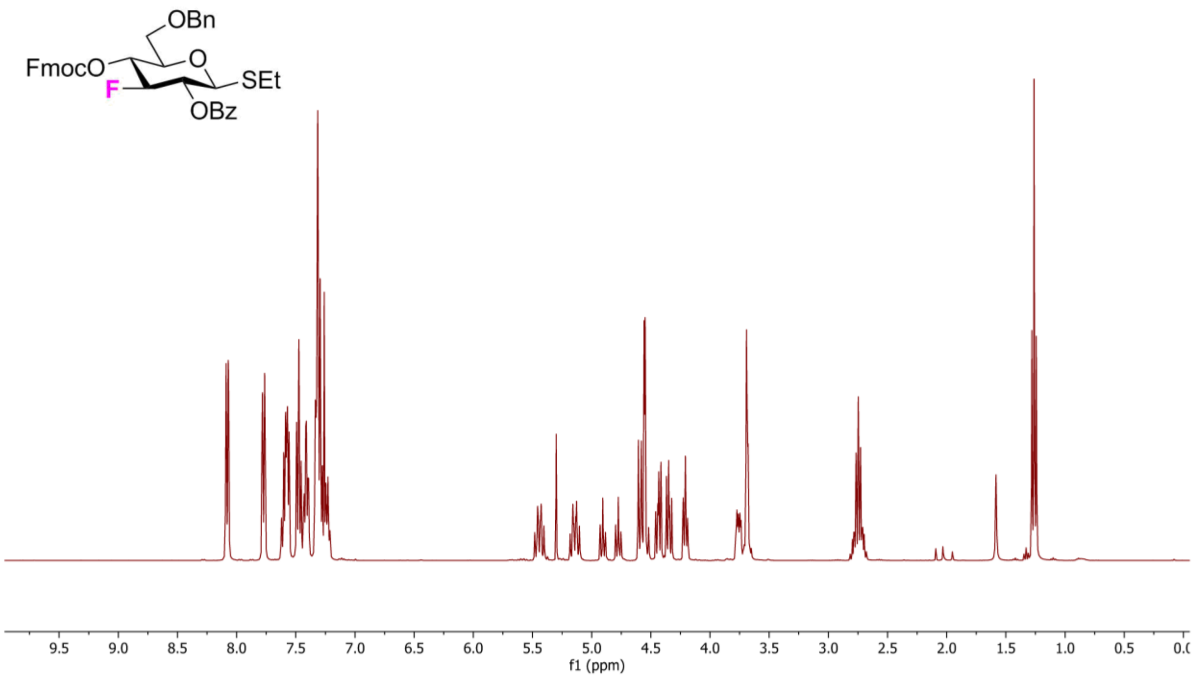

^{13}C NMR of 5 (151 MHz, $CDCl_3$)

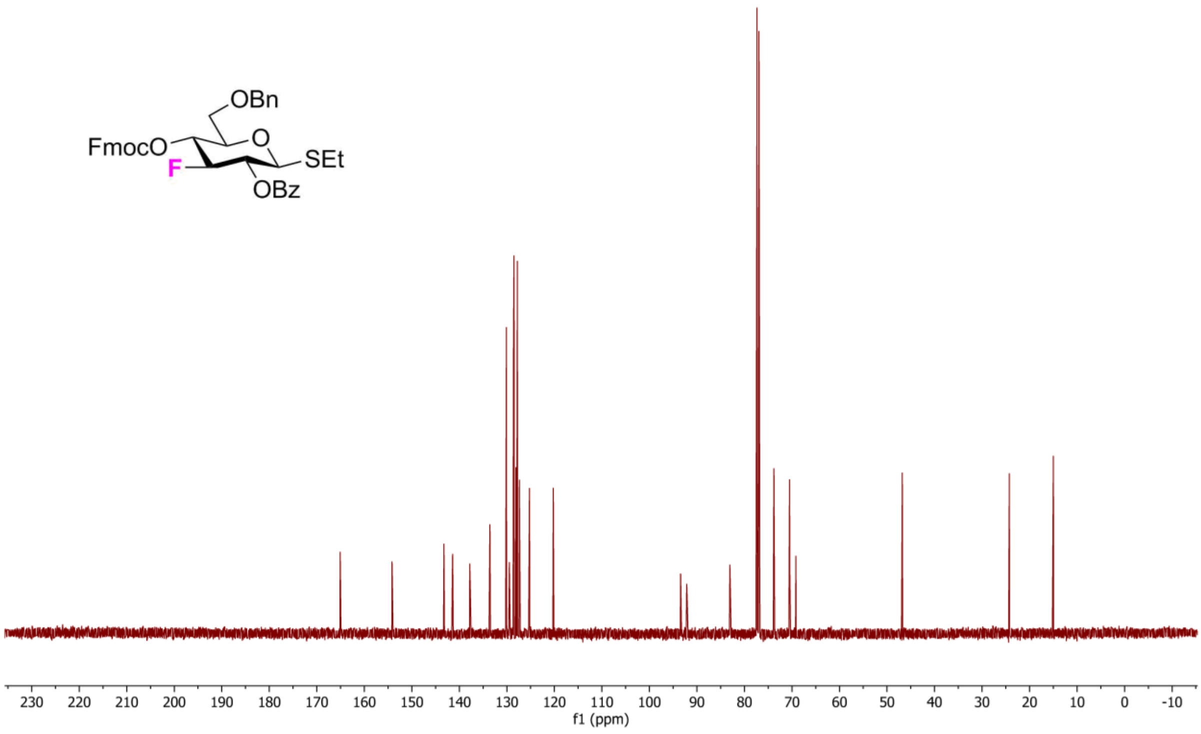

^{19}F NMR of 5 (564 MHz, $CDCl_3$)

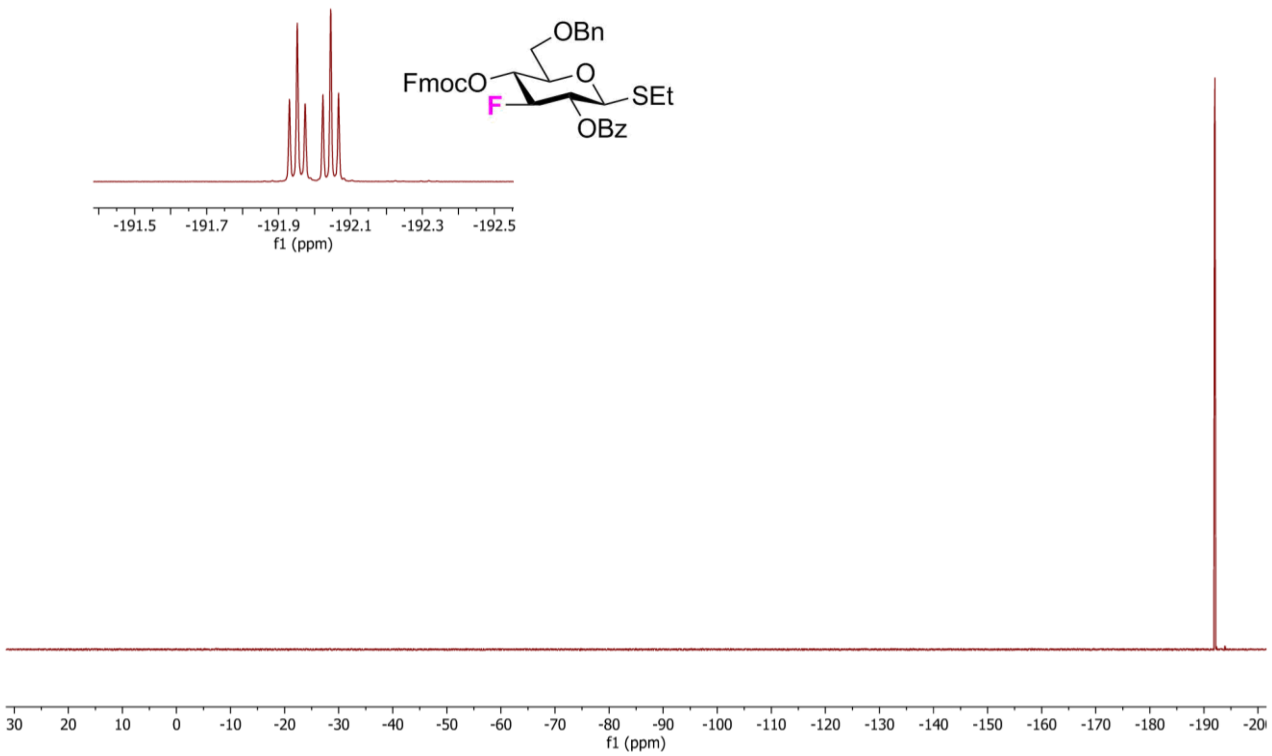

HSQC NMR of 5 ($CDCl_3$)

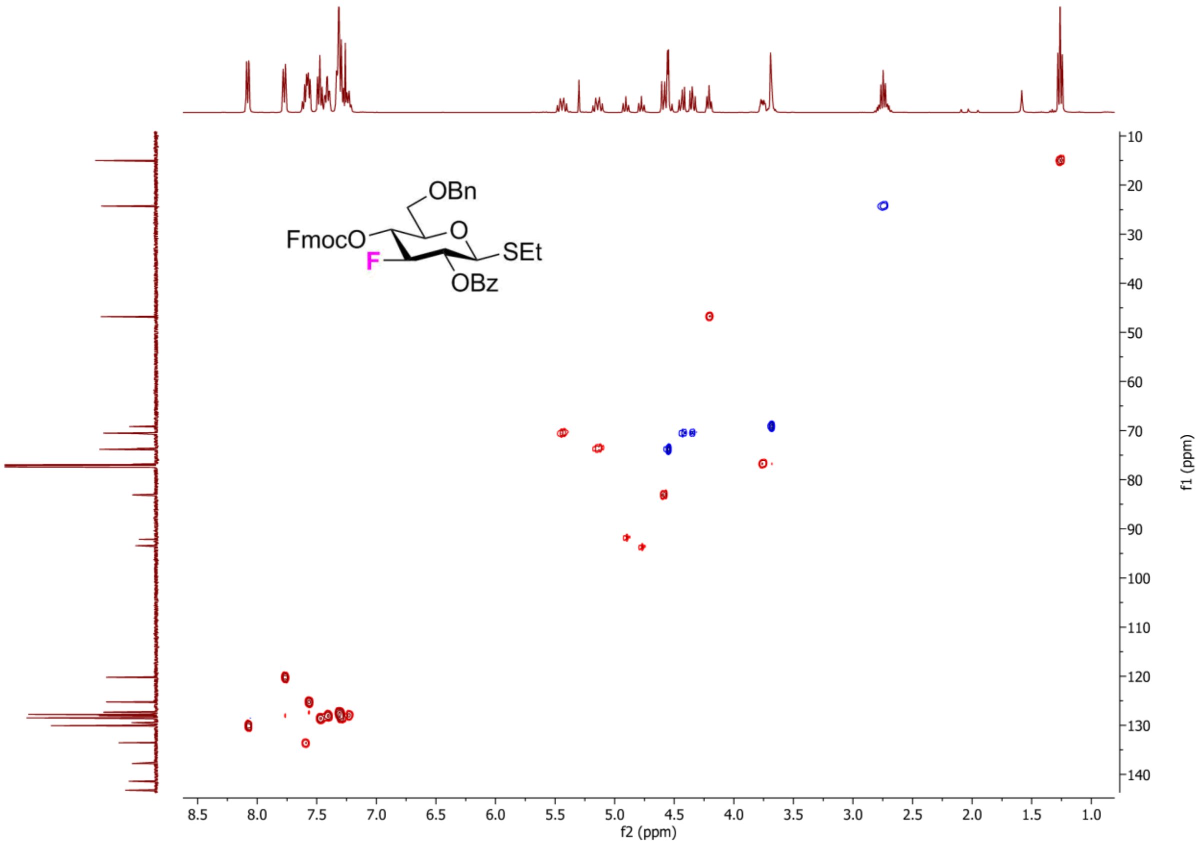

2.3. Synthesis of 6

Ethyl 3-*O*-methoxycarbonylmethyl-4,6-*O*-phenylmethylene-1-thio-β-D-glucopyranoside, 6-1

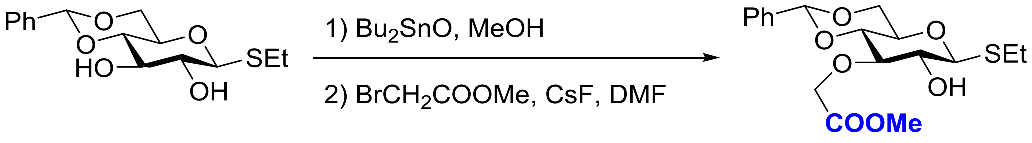

6-0

6-1

6-0 was prepared according to previously established procedures.[4]

Ethyl 4,6-*O*-phenylmethylene-1-thio-β-D-glucopyranoside **6-1** (5.20 g, 16.6 mmol) was dissolved in MeOH (150 mL). Dibutyltin oxide (5.15 g, 20.7 mmol) was added. The reaction mixture was charged with nitrogen and refluxed for 18 h. The methanol was removed under reduced pressure and DMF (100 mL) was added. Cesium fluoride (3.40 g, 22.4 mmol) and methyl 2-bromoacetate (1.87 mL, 20.0 mmol) were added, and the reaction was stirred at room temperature for 24 h. DMF was removed under reduced pressure and DCM (100 mL) was added. 1M KF (100 mL) solution (aq) was used to wash the organic layer. The water layer was extracted with additional DCM (50 mL). The organic layers were combined and dried with Na_2SO_4 and evaporated. The resulting crude product was purified by column chromatography (Hexane : EtOAc = 4:1→2:1) to give **6-1** as a colorless oil (4.0 g, 63 %). ^{1}H NMR (400 MHz, $CDCl_3$) δ 7.48 (m, 2H), 7.40 (m, *J* = 5.1, 2.1 Hz, 3H), 5.55 (s, 1H), 4.53 (d, *J* = 9.6 Hz, 1H), 4.50 – 4.33 (m, 3H), 3.89 – 3.61 (m, 6H), 3.59 – 3.53 (m, 1H), 3.53 – 3.42 (m, 1H), 2.79 (qd, *J* = 7.4, 2.3 Hz, 2H), 1.34 (t, *J* = 7.5 Hz, 3H); ^{13}C NMR (101 MHz, $CDCl_3$) δ 173.00, 137.01, 129.17, 128.34, 126.00, 101.34, 86.32, 84.19, 80.99, 72.06, 70.37, 68.81, 68.62, 52.37, 24.58, 15.11; $[\alpha]_D^{25}$ -10.23 (c = 1, $CHCl_3$); IR (neat) ν_{max} = 1736, 1083, 700 cm^{-1}; m/z (HRMS$^+$) 407.1143 $[M + Na]^+$ ($C_{18}H_{24}O_7SNa^+$ requires 407.1135).

^{1}H NMR of 6-1 (400 MHz, $CDCl_3$)

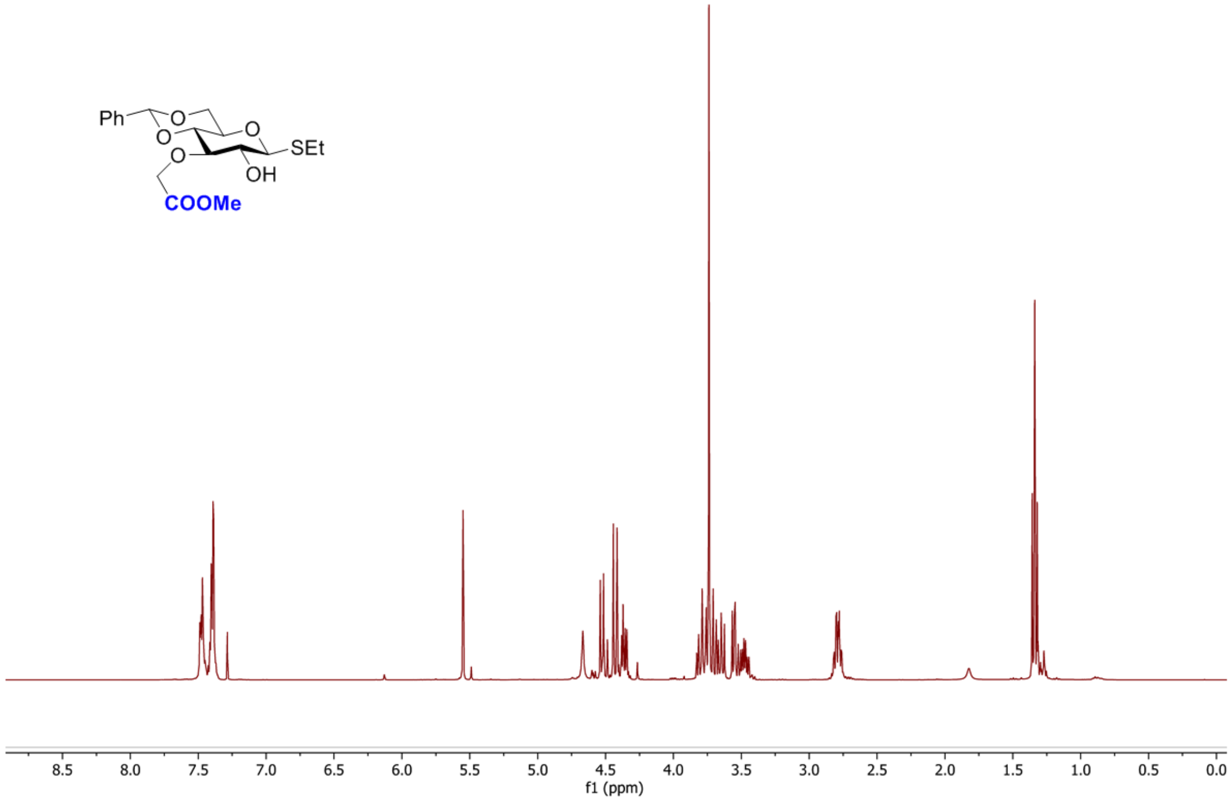

^{13}C NMR of 6-1 (101 MHz, $CDCl_3$)

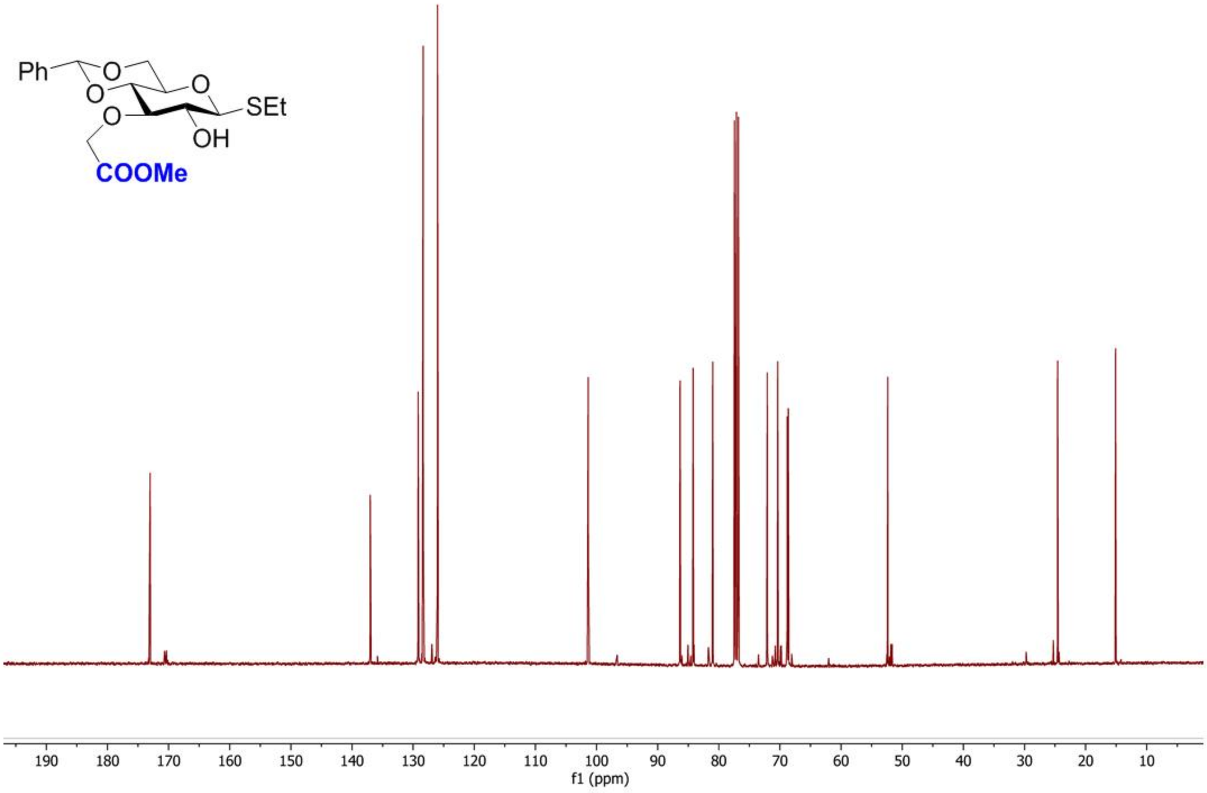

Ethyl 2-*O*-benzoyl-3-*O*-methoxycarbonylmethyl-4,6-*O*-phenylmethylene-1-thio-β-D-glucopyranoside, 6-2

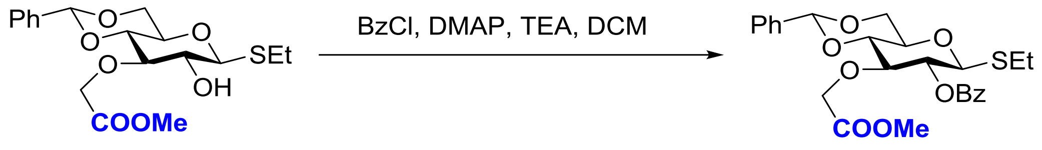

6-1 **6-2**

Ethyl 3-*O*-methoxycarbonylmethyl-4,6-*O*-phenylmethylene-1-thio-β-D-glucopyranoside **6-1** (4.00 g, 10.4 mmol) was dissolved in anhydrous DCM (80 mL). TEA (3.8 mL, 52.1 mmol) and 4-dimethylaminopyridine (0.25 g, 2.0 mmol) were added. Benzoyl chloride (1.9 mL, 16.3 mmol) was added dropwise to the solution cooled with an ice bath. After 30 min, the system was allowed to room temperature and the reaction stirred for additional 10 h. The reaction mixture was washed with water (50 mL), dried with Na_2SO_4 and evaporated. The resulting crude product was purified by column chromatography (Hexane : EtOAc = 5:1→3:1) to give **6-2** as a white solid (3.92 g, 77 %). ^{1}H NMR (400 MHz, $CDCl_3$) δ 8.17 – 8.09 (m, 2H), 7.66 – 7.57 (m, 1H), 7.54 – 7.44 (m, 4H), 7.40 (dd, *J* = 5.0, 2.0 Hz, 3H), 5.58 (s, 1H), 5.38 (dd, *J* = 10.1, 8.5 Hz, 1H), 4.70 (d, *J* = 10.1 Hz, 1H), 4.43 (dd, *J* = 10.5, 5.0 Hz, 1H), 4.35 (d, *J* = 2.3 Hz, 2H), 3.97 (t, *J* = 8.9 Hz, 1H), 3.89 (t, *J* = 8.3 Hz, 1H), 3.85 (t, *J* = 9.4 Hz, 1H), 3.59 (td, *J* = 9.7, 5.0 Hz, 1H), 3.37 (s, 3H), 2.76 (qd, *J* = 7.5, 4.4 Hz, 2H), 1.26 (t, *J* = 7.4 Hz, 3H); ^{13}C NMR (101 MHz, $CDCl_3$) δ 170.46, 165.22, 136.86, 133.22, 129.96, 129.82, 129.21, 128.42, 128.34, 126.03, 101.38, 84.17, 81.44, 81.42, 71.27, 70.44, 69.45, 68.63, 51.52, 23.98, 14.82; $[\alpha]_D^{25}$ -26.67 (c = 1, $CHCl_3$); IR (neat) ν_{max} = 1728, 1267, 1094 cm^{-1}; m/z ($HRMS^+$) 511.1403 $[M + Na]^+$ ($C_{25}H_{28}O_8SNa^+$ requires 511.1397).

^{1}H NMR of 6-2 (400 MHz, $CDCl_3$)

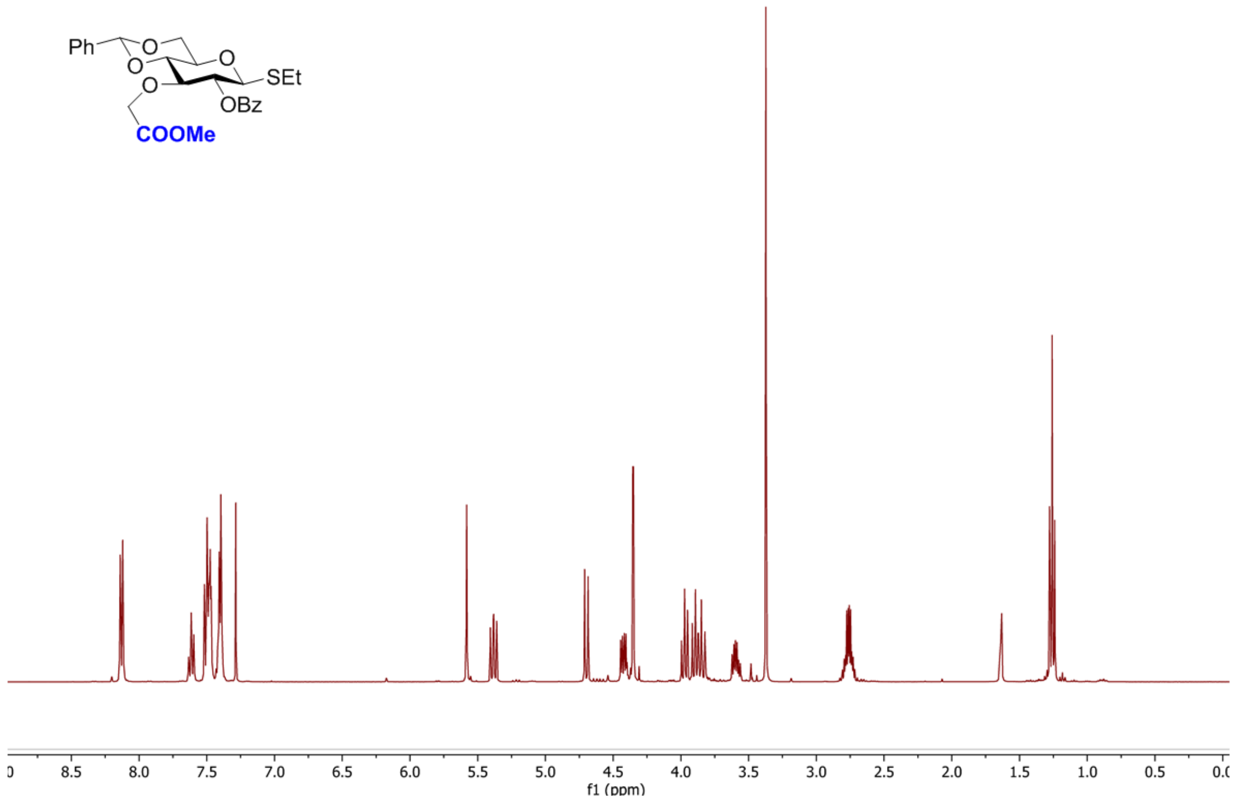

^{13}C NMR of 6-2 (101 MHz, $CDCl_3$)

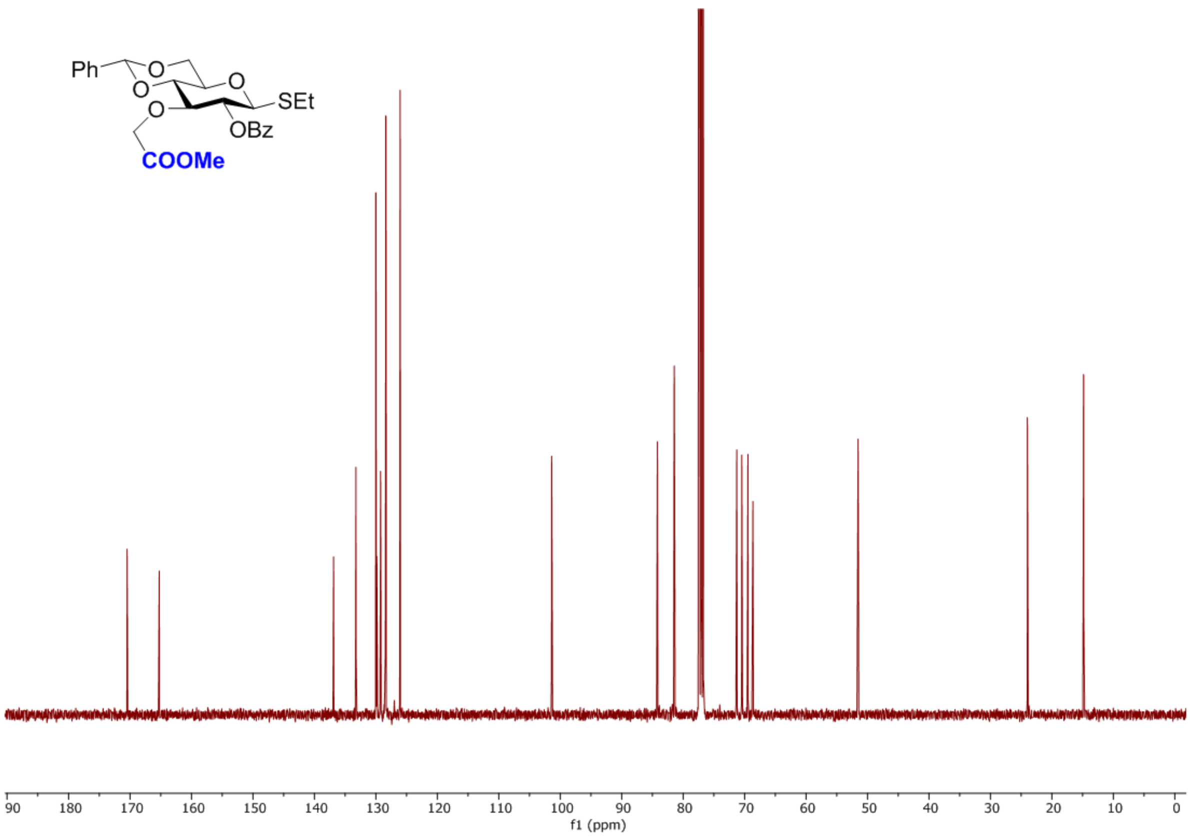

Ethyl 2-*O*-benzoyl-3-*O*-methoxycarbonylmethyl-6-*O*-benzyl-1-thio-β-D-glucopyranoside, 6-3

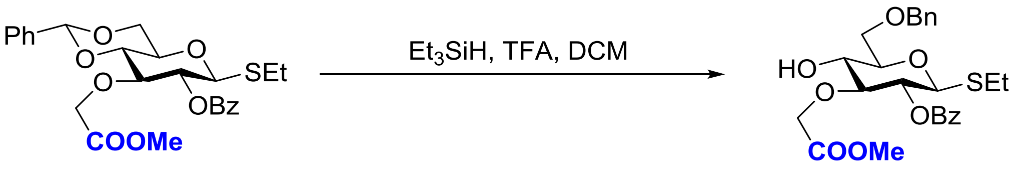

Ethyl 2-*O*-benzoyl-3-*O*-methoxycarbonylmethyl-4,6-*O*-phenylmethylene-1-thio-β-D-glucopyranoside **6-2** (3.92 g, 8.0 mmol) was dissolved in anhydrous DCM (70 mL). 4Å molecular sieves were added and the system was charged with nitrogen. Triethylsilane (13 mL, 81.4 mmol) was added and the mixture was stirred at room temperature for 30 min, and then cooled to 0°C. TFA (6.5 mL, 83.2 mmol) was added and the mixture was stirred for 1 h at 0°C. After the reaction was finished, molecular sieves were filtered and the organic phase was washed with saturated $NaHCO_3$ solution (aq) (100 mL, twice) and water (50 mL). The water layers were combined and extracted with DCM (50 mL). The organic layers were combined and dried over Na_2SO_4 and evaporated. The resulting crude product was purified by column chromatography (Hexane : EtOAc = 4:1→3:1) to give **6-3** as a colorless oil (3.31 g, 84 %). ^{1}H NMR (400 MHz, $CDCl_3$) δ 8.16 – 8.03 (m, 2H), 7.69 – 7.59 (m, 1H), 7.56 – 7.45 (m, 2H), 7.42 – 7.34 (m, 4H), 7.33 – 7.29 (m, 1H), 5.28 (dd, *J* = 10.1, 9.0 Hz, 1H), 4.95 (s, 1H), 4.66 (s, 2H), 4.56 (d, *J* = 10.0 Hz, 1H), 4.35 and 4.14 (ABq, *J* = 17.5 Hz, 2H), 3.95 (dd, *J* = 10.9, 2.2 Hz, 1H), 3.82 – 3.71 (m, 5H), 3.64 – 3.53 (m, 2H), 2.85 – 2.65 (m, 2H), 1.26 (t, *J* = 7.4 Hz, 3H); ^{13}C NMR (101 MHz, $CDCl_3$) δ 173.30,

165.11, 138.26, 133.49, 129.86, 129.47, 128.63, 128.38, 127.66, 127.59, 87.18, 83.39, 79.92, 73.55, 72.41, 69.83, 69.64, 68.62, 52.51, 24.16, 14.96; $[\alpha]_D^{25}$ -32.08 (c = 1, $CHCl_3$); IR (neat) ν_{max} = 1728, 1268, 1069 cm^{-1}; m/z ($HRMS^+$) 513.1559 $[M + Na]^+$ ($C_{25}H_{30}O_8SNa^+$ requires 513.1554).

^{1}H NMR of 6-3 (400 MHz, $CDCl_3$)

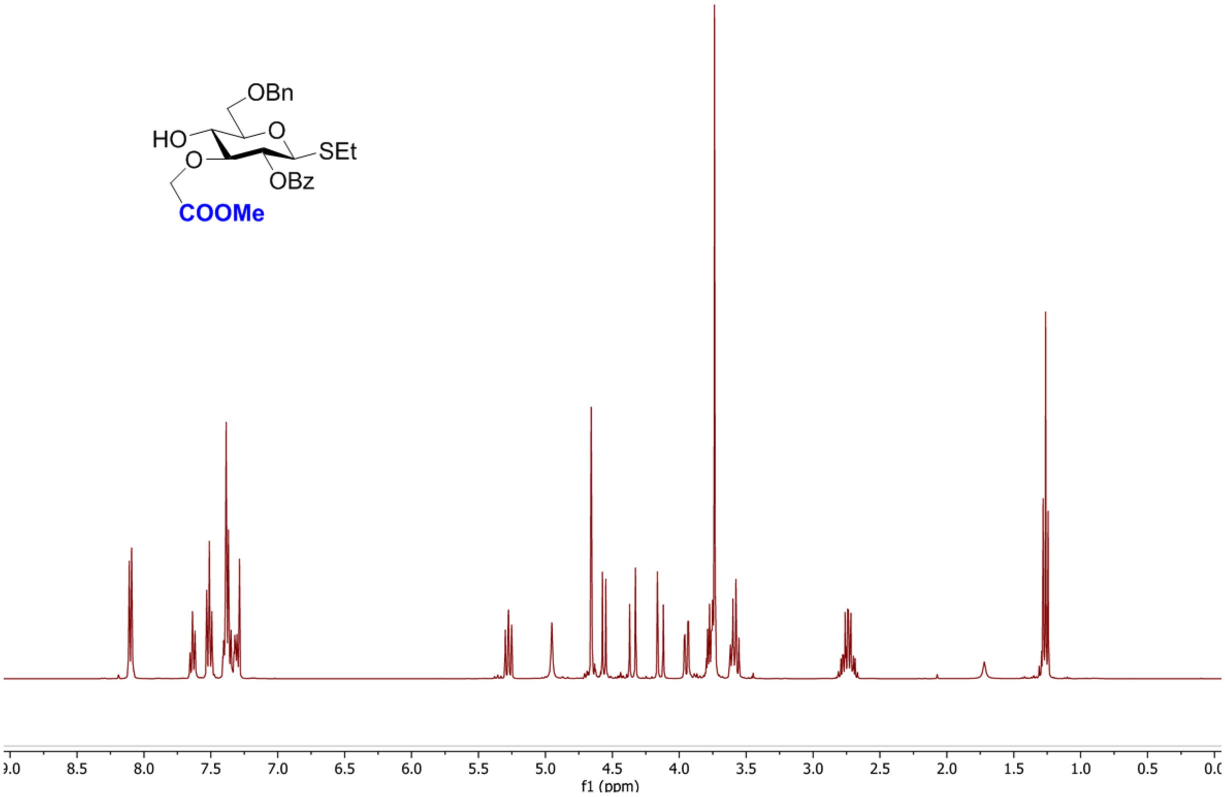

^{13}C NMR of 6-3 (101 MHz, $CDCl_3$)

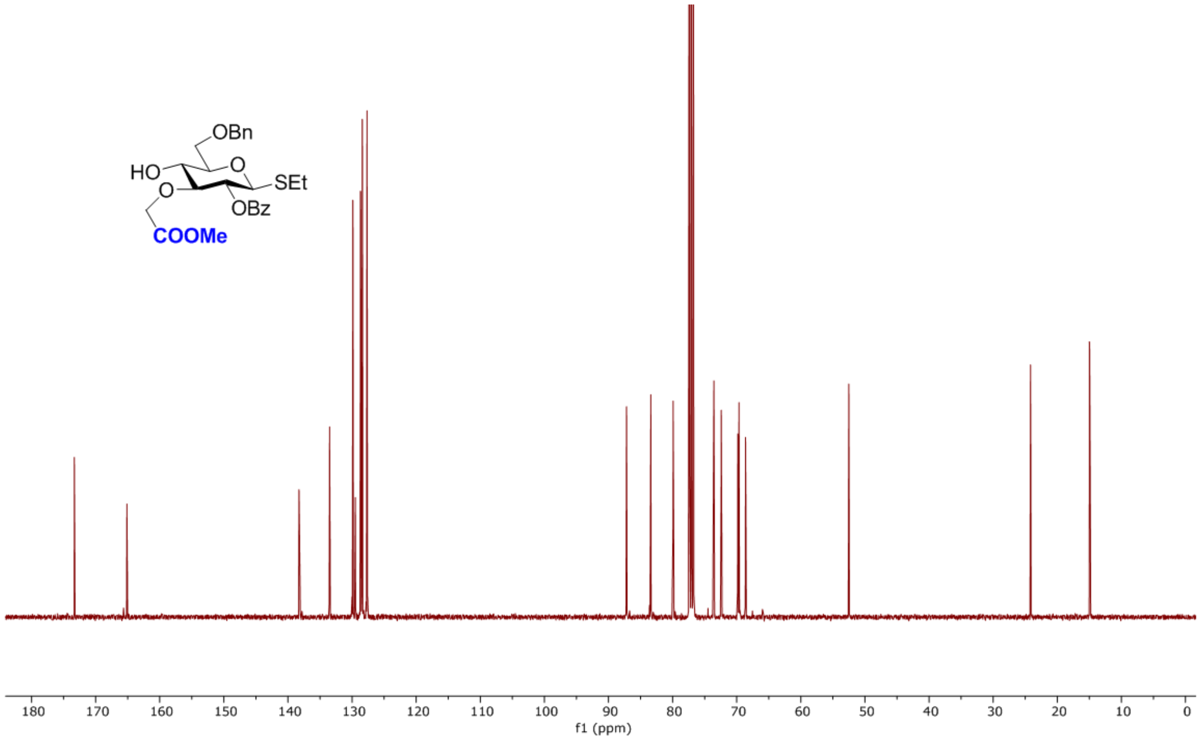

Ethyl 2-*O*-benzoyl-3-*O*-methoxycarbonylmethyl-4-*O*-(9-fluorenylmethoxycarbonyl)-6-*O*-benzyl-1-thio-β-D-glucopyranoside, 6

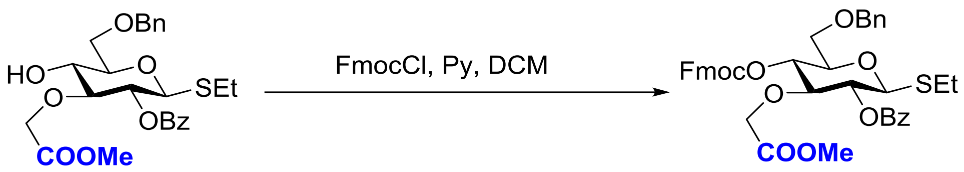

6-3 6

Ethyl 2-*O*-benzoyl-3-*O*-methoxycarbonylmethyl-6-*O*-benzyl-1-thio-β-D-glucopyranoside **6-3** (3.31 g, 6.7 mmol) was dissolved in anhydrous DCM (50 mL) and pyridine (2 mL, 24.8 mmol) was added. Then the solution was cooled with an ice bath for 15 min, after which time fluorenylmethyloxycarbonyl chloride (3.47 g, 13.4 mmol) was added slowly. The reaction was allowed to warm to room temperature and stirred for 6 h. After the reaction was finished, DCM (50 mL) was added and the organic phase was washed with aqueous citric acid (50 mL). After extracting the water phase with DCM (50 mL), the organic layers were combined and dried with Na_2SO_4. The solvent was evaporated and the resulting crude product was purified by column chromatography. (Hexane : EtOAc = 5:1→3:1) to give **6** as a white solid (3.80 g, 80 %). ^{1}H NMR (400 MHz, $CDCl_3$) δ 8.02 – 7.97 (m, 2H), 7.69 (d, *J* = 7.5 Hz, 2H), 7.59 – 7.50 (m, 3H), 7.40 (dd, *J* = 8.4, 7.1 Hz, 2H), 7.33 (t, *J* = 7.4 Hz, 2H), 7.27 – 7.20 (m, 6H), 7.18 – 7.12 (m, 1H), 5.30 – 5.22 (m, 1H), 4.94 (t, *J* = 9.6 Hz, 1H), 4.50 (d, *J* = 9.9 Hz, 1H), 4.48 (s, 2H), 4.38 (dd, *J* = 10.4, 7.3 Hz, 1H), 4.29 (dd, *J* = 10.5, 7.1 Hz, 1H), 4.19 – 4.09 (m, 3H), 3.85 (t, *J* = 9.2 Hz, 1H), 3.69 (ddd, *J* = 9.6, 5.1, 3.7 Hz, 1H), 3.65 – 3.57 (m, 2H), 3.33 (s, 3H), 2.65 (p, *J* = 7.4 Hz, 2H), 1.17 (t, *J* = 7.5 Hz, 3H); ^{13}C NMR (101 MHz, $CDCl_3$) δ 169.81, 164.96, 154.28, 143.33, 141.30 (d, *J* = 1.5 Hz), 137.81, 133.44, 129.90, 129.52, 128.55, 128.38, 127.92, 127.70, 127.67, 127.21, 127.18, 125.17, 125.15, 120.10, 83.61, 83.32, 74.43, 73.62, 71.68, 70.20, 69.48, 69.14, 51.74, 46.72, 24.16, 14.90; $[\alpha]_D^{25}$ 10.18 (c = 1, $CHCl_3$); IR (neat) ν_{max} = 1756, 1248, 1070 cm^{-1}; m/z ($HRMS^+$) 735.2244 $[M + Na]^+$ ($C_{40}H_{40}O_{10}SNa^+$ requires 735.2234).

^{1}H NMR of 6 (400 MHz, $CDCl_3$)

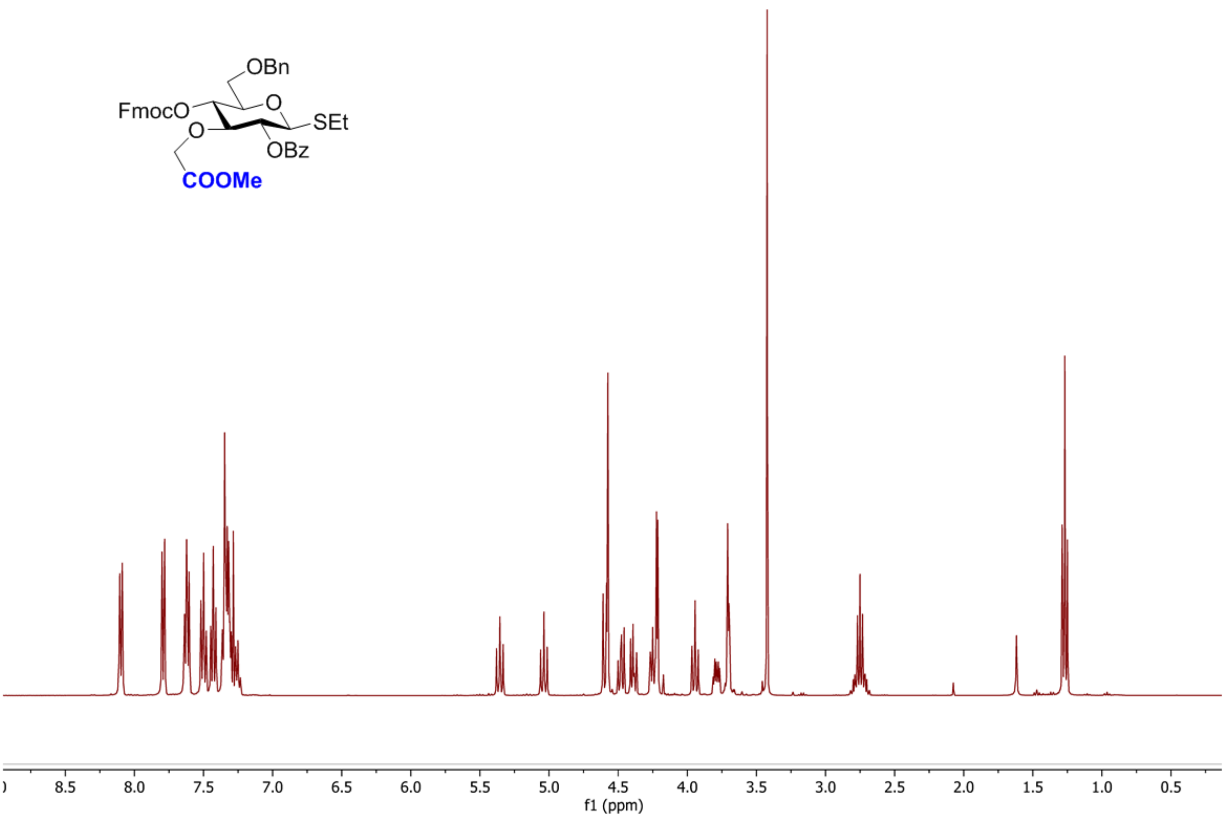

^{13}C NMR of 6 (101 MHz, $CDCl_3$)

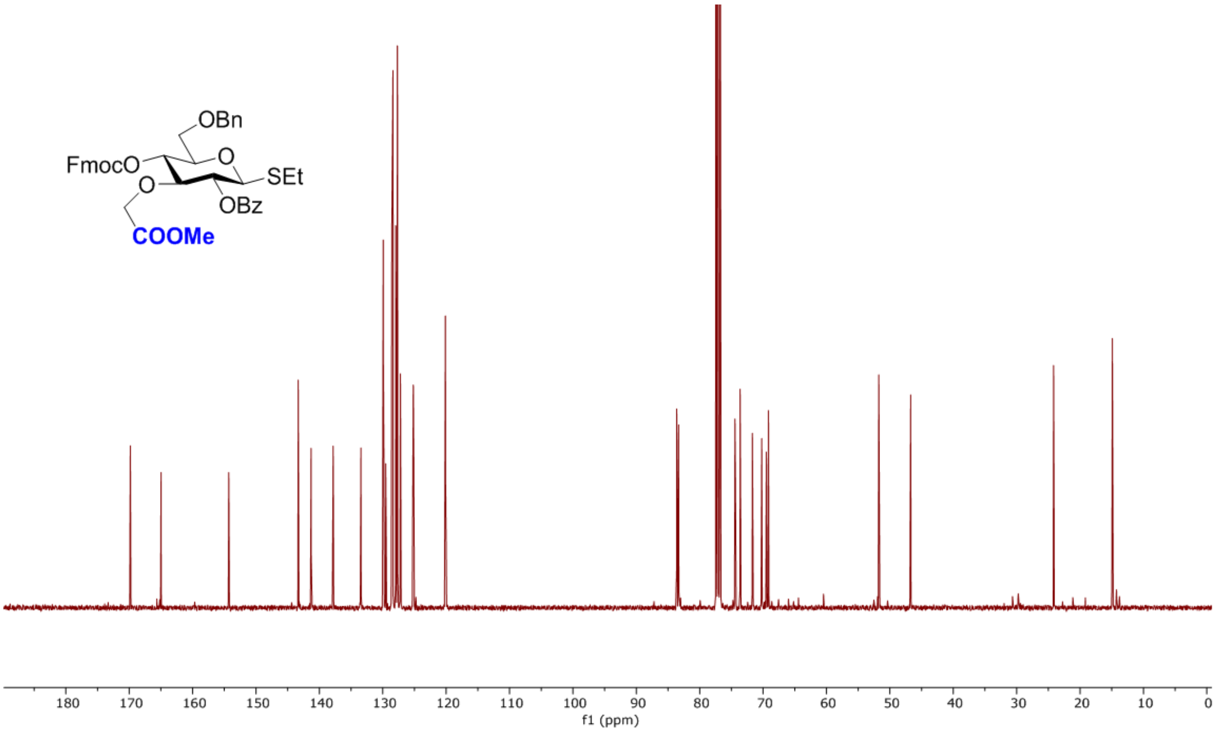

HSQC NMR of 6 ($CDCl_3$)

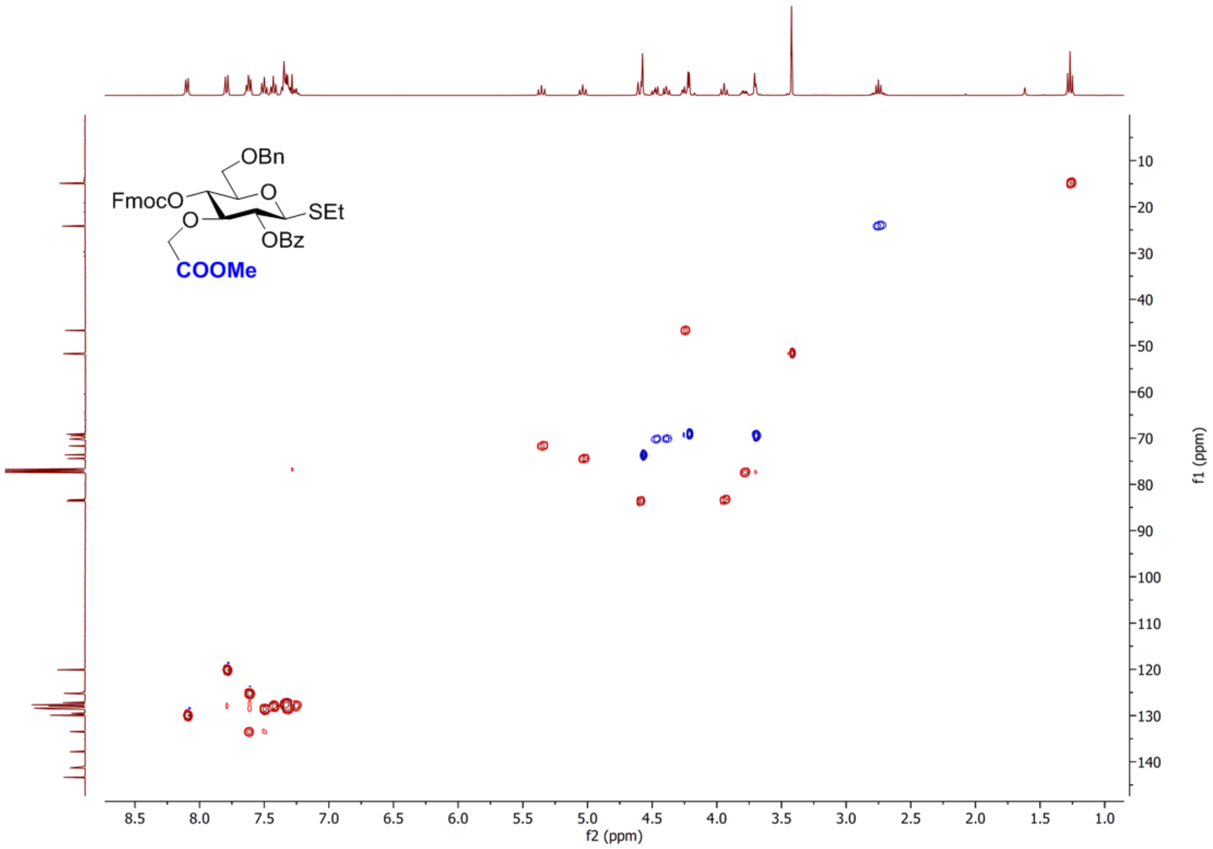

2.4. Synthesis of 7

Ethyl 2,4,6-tri-*O*-acetyl-3-deoxy-1-thio-β-D-glucopyranoside, 7-1

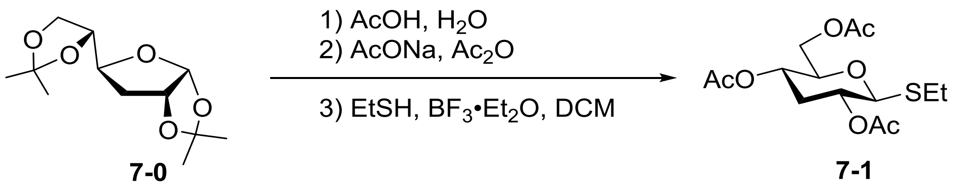

7-0 **7-1**

7-0 was prepared according to previously established procedures.[5]

3-Deoxy-1,2:5,6-di-*O*-isopropylidene-α-D-ribohexofuranose **7-0** (11.0 g, 45.0 mmol) was dissolved AcOH (90 ml) and water (10 mL) was added. The solution was heated to 105°C and stirred for 1 h. After the deprotection finished, the solvent was evaporated. A mixture of Ac_2O (100 mL) and NaOAc (1.84 g, 22.5 mmol) was heated to 110°C and the crude product added to the mixture. After 1 h, the solvent was evaporated and the crude per-acetylated compound was purified by column chromatography (Hexane : EtOAc = 4:1→2:1). The pure peracetylated compound was dissolved in anhydrous DCM (180 mL) and EtSH (4.86 mL, 67.5 mmol) was added under nitrogen atmosphere. The solution was cooled to 0°C and boron trifluoride etherate (8.33 mL, 67.5 mmol) was added dropwise. The reaction was allowed to room temperature and stirred overnight. The reaction mixture was wash

with saturated $NaHCO_3$ solution (aq) (100 mL, twice) and water (50 mL). The water layers were combined and extracted with DCM (50 mL). The organic layers were combined and dried with Na_2SO_4 and evaporated. The resulting crude product was purified by column chromatography (Hexane : EtOAc = 5:1→3:1) to give **7-1** (with 10 % α anomer) as a white solid (1.70 g, 11 % over three steps). The yield was lowered by the formation of furanose by-products during acetylation, which was also reported in the previous literature.[6] ^{1}H NMR (400 MHz, $CDCl_3$) δ 4.84 (dddd, *J* = 16.5, 11.2, 9.8, 4.9 Hz, 2H), 4.44 (d, *J* = 9.8 Hz, 1H), 4.19 (d, *J* = 4.0 Hz, 2H), 3.64 (dt, *J* = 9.8, 4.0 Hz, 1H), 2.77 – 2.56 (m, 3H), 2.067 (s, 3H), 2.065 (s, 3H), 2.04 (s, 3H), 1.62 (q, *J* = 11.4 Hz, 1H), 1.28 (t, *J* = 7.4 Hz, 3H); ^{13}C NMR (101 MHz, $CDCl_3$) δ 170.83, 169.49, 169.43, 85.21, 77.88, 67.33, 65.94, 62.80, 35.26, 24.24, 20.99, 20.91, 20.82, 14.99; $[\alpha]_D^{25}$ -17.03 (c = 1, $CHCl_3$); IR (neat) ν_{max} = 1744, 1227, 1035 cm^{-1}; m/z (HRMS$^+$) 357.0981 [M + Na]$^+$ ($C_{14}H_{22}O_7SNa^+$ requires 357.0978).

^{1}H NMR of 7-1 (400 MHz, $CDCl_3$)

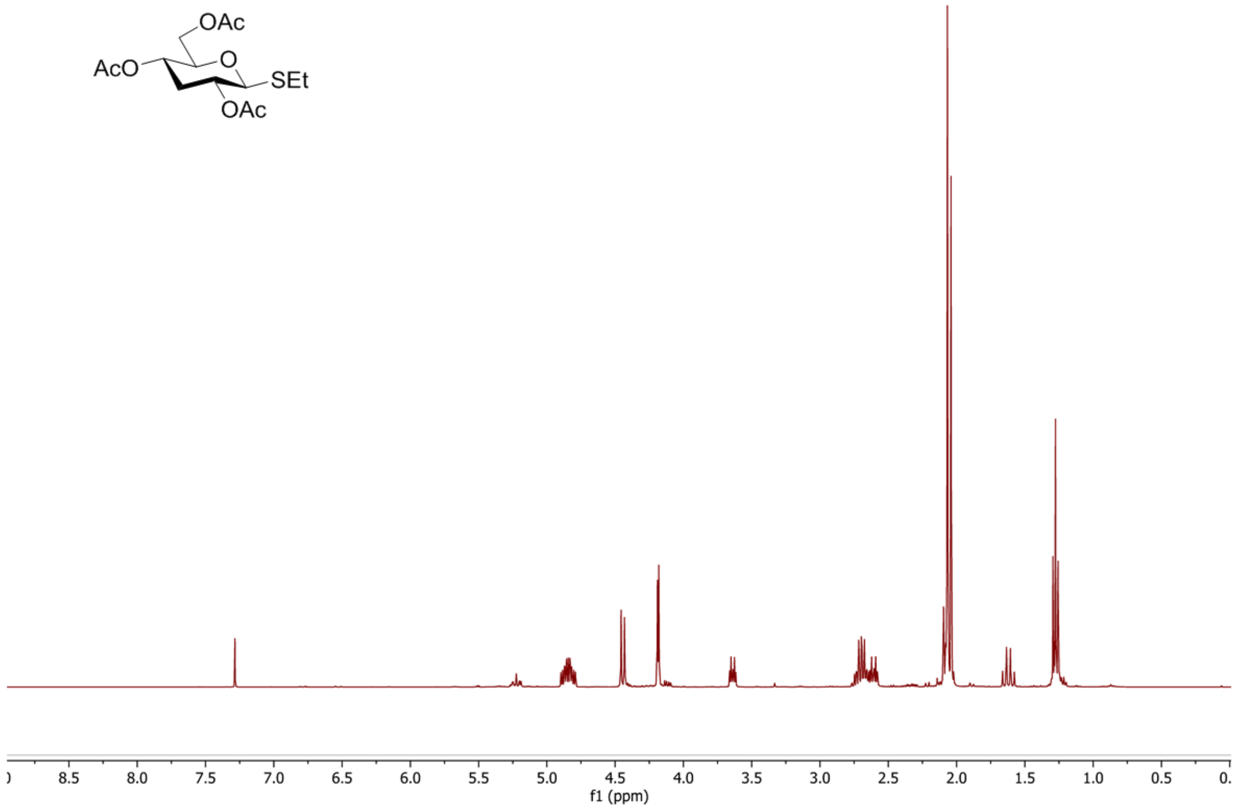

^{13}C NMR of 7-1 (101 MHz, $CDCl_3$)

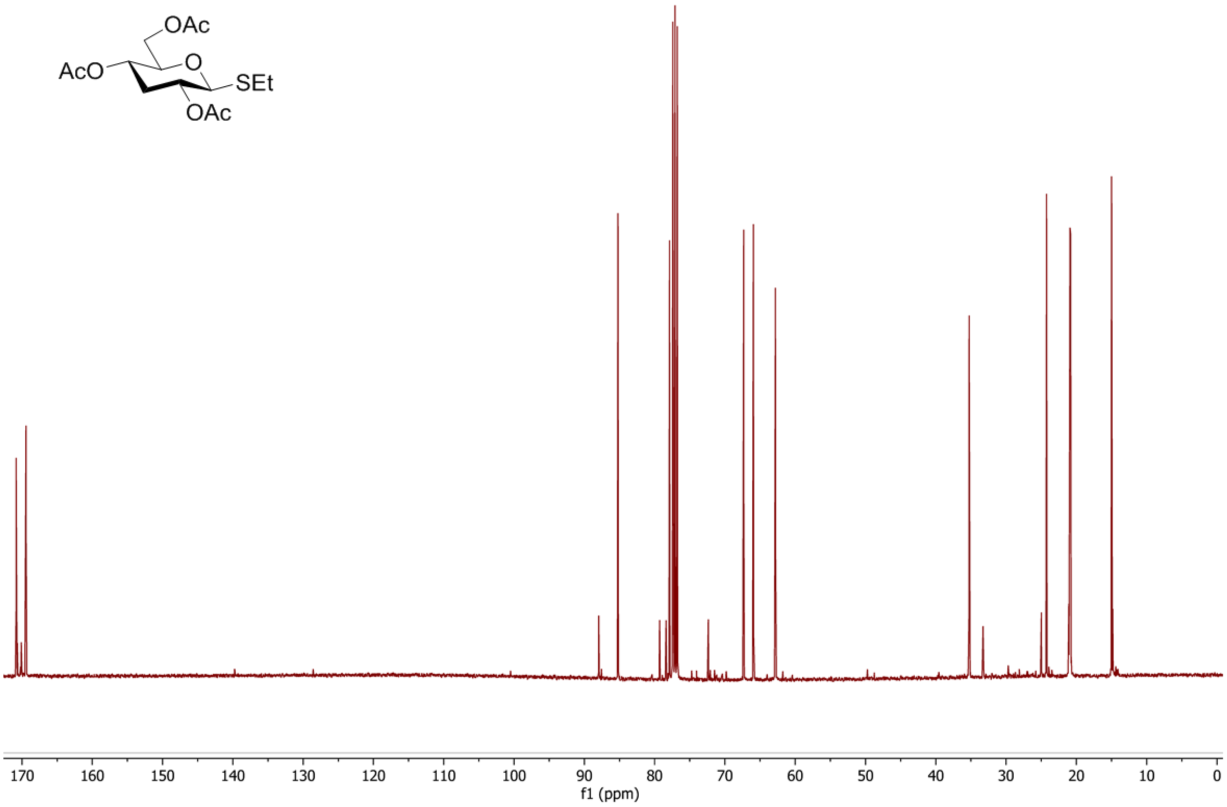

Ethyl 3-deoxy-4,6-*O*-phenylmethylene-1-thio-β-D-glucopyranoside, 7-2

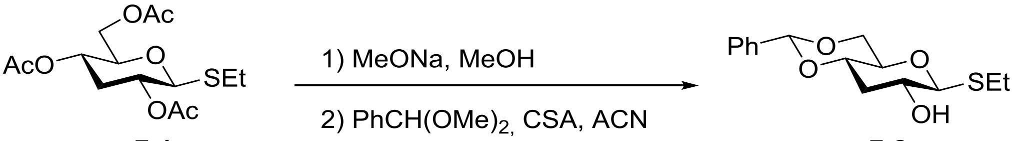

7-1 **7-2**

Ethyl 2,4,6-tri-*O*-acetyl-3-deoxy-1-thio-β-D-glucopyranoside **7-1** (1.70 g, 5.1 mmol) was dissolved in MeOH (20 ml), MeONa (2 mL, 0.5 M in MeOH) was added. The reaction was stirred at room temperature overnight. Amberlite IR-120 (H^+ form) was added to neutralize the reaction. After filtered the resin, the solvent was evaporated and the crude compound dissolved in acetonitrile (20 mL). Camphorsulfonic acid (0.24 g, 1.0 mmol) and benzaldehyde dimethyl acetal (1.6 mL, 10.4 mmol) were added, and the reaction was stirred at room temperature overnight. Upon completion, TEA (1 mL) was added to quench the reaction. The solvent was evaporated and the resulting crude product was purified by column chromatography (Hexane : EtOAc = 4:1→2:1) to give **7-2** as a white solid (0.92 g, 61 % over two steps). ^{1}H NMR (400 MHz, $CDCl_3$) δ 7.54 – 7.49 (m, 2H), 7.43 – 7.36 (m, 3H), 5.55 (s, 1H), 4.44 – 4.27 (m, 2H), 3.76 (t, *J* = 10.3 Hz, 1H), 3.60 (dtd, *J* = 11.0, 9.4, 4.5 Hz, 2H), 3.46 (ddd, *J* = 10.2, 9.0, 4.9 Hz, 1H), 2.77 (qd, *J* = 7.5, 2.0 Hz, 2H), 2.67 – 2.65 (m, 1H), 2.58 (dt, *J* = 11.8, 4.5 Hz, 1H), 1.76 (q, *J* = 11.6 Hz, 1H), 1.35 (t, *J* = 7.4 Hz, 3H); ^{13}C NMR (101 MHz, $CDCl_3$) δ 137.25, 129.22, 128.41, 126.24, 101.73, 89.30, 76.06, 73.84, 69.02, 67.93, 36.83, 24.91, 15.54); $[\alpha]_D^{25}$ -66.80 (c = 1, $CHCl_3$); IR (neat) ν_{max} = 1068, 1029, 998 cm^{-1}; m/z (HRMS$^+$) 319.0983 $[M + Na]^+$ ($C_{15}H_{20}O_4SNa^+$ requires 319.0975).

^{1}H NMR of 7-2 (400 MHz, $CDCl_3$)

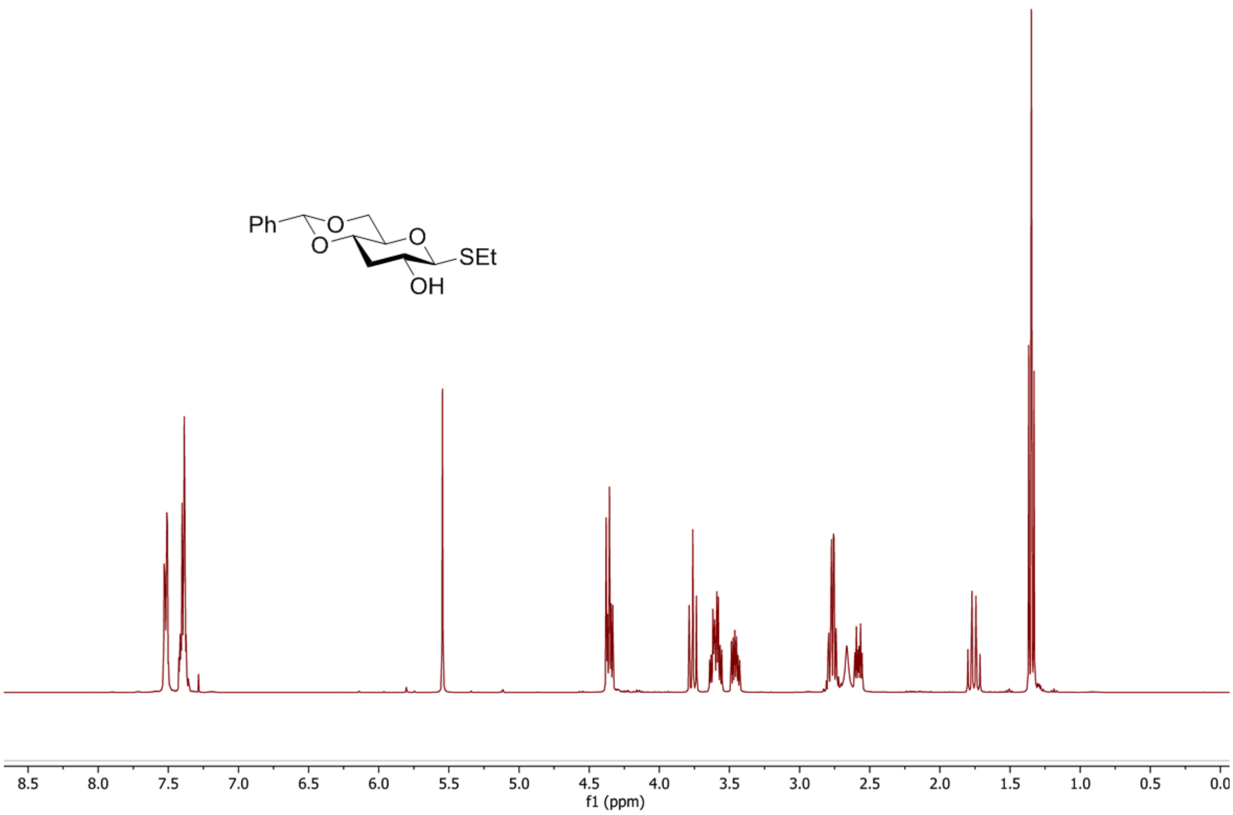

^{13}C NMR of 7-2 (101 MHz, $CDCl_3$)

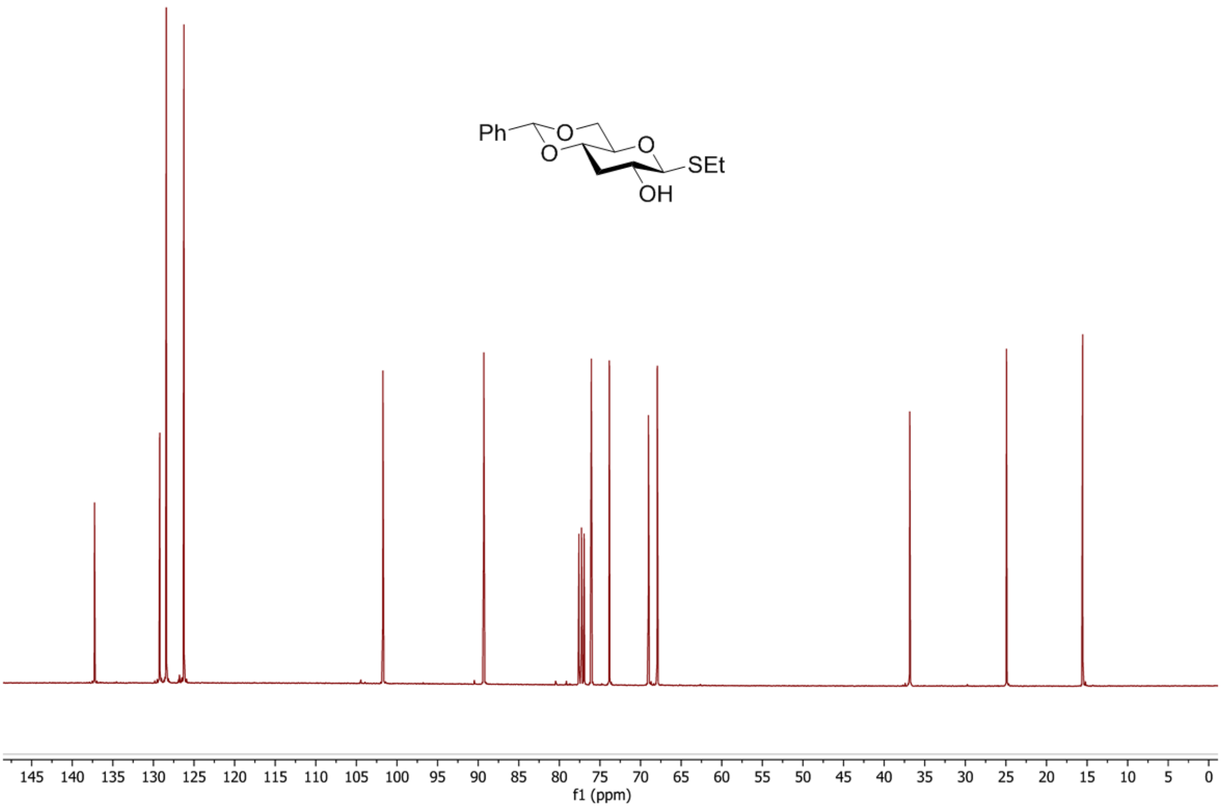

Ethyl 2-*O*-benzoyl-3-deoxy-4,6-*O*-phenylmethylene-1-thio-β-D-glucopyranoside, 7-3

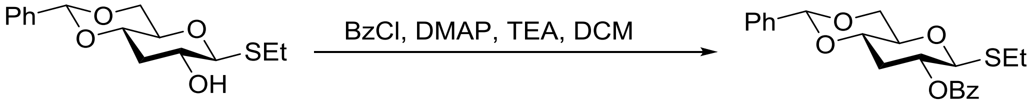

7-2 **7-3**

Ethyl 3-deoxy-4,6-*O*-phenylmethylene-1-thio-β-D-glucopyranoside **7-2** (0.92 g, 3.1 mmol) was dissolved in anhydrous DCM (10 mL). TEA (1.3 mL, 17.8 mmol) and 4-dimethylaminopyridine (75 mg, 0.6 mmol) were added. Benzoyl chloride (0.52 mL, 4.5 mmol) was added dropwise to the solution cooled with an ice bath. After 30 min, the system was allowed to room temperature and stirred for additional 10 h. The reaction mixture was washed with water (10 mL), dried with Na_2SO_4 and evaporated. The resulting crude product was purified by column chromatography (Hexane : EtOAc = 5:1→3:1) to give **7-3** as a white solid (1.05 g, 84 %). ^{1}H NMR (400 MHz, $CDCl_3$) δ 8.00 – 7.95 (m, 2H), 7.54 – 7.46 (m, 1H), 7.44 – 7.33 (m, 4H), 7.32 – 7.25 (m, 3H), 5.49 (s, 1H), 5.10 (ddd, *J* = 11.0, 9.9, 5.0 Hz, 1H), 4.61 (d, *J* = 9.9 Hz, 1H), 4.30 (dd, *J* = 10.6, 4.9 Hz, 1H), 3.72 (t, *J* = 10.3 Hz, 1H), 3.65 (ddd, *J* = 11.7, 9.0, 4.2 Hz, 1H), 3.46 (ddd, *J* = 10.1, 9.0, 4.9 Hz, 1H), 2.74 – 2.53 (m, 3H), 1.86 (q, *J* = 11.5 Hz, 1H), 1.19 (t, *J* = 7.4 Hz, 3H); ^{13}C NMR (101 MHz, $CDCl_3$) δ 165.24, 137.17, 133.35, 129.87, 129.76, 129.23, 128.50, 128.42, 126.18, 101.72, 85.49, 75.65, 73.96, 69.03, 68.76, 35.53, 24.25, 15.10; $[\alpha]_D^{25}$ -53.11 (c = 1, $CHCl_3$); IR (neat) ν_{max} = 1723, 1270, 1097 cm^{-1}; m/z (HRMS$^+$) 439.1187 $[M + K]^+$ ($C_{22}H_{24}O_5SK^+$ requires 439.0976).

^{1}H NMR of 7-3 (400 MHz, $CDCl_3$)

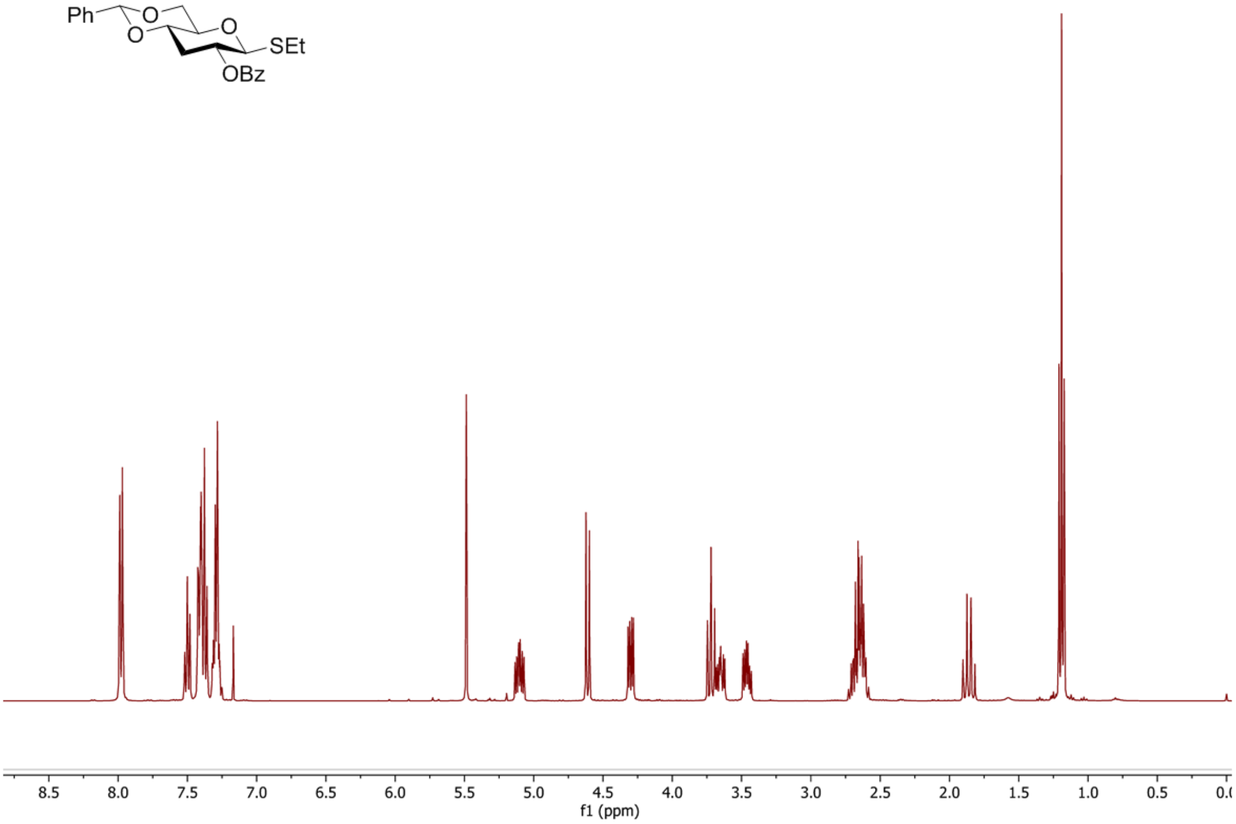

^{13}C NMR of 7-3 (101 MHz, $CDCl_3$)

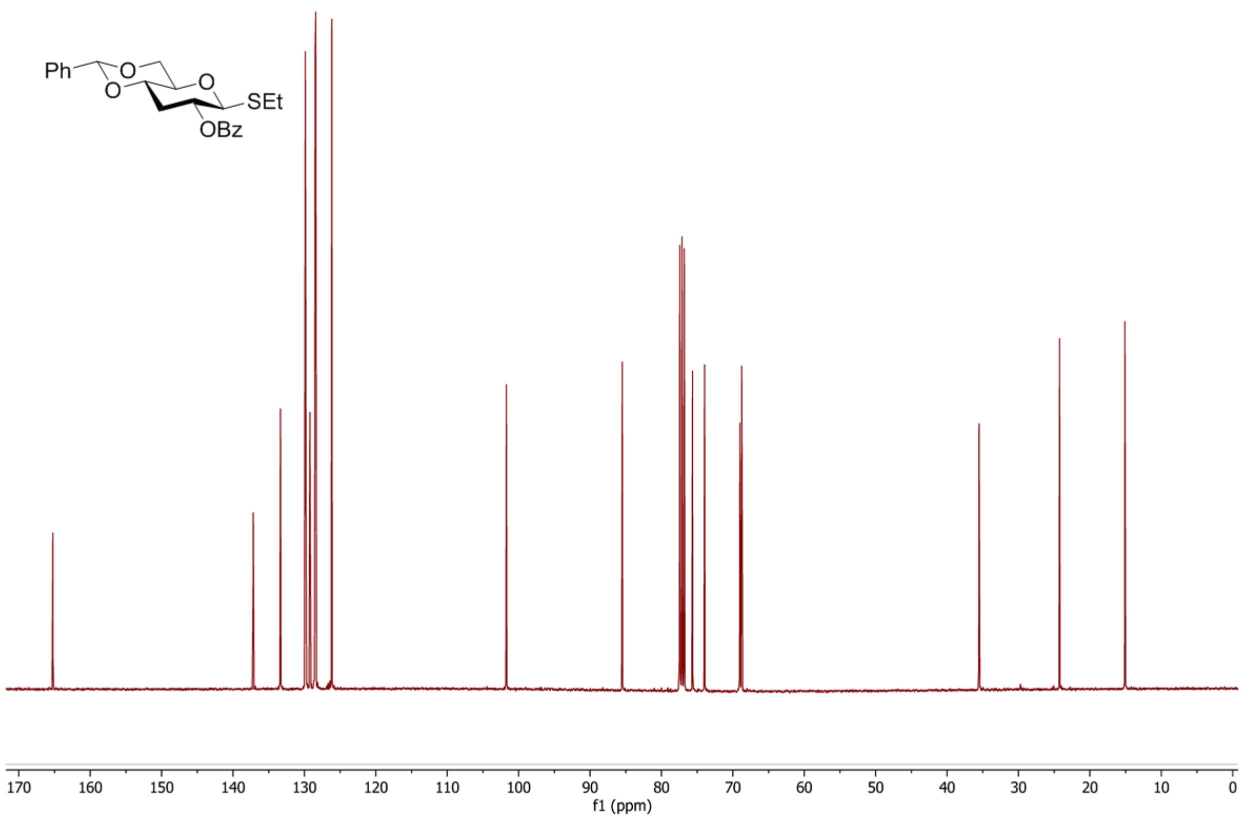

Ethyl 2-*O*-benzoyl-3-*O*-deoxy-6-*O*-benzyl-1-thio-β-D-glucopyranoside, 7-4

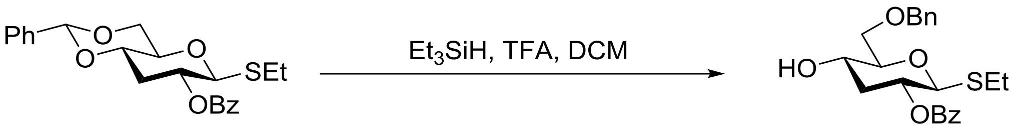

7-3 **7-4**

Ethyl 2-*O*-benzoyl-3-deoxy-4,6-*O*-phenylmethylene-1-thio-β-D-glucopyranoside **7-3** (1.05 g, 2.6 mmol) was dissolved in anhydrous DCM (10 mL). 4Å molecular sieves were added and the system was charged with nitrogen. Triethylsilane (4.2 mL, 26.0 mmol) was added and the mixture was stirred at room temperature for 30 min, after which time it was cooled to 0°C. TFA (2.1 mL, 27.0 mmol) was added and the mixture was stirred for 1 h at 0°C. Upon completion, molecular sieves were filtered and the organic phase was washed with saturated $NaHCO_3$ solution (aq) (10 mL, twice) and water (10 mL). The water layers were combined and extracted with DCM (10 mL). The organic layers were combined and dried with Na_2SO_4 and evaporated. The resulting crude product was purified by column chromatography (Hexane : EtOAc = 4:1→3:1) to give **7-4** as a colorless oil (0.98 g, 93 %). ^{1}H NMR (400 MHz, $CDCl_3$) δ 8.11 – 8.03 (m, 2H), 7.63 – 7.56 (m, 1H), 7.47 (dd, *J* = 8.4, 7.1 Hz, 2H), 7.43 – 7.31 (m, 5H), 5.07 (ddd, *J* = 11.3, 9.7, 4.8 Hz, 1H), 4.69 – 4.55 (m, 3H), 3.86 (ddd, *J* = 11.2, 7.8, 4.8 Hz, 2H), 3.74 (dd, *J* = 9.7, 6.8 Hz, 1H), 3.54 (ddd, *J* = 9.1, 6.8, 4.8 Hz, 1H), 3.16 (s, 1H), 2.81 – 2.59 (m, 3H), 1.75 (q, *J* = 11.5 Hz, 1H), 1.27 (t, *J* = 7.4 Hz, 3H); ^{13}C NMR (101 MHz, $CDCl_3$) δ 165.37, 137.28, 133.23, 129.90, 129.82, 128.63, 128.44, 128.12, 127.93, 84.93, 79.50, 73.93, 71.66, 68.58, 68.41, 38.06,

24.06, 15.10; $[\alpha]_D^{25}$ -65.62 (c = 1, $CHCl_3$); IR (neat) ν_{max} = 1721, 1272, 1069 cm^{-1}; m/z ($HRMS^+$) 425.1401 $[M + Na]^+$ ($C_{22}H_{26}O_5SNa^+$ requires 425.1393).

^{1}H NMR of 7-4 (400 MHz, $CDCl_3$)

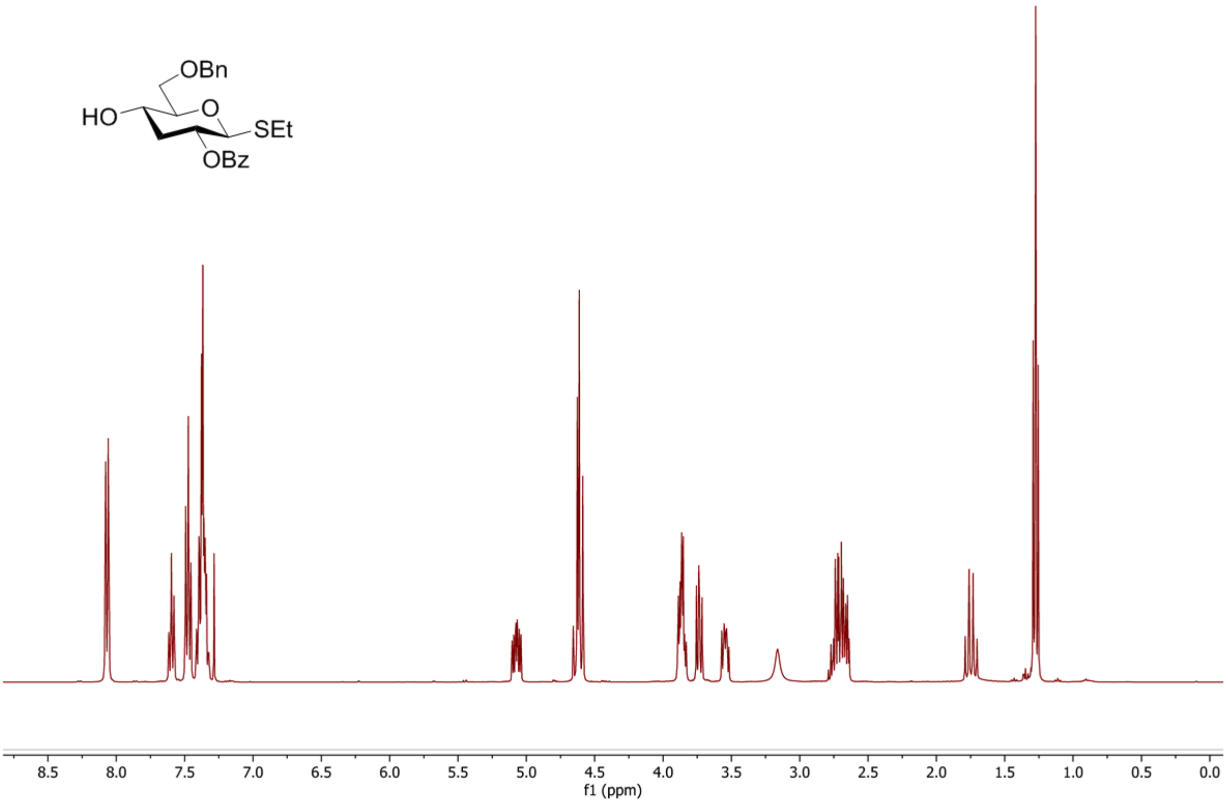

^{13}C NMR of 7-4 (101 MHz, $CDCl_3$)

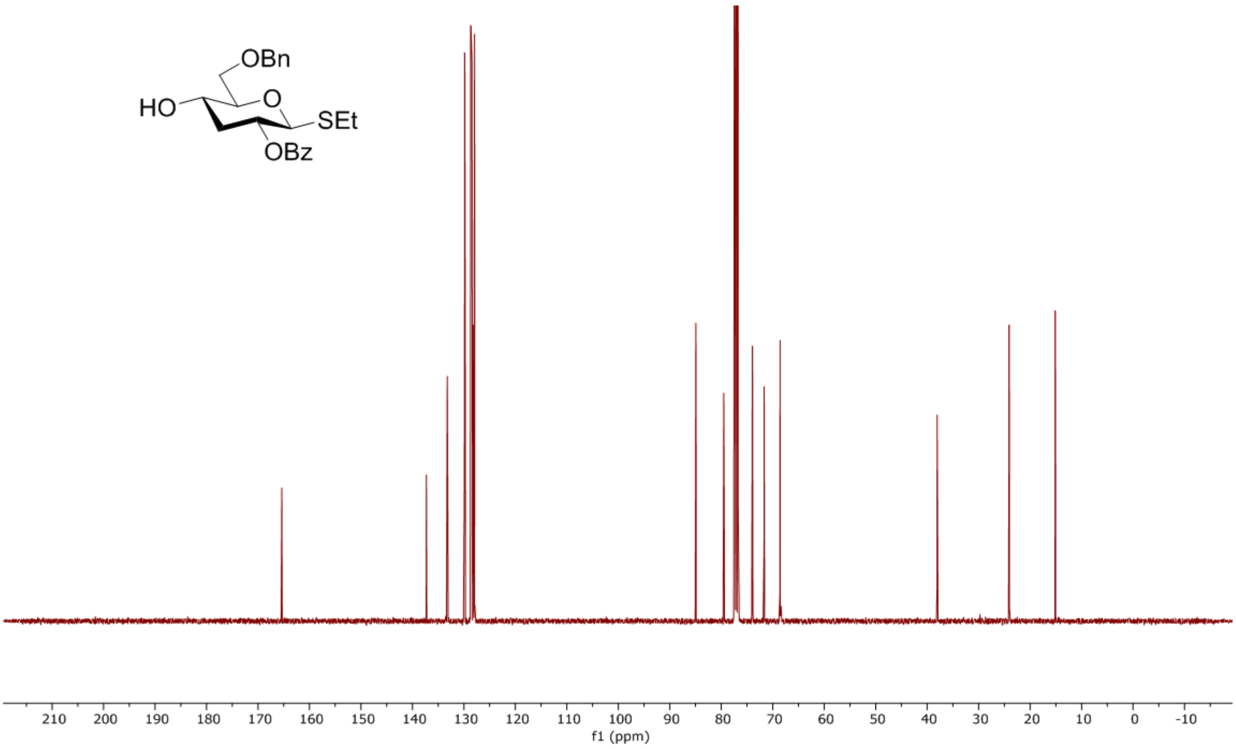

Ethyl 2-*O*-benzoyl-3-deoxy-4-*O*-(9-fluorenylmethoxycarbonyl)-6-*O*-benzyl-1-thio-β-D-glucopyranoside, 7

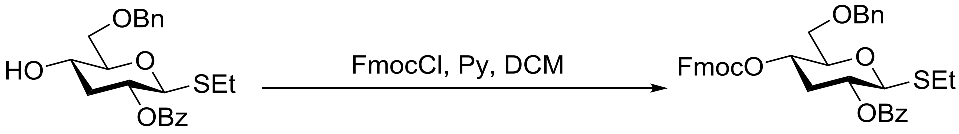

7-4 **7**

Ethyl 2-*O*-benzoyl-3-*O*-deoxy-6-*O*-benzyl-1-thio-β-D-glucopyranoside **7-4** (0.98 g, 2.4 mmol) was dissolved in anhydrous DCM (20 mL) and pyridine (1 mL, 12.4 mmol) was added. Then the solution was cooled with an ice bath for 15 min, after which time fluorenylmethyloxycarbonyl chloride (1.60 g, 6.2 mmol) was added slowly. The reaction was allowed to room temperature and stirred for 6 h. Upon completion, DCM (10 mL) was added and the organic phase was washed with aqueous citric acid (20 mL). After extracting the water phase with DCM (10 mL), the organic layers were combined and dried with Na_2SO_4. The solvent was evaporated and the resulting crude product was purified by column chromatography. (Hexane : EtOAc = 5:1→3:1) to give **7** as a white solid (1.20 g, 80 %). ^{1}H NMR (400 MHz, $CDCl_3$) δ 8.11 – 8.07 (m, 2H), 7.79 (d, *J* = 7.6 Hz, 2H), 7.63 – 7.57 (m, 3H), 7.49 (dd, *J* = 8.3, 7.1 Hz, 2H), 7.43 (t, *J* = 7.7 Hz, 2H), 7.39 – 7.31 (m, 6H), 7.30 – 7.26 (m, 1H), 5.16 (ddd, *J* = 11.0, 9.5, 4.9 Hz, 1H), 4.88 (ddd, *J* = 11.0, 9.3, 4.9 Hz, 1H), 4.69 (d, *J* = 9.6 Hz, 1H), 4.66 – 4.56 (m, 2H), 4.45 – 4.37 (m, 2H), 4.22 (t, *J* = 7.2 Hz, 1H), 3.82 – 3.68 (m, 3H), 2.86 – 2.70 (m, 3H), 1.90 (q, *J* = 11.2 Hz, 1H), 1.32 (t, *J* = 7.5 Hz, 3H); ^{13}C NMR (101 MHz, $CDCl_3$) δ 153.98, 143.25, 143.15, 141.33, 138.02, 133.31, 129.85, 129.77, 128.48, 128.40, 127.97, 127.69, 127.68, 127.22, 125.13, 120.14, 85.03, 79.47, 73.58, 70.40, 69.99, 69.33, 68.25, 46.72, 35.26, 24.22, 15.15; $[\alpha]_D^{25}$ 1.52 (c = 1, $CHCl_3$); IR (neat) ν_{max} = 1749, 1252, 1070 cm^{-1}; m/z ($HRMS^+$) 647.2080 $[M + Na]^+$ ($C_{37}H_{36}O_7SNa^+$ requires 647.2074).

^{1}H NMR of 7 (400 MHz, $CDCl_3$)

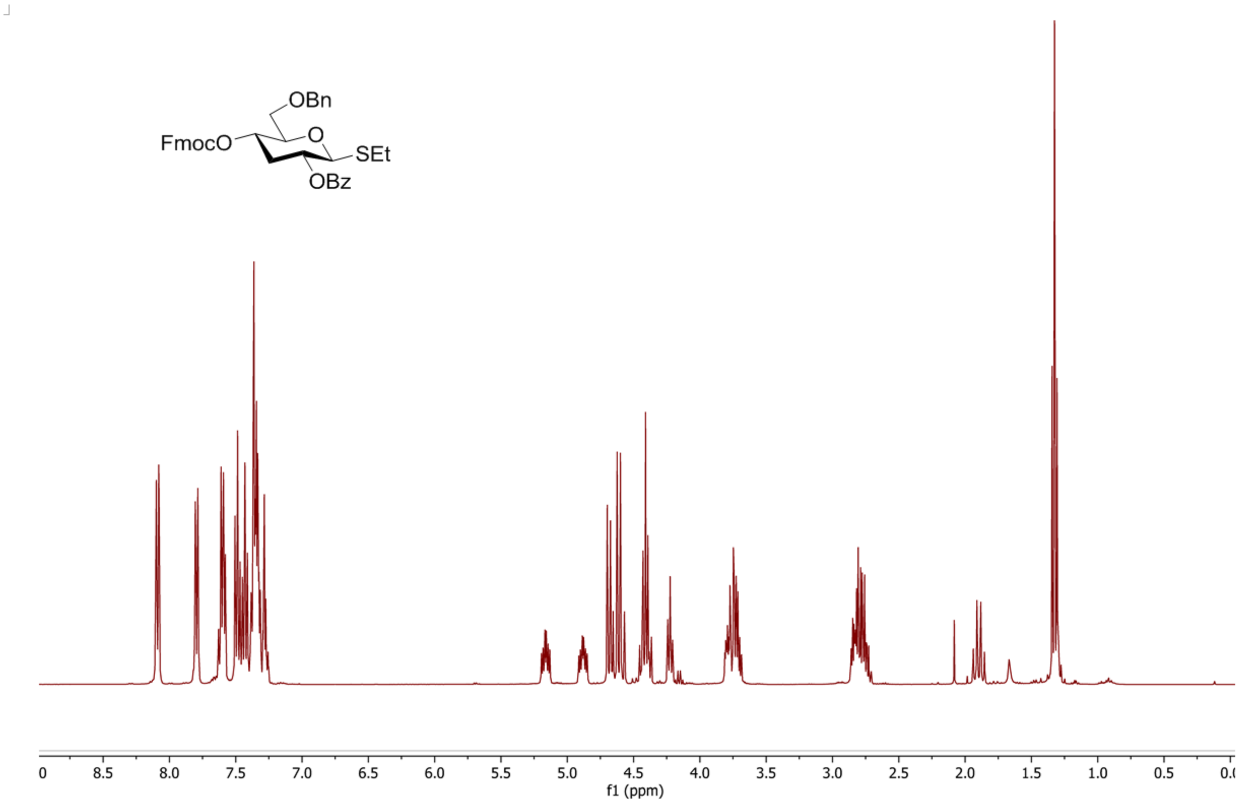

^{13}C NMR of 7 (101 MHz, $CDCl_3$)

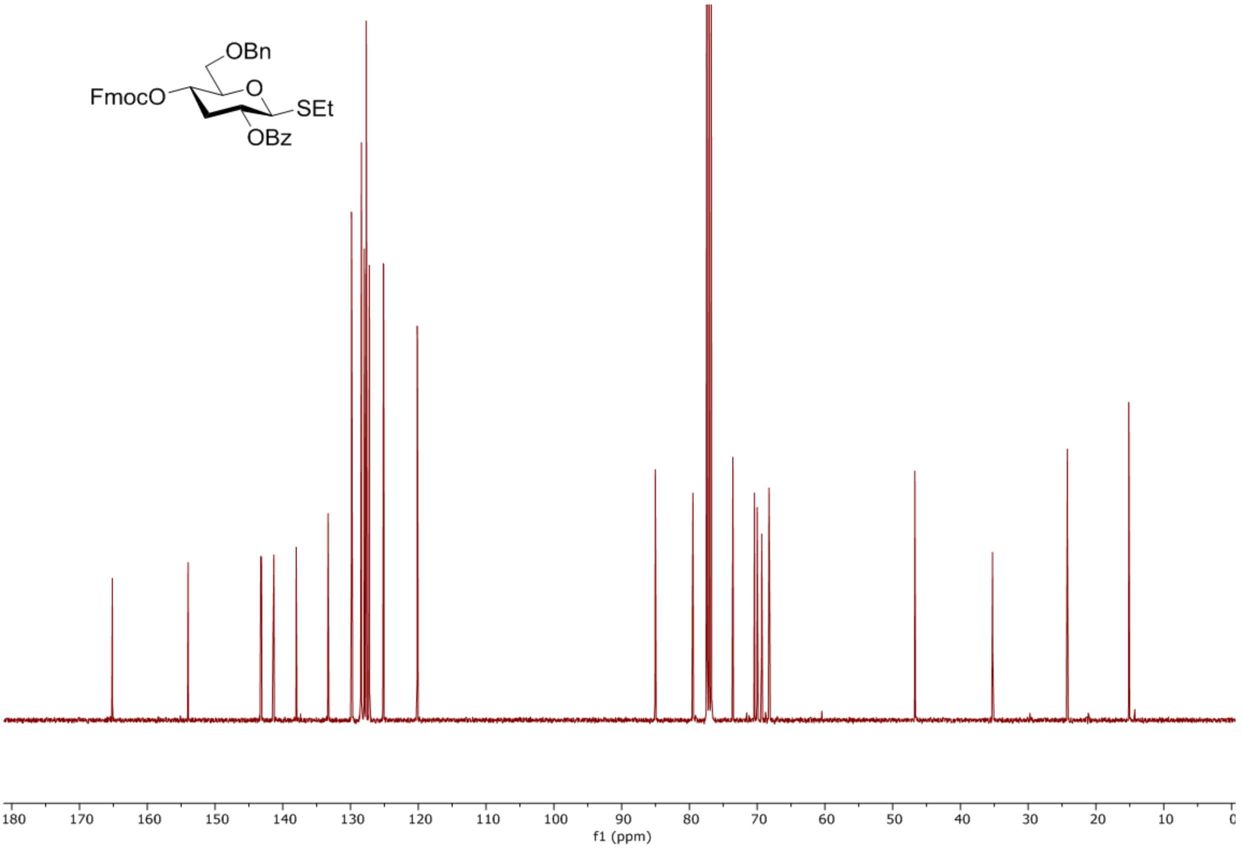

HSQC NMR of 7 ($CDCl_3$)

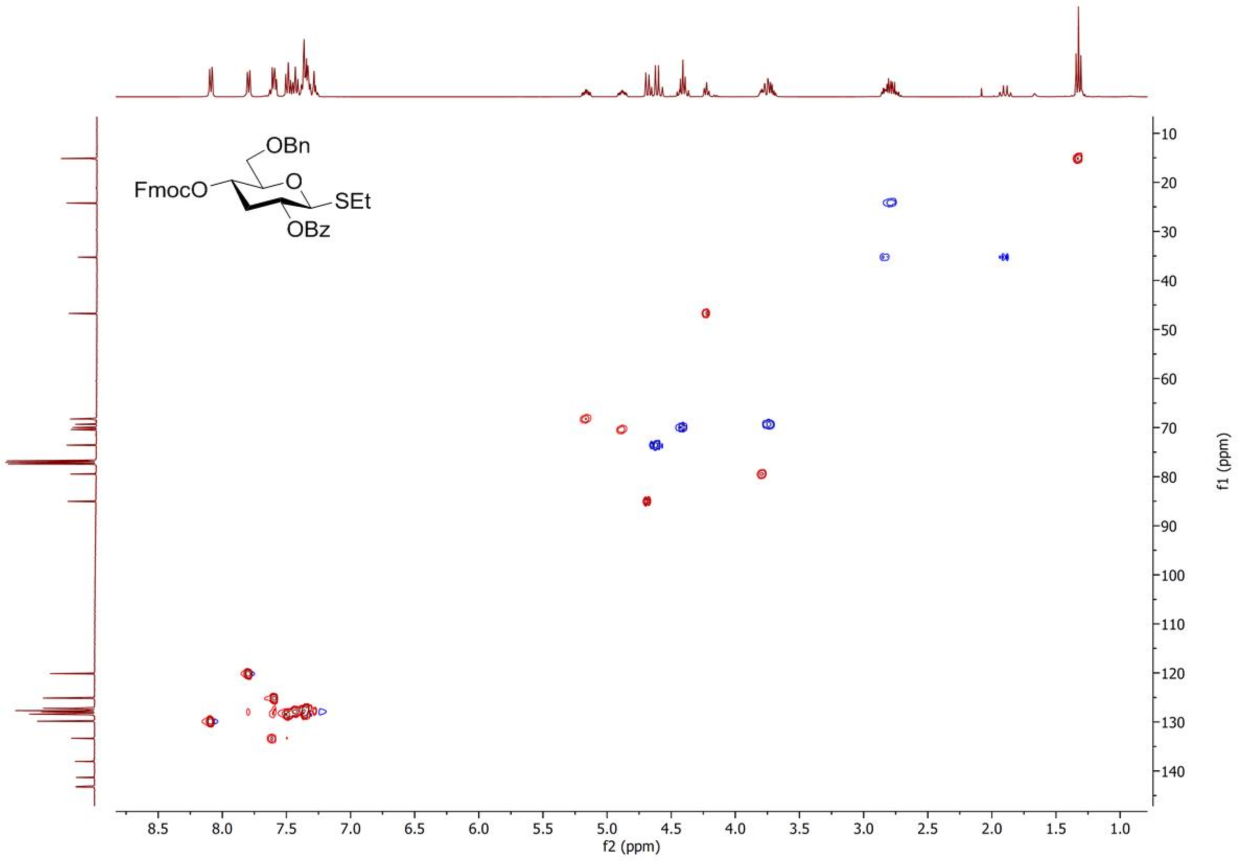

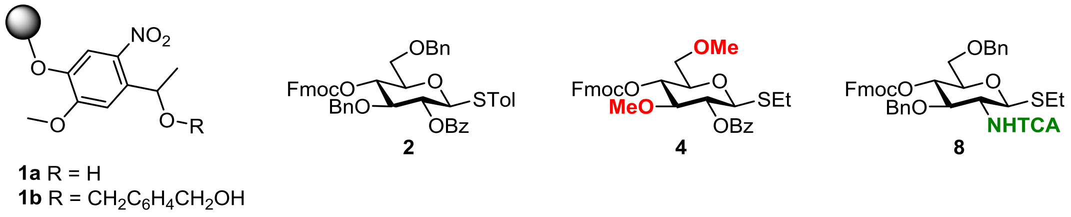

Building block **2** and **8** are commercially available. Photo-cleavable linkers and building block **4** were prepared according to previously established procedures.[2]

3. Automated Glycan Assembly

3.1. General materials and methods

All solvents used were HPLC-grade. The solvents used for the building block, activator, TMSOTf and capping solutions were taken from an anhydrous solvent system (jcmeyer-solvent systems). The building blocks were co-evaporated three times with toluene and dried for 1 h on high vacuum before use. Activator, capping, deprotection, acidic wash and building block solutions were freshly prepared and kept under argon during the automation run. All yields of products obtained by AGA

were calculated on the basis of resin loading. Resin loading was determined following previously established procedures.[1]

3.2. Preparation of stock solutions

- **Building Block**: between 0.06 and 0.08 mmol of building block (depending on BB) was dissolved in DCM (1 mL).
- **Activator solution**: 1.35 g of recrystallized NIS was dissolved in 40 mL of a 2:1 mixture of anhydrous DCM and anhydrous dioxane. Then triflic acid (55 µL) was added. The solution is kept at 0°C for the duration of the automation run.
- **Fmoc deprotection solution 1**: A solution of 20% piperidine in DMF (v/v) was prepared.
- **Fmoc deprotection solution 2**: A solution of 20% triethylamine in DMF (v/v) was prepared.
- **TMSOTf solution**: TMSOTf (0.45 mL) was added to DCM (40 mL).
- **Capping solution**: A solution of 10% acetic anhydride and 2% methanesulfunic acid in DCM (v/v) was prepared.

3.3. Modules for automated synthesis

Module A: Resin Preparation for Synthesis (20 min)

All automated syntheses were performed on 0.0125 mmol scale. Resin was placed in the reaction vessel and swollen in DCM for 20 min at room temperature prior to synthesis. During this time, all reagent lines needed for the synthesis were washed and primed. Before the first glycosylation, the resin was washed with the DMF, THF, and DCM (three times each with 2 mL for 25 s).

Module B: Acidic Wash with TMSOTf Solution (20 min)

The resin was swollen in 2 mL DCM and the temperature of the reaction vessel was adjusted to -20 °C. Upon reaching the low temperature, TMSOTf solution (1 mL) was added drop wise to the reaction vessel. After bubbling for 3 min, the acidic solution was drained and the resin was washed with 2 mL DCM for 25 s.

Action	Cycles	Solution	Amount	T (°C)	Incubation time
Cooling	-	-	-	-20	(15 min)*
Deliver	1	DCM	2 mL	-20	-
Deliver	1	TMSOTf solution	1 mL	-20	3 min
Wash	1	DCM	2 mL	-20	25 sec

*Time required to reach the desired temperature.

Module C: Thioglycoside Glycosylation (35 min)

The building block solution (0.08 mmol of BB in 1 mL of DCM per glycosylation) was delivered to the reaction vessel. After the set temperature was reached, the reaction was started by drop wise addition of the activator solution (1.0 mL, excess). The glycosylation conditions are building block

dependent (we report the most common set of conditions). After completion of the reaction, the solution is drained and the resin was washed with DCM, DCM:dioxane (1:2, 3 mL for 20 s) and DCM (two times, each with 2 mL for 25 s). The temperature of the reaction vessel is increased to 25 °C for the next module.

Action	Cycles	Solution	Amount	T (°C)	Incubation time
Cooling	-	-	-	-20	-
Deliver	1	BB solution	1 mL	-20	-
Deliver	1	Activator solution	1 mL	-20	-
Reaction time (BB dependent)	1			-20 to 0	5 min 20 min
Wash	1	DCM	2 mL	0	5 sec
Wash	1	DCM : Dioxane (1:2)	2 mL	0	20 sec
Heating	-	-	-	25	-
Wash	2	DCM	2 mL	> 0	25 sec

Module D: Capping (30 min)

The resin was washed with DMF (two times with 2 mL for 25 s) and the temperature of the reaction vessel was adjusted to 25 °C. 2 mL of Pyridine solution (10% in DMF) was delivered into the reaction vessel. After 1 min, the reaction solution was drained and the resin washed with DCM (three times with 3 mL for 25 s). 4 mL of capping solution was delivered into the reaction vessel. After 20 min, the reaction solution was drained and the resin washed with DCM (three times with 3 mL for 25 s).

Action	Cycles	Solution	Amount	T (°C)	Incubation time
Heating	-	-	-	25	(5 min)*
Wash	2	DMF	2 mL	25	25 sec
Deliver	1	10% Pyridine in DMF	2 mL	25	1 min
Wash	3	DCM	2 mL	25	25 sec
Deliver	1	Capping Solution	4 mL	25	20 min
Wash	3	DCM	2 mL	25	25 sec

*Time required to reach the desired temperature.

Module E: Fmoc Deprotection (9 min)

The resin was washed with DMF (three times with 2 mL for 25 s) and the temperature of the reaction vessel was adjusted to 25 °C. 2 mL of Fmoc deprotection solution 1 was delivered to the reaction vessel. After 5 min, the reaction solution was drained and the resin washed with DMF (three times with 3 mL for 25 s) and DCM (five times each with 2 mL for 25 s). The temperature of the reaction vessel is decreased to -20 °C for the next module.

Action	Cycles	Solution	Amount	T (°C)	Incubation time
Wash	3	DMF	2 mL	25	25 sec
Deliver	1	Fmoc depr. Solution 1	2 mL	25	5 min
Wash	1	DMF	2 mL		
Cooling	-	-	-	-20	-
Wash	3	DMF	2 mL	< 25	25 sec
Wash	5	DCM	2 mL	< 25	25 sec

Module E*: Fmoc Deprotection with TEA (20 min)

The resin was washed with DMF (three times with 2 mL for 25 s) and the temperature of the reaction vessel was adjusted to 25 °C. 2 mL of Fmoc deprotection solution 2 was delivered to the reaction vessel. After 5 min, the reaction solution was drained. (The deprotection process was repeated for 3 times). The resin was washed with DMF (three times with 3 mL for 25 s) and DCM (five times each with 2 mL for 25 s). The temperature of the reaction vessel is decreased to -20 °C for the next module.

Action	Cycles	Solution	Amount	T (°C)	Incubation time
**Wash**	3	DMF	2 mL	25	25 sec
**Deliver**	3	Fmoc depr. Solution 2	2 mL	25	5 min
**Wash**	1	DMF	2 mL		
**Cooling**	-	-	-	-20	-
**Wash**	3	DMF	2 mL	< 25	25 sec
**Wash**	5	DCM	2 mL	< 25	25 sec

3.4. Post-synthesizer manipulations

Module F: On-resin Methanolysis

The resin was suspended THF (5 mL). 0.5 mL of NaOMe in MeOH (0.5 M) was added and the suspension was gently shaken at room temperature. After micro-cleavage (see **Module G1**) indicated the complete removal of benzoyl groups (generally around 4 hours), the resin was repeatedly washed with MeOH (2mL x 3) and DCM (2mL x 3).

Module G: Cleavage from Solid Support

The oligosaccharides were cleaved from the solid support using a continuous-flow photoreactor as described previously.[7]

Module G1: Micro-cleavage from Solid Support

Trace amount of resin (around 20 beads) was dispersed in DCM (0.1 mL) and irradiated with a UV lamp (6 watt, 356 nm) for 10 minutes. ACN (10 µL) was then added to the resin and the resulting solution analyzed by MALDI.

Module H: Solution-phase methanolysis

The protected oligosaccharide was dissolved in MeOH: DCM (1.5 mL, 1:1). NaOMe in MeOH (0.5 M, 3 equiv. per benzoyl ester) was added and the solution was stirred at room temperature for 12 h, neutralized with Amberlite IR-120 (H^+ form), filtered and concentrated *in vacuo.* The crude compound was used for hydrogenolysis without further purification.

Module H*: Solution-phase methanolysis for 3-*O*-methoxycarbonyl sugars

The protected oligosaccharide was dissolved in THF (1.5 mL). NaOMe in MeOH (0.5 M, 3 equiv. per benzoyl ester) was added and the solution was stirred at room temperature for 12 h, neutralized with HOAc and concentrated *in vacuo.* The crude compound was used for hydrogenolysis without further purification. These conditions also hydrolyze the methyl esters due to the trace amount of water in the reaction mixture.

Module I: Hydrogenolysis

The crude compound obtained from *Module F* or *H* was dissolved in 2 mL of EA:*t*BuOH:H_2O (1:0.5:0.5). 100% by weight Pd-C (10%) was added and the reaction was stirred in H_2 bomb with 60 psi pressure. Generally, the hydrogenolysis is completed within 1 hour. If the reaction does not go to completion after 1 hour, the reaction should be monitored every 30 min and stopped right after completion to prevent the undesired reduction of the free reducing end. Upon completion, the reaction was filtered through celite and washed with DCM, *t*BuOH and H_2O. The filtrates were concentrated *in vacuo.*

Module I*: Hydrogenolysis at ambient pressure

The crude compound obtained from *Module H** was dissolved in 2 mL of EA:*t*BuOH:H_2O (1:0.5:0.5). 100% by weight Pd-C (10%) was added and the reaction was stirred under H_2–atmosphere for 6 h. The reaction was filtered through celite and washed with *t*BuOH and H_2O. The filtrates were concentrated *in vacuo.* For 3-*O*-methoxycarbonyl sugars, the pH value was adjusted to 2-3 by using formic acid before HPLC purification.

Module J: Purification

Purification was conducted at different stage of the synthesis as reported for the individual procedures. The crude products were analyzed using analytical HPLC (Agilent 1200 Series spectrometer, **Method A** and **Method C**). The purification was conducted using preparative HPLC (Agilent 1200 Series spectrometer) or C_{18} reverse phase silica gel column chromatography.

- **Method A:** (YMC-Diol-300 column, 150 x 4.6 mm) flow rate of 1.0 mL / min with Hex – 20% EtOAc as eluent [isocratic 20% EtOAc (5 min), linear gradient to 55% EtOAc (35 min), linear gradient to 100% EtOAc (5 min)].

- **Method B:** (YMC-Diol-300 column, 150 x 20 mm) flow rate of 15 mL / min with Hex – 20% EtOAc as eluents [isocratic 20% EtOAc (5 min), linear gradient to 55% EtOAc (35 min), linear gradient to 100%.
- **Method B1:** (YMC-Diol-300 column, 150 x 20 mm) flow rate of 15 mL/min with Heptane/EtOAc as eluent [isocratic 20% EtOAc (5 min), linear gradient to 100% EtOAc].
- **Method C:** (Synergi Hydro RP18 column, 250 x 4.6 mm) flow rate of 1.0 mL / min with H_2O (0.1% formic acid) as eluents [isocratic (5 min), linear gradient to 30% ACN (30 min), linear gradient to 100% ACN (5 min)].
- **Method D:** (Synergi Hydro RP18 column, 250 x 10 mm) flow rate of 4.0 mL / min with H_2O (0.1% formic acid) as eluents [isocratic (5 min), linear gradient to 30% ACN (30 min), linear gradient to 100% ACN (5 min)].
- **Method E:** (Hypercarb column, 150 x 10 mm) flow rate of 1.3 mL / min with H_2O (0.1% formic acid) as eluents [isocratic (5 min), linear gradient to 30% ACN (30 min), linear gradient to 100% ACN (5 min)].
- **Method M:** (Manual reverse phase C_{18} silica gel column chromatography): H_2O (10 mL), 5% MeOH (10 mL), 7.5% MeOH (10 mL), 10% MeOH (10 mL), 15% MeOH (10 mL), 20% MeOH (10 mL).
- **Method N:** (Manual normal phase silica gel column chromatography): Hexanes: EtOAc = 2:1 to Hexanes: EtOAc = 1:2

Following final purification, all deprotected products were lyophilized on a Christ Alpha 2-4 LD plus freeze dryer prior to characterization.

3.5. Oligosaccharides synthesis

3.5.1. Synthesis of hexamers

3.5.1.1. ***Synthesis of AAAAAA-OH***

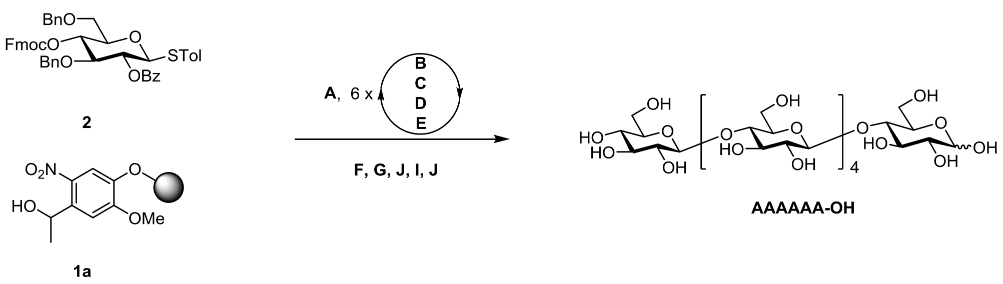

	Module	Conditions
	**A**: Resin Preparation for Synthesis	
	**B**: Acidic Wash with TMSOTf Solution	
6	**C**: Thioglycoside Glycosylation	**2**, 6.0 equiv.(-20°C for 5 min, 0°C for 20 min)
	**D**: Capping	
	**E**: Fmoc Deprotection	
	**F**: On-resin Methanolysis	
	**G**: Cleavage from Solid Support	
	**J**: Purification	**Method B1**, t_R = 31.9 and 32.7 min
	**I**: Hydrogenolysis	
	**J**: Purification	**Method D**, t_R = 14.1 min

Automated synthesis, global deprotection, and purification afforded **AAAAAA-OH** as white solid (2.4 mg, 18% overall yield).

Analytical data for **AAAAAA-OH**: ^{1}H NMR (600 MHz, D_2O) δ 5.24 (d, *J* = 3.7 Hz, 0.4H, α-H1), 4.68 (d, *J* = 7.9 Hz, 0.6H, β-H1), 4.58 – 4.50 (m, 5H), 4.05 – 3.90 (m, 6H), 3.90 – 3.80 (m, 6H), 3.79 – 3.57 (m, 16H), 3.55 – 3.48 (m, 2H), 3.46 – 3.41 (m, 1H), 3.41 – 3.27 (m, 5H); ^{13}C NMR (151 MHz, D_2O) δ 102.49, 102.27, 95.69 (β-C1), 91.75 (α-C1), 78.57, 78.42, 78.31, 78.19, 75.91, 75.41, 74.75, 74.18, 73.97, 73.93, 73.83, 73.08, 72.86, 71.23, 71.16, 70.05, 69.38, 60.50, 59.95, 59.82, 59.78; solubility not enough for optical rotation measurement; IR (neat) ν_{max} = 3378, 2928, 1656, 1054 cm^{-1}; (HRMS+) 1013.316 $[M + Na]^+$ ($C_{36}H_{62}NaO_{31}$ requires 1013.317).

RP-HPLC of AAAAAA-OH (ELSD trace, Method C, t_R = 14.1 min)

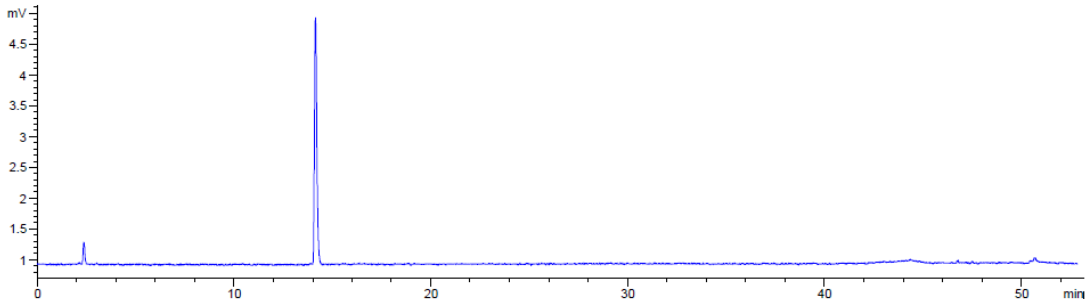

^{1}H NMR of AAAAAA-OH (600 MHz, D_2O)

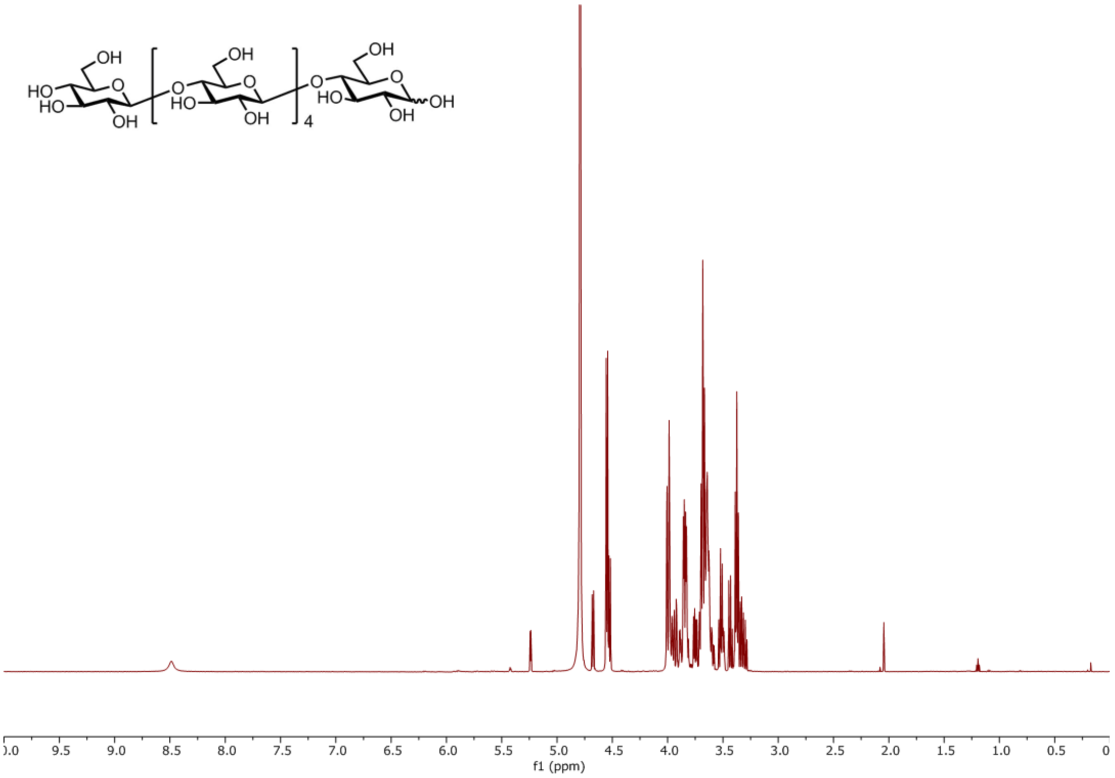

^{13}C NMR of AAAAAA-OH (151 MHz, D_2O)

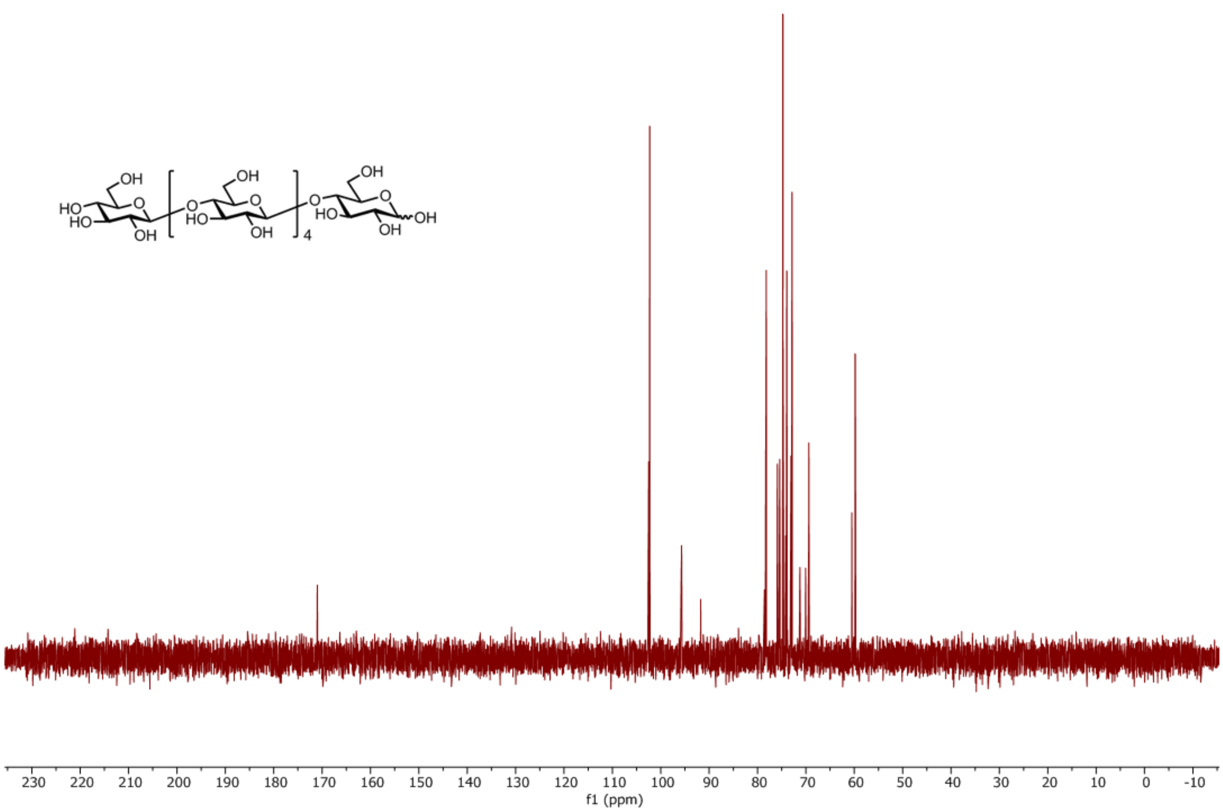

HSQC NMR of AAAAAA-OH (D_2O)

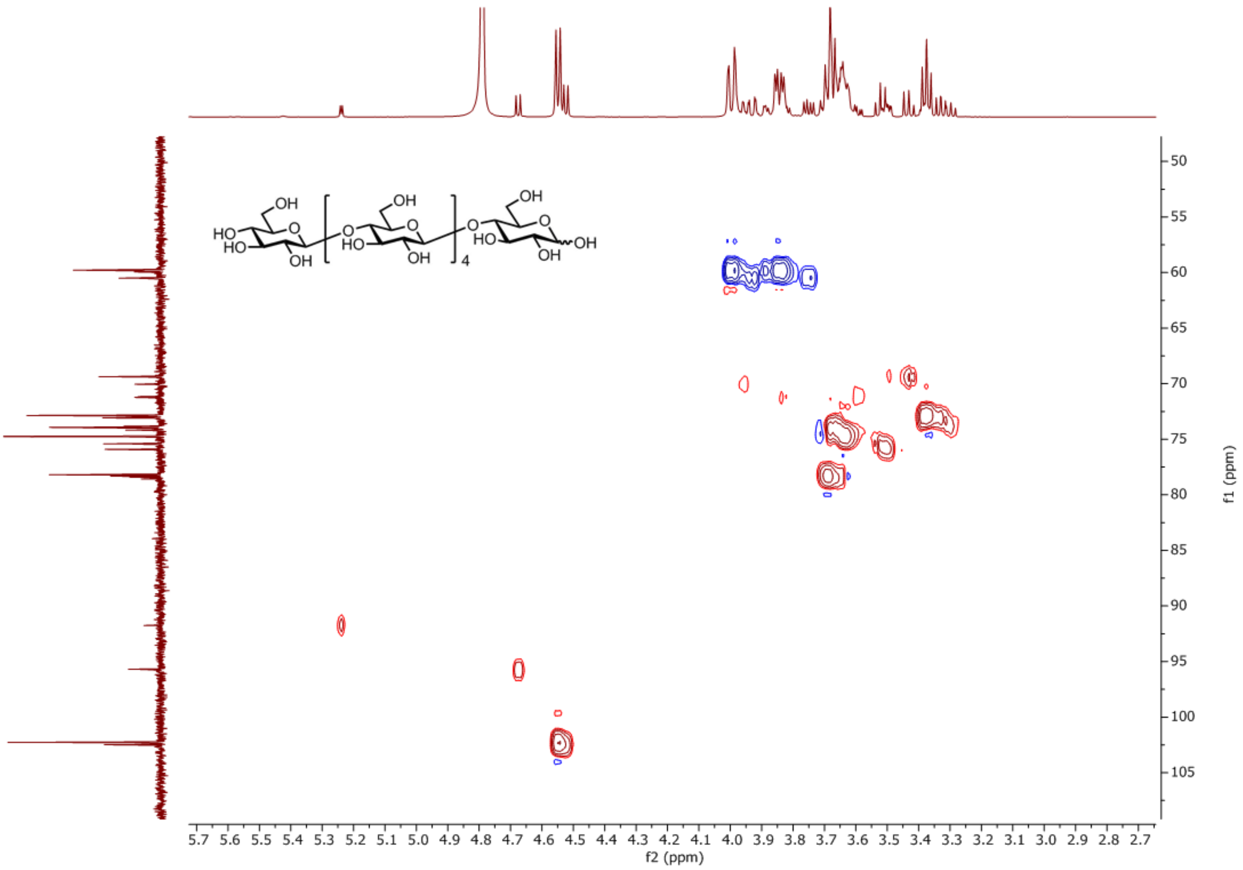

3.5.1.2. *Synthesis of AAABBB-OH*

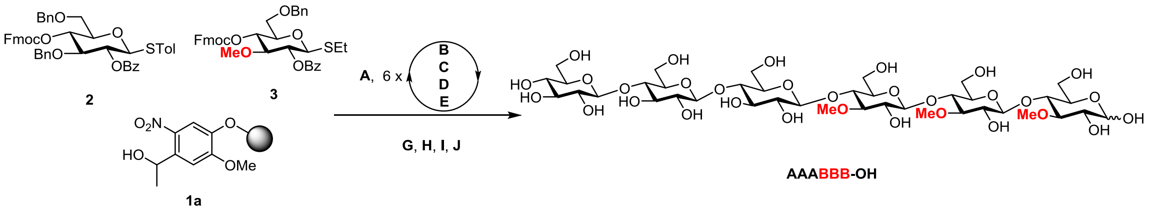

Module	Conditions
**A**: Resin Preparation for Synthesis	
3 × [**B**: Acidic Wash with TMSOTf Solution	
**C**: Thioglycoside Glycosylation	**3**, 6.5 equiv.(-20°C for 5 min, 0°C for 20 min)
**D**: Capping	
**E**: Fmoc Deprotection]	
3 × [**B**: Acidic Wash with TMSOTf Solution	
**C**: Thioglycoside Glycosylation	**2**, 6.5 equiv.(-20°C for 5 min, 0°C for 20 min)
**D**: Capping	
**E**: Fmoc Deprotection]	
**F**: On-resin Methanolysis	
**G**: Cleavage from Solid Support	
**I**: Hydrogenolysis	
**J**: Purification	**Method M**

Automated synthesis and global deprotection and purification afforded **AAABBB-OH** as white solid (6.3 mg, 49% overall yield).

Analytical data for **AAABBB-OH**: ^{1}H NMR (600 MHz, D_2O) δ 5.23 (d, *J* = 3.6 Hz, 0.42 H, α-H1), 4.67 (d, *J* = 7.8 Hz, 0.58 H, β-H1), 4.59 – 4.50 (m, 5H), 4.01 (ddt, *J* = 12.2, 10.1, 2.2 Hz, 4H), 3.98 – 3.91 (m, 2H), 3.90 – 3.84 (m, 4H), 3.84 – 3.78 (m, 4H), 3.75 (dd, *J* = 12.5, 5.9 Hz, 1H), 3.68 (td, *J* = 8.0, 7.4, 4.1 Hz, 3H), 3.65 (t, *J* = 4.8 Hz, 2H), 3.62 (s, 3H), 3.62 (s, 6H), 3.60 – 3.51 (m, 5H), 3.51 – 3.44 (m, 5H), 3.44 – 3.40 (m, 2H), 3.40 – 3.30 (m, 4H); ^{13}C NMR (151 MHz, D_2O) δ 102.48, 102.27, 102.22, 102.18, 102.17, 95.72 (β-C1), 91.75 (α-C1), 83.51, 83.44, 83.43, 83.39, 80.95, 78.34, 78.32, 75.91, 75.58, 75.49, 75.47, 75.44, 75.40, 74.99, 74.97, 74.96, 74.77, 74.74, 74.12, 73.98, 73.29, 73.18, 73.07, 72.87, 72.69, 72.68, 72.64, 70.69, 70.49, 69.37, 60.49, 59.94, 59.91, 59.87, 59.82, 59.50, 59.11, 59.08, 59.03, 59.00; $[\alpha]_D^{20}$ +14.62 (c 0.3, H_2O); IR (neat) ν_{max} = 3340, 2927, 1649, 1032 cm^{-1}; m/z (HRMS+) 1055.364 $[M + Na]^+$ ($C_{39}H_{68}NaO_{31}$ requires 1055.364).

RP-HPLC of AAABBB-OH (ELSD trace, Method C, t_R = 17.6 min)

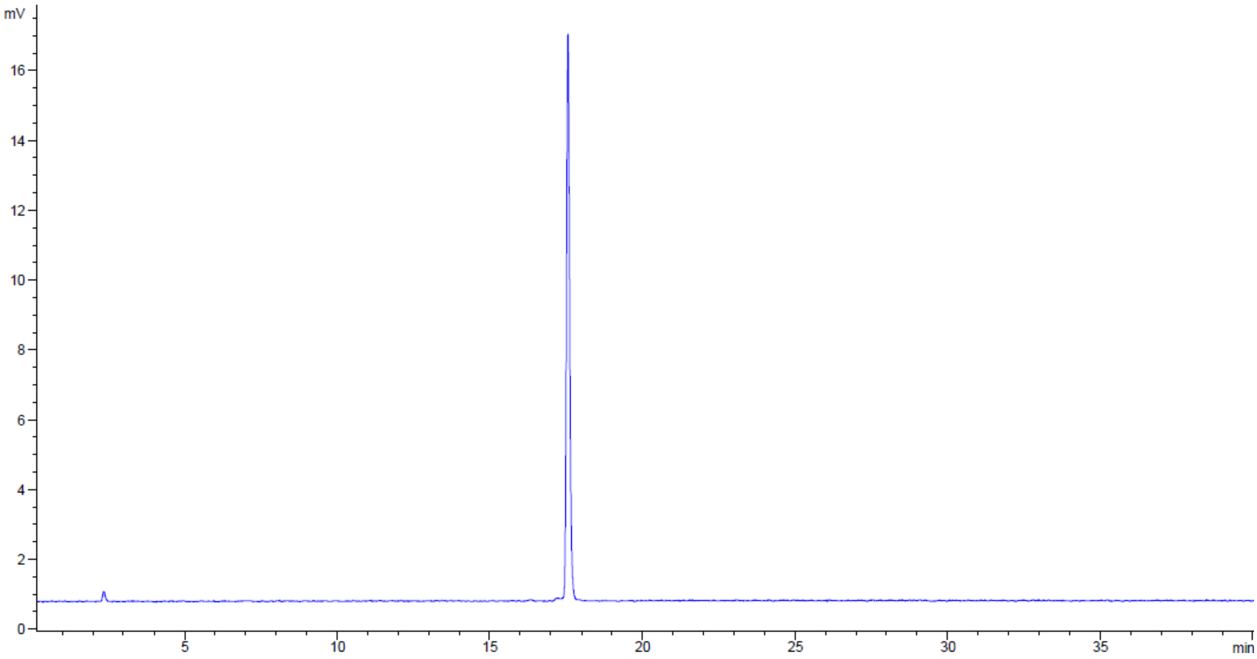

^{1}H NMR of AAABBB-OH (600 MHz, D_2O)

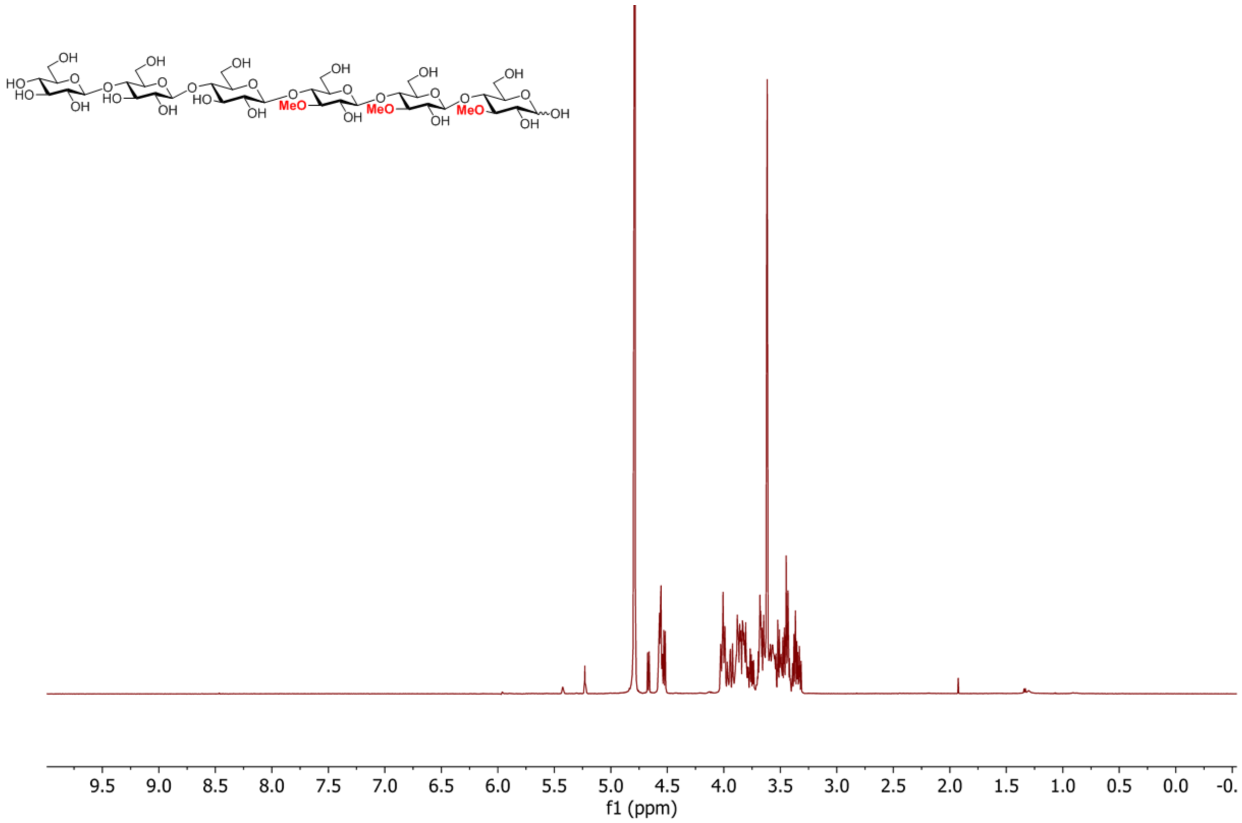

^{13}C NMR of AAABBB-OH (151 MHz, D_2O)

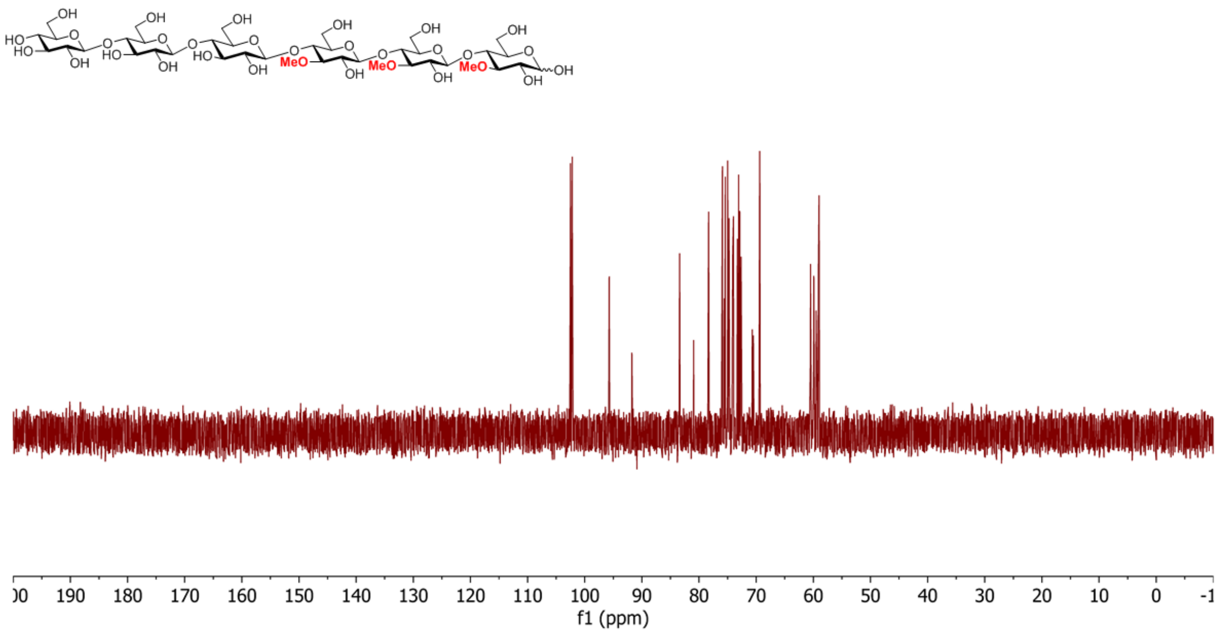

HSQC NMR of AAABBB-OH (D_2O)

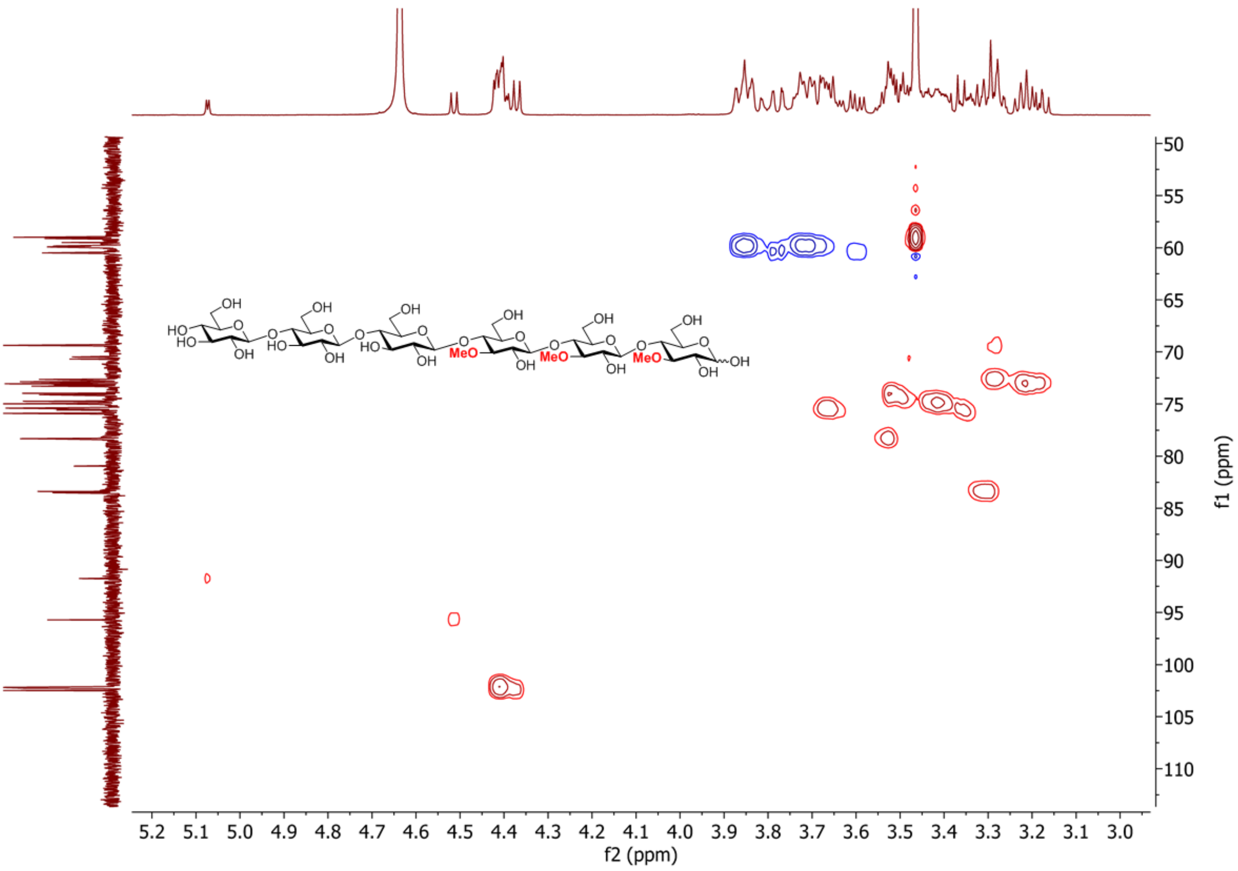

3.5.1.3. *Synthesis of ABAABA-OH*

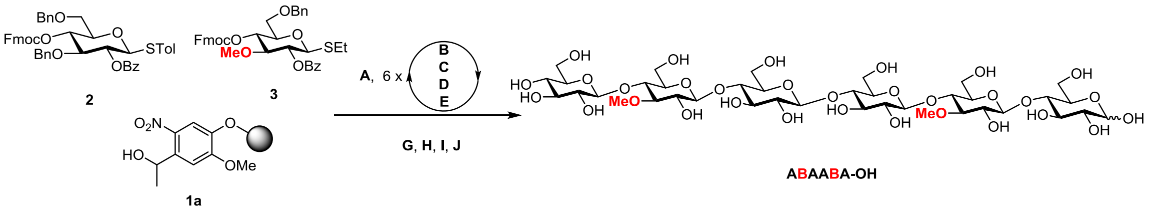

	Module	Conditions
	**A**: Resin Preparation for Synthesis	
2	**B**: Acidic Wash with TMSOTf Solution	
	**C**: Thioglycoside Glycosylation	**2**, 6.5 equiv.(-20°C for 5 min, 0°C for 20 min)
	**D**: Capping	
	**E**: Fmoc Deprotection	
	**B**: Acidic Wash with TMSOTf Solution	
	**C**: Thioglycoside Glycosylation	**3**, 6.5 equiv.(-20°C for 5 min, 0°C for 20 min)
	**D**: Capping	
	**E**: Fmoc Deprotection	
	**B**: Acidic Wash with TMSOTf Solution	
	**C**: Thioglycoside Glycosylation	**2**, 6.5 equiv.(-20°C for 5 min, 0°C for 20 min)
	**D**: Capping	
	**E**: Fmoc Deprotection	
	**F**: On-resin Methanolysis	
	**G**: Cleavage from Solid Support	
	**I**: Hydrogenolysis	
	**J**: Purification	**Method M**

Automated synthesis and global deprotection and purification afforded **ABAABA-OH** as white solid (4.9 mg, 39% overall yield).

Analytical data for **ABAABA-OH**: ^{1}H NMR (600 MHz, D_2O) δ 5.24 (d, *J* = 3.8 Hz, 0.38 H, α-H1), 4.67 (d, *J* = 7.9 Hz, 0.62 H, β-H1), 4.58 – 4.51 (m, 5H), 4.00 (ddt, *J* = 11.7, 6.5, 3.4 Hz, 4H), 3.97 – 3.93 (m, 1H), 3.92 (d, *J* = 1.8 Hz, 1H), 3.89 (d, *J* = 9.1 Hz, 1H), 3.87 – 3.81 (m, 6H), 3.79 (td, *J* = 12.0, 4.8 Hz, 2H), 3.68 (dq, *J* = 12.4, 3.8 Hz, 4H), 3.66 – 3.64 (m, 3H), 3.63 (s, 3H), 3.62 (s, 3H), 3.61 – 3.57 (m, 3H), 3.53 – 3.42 (m, 7H), 3.40 – 3.34 (m, 2H), 3.34 – 3.28 (m, 2H); ^{13}C NMR (151 MHz, D_2O) δ 102.32, 102.28, 102.28, 102.25, 102.19, 95.68 (β-C1), 91.74 (α-C1), 83.21, 83.19, 78.55, 78.41, 78.32, 78.20, 75.96, 75.60, 75.41, 75.34, 75.01, 74.97, 74.77, 74.73, 74.71, 74.18, 74.11, 73.94, 73.81, 73.50, 73.26, 72.87, 72.29, 72.24, 71.24, 71.15, 70.03, 69.41, 60.67, 59.96, 59.93, 59.82, 59.76, 59.16, 59.07, 59.02; $[\alpha]_D^{20}$ +13.27 (c 0.75, H_2O); IR (neat) ν_{max} = 3360, 2876, 1021 cm^{-1}; m/z (HRMS+) 1041.347 $[M + Na]^+$ ($C_{38}H_{66}NaO_{31}$ requires 1041.348).

RP-HPLC of ABAABA-OH (ELSD trace, Method C, t_R = 17.6 min)

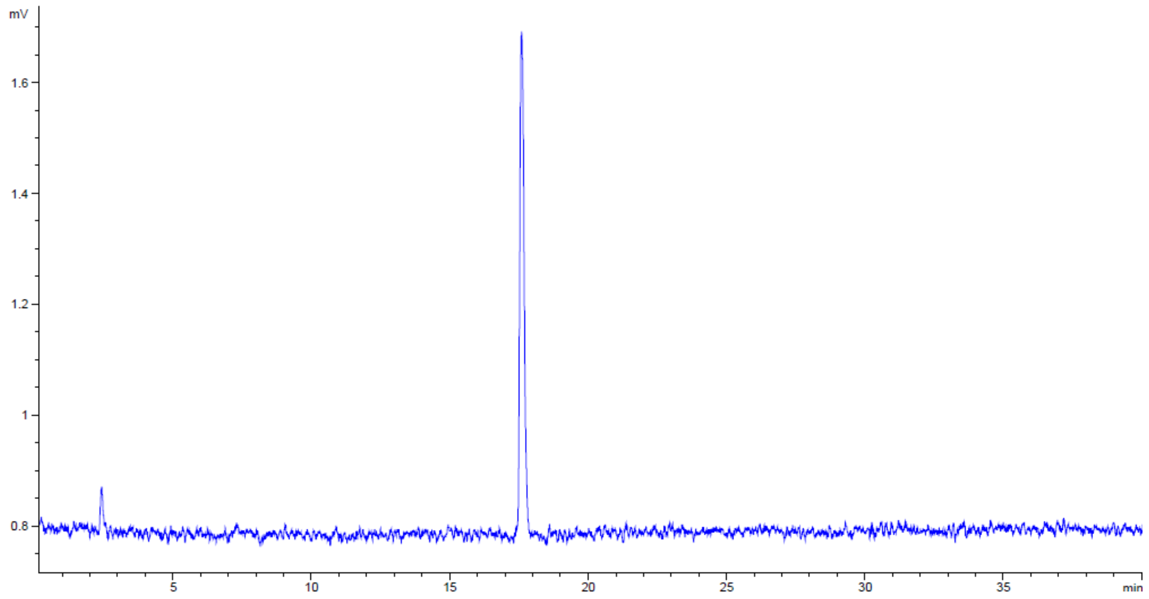

^{1}H NMR of ABAABA-OH (600 MHz, D_2O)

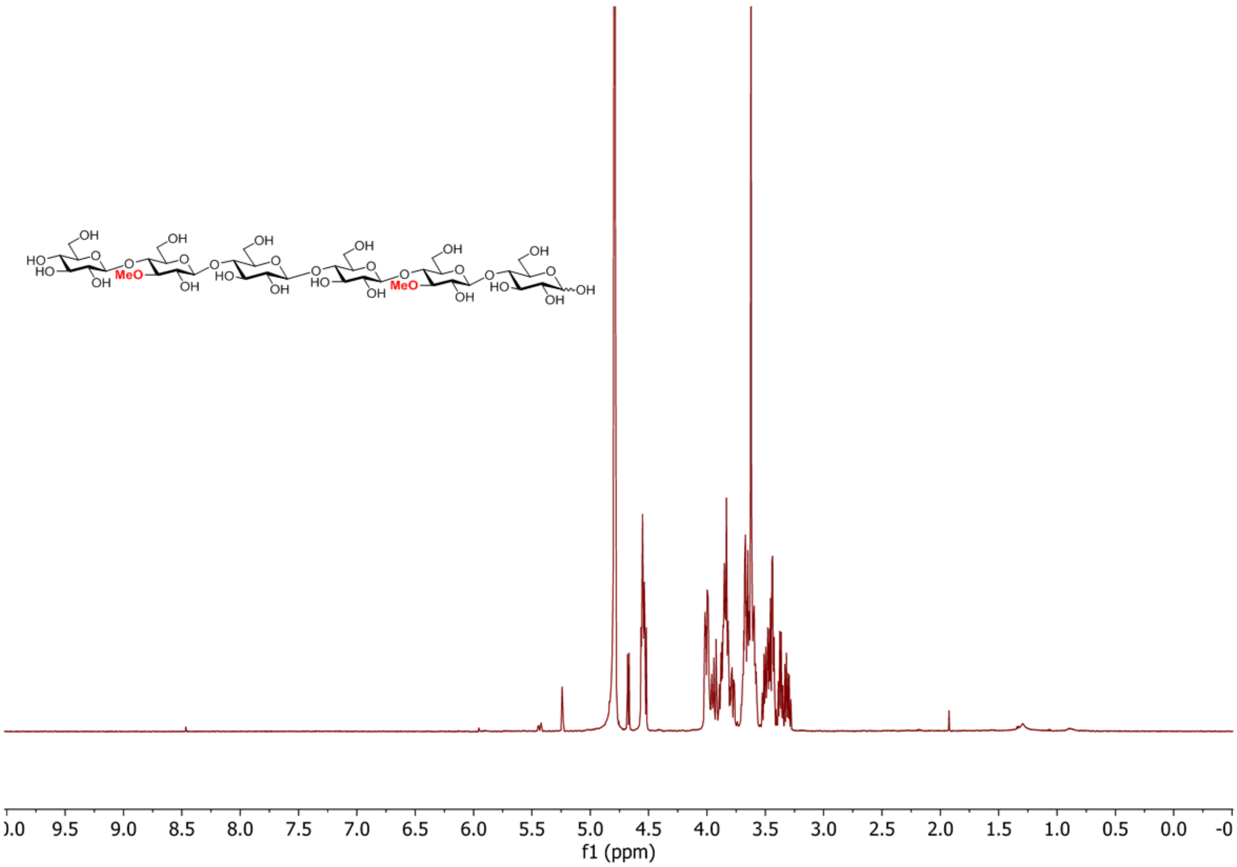

^{13}C NMR of ABAABA-OH (151 MHz, D_2O)

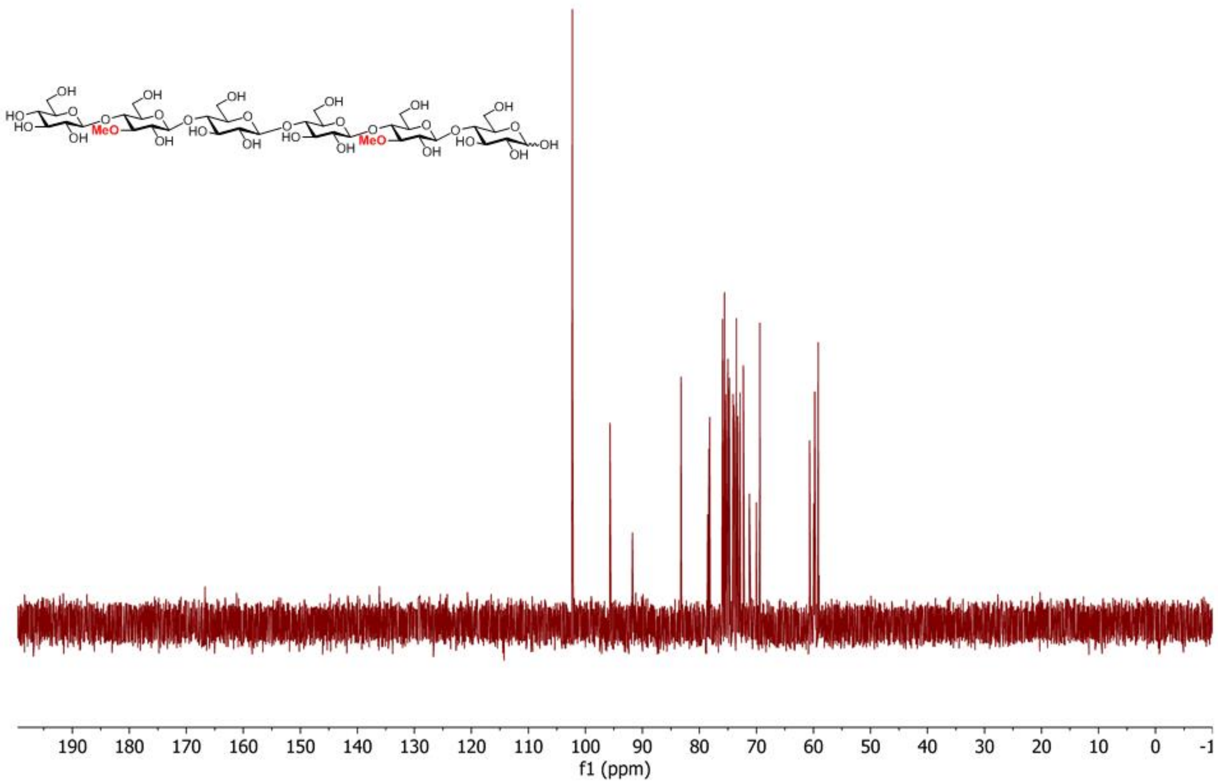

HSQC NMR of ABAABA-OH (D_2O)

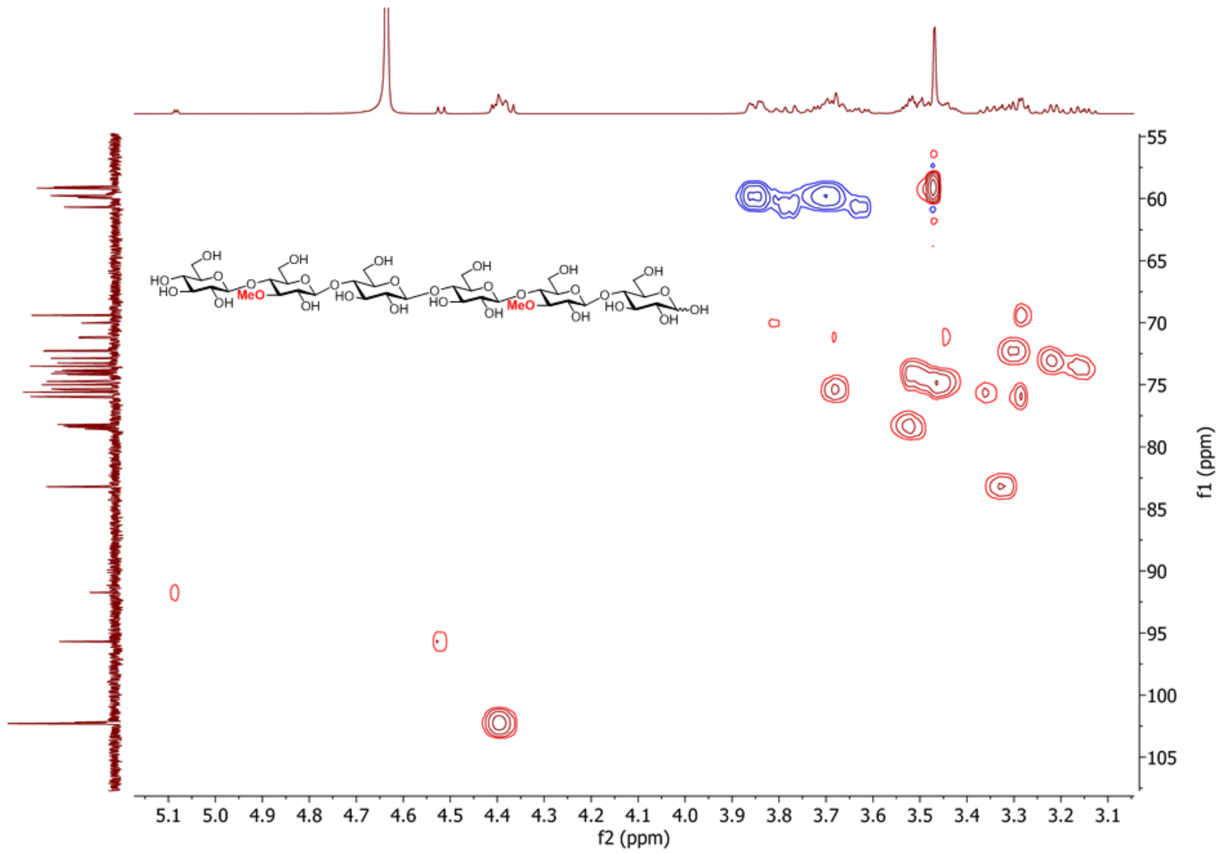

3.5.1.4. *Synthesis of ABABAB-OH*

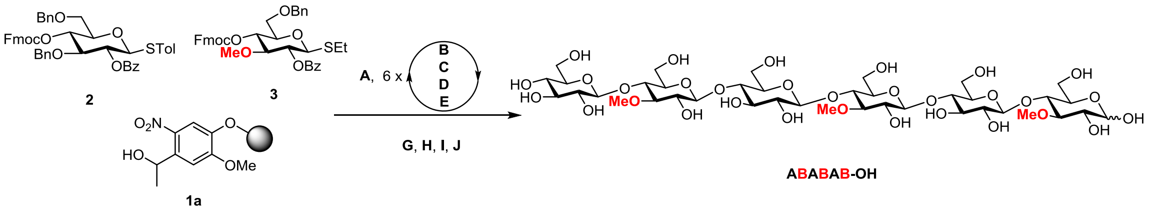

Module	Conditions
**A**: Resin Preparation for Synthesis	
**B**: Acidic Wash with TMSOTf Solution	
**C**: Thioglycoside Glycosylation	**3**, 6.5 equiv.(-20°C for 5 min, 0°C for 20 min)
**D**: Capping	
**E**: Fmoc Deprotection	
3 × **B**: Acidic Wash with TMSOTf Solution	
**C**: Thioglycoside Glycosylation	**2**, 6.5 equiv.(-20°C for 5 min, 0°C for 20 min)
**D**: Capping	
**E**: Fmoc Deprotection	
**F**: On-resin Methanolysis	
**G**: Cleavage from Solid Support	
**I**: Hydrogenolysis	
**J**: Purification	**Method M**

Automated synthesis and global deprotection and purification afforded **ABABAB-OH** as white solid (3.3 mg, 26% overall yield).

Analytical data for **ABABAB-OH**: ^{1}H NMR (400 MHz, D_2O) δ 5.14 (d, *J* = 3.3 Hz, 0.45 H, α-H1), 4.59 (d, *J* = 7.8 Hz, 0.55 H, β-H1), 4.51 – 4.41 (m, 5H), 3.93 (d, *J* = 12.3 Hz, 4H), 3.87 (d, *J* = 6.9 Hz, 1H), 3.84 – 3.76 (m, 5H), 3.76 – 3.66 (m, 5H), 3.63 – 3.56 (m, 4H), 3.56 – 3.53 (m, 9H), 3.52 (d, *J* = 5.9 Hz, 5H), 3.46 – 3.39 (m, 3H), 3.39 – 3.32 (m, 5H), 3.32 – 3.20 (m, 4H); ^{13}C NMR (101 MHz, D_2O) δ 102.30, 102.23, 102.19, 102.16, 102.15, 95.65 (β-C1), 91.69 (α-C1), 83.39, 83.15, 83.10, 80.86, 78.21, 75.92, 75.82, 75.52, 75.49, 75.35, 75.30, 74.94, 74.72, 74.05, 73.42, 73.24, 73.21, 73.19, 73.08, 72.27, 72.20, 70.62, 70.47, 69.34, 60.59, 59.87, 59.77, 59.66, 59.47, 59.17, 59.07, 59.05, 48.72; $[\alpha]_D^{20}$ +10.36 (c 0.2, H_2O); IR (neat) ν_{max} = 3348, 2929, 1651, 1031 cm^{-1}; m/z (HRMS+) 1055.361 $[M + Na]^+$ ($C_{39}H_{68}NaO_{31}$ requires 1055.364).

RP-HPLC of ABABAB-OH (ELSD trace, Method C, t_R = 16.9 min)

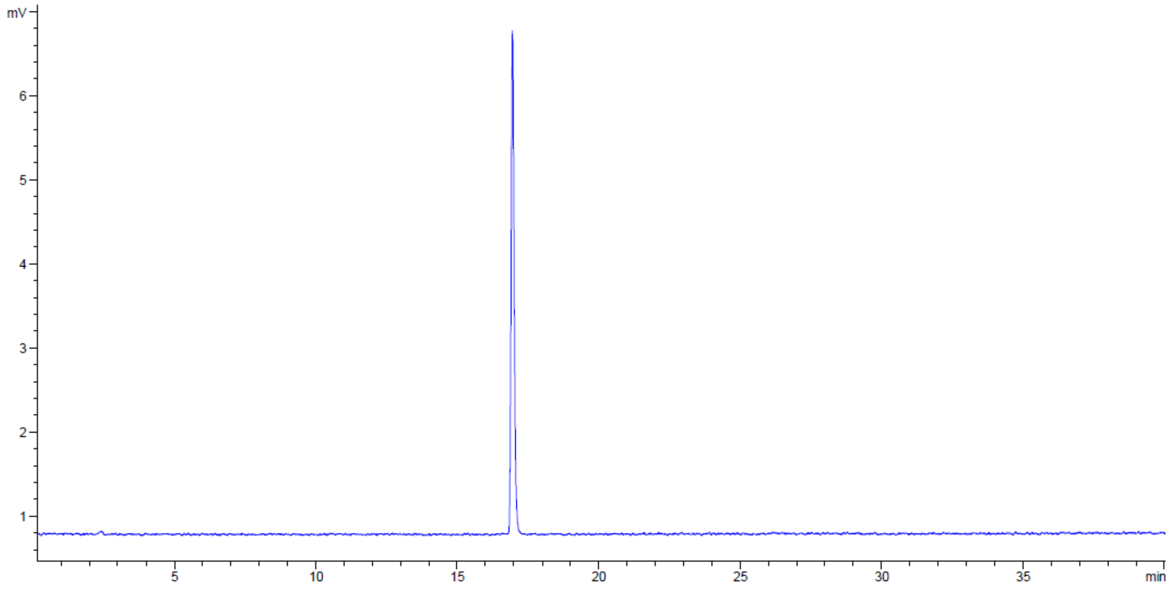

^{1}H NMR of ABABAB-OH (400 MHz, D_2O)

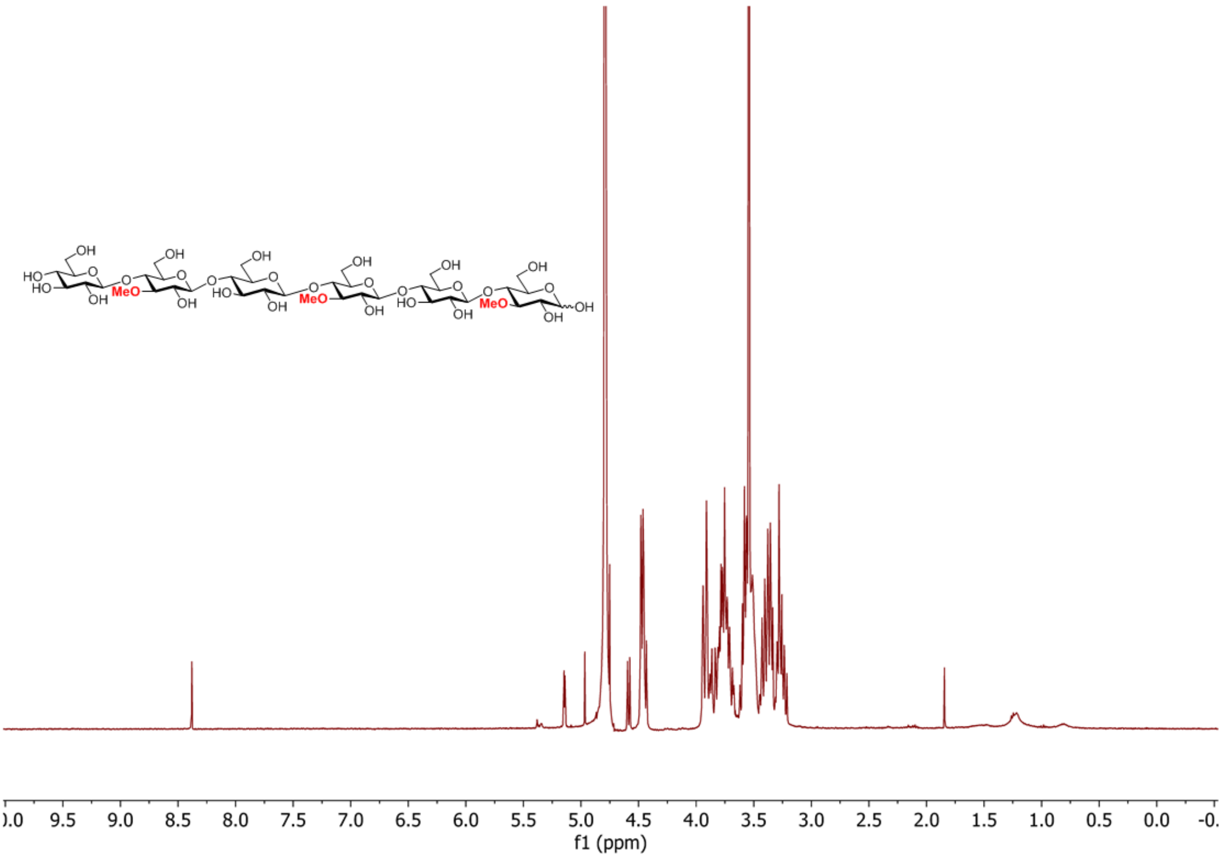

^{13}C NMR of ABABAB-OH (101 MHz, D_2O)

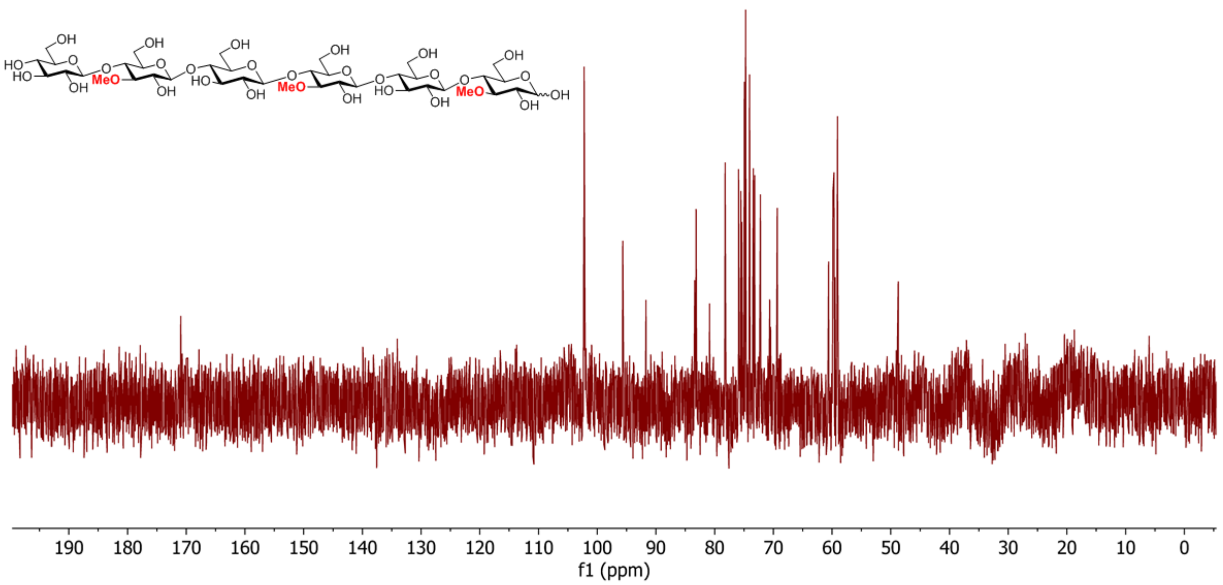

HSQC NMR of ABABAB-OH (D_2O)

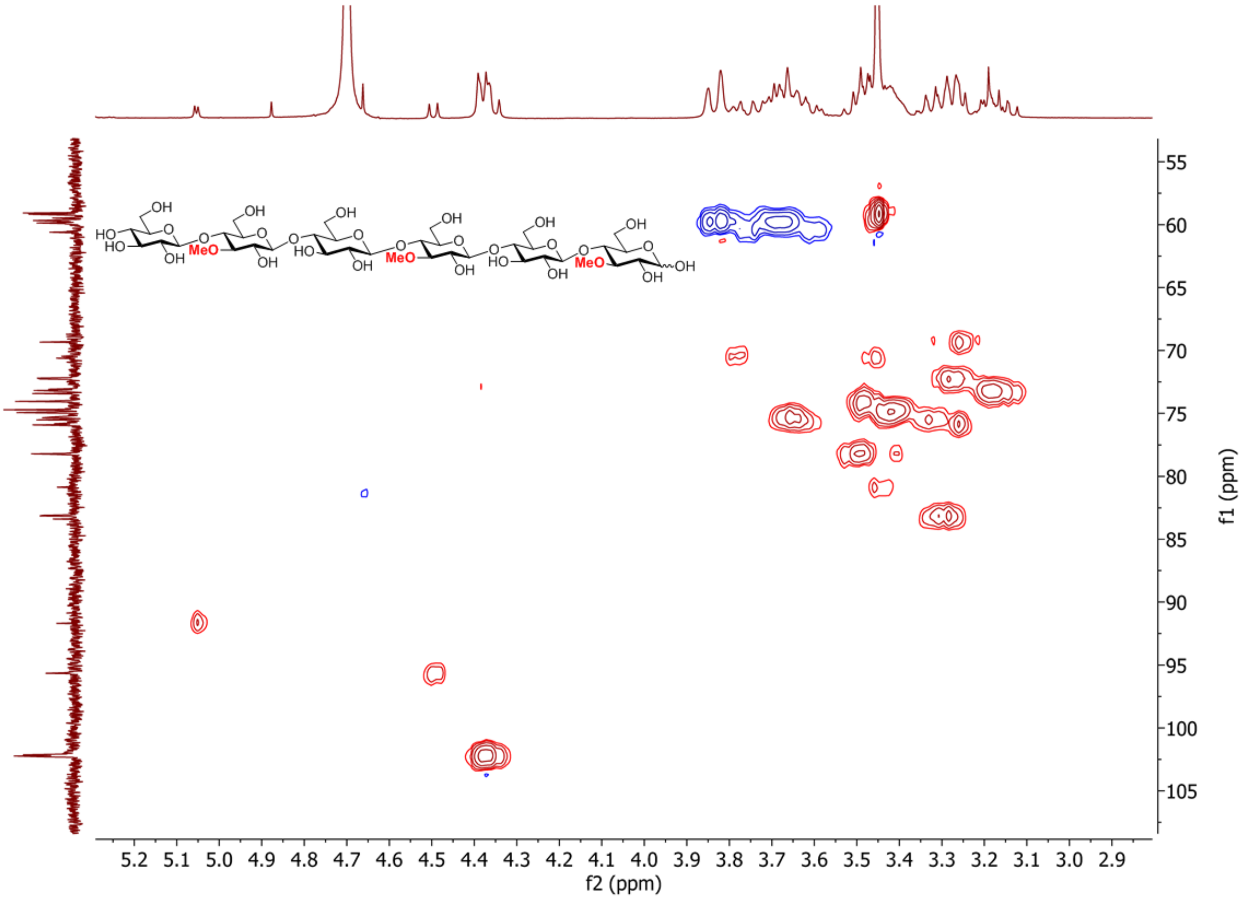

3.5.1.5. *Synthesis of ABACAB-OH*

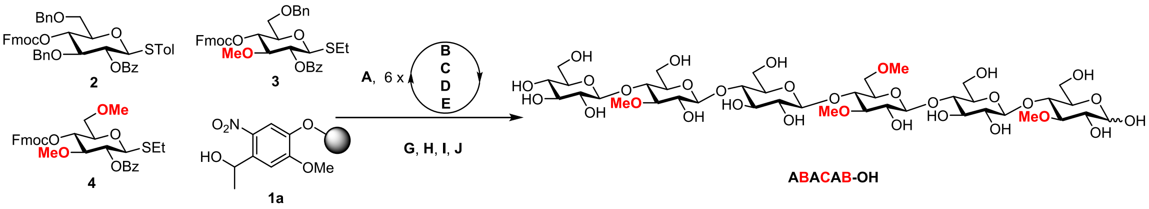

Module	Conditions
**A**: Resin Preparation for Synthesis	
**B**: Acidic Wash with TMSOTf Solution<br>**C**: Thioglycoside Glycosylation<br>**D**: Capping<br>**E**: Fmoc Deprotection	 **3**, 6.5 equiv.(-20°C for 5 min, 0°C for 20 min)
**B**: Acidic Wash with TMSOTf Solution<br>**C**: Thioglycoside Glycosylation<br>**D**: Capping<br>**E**: Fmoc Deprotection	 **2**, 6.5 equiv.(-20°C for 5 min, 0°C for 20 min)
**B**: Acidic Wash with TMSOTf Solution<br>**C**: Thioglycoside Glycosylation<br>**D**: Capping<br>**E**: Fmoc Deprotection	 **4**, 6.5 equiv.(-20°C for 5 min, 0°C for 20 min)
**B**: Acidic Wash with TMSOTf Solution<br>**C**: Thioglycoside Glycosylation<br>**D**: Capping<br>**E**: Fmoc Deprotection	 **2**, 6.5 equiv.(-20°C for 5 min, 0°C for 20 min)
**B**: Acidic Wash with TMSOTf Solution<br>**C**: Thioglycoside Glycosylation<br>**D**: Capping<br>**E**: Fmoc Deprotection	 **3**, 6.5 equiv.(-20°C for 5 min, 0°C for 20 min)
**B**: Acidic Wash with TMSOTf Solution<br>**C**: Thioglycoside Glycosylation<br>**D**: Capping<br>**E**: Fmoc Deprotection	 **2**, 6.5 equiv.(-20°C for 5 min, 0°C for 20 min)
**F**: On-resin Methanolysis<br>**G**: Cleavage from Solid Support<br>**I**: Hydrogenolysis<br>**J**: Purification	 <br><br>**Method M**

Automated synthesis and global deprotection and purification afforded **ABACAB-OH** as white solid (9.5 mg, 73% overall yield).

Analytical data for **ABACAB-OH**: ^{1}H NMR (600 MHz, D_2O) δ 5.23 (d, *J* = 3.5 Hz, 0.39 H, α-H1), 4.67 (d, *J* = 7.9, Hz, 0.61H, β-H1), 4.58 – 4.51 (m, 4H), 4.46 (d, *J* = 7.9 Hz, 1H), 4.01 (ddd, *J* = 12.8, 5.8, 2.8 Hz, 3H), 3.98 – 3.88 (m, 3H), 3.88 – 3.76 (m, 10H), 3.73 – 3.67 (m, 2H), 3.67 – 3.64 (m, 4H), 3.64 – 3.61 (m, 9H), 3.60 – 3.54 (m, 3H), 3.54 – 3.47 (m, 3H), 3.45 (ddd, *J* = 10.6, 5.1, 2.8 Hz, 4H), 3.42 (s, 3H), 3.40 – 3.35 (m, 3H), 3.34 – 3.30 (m, 1H); ^{13}C NMR (151 MHz, D_2O) δ 102.32, 102.29, 102.23, 102.21, 102.15, 95.71 (β-C1), 91.75 (α-C1), 83.45, 83.23, 83.01, 80.90, 78.70, 78.66, 78.37, 75.96, 75.88, 75.60, 75.55, 75.36, 75.31, 75.00, 74.98, 74.80, 74.68, 74.16, 73.50, 73.45, 73.26, 73.23, 73.15, 73.14, 72.31, 72.11, 70.66, 70.52, 69.91, 69.41, 60.67, 59.97, 59.95, 59.87, 59.77, 59.43, 59.17, 59.03, 58.31; $[\alpha]_D^{20}$ +14.66 (c 1, H_2O); IR (neat) ν_{max} = 3361, 2953, 1035 cm^{-1}; m/z (HRMS+) 1069.379 $[M + Na]^+$ ($C_{40}H_{70}NaO_{31}$ requires 1069.379).

RP-HPLC of ABACAB-OH (ELSD trace, Method C, t_R = 18.6, 18.7 min)

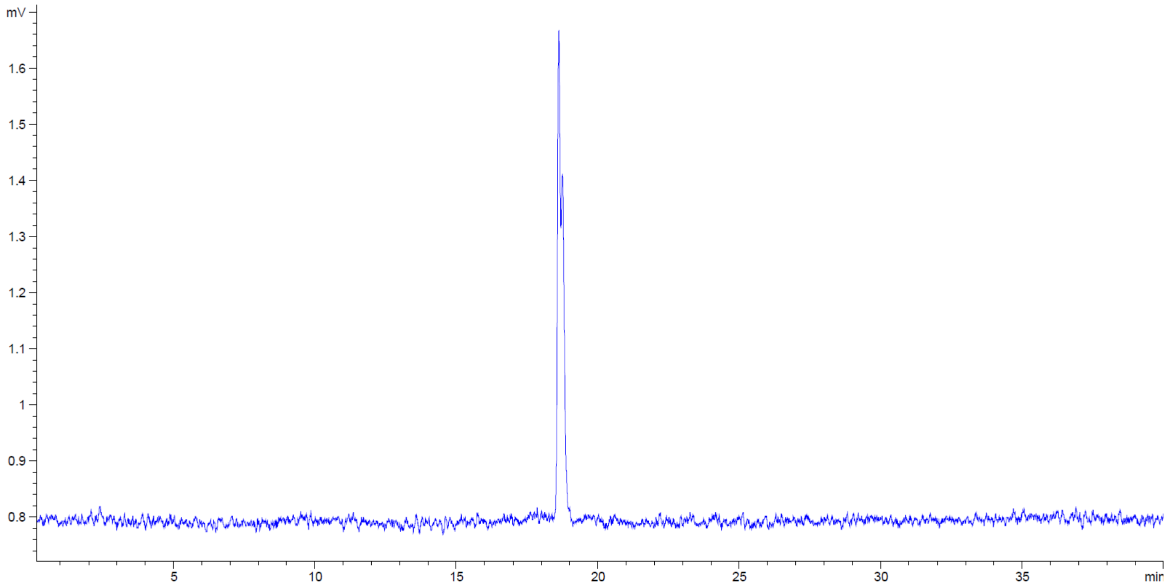

^{1}H NMR of ABACAB-OH (600 MHz, D_2O)

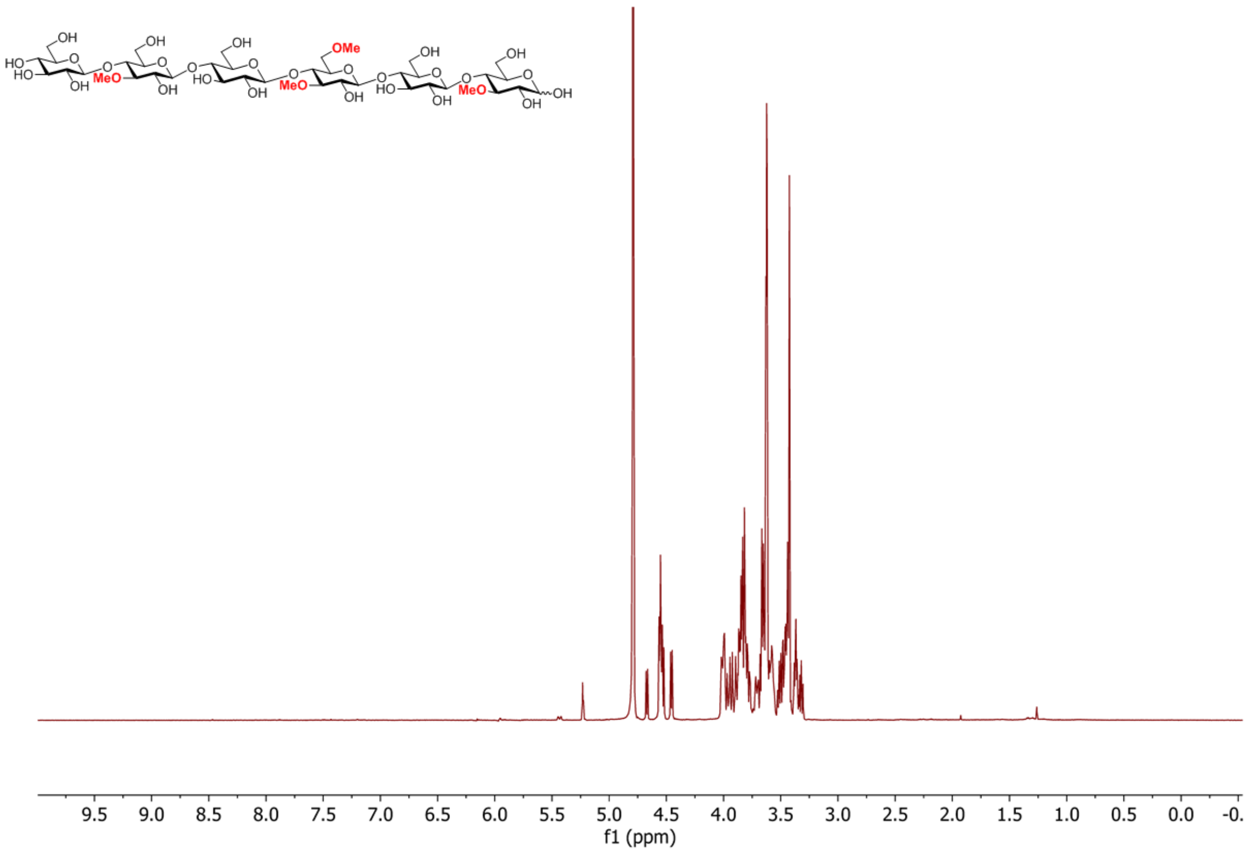

^{13}C NMR of ABACAB-OH (151 MHz, D_2O)

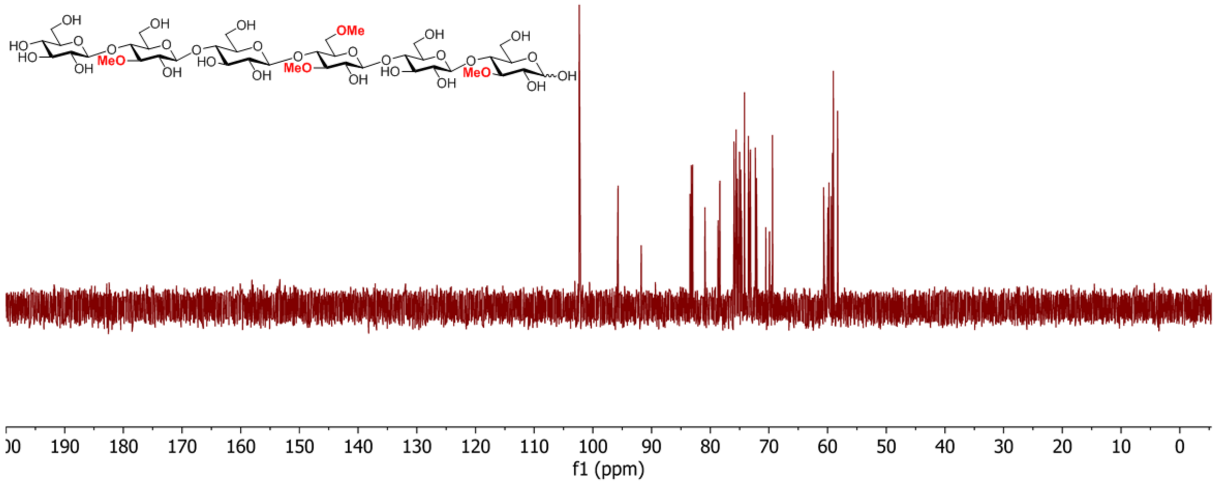

HSQC NMR of ABACAB-OH (D_2O)

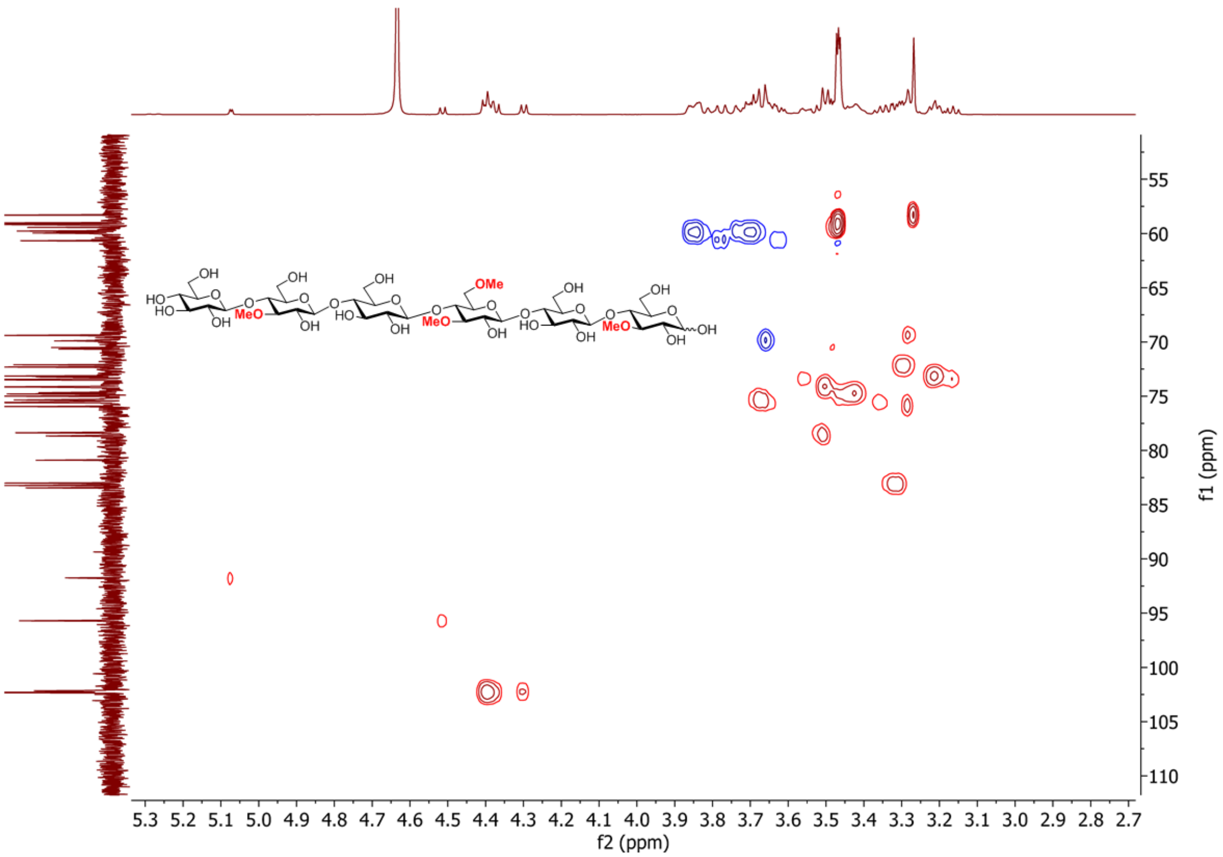

3.5.1.6. *Synthesis of ACAACA-OH*

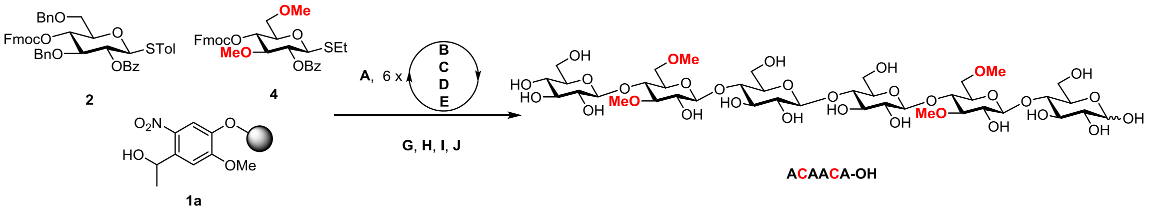

	Module	Conditions
	**A**: Resin Preparation for Synthesis	
	**B**: Acidic Wash with TMSOTf Solution	
	**C**: Thioglycoside Glycosylation	**2**, 6.5 equiv.(-20°C for 5 min, 0°C for 20 min)
	**D**: Capping	
	**E**: Fmoc Deprotection	
	**B**: Acidic Wash with TMSOTf Solution	
	**C**: Thioglycoside Glycosylation	**4**, 6.5 equiv.(-20°C for 5 min, 0°C for 20 min)
2	**D**: Capping	
	**E**: Fmoc Deprotection	
	**B**: Acidic Wash with TMSOTf Solution	
	**C**: Thioglycoside Glycosylation	**2**, 6.5 equiv.(-20°C for 5 min, 0°C for 20 min)
	**D**: Capping	
	**E**: Fmoc Deprotection	
	**F**: On-resin Methanolysis	
	**G**: Cleavage from Solid Support	
	**I**: Hydrogenolysis	
	**J**: Purification	**Method M**

Automated synthesis and global deprotection and purification afforded **ACAACA-OH** as white solid (5.9 mg, 45% overall yield).

Analytical data for **ACAACA-OH**: ^{1}H NMR (600 MHz, D_2O) δ 5.24 (d, *J* = 3.8 Hz, 0.39 H, α-H1), 4.67 (d, *J* = 8.0 Hz, 0.61 H, β-H1), 4.55 (dd, *J* = 15.8, 7.7 Hz, 3H), 4.48 – 4.41 (m, 2H), 4.02 – 3.96 (m, 2H), 3.95 (dd, *J* = 6.1, 1.8 Hz, 1H), 3.91 (dd, *J* = 9.5, 1.9 Hz, 1H), 3.90 – 3.86 (m, 2H), 3.86 – 3.82 (m, 3H), 3.82 (t, *J* = 2.1 Hz, 3H), 3.81 – 3.78 (m, 2H), 3.78 – 3.75 (m, 1H), 3.73 – 3.68 (m, 3H), 3.68 – 3.63 (m, 6H), 3.63 - 3.62 (m, 6H), 3.61 – 3.54 (m, 2H), 3.53 – 3.50 (m, 1H), 3.50 - 3.48 (m, 1H), 3.48 – 3.47 (m, 1H), 3.46 – 3.43 (m, 3H), 3.43 – 3.41 (m, 6H), 3.40 – 3.36 (m, 2H), 3.36 – 3.32 (m, 1H), 3.32 – 3.28 (m, 1H); ^{13}C NMR (151 MHz, D_2O) δ 102.36, 102.31, 102.31, 102.27, 102.24, 95.67 (β-C1), 91.74 (α-C1), 83.04, 83.01, 78.86, 78.73, 78.52, 78.34, 76.02, 75.64, 75.31, 75.23, 74.81, 74.65, 74.63, 74.22, 74.14, 73.99, 73.76, 73.48, 73.44, 73.40, 73.16, 72.81, 72.16, 72.11, 71.26, 71.10, 69.93, 69.42, 60.66, 59.97, 59.91, 59.83, 59.82, 59.12, 59.02, 58.97, 58.30; $[\alpha]_D^{20}$ +14.00 (c 0.8, H_2O); IR (neat) ν_{max} = 3387, 2925, 1650, 1064 cm^{-1}; m/z (HRMS+) 1069.379 $[M + Na]^+$ ($C_{40}H_{70}NaO_{31}$ requires 1069.379).

RP-HPLC of ACAACA-OH (ELSD trace, Method C, t_R = 18.8 min)

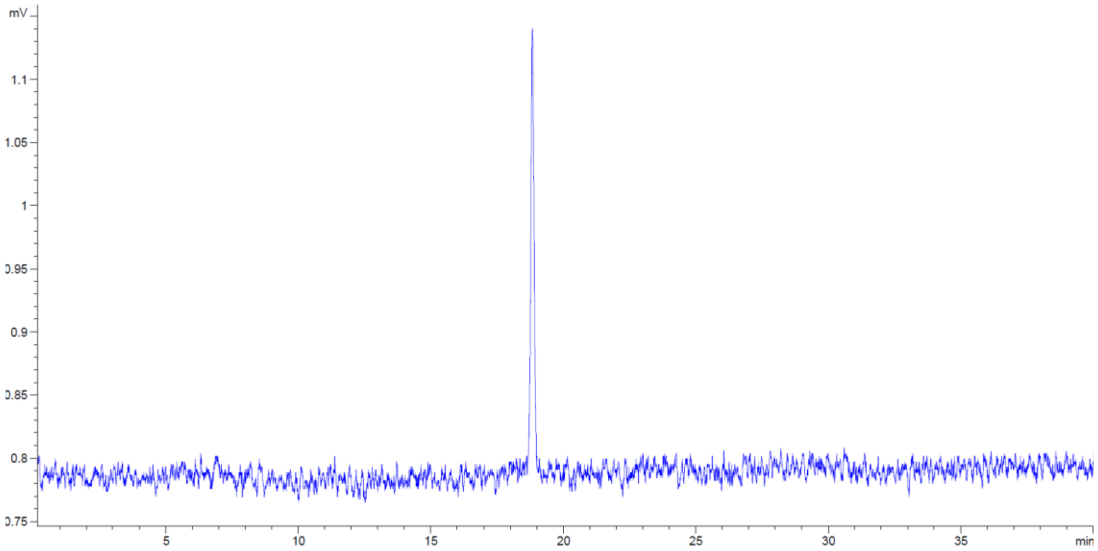

^1H NMR of ACAACA-OH (600 MHz, D_2O)

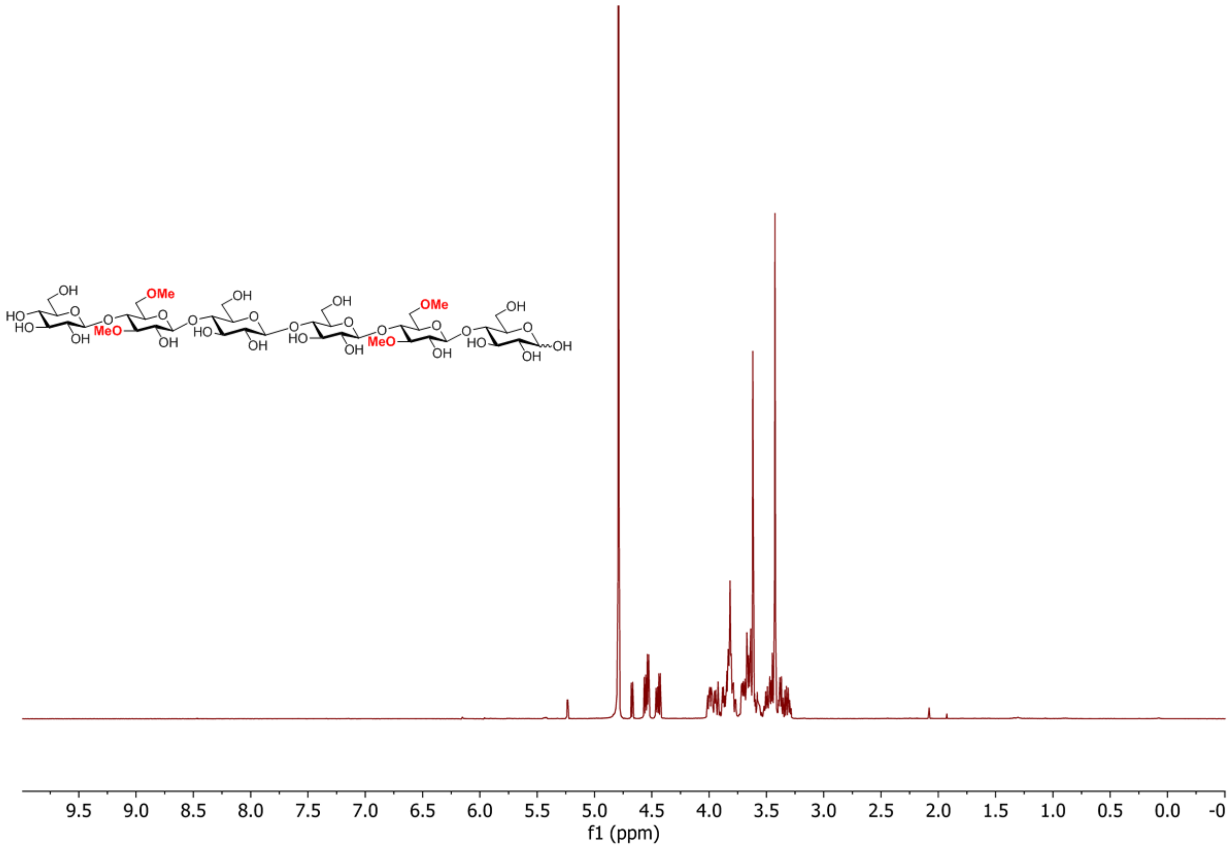

^{13}C NMR of ACAACA-OH (151 MHz, D_2O)

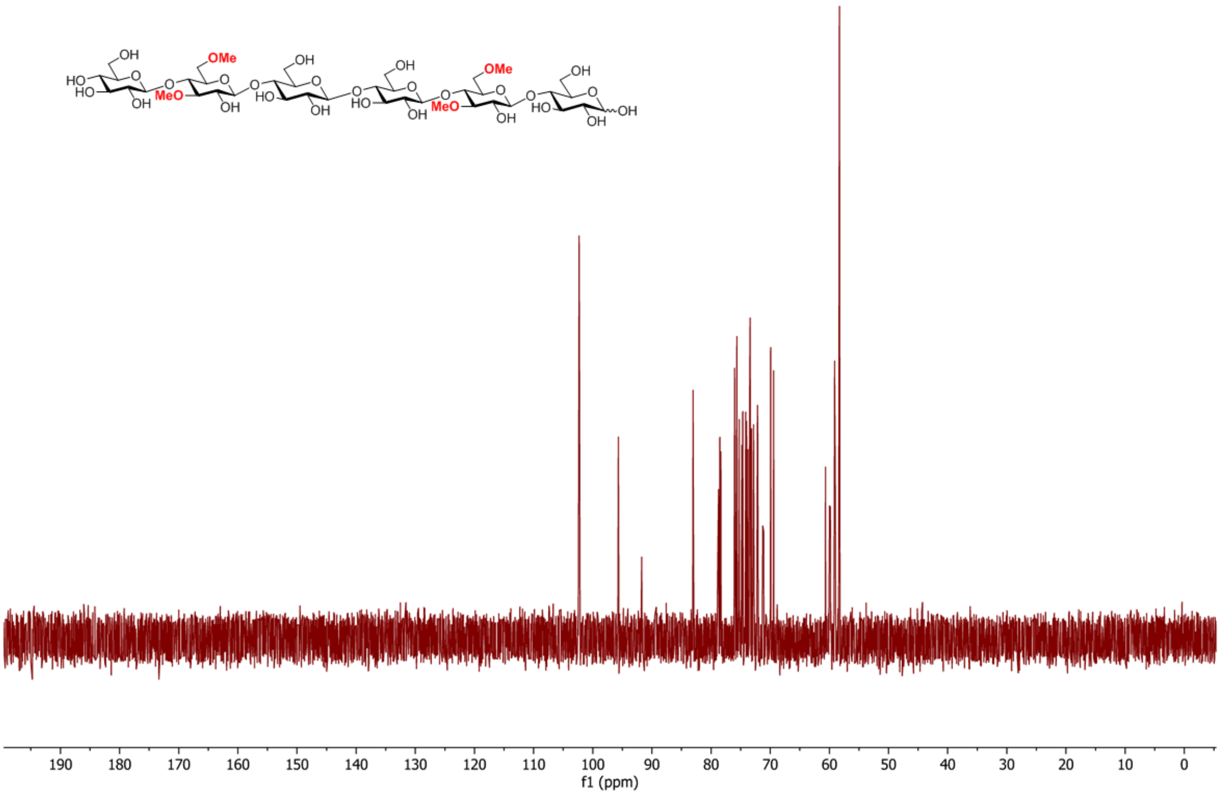

HSQC NMR of ACAACA-OH (D_2O)

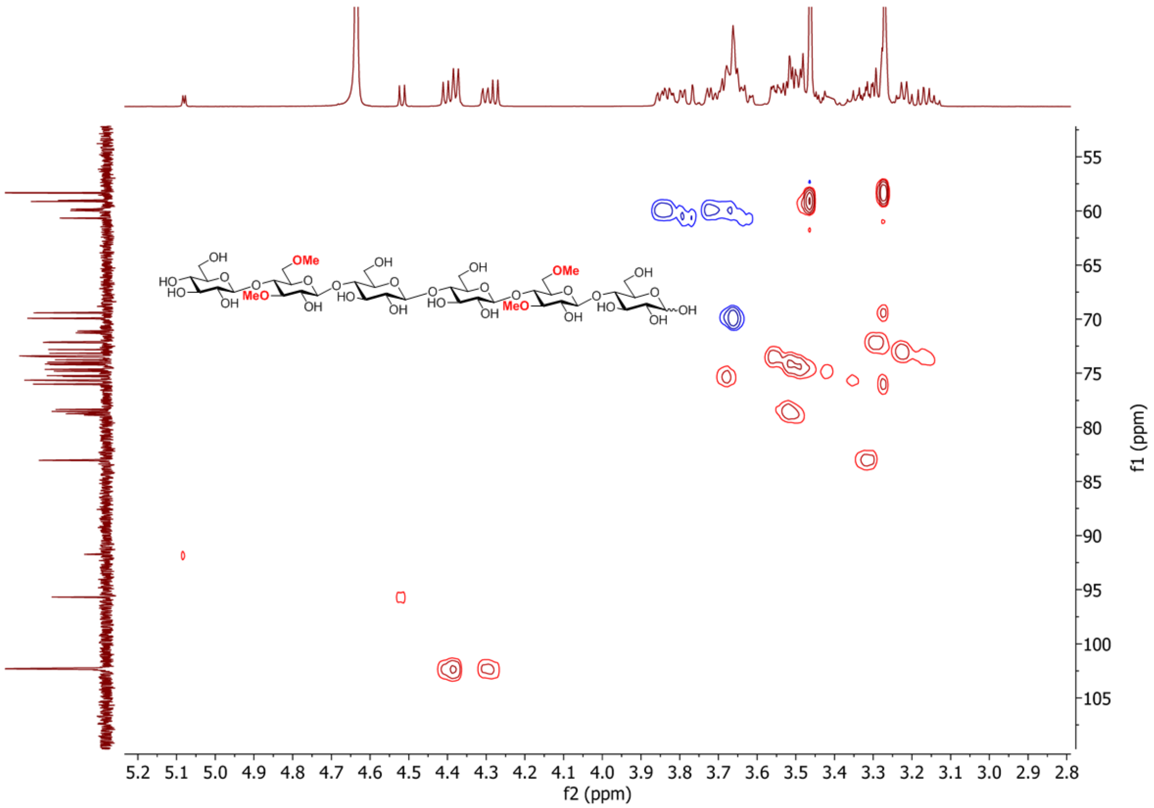

3.5.1.7. ***Synthesis of ACACAC-OH***

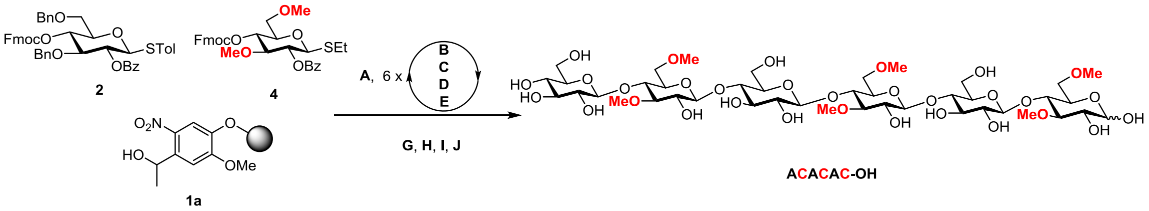

Module	Conditions
**A**: Resin Preparation for Synthesis	
**B**: Acidic Wash with TMSOTf Solution	
**C**: Thioglycoside Glycosylation	**4**, 6.5 equiv.(-20°C for 5 min, 0°C for 20 min)
**D**: Capping	
**E**: Fmoc Deprotection	
3 × **B**: Acidic Wash with TMSOTf Solution	
**C**: Thioglycoside Glycosylation	**2**, 6.5 equiv.(-20°C for 5 min, 0°C for 20 min)
**D**: Capping	
**E**: Fmoc Deprotection	
**F**: On-resin Methanolysis	
**G**: Cleavage from Solid Support	
**I**: Hydrogenolysis	
**J**: Purification	**Method M**

Automated synthesis and global deprotection and purification afforded **ACACAC-OH** as white solid (4.9 mg, 36% overall yield).

Analytical data for **ACACAC-OH**: ^{1}H NMR (600 MHz, D_2O) δ 5.21 (d, *J* = 3.6 Hz, 0.43 H, α-H1), 4.66 (d, *J* = 7.9 Hz, 0.57 H, β-H1), 4.55 (d, *J* = 7.5 Hz, 2H), 4.47 – 4.41 (m, 3H), 4.06 – 4.02 (m, 1H), 4.00 (dd, *J* = 12.4, 2.1 Hz, 2H), 3.93 (dd, *J* = 12.3, 1.5 Hz, 1H), 3.88 – 3.83 (m, 4H), 3.83 – 3.79 (m, 6H), 3.77 (d, *J* = 5.2 Hz, 1H), 3.71 (ddd, *J* = 9.8, 4.4, 2.5 Hz, 3H), 3.69 – 3.63 (m, 5H), 3.63 – 3.60 (m, 9H), 3.57 (q, *J* = 6.1, 5.2 Hz, 2H), 3.53 – 3.43 (m, 6H), 3.43 – 3.41 (m, 6H), 3.41 (d, *J* = 6.2 Hz, 3H), 3.39 – 3.37 (m, 1H), 3.37 (s, 3H), 3.35 – 3.30 (m, 1H); ^{13}C NMR (151 MHz, D_2O) δ 102.36, 102.34, 102.25, 102.22, 102.18, 95.78 (β-C1), 91.72 (α-C1), 83.30, 83.06, 83.01, 80.76, 78.68, 76.02, 75.81, 75.64, 75.41, 75.30, 75.24, 74.73, 74.21, 74.19, 73.62, 73.47, 73.44, 73.40, 73.10, 73.00, 72.18, 72.11, 70.56, 70.03, 69.94, 69.91, 69.42, 69.17, 60.66, 59.96, 59.40, 59.13, 59.03, 58.98, 58.30, 58.20, 58.08; $[\alpha]_D^{20}$ +19.86 (c 0.75, H_2O); IR (neat) ν_{max} = 3395, 2928, 1648, 1066 cm^{-1}; m/z (HRMS+) 1097.409 $[M + Na]^+$ ($C_{42}H_{74}NaO_{31}$ requires 1097.411).

RP-HPLC of ACACAC-OH (ELSD trace, Method C, t_R = 21.0, 21.3 min)

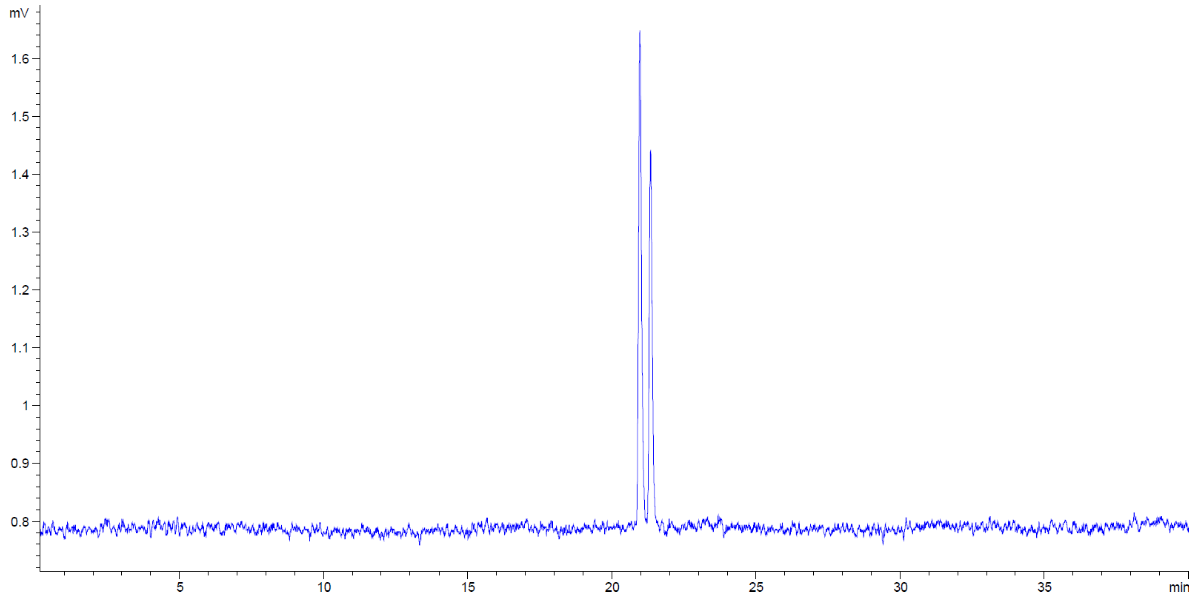

^{1}H NMR of ACACAC-OH (600 MHz, D_2O)

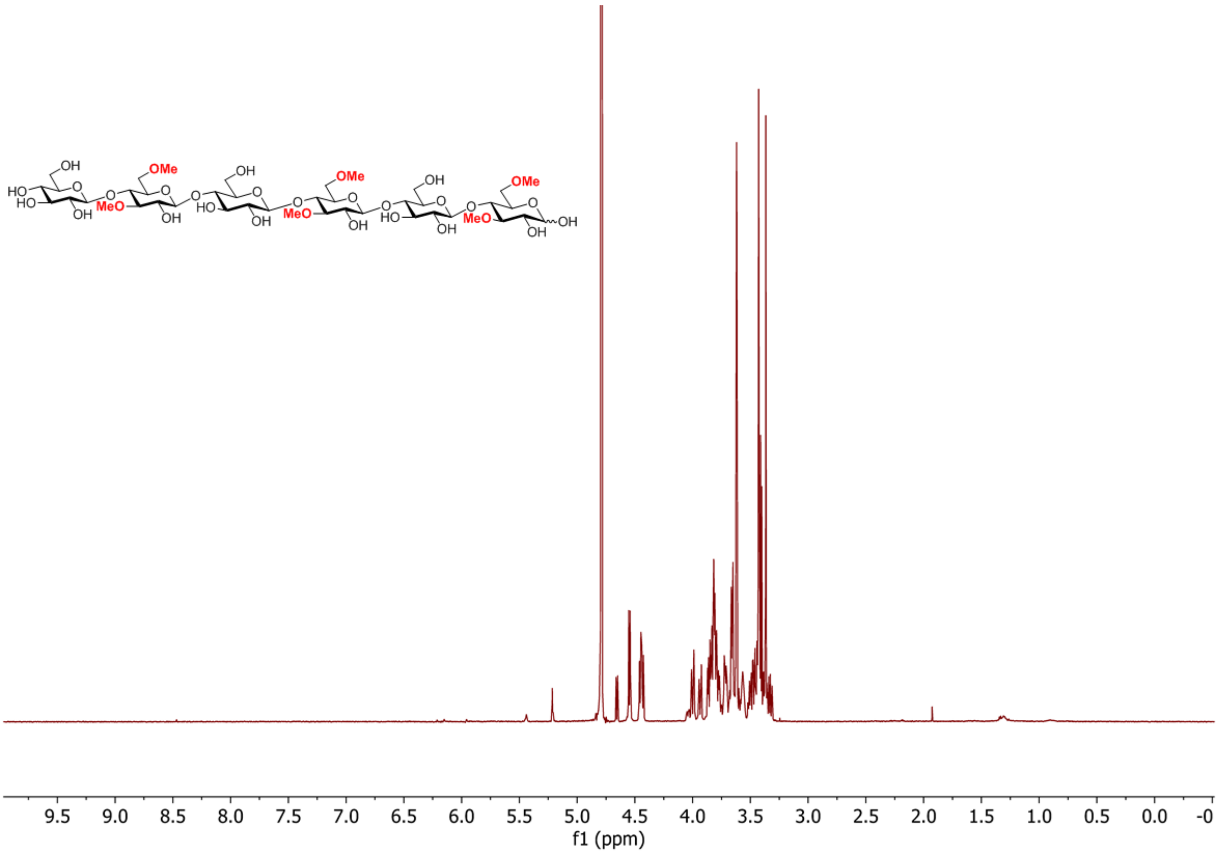

^{13}C NMR of ACACAC-OH (151 MHz, D_2O)

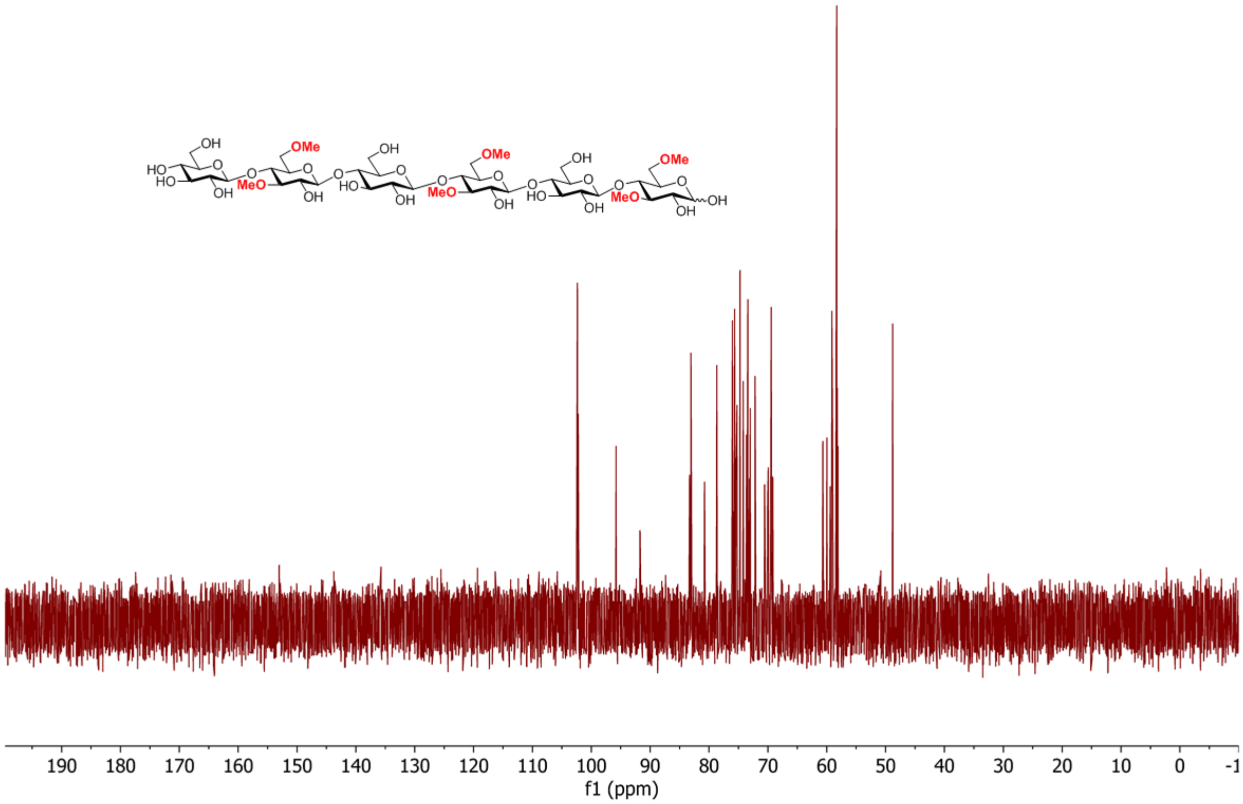

HSQC NMR of ACACAC-OH (D_2O)

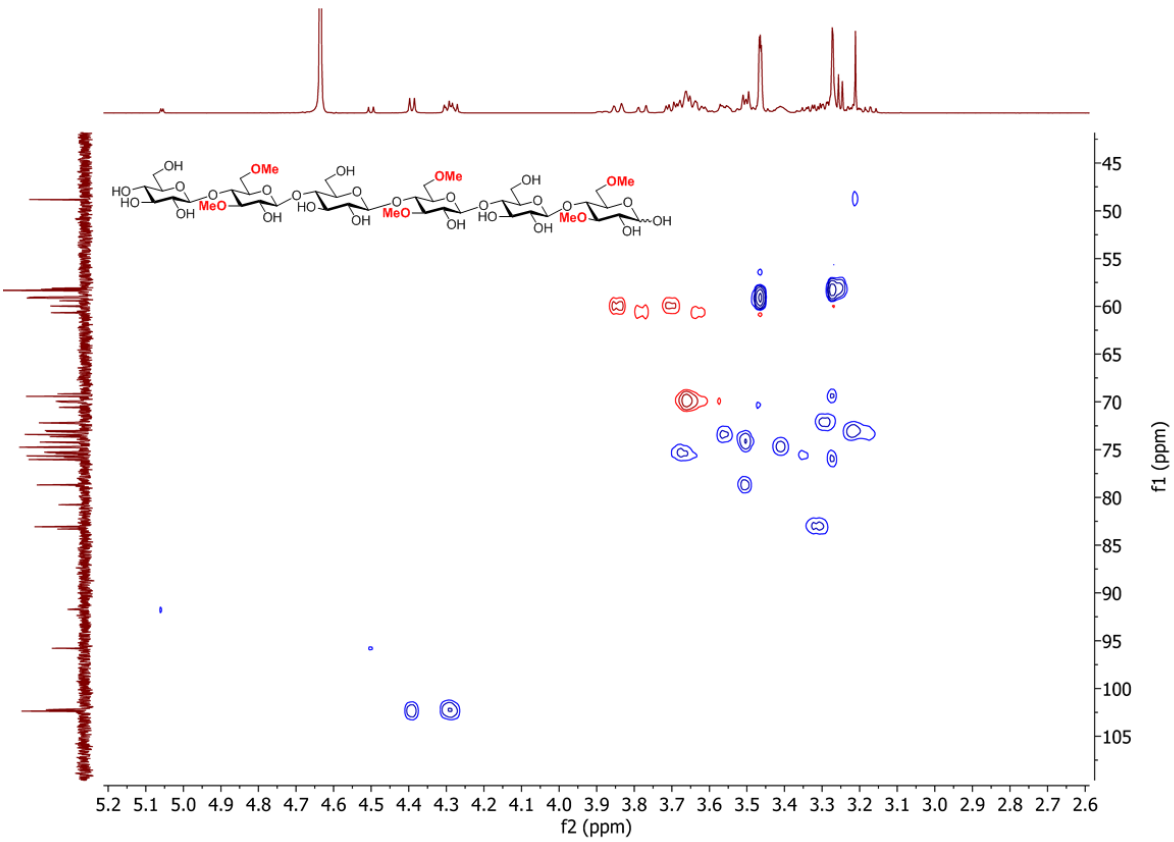

3.5.1.8. *Synthesis of AFAAFA-OH*

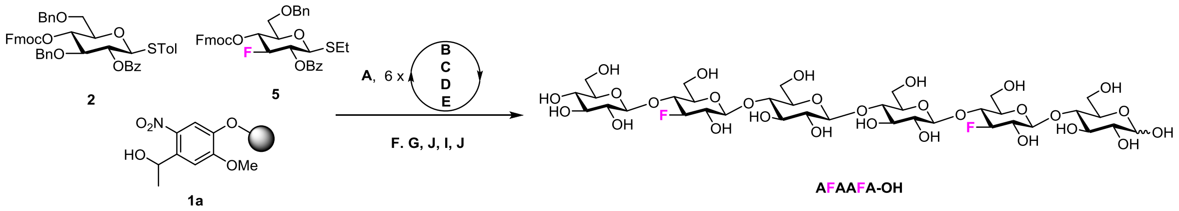

	Module	Conditions
	**A**: Resin Preparation for Synthesis	
	**B**: Acidic Wash with TMSOTf Solution	
	**C**: Thioglycoside Glycosylation	**2**, 6.8 equiv.(-20° for 5 min, 0° for 20 min)
	**D**: Capping	
	**E**: Fmoc Deprotection	
	**B**: Acidic Wash with TMSOTf Solution	
	**C**: Thioglycoside Glycosylation	**5**, 6.0 equiv.(-20° for 5 min, 0° for 20 min)
2	**D**: Capping	
	**E**: Fmoc Deprotection	
	**B**: Acidic Wash with TMSOTf Solution	
	**C**: Thioglycoside Glycosylation	**2**, 6.8 equiv.(-20° for 5 min, 0° for 20 min)
	**D**: Capping	
	**E**: Fmoc Deprotection	
	**F**: On-resin Methanolysis	
	**G**: Cleavage from Solid Support	
	**J**: Purification	**Method B1:** t_R = 34.2 and 34.7 min
	**I**: Hydrogenolysis	
	**J**: Purification	**Method D:** t_R = 14.9 min

Automated synthesis, global deprotection, and purification afforded **AFAAFA-OH** as white solid (1.1 mg, 9% overall yield).

Analytical data for **AFAAFA-OH**: ^{1}H NMR (600 MHz, D_2O) δ 5.24 (d, *J* = 3.8 Hz, 0.3H, α-H1), 4.70 – 4.52 (m, 7.7H, H3, H3, β-H1), 4.09 – 3.75 (m, 15H), 3.75 – 3.56 (m, 14H), 3.52 (t, *J* = 9.2 Hz, 1H), 3.47 (ddd, *J* = 10.0, 5.9, 2.3 Hz, 1H), 3.43 – 3.26 (m, 4H); ^{13}C NMR (151 MHz, D_2O) δ 102.35, 102.28, 102.17, 101.45, 101.38, 95.70 (β-C1), 94.32 (d, *J* = 184.1 Hz, C3), 91.80 (α-C1), 78.37, 78.08, 75.99, 75.51, 74.83, 74.70, 74.12, 74.03, 73.87, 73.84, 73.19, 72.97, 72.88, 71.71, 70.03, 69.54, 60.60, 59.88, 59.54; ^{19}F NMR (564 MHz, D_2O) δ -192.32 – -192.60 (m); $[\alpha]_D^{20}$ +16.07 (c 0.1, H_2O); IR (neat) ν_{max} = 3394, 2951, 1594, 1033 cm^{-1}; (ESI-HRMS+) m/z 1017.308 $[M+Na]^+$ ($C_{36}H_{60}F_2O_{29}Na$ requires 1017.308).

RP-HPLC of AFAAFA-OH (ELSD trace, Method D, t_R = 14.9 min)

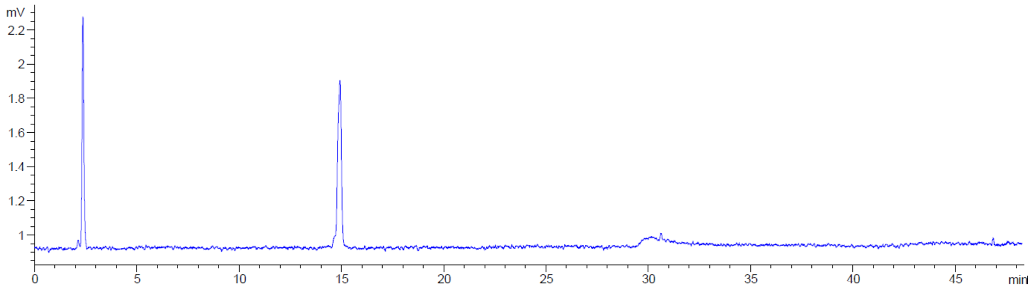

^{1}H NMR of AFAAFA-OH (600 MHz, D_2O)

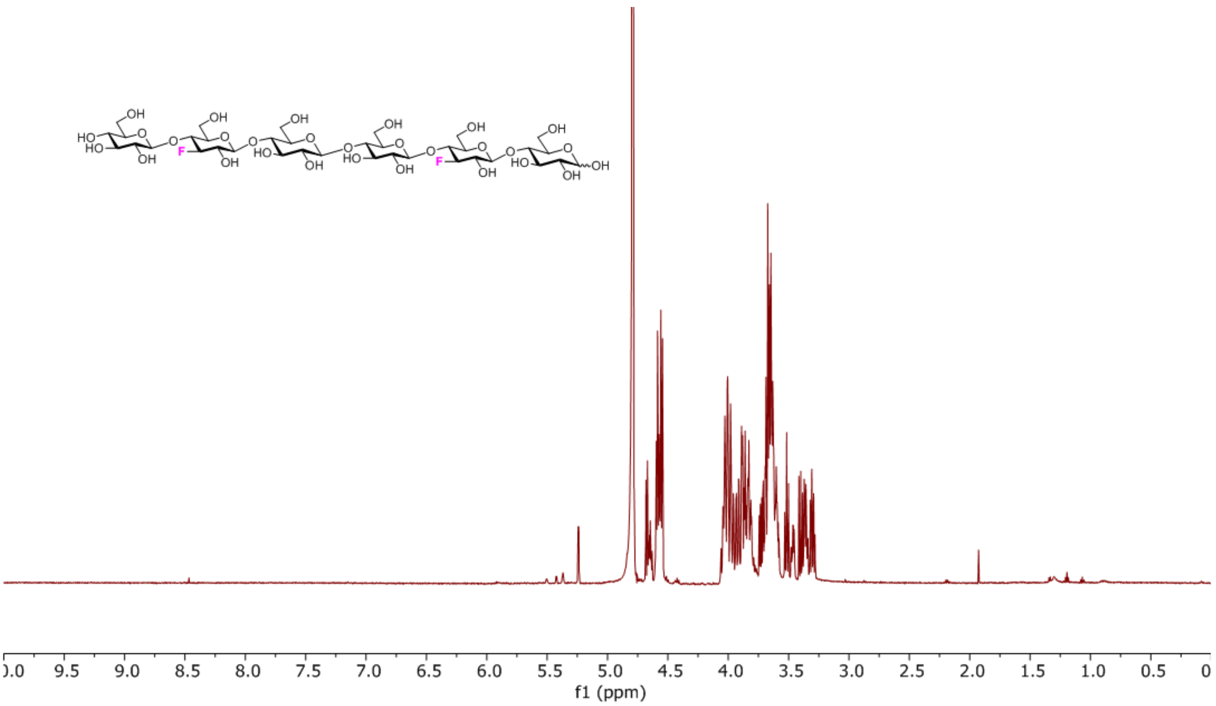

^{13}C NMR of AFAAFA-OH (151 MHz, D_2O)

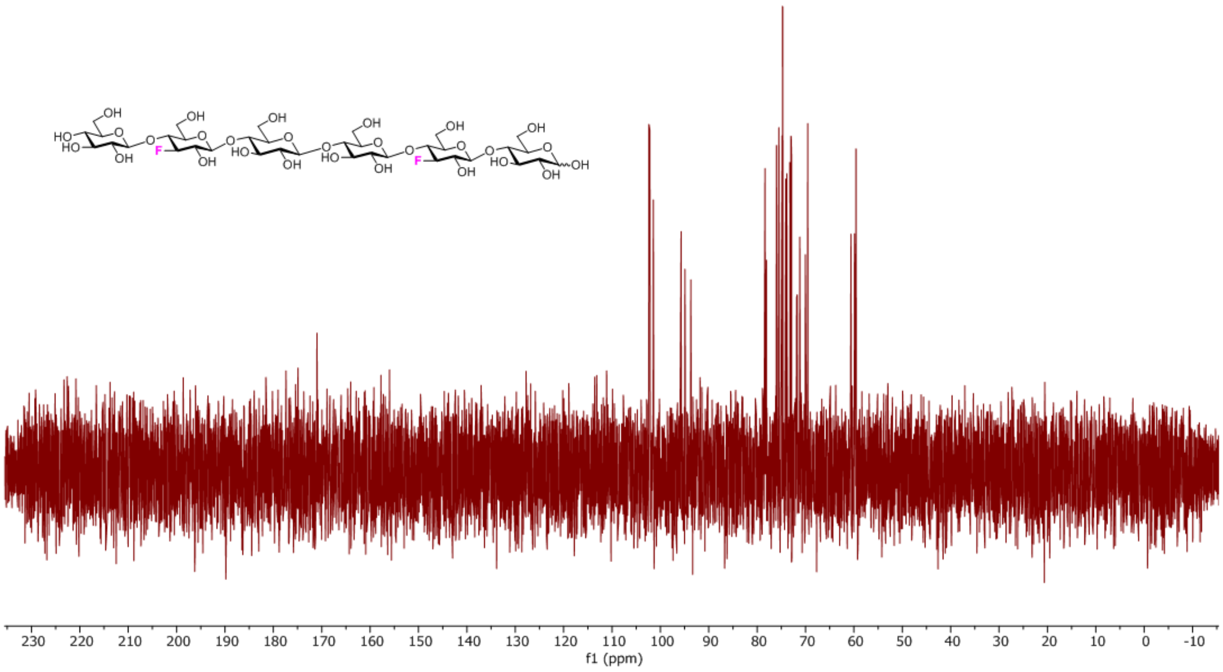

^{13}F NMR of AFAAFA-OH (564 MHz, D_2O)

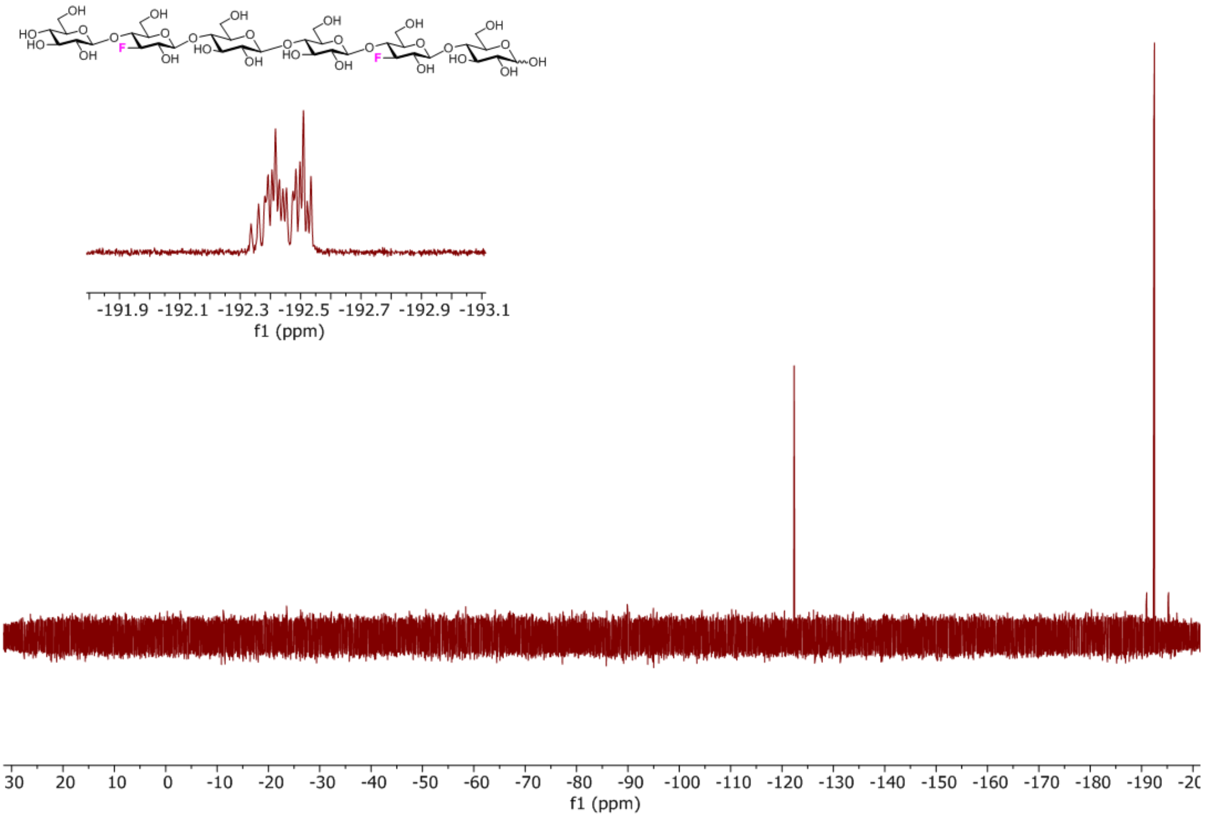

HSQC NMR of AFAAFA-OH (D_2O)

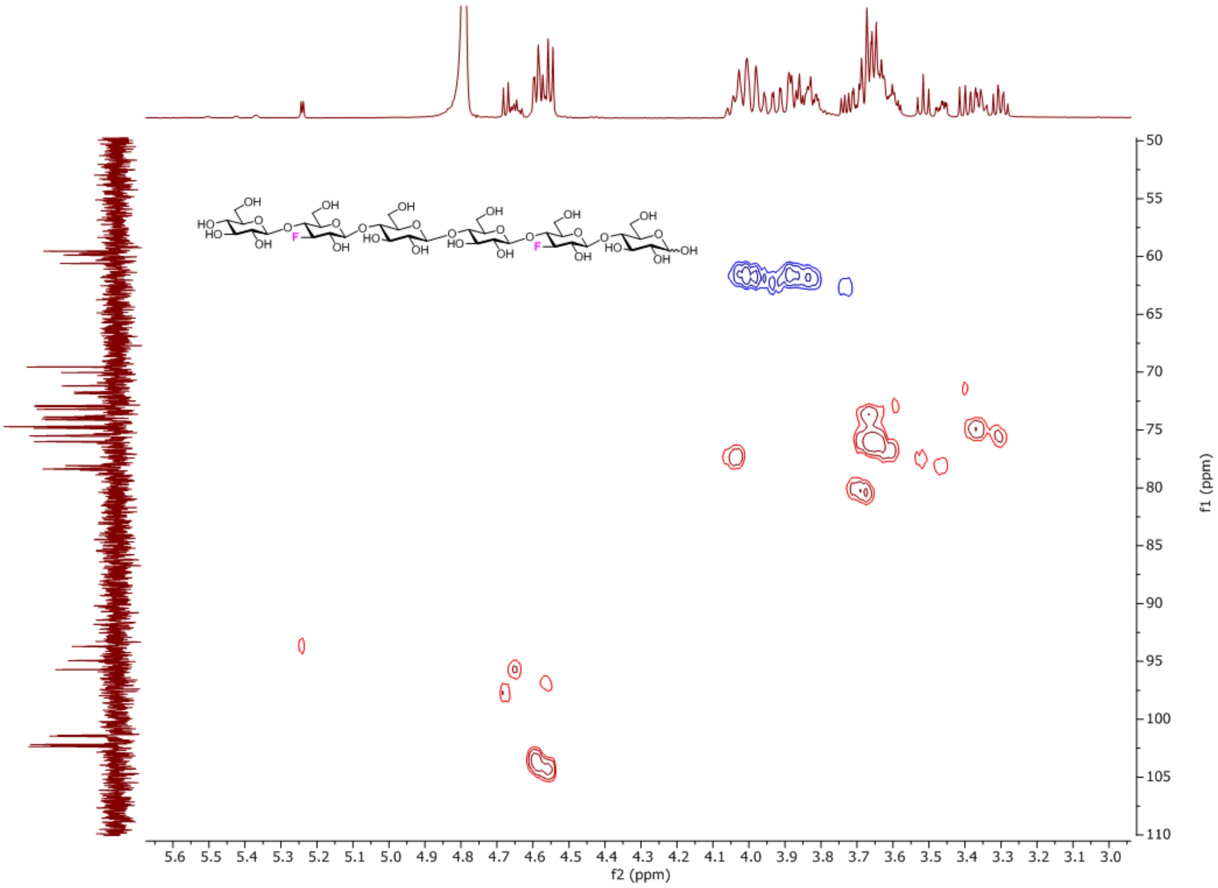

3.5.1.9. *Synthesis of FAFAFA-OH*

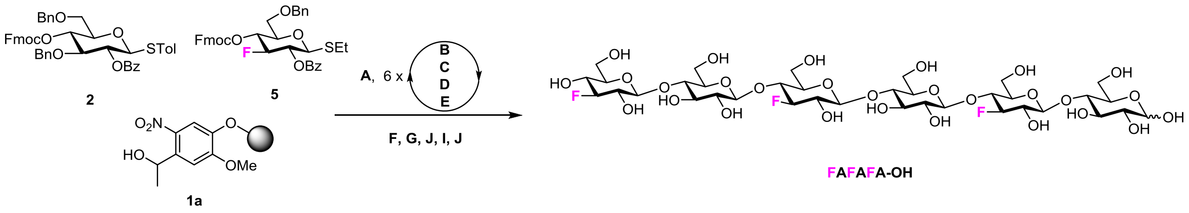

	Module	Conditions
	**A**: Resin Preparation for Synthesis	
3	**B**: Acidic Wash with TMSOTf Solution	
	**C**: Thioglycoside Glycosylation	**2**, 6.8 equiv.(-20° for 5 min, 0° for 20 min)
	**D**: Capping	
	**E**: Fmoc Deprotection	
	**B**: Acidic Wash with TMSOTf Solution	
	**C**: Thioglycoside Glycosylation	**5**, 6 equiv.(-20° for 5 min, 0° for 20 min)
	**D**: Capping	
	**E**: Fmoc Deprotection	
	**F**: On-resin Methanolysis	
	**G**: Cleavage from Solid Support	
	**J**: Purification	**Method B1:** t_R = 35.6 min
	**I**: Hydrogenolysis	
	**J**: Purification	**Method D**, t_R = 17.5 and 17.7 min

Automated synthesis, global deprotection, and purification afforded **FAFAFA-OH** as white solid (3.1 mg, 24% overall yield).

Analytical data for **FAFAFA-OH**: ^{1}H NMR (600 MHz, D_2O) δ 5.24 (d, *J* = 3.8 Hz, 0.3H, β-H1), 4.70 – 4.54 (m, 7.7H, α-H1, H3, H3), 4.47 (dt, *J* = 52.8, 8.9 Hz, 1H, H3), 4.09 – 3.72 (m, 17H), 3.72 – 3.56 (m, 13H), 3.55 – 3.49 (m, 1H), 3.40 – 3.27 (m, 2H); ^{13}C NMR (151 MHz, D_2O) δ 102.17, 101.62, 101.54, 101.45, 101.37, 96.08 (d, *J* = 181.4 Hz, C3), 95.70 (β-C1), 94.31 (d, *J* = 183.5 Hz, C3, C3), 91.75 (α-C1), 78.46, 78.31, 78.26, 75.43, 75.32, 74.80, 74.69, 74.57, 74.51, 74.11, 74.00, 73.98, 73.84, 73.76, 73.71, 72.98, 71.85, 71.72, 71.60, 71.17, 70.03, 67.72, 67.60, 60.10, 59.87, 59.76, 59.53; ^{19}F NMR (564 MHz, D_2O) δ -192.22 – -192.59 (m), -195.24 (dt, *J* = 52.8, 13.9 Hz); $[\alpha]_D^{20}$ +7.48 (c 0.2, H_2O); IR (neat) ν_{max} = 3357, 2924, 1595, 1029 cm^{-1}; (ESI-HRMS+) m/z 1019.304 $[M+Na]^+$ ($C_{36}H_{59}F_3O_{28}Na$ requires 1019.304).

RP-HPLC of FAFAFA-OH (ELSD trace, Method D, t_R = 17.5 and 17.7 min)

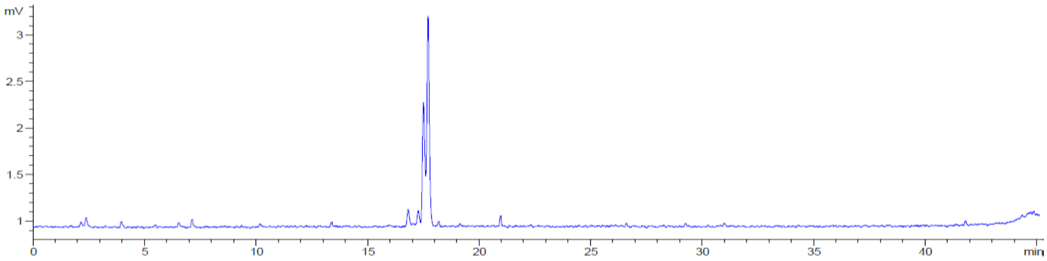

^{1}H NMR of FAFAFA-OH (600 MHz, D_2O)

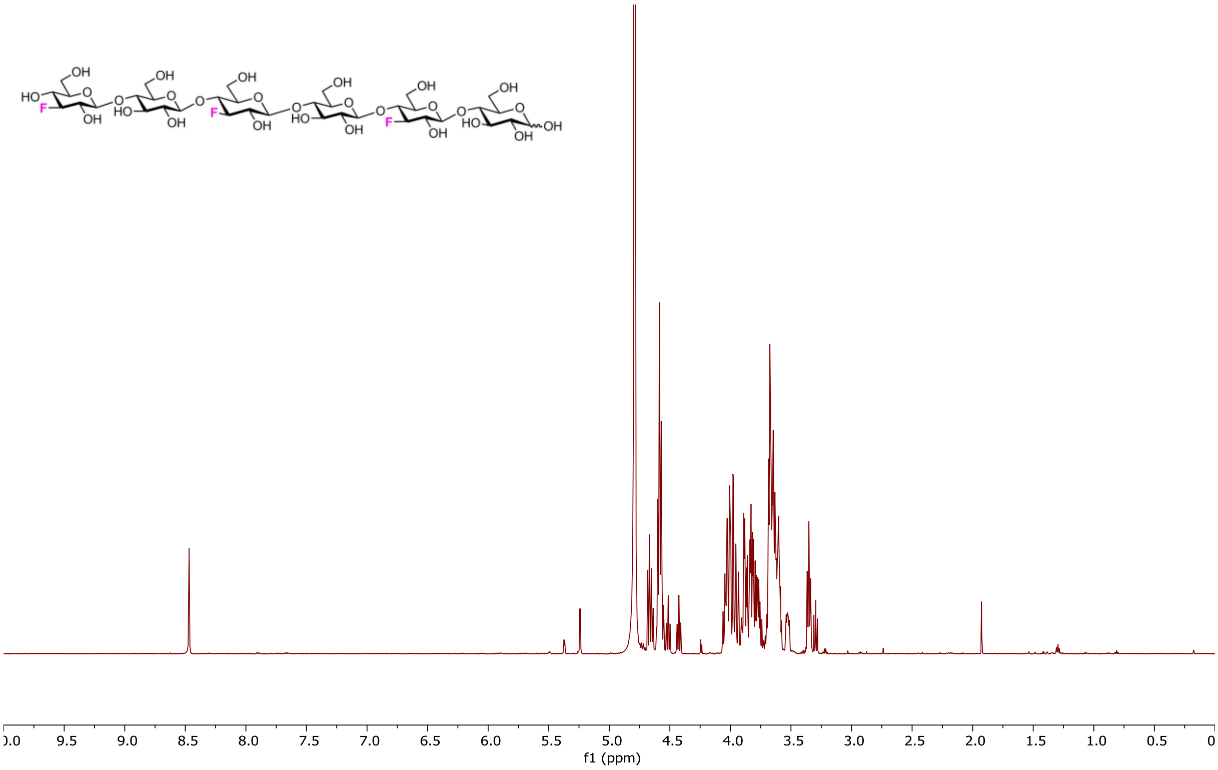

^{13}C NMR of FAFAFA-OH (151 MHz, D_2O)

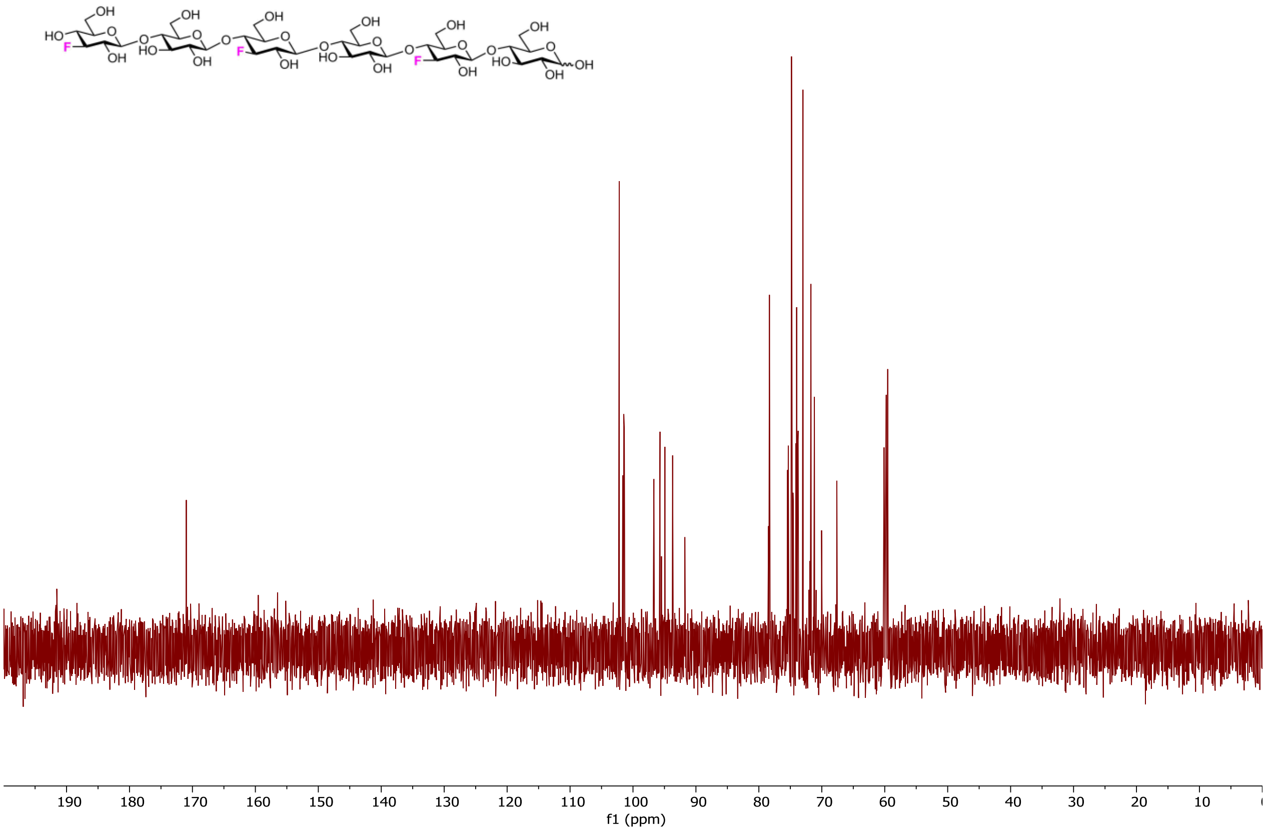

^{13}F NMR of FAFAFA-OH (564 MHz, D_2O)

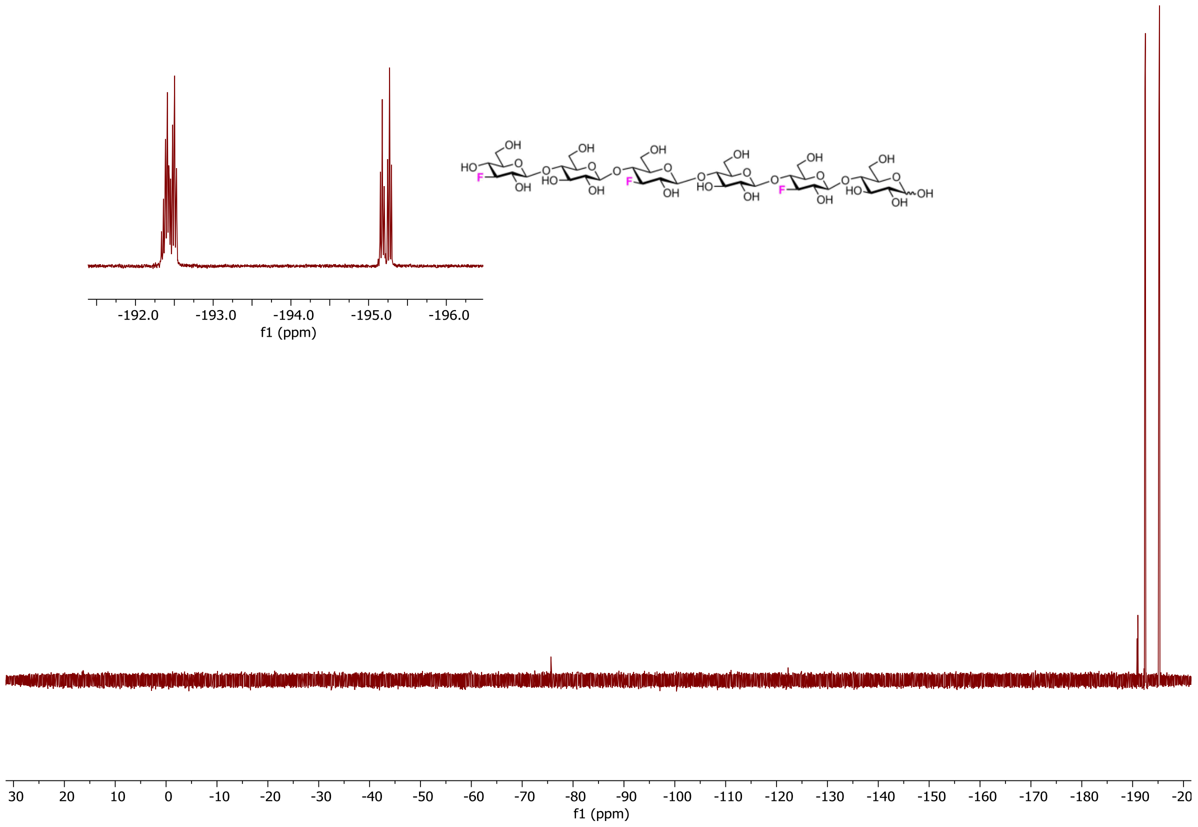

HSQC NMR of FAFAFA-OH (D_2O)

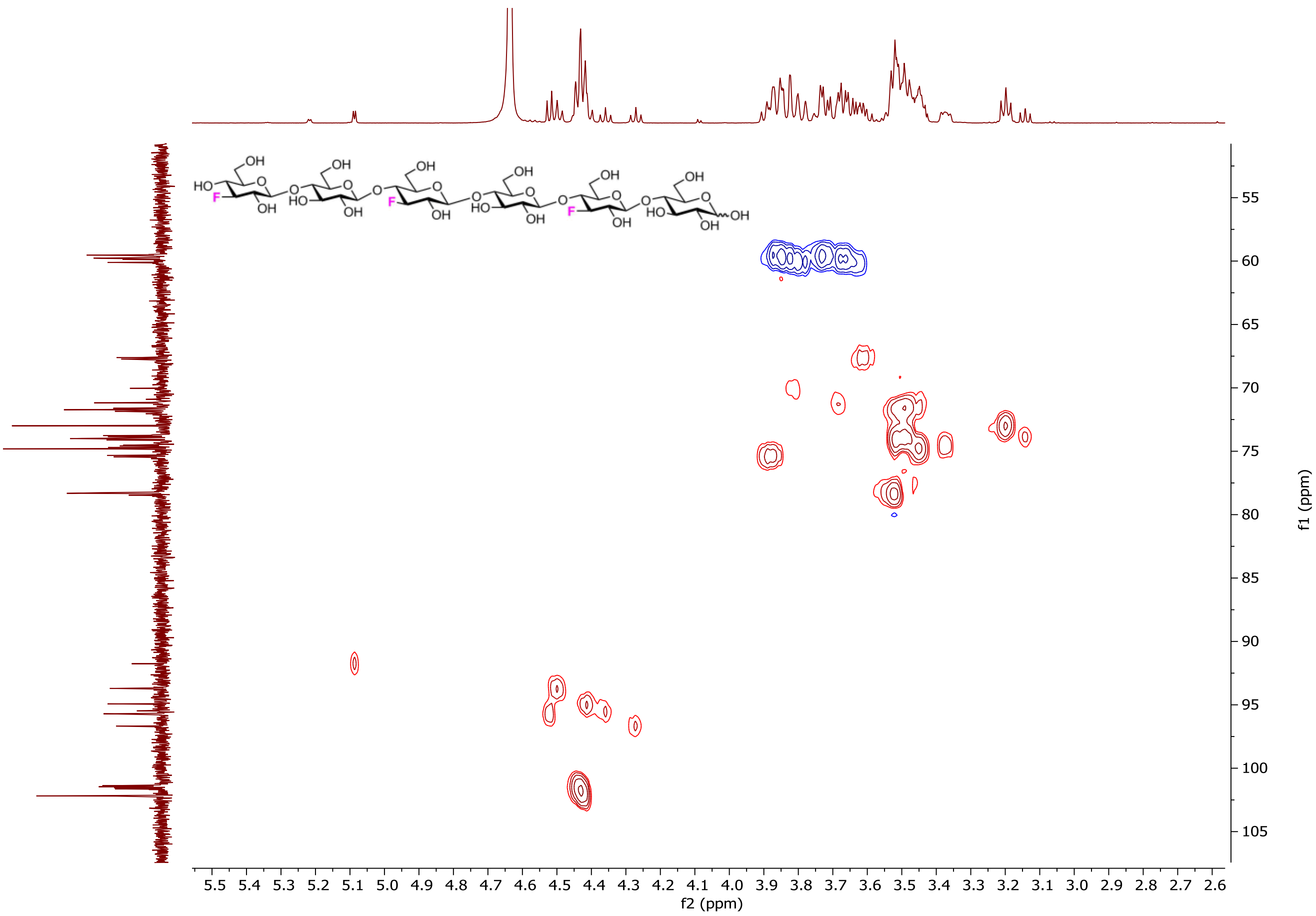

3.5.1.10. *Synthesis of ADAADA-OH*

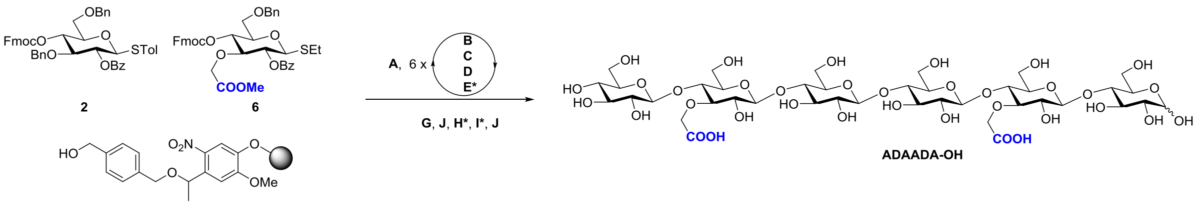

	Module	Conditions
	**A**: Resin Preparation for Synthesis	
2	**B**: Acidic Wash with TMSOTf Solution	
	**C**: Thioglycoside Glycosylation	**2**, 6.5 equiv.(-20°C for 5 min, 0°C for 20 min)
	**D**: Capping	
	**E***: Fmoc Deprotection (TEA)	
	**B**: Acidic Wash with TMSOTf Solution	
	**C**: Thioglycoside Glycosylation	**6**, 6.5 equiv.(-20°C for 5 min, 0°C for 20 min)
	**D**: Capping	
	**E***: Fmoc Deprotection (TEA)	
	**B**: Acidic Wash with TMSOTf Solution	

**C**: Thioglycoside Glycosylation	**2**, 6.5 equiv.(-20°C for 5 min, 0°C for 20 min)
**D**: Capping	
**E***: Fmoc Deprotection (TEA)	
**G**: Cleavage from Solid Support	
**J**: Purification	**Method N and Method B**
**H***: Solution-phase Methanolysis	
**I***: Hydrogenolysis with balloon	
**J**: Purification	**Method D**

Automated synthesis, global deprotection, and purification afforded **ADAADA-OH** as white solid (3.5 mg, 25 % overall yield).

Analytical data for **ADAADA-OH**: ^{1}H NMR (600 MHz, D_2O) δ 5.24 (d, *J* = 3.8 Hz, 0.29 H, α-H1), 4.68 (d, *J* = 8.0 Hz, 0.71H, β-H1), 4.62 – 4.46 (m, 7H), 4.42 – 4.35 (m, 2H), 4.05 – 3.94 (m, 5H), 3.94 – 3.77 (m, 9H), 3.77 – 3.53 (m, 13H), 3.53 – 3.45 (m, 3H), 3.45 – 3.35 (m, 4H), 3.34 – 3.20 (m, 2H); ^{13}C NMR (151 MHz, D_2O) δ 175.93, 166.68, 102.33, 102.28, 102.24, 102.16, 95.68 (β-C1), 91.75 (α-C1), 82.94, 78.48, 78.43, 78.31, 78.08, 76.18, 76.01, 75.49, 74.90, 74.89, 74.84, 74.71, 74.15, 74.01, 73.89, 73.81, 73.58, 73.32, 72.87, 72.42, 71.21, 71.15, 70.03, 69.97, 69.48, 60.81, 60.07, 59.90, 59.75, 59.68; $[\alpha]_D^{20}$ +15.72 (c 0.35, H_2O); IR (neat) ν_{max} = 3375, 2929, 1722, 1069 cm^{-1}; m/z ($HRMS^+$) 1129.341 $[M + Na]^+$ ($C_{40}H_{66}O_{35}Na^+$ requires 1129.327).

RP-HPLC of ADAADA-OH (ELSD trace, Method C, t_R = 16.8 min)

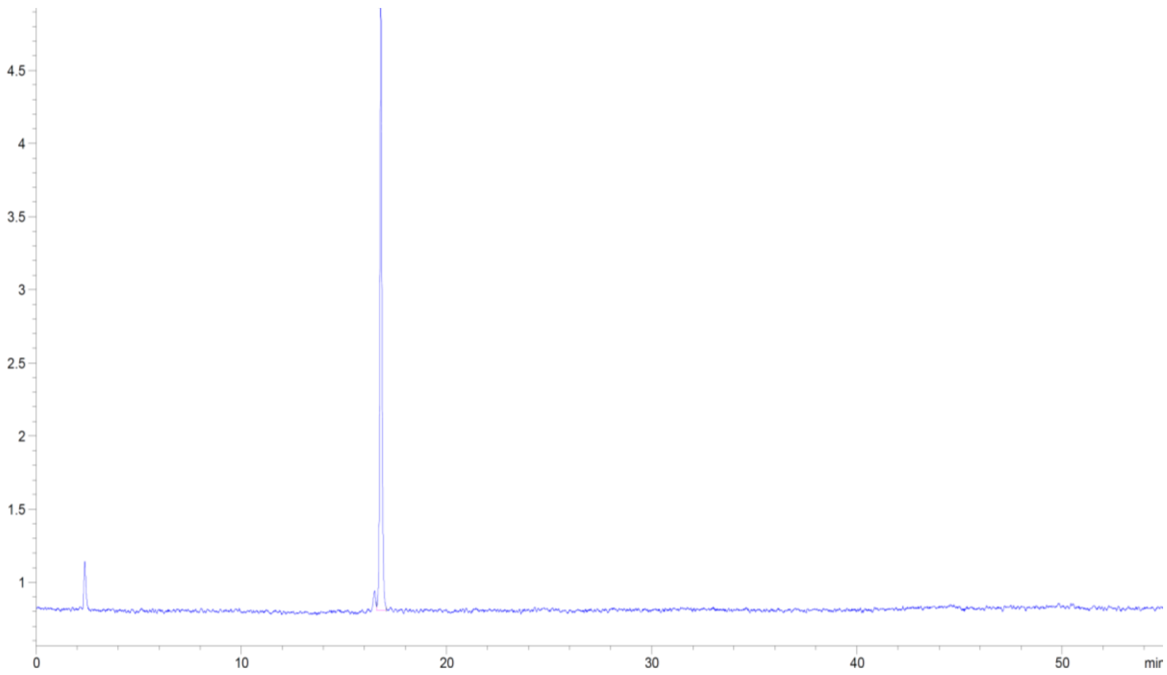

^{1}H NMR of ADAADA-OH (600 MHz, D_2O)

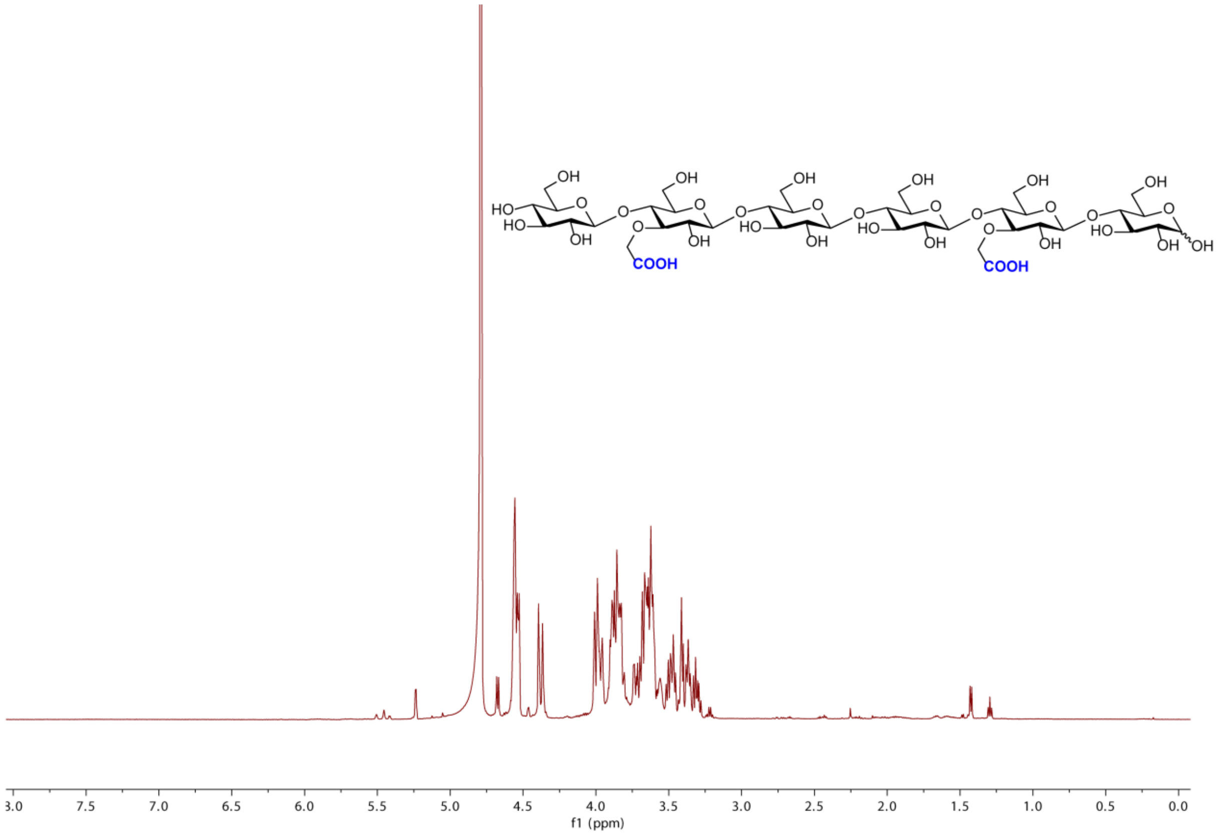

^{13}C NMR of ADAADA-OH (151 MHz, D_2O)

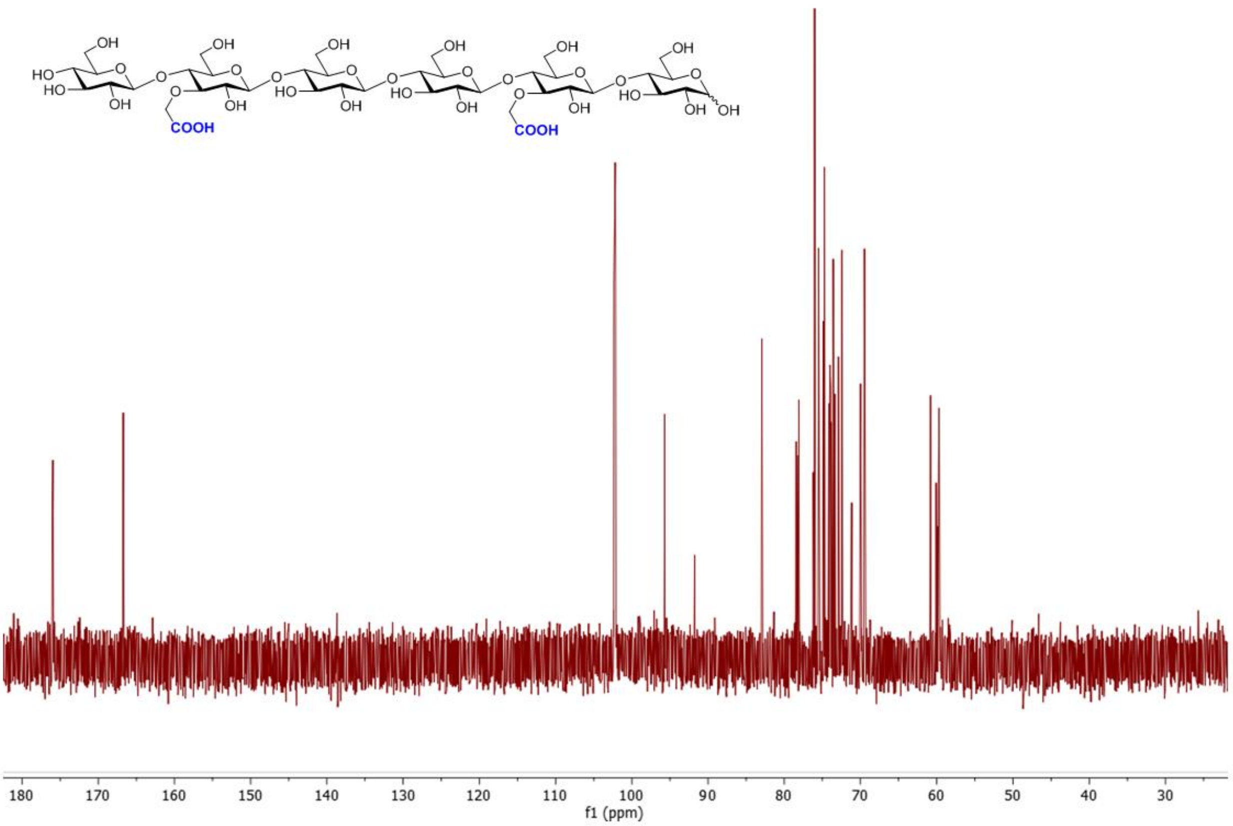

HSQC NMR of ADAADA-OH (D_2O)

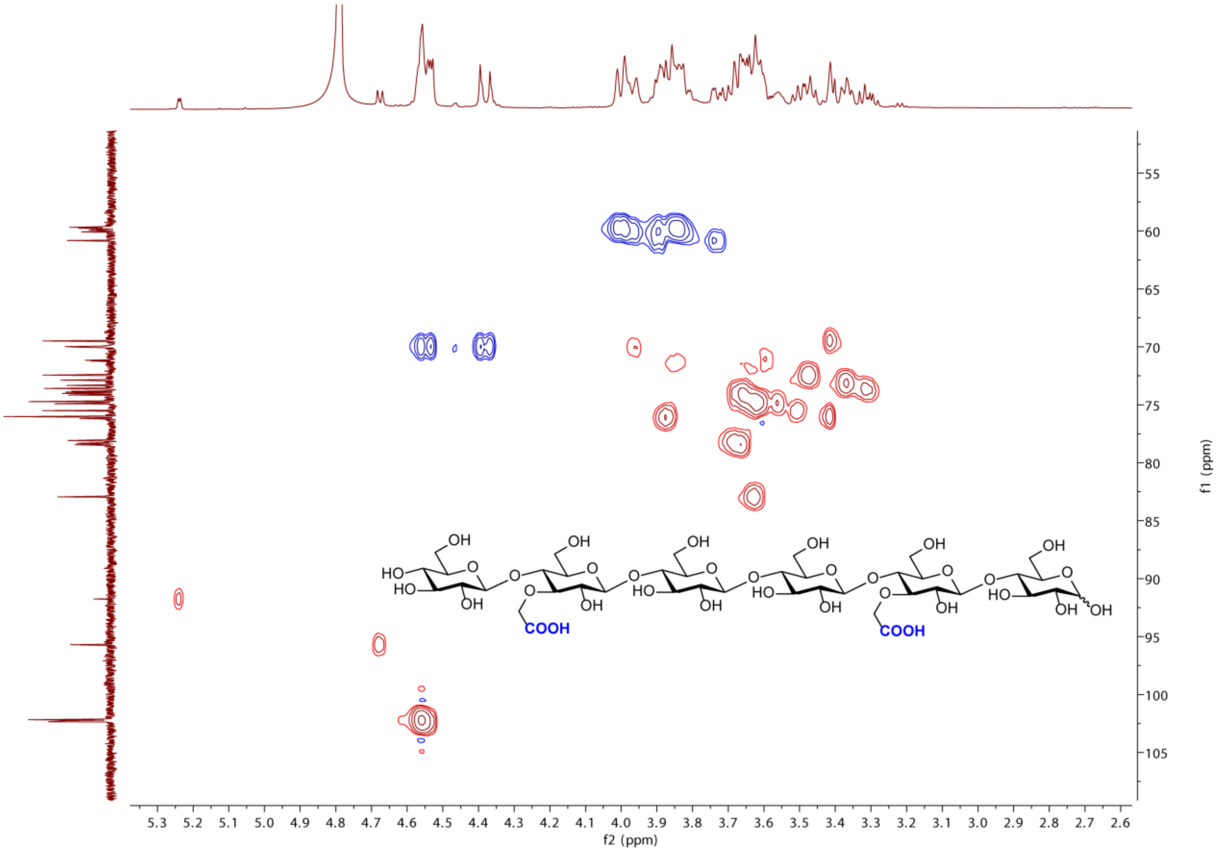

3.5.1.11. *Synthesis of DADADA-OH*

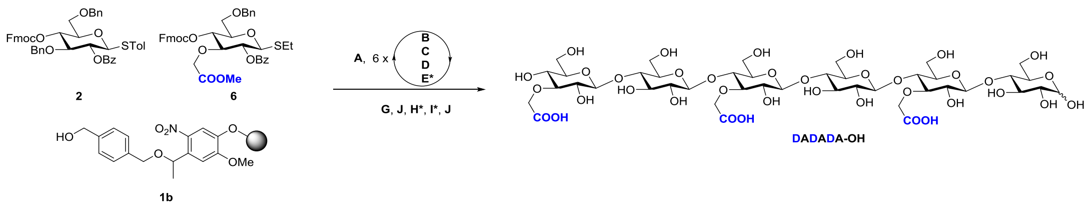

Module	Conditions
**A**: Resin Preparation for Synthesis	
**B**: Acidic Wash with TMSOTf Solution	
**C**: Thioglycoside Glycosylation	**2**, 6.5 equiv.(-20°C for 5 min, 0°C for 20 min)
**D**: Capping	
**E***: Fmoc Deprotection (TEA)	
**3** × **B**: Acidic Wash with TMSOTf Solution	
**C**: Thioglycoside Glycosylation	**2a**, 6.5 equiv.(-20°C for 5 min, 0°C for 20 min)
**D**: Capping	
**E***: Fmoc Deprotection (TEA)	
**G**: Cleavage from Solid Support	
**J**: Purification	**Method N and Method B**
**H***: Solution-phase Methanolysis	
**I***: Hydrogenolysis with balloon	
**J**: Purification	**Method D**

Automated synthesis, global deprotection, and purification afforded **DADADA-OH** as white solid (3.4 mg, 24 % overall yield).

Analytical data for **DADADA-OH**: ^{1}H NMR (600 MHz, D_2O) δ 5.24 (d, *J* = 3.8 Hz, 0.35 H, α-H1), 4.68 (d, *J* = 8.0 Hz, 0.65H, β-H1), 4.66 – 4.48 (m, 7H), 4.48 – 4.37 (m, 4H), 4.04 – 3.80 (m, 14H), 3.80 – 3.71 (m, 2H), 3.70 – 3.59 (m, 9H), 3.59 – 3.53 (m, 3H), 3.53 – 3.44 (m, 5H), 3.36 (ddd, *J* = 10.1, 7.4, 5.0 Hz, 2H), 3.32 – 3.20 (m, 1H); ^{13}C NMR (151 MHz, D_2O) δ 175.75, 175.24, 175.21, 175.17, 166.06, 102.28, 102.25, 102.24, 102.15, 102.12, 95.68 (β-C1), 91.74 (α-C1), 85.22, 82.89, 82.87, 78.50, 78.44, 78.35, 76.13, 75.61, 74.84, 74.71, 74.15, 74.01, 73.98, 73.81, 73.31, 72.74, 72.46, 71.21, 71.15, 70.03, 69.82, 69.63, 68.89, 60.36, 60.08,60.05, 59.92, 59.79, 59.67; $[\alpha]_D^{20}$ +20.51 (c 0.2, H_2O); IR (neat) ν_{max} = 3361, 2927, 1726, 1073 cm^{-1}; m/z (HRMS$^+$) 1187.332 $[M + Na]^+$ ($C_{42}H_{68}O_{37}Na^+$ requires 1187.333).

RP-HPLC of DADADA-OH (ELSD trace, Method C, t_R = 18.1, 18.3 min)

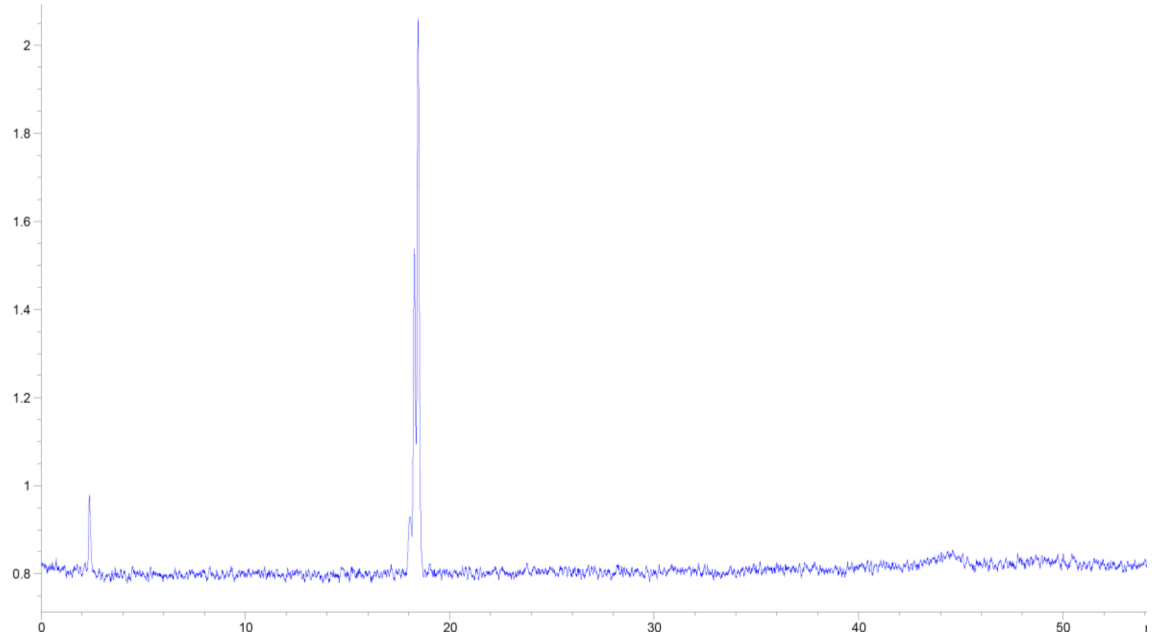

^{1}H NMR of DADADA-OH (600 MHz, D_2O)

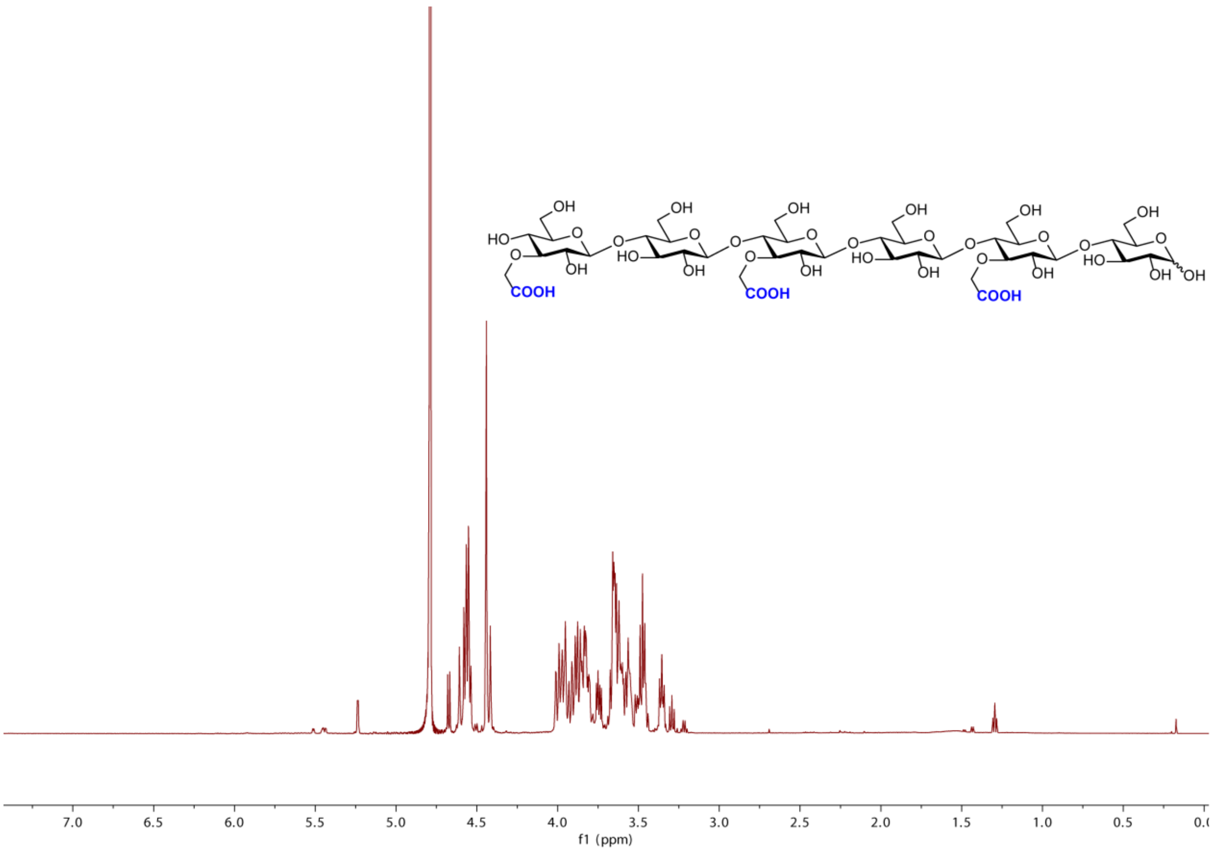

^{13}C NMR of DADADA-OH (151 MHz, D_2O)

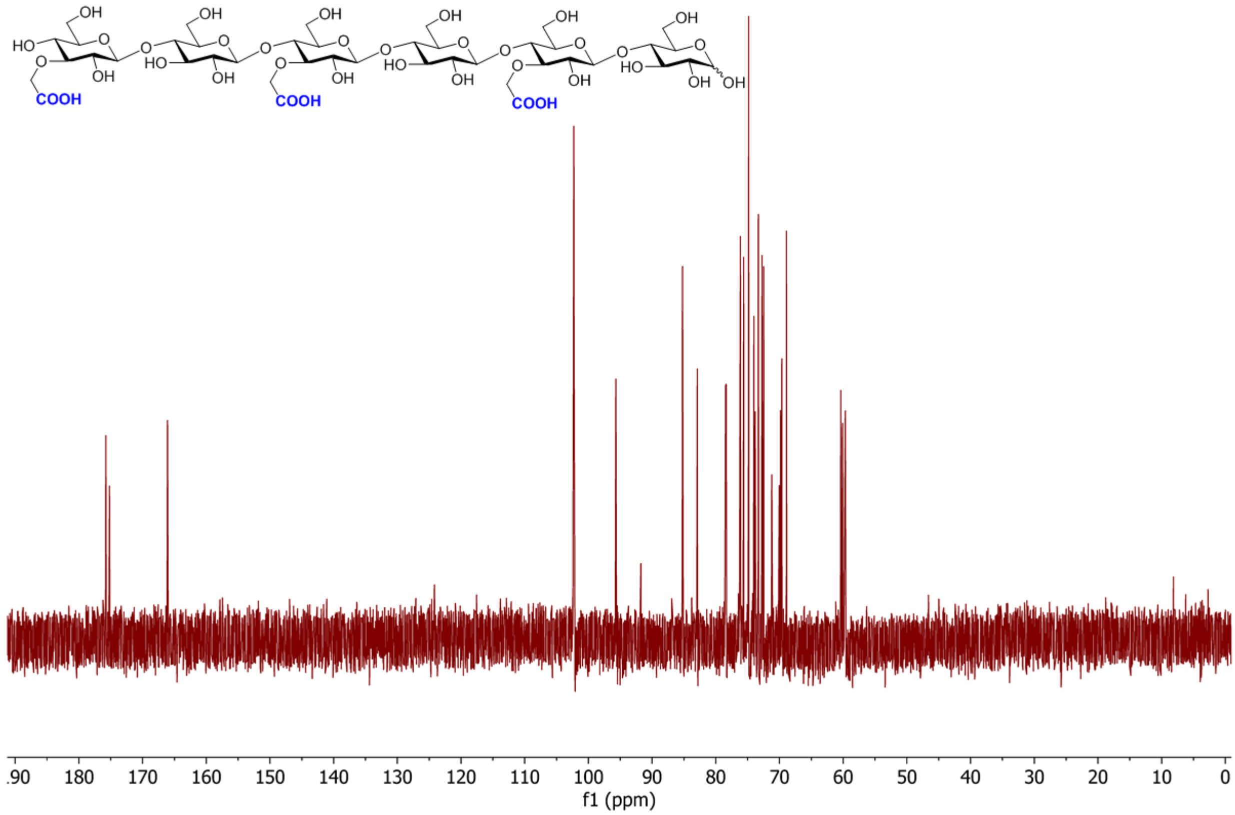

HSQC NMR of DADADA-OH (D_2O)

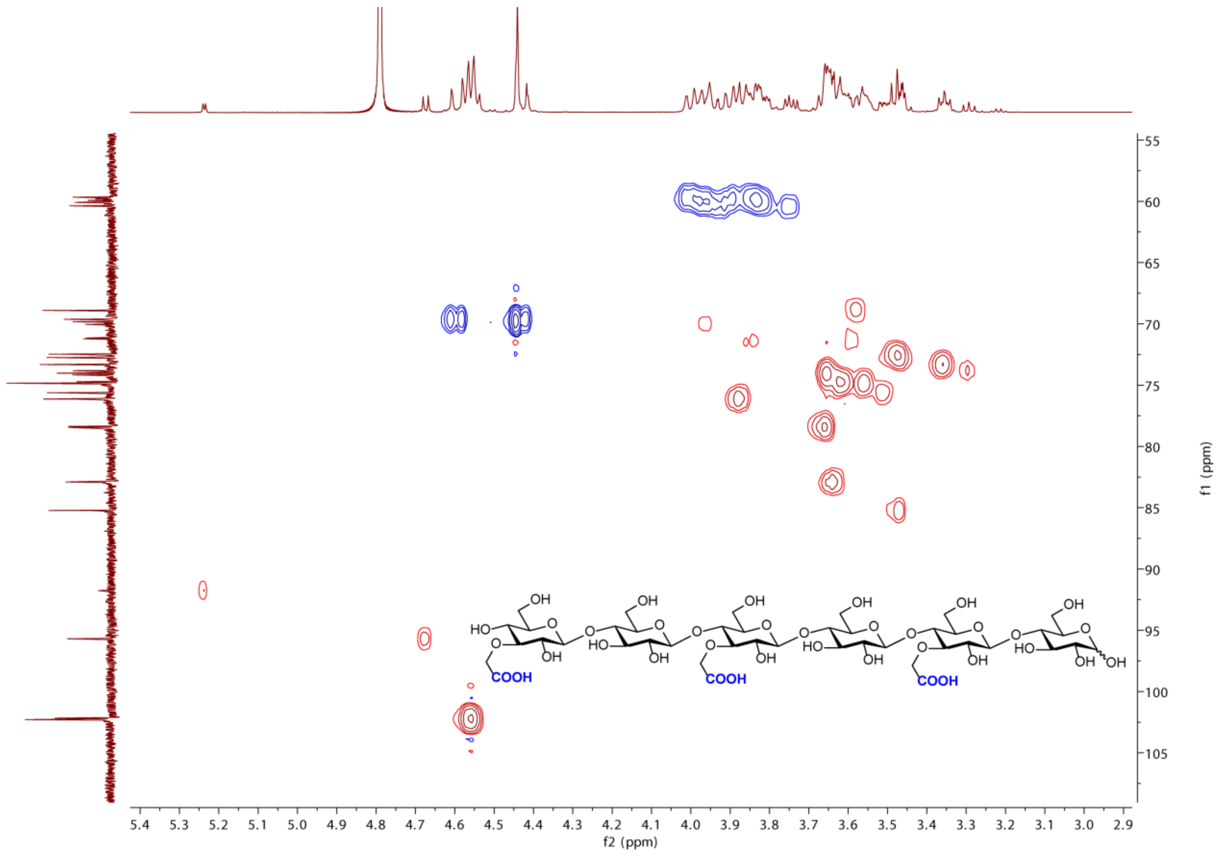

3.5.1.12. *Synthesis of AEAAEA-OH*

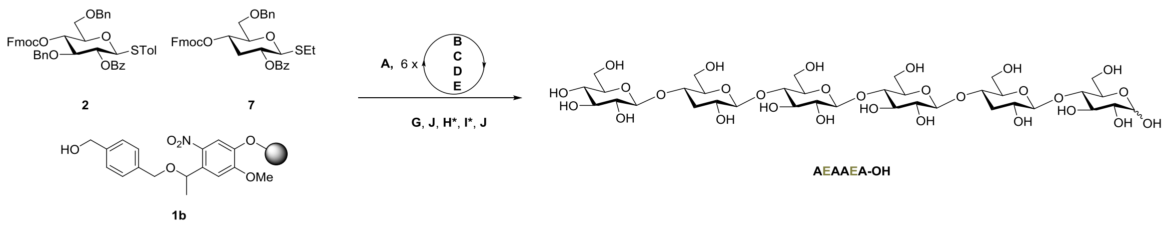

	Module	Conditions
	**A**: Resin Preparation for Synthesis	
2	**B**: Acidic Wash with TMSOTf Solution	
	**C**: Thioglycoside Glycosylation	**2**, 6.5 equiv.(-20°C for 5 min, 0°C for 20 min)
	**D**: Capping	
	**E**: Fmoc Deprotection	
	**B**: Acidic Wash with TMSOTf Solution	
	**C**: Thioglycoside Glycosylation	**7**, 6.5 equiv.(-20°C for 5 min, 0°C for 20 min)
	**D**: Capping	
	**E**: Fmoc Deprotection	
	**B**: Acidic Wash with TMSOTf Solution	
	**C**: Thioglycoside Glycosylation	**2**, 6.5 equiv.(-20°C for 5 min, 0°C for 20 min)
	**C**: Thioglycoside Glycosylation	**2**, 6.5 equiv.(-20°C for 5 min, 0°C for 20 min)
	**D**: Capping	
	**E**: Fmoc Deprotection	
	**G**: Cleavage from Solid Support	
	**J**: Purification	**Method N and Method B**
	**H**: Solution-phase Methanolysis	
	**I***: Hydrogenolysis with balloon	
	**J**: Purification	**Method D**

Automated synthesis, global deprotection, and purification afforded **AEAAEA-OH** as white solid (1.8 mg, 15 % overall yield).

Analytical data for **AEAAEA-OH**: ^{1}H NMR (600 MHz, D_2O) δ 5.24 (d, *J* = 3.8 Hz, 0.35 H, α-H1), 4.67 (d, *J* = 7.8 Hz, 0.65H, β-H1), 4.59– 4.51 (m, 3H), 4.50 – 4.44 (m, 2H), 4.02 – 3.89 (m, 6H), 3.89 – 3.70 (m, 10H), 3.70 – 3.57 (m, 9H), 3.57 – 3.50 (m, *J* = 18.1, 10.5, 7.4 Hz, 2H), 3.50 – 3.44 (m, 2H), 3.42 – 3.34 (m, 2H), 3.33 – 3.19 (m, 3H), 2.61 (dq, *J* = 15.6, 9.8, 7.4 Hz, 2H), 1.70 (tt, *J* = 11.7, 6.6 Hz, 2H); ^{13}C NMR (151 MHz, D_2O) δ 104.35, 103.14, 102.93, 102.24, 95.67 (β-C1), 91.75 (α-C1), 78.35, 78.31, 78.05, 75.81, 75.53, 74.74, 74.64, 74.20, 74.05, 73.95, 73.83, 73.56, 73.10, 72.87, 71.23, 71.16, 70.04, 69.49, 67.52, 60.54, 60.20, 59.82, 46.61, 37.24; $[\alpha]_D^{20}$ +17.54 (c 0.2, H_2O); IR (neat) ν_{max} = 3359, 2929, 1033 cm^{-1}; m/z ($HRMS^+$) 981.3264 $[M + Na]^+$ ($C_{36}H_{62}O_{29}Na^+$ requires 981.3269).

RP-HPLC of AEAAEA-OH (ELSD trace, Method C, t_R = 16.2 min)

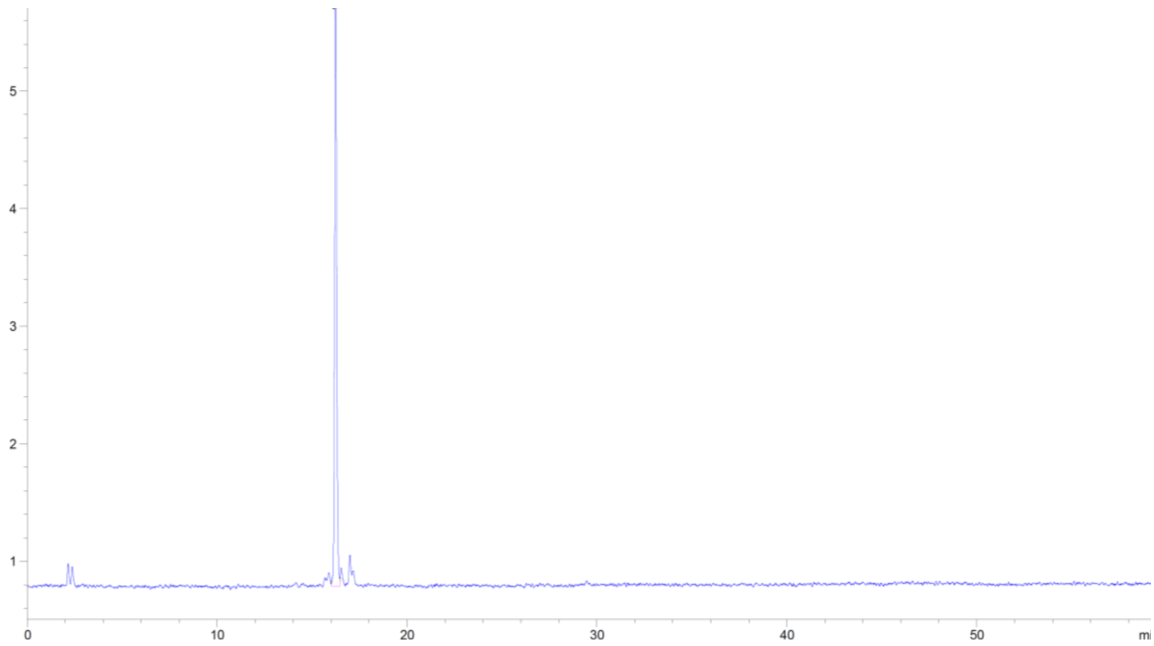

^{1}H NMR of AEAAEA-OH (600 MHz, D_2O)

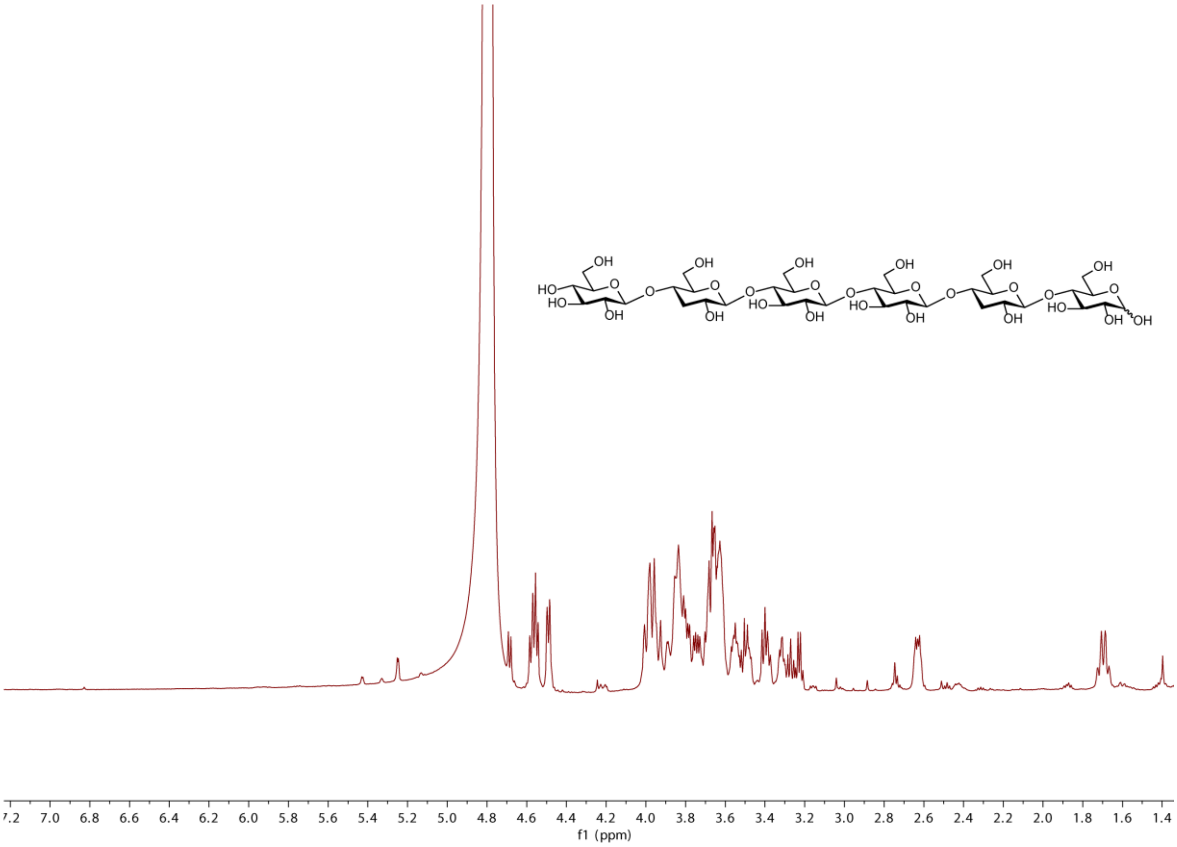

^{13}C NMR of AEAAEA-OH (151 MHz, D_2O)

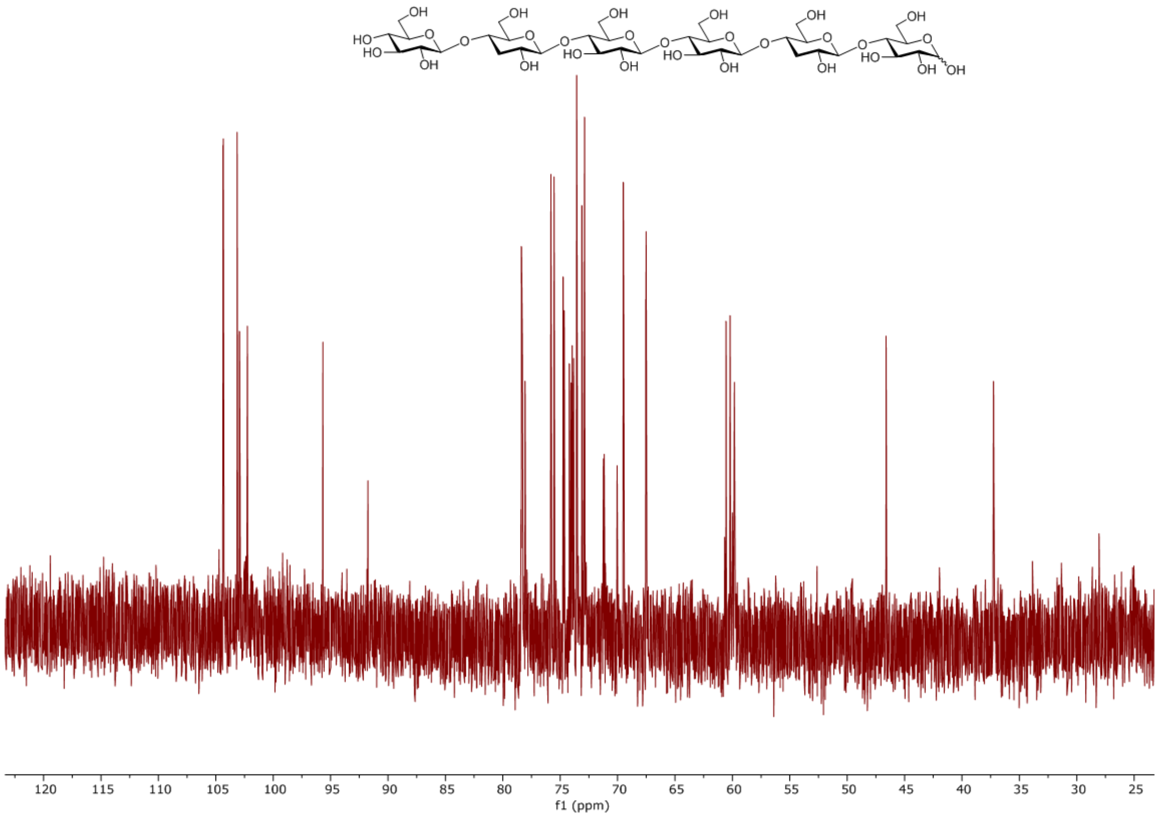

HSQC NMR of AEAAEA-OH (D_2O)

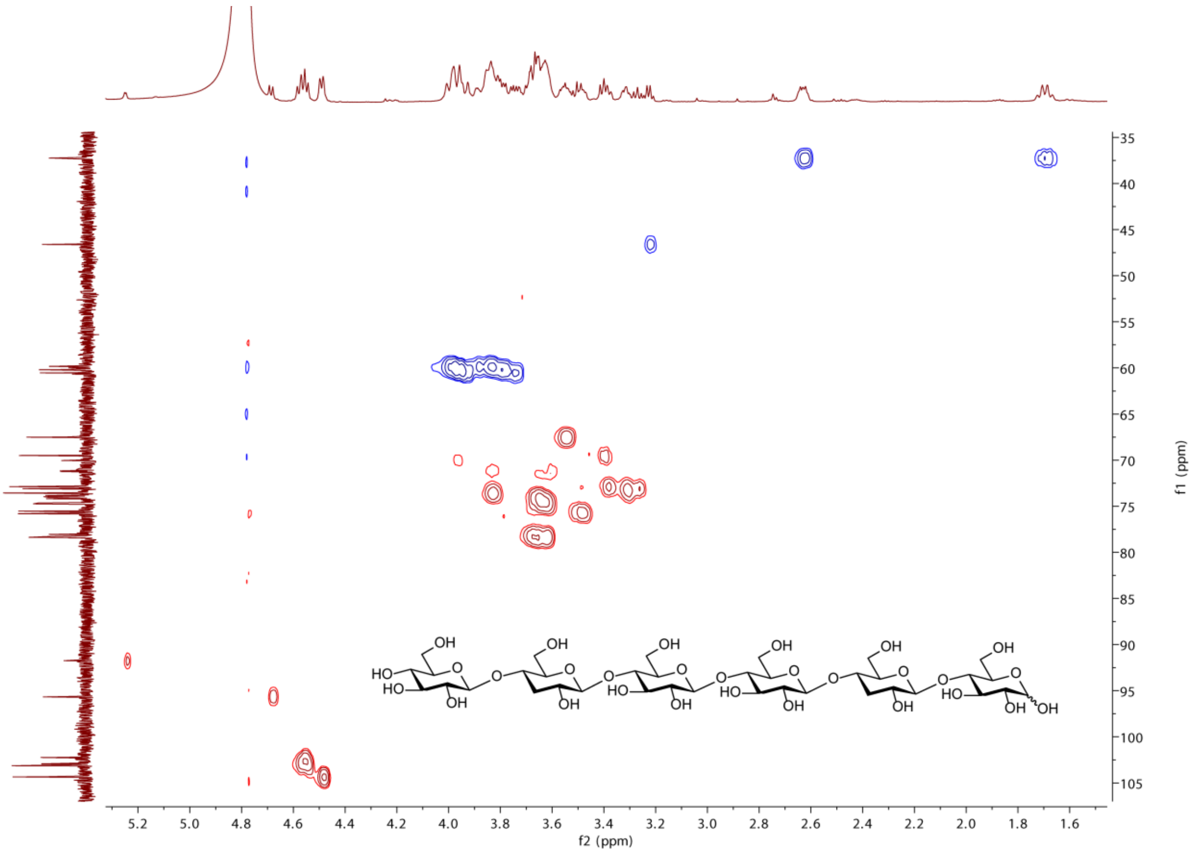

3.5.1.13. *Synthesis of AEAEAE-OH*

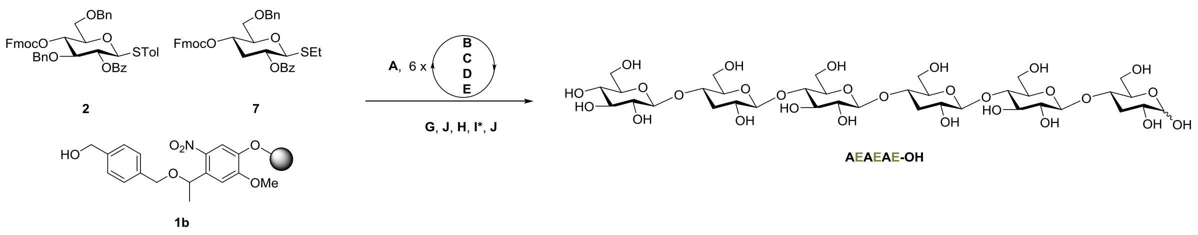

	Module	Conditions
	**A**: Resin Preparation for Synthesis	
	**B**: Acidic Wash with TMSOTf Solution	
	**C**: Thioglycoside Glycosylation	**7**, 6.5 equiv.(-20°C for 5 min, 0°C for 20 min)
	**C**: Thioglycoside Glycosylation	**7**, 6.5 equiv.(-20°C for 5 min, 0°C for 20 min)
	**D**: Capping	
	**D**: Capping	
	**E**: Fmoc Deprotection	
	**B**: Acidic Wash with TMSOTf Solution	
	**C**: Thioglycoside Glycosylation	**2**, 6.5 equiv.(-20°C for 5 min, 0°C for 20 min)
	**C**: Thioglycoside Glycosylation	**2**, 6.5 equiv.(-20°C for 5 min, 0°C for 20 min)
	**D**: Capping	
	**E**: Fmoc Deprotection	
**2**	**B**: Acidic Wash with TMSOTf Solution	
**2**	**C**: Thioglycoside Glycosylation	**7**, 6.5 equiv.(-20°C for 5 min, 0°C for 20 min)
**2**	**D**: Capping	
**2**	**E**: Fmoc Deprotection	
**2**	**B**: Acidic Wash with TMSOTf Solution	
**2**	**C**: Thioglycoside Glycosylation	**2**, 6.5 equiv.(-20°C for 5 min, 0°C for 20 min)
**2**	**C**: Thioglycoside Glycosylation	**2**, 6.5 equiv.(-20°C for 5 min, 0°C for 20 min)
**2**	**D**: Capping	
**2**	**E**: Fmoc Deprotection	
	**G**: Cleavage from Solid Support	
	**J**: Purification	**Method N and Method B**
	**H**: Solution-phase Methanolysis	
	**I***: Hydrogenolysis with balloon	
	**J**: Purification	**Method D**

Automated synthesis, global deprotection, and purification afforded **AEAEAE-OH** as white solid (1.3 mg, 11 % overall yield).

Analytical data for **AEAEAE-OH**: ^{1}H NMR (600 MHz, D_2O) δ 5.15 (d, *J* = 3.4 Hz, 0.29 H, α-H1), 4.60 (d, *J* = 8.1 Hz, 0.71H, β-H1), 4.58 (d, *J* = 8.3 Hz, 2H), 4.55 (d, *J* = 8.1 Hz, 1H), 4.48 (d, *J* = 7.9 Hz, 2H), 4.03 – 3.90 (m, 6H), 3.89 – 3.70 (m, 11H), 3.69 – 3.57 (m, 9H), 3.56 – 3.50 (m, 2H), 3.50 – 3.43 (m, 2H), 3.40 (t, *J* = 9.4 Hz, 1H), 3.32 (td, *J* = 8.6, 4.3 Hz, 1H), 3.27 (dd, *J* = 9.4, 8.0 Hz, 1H), 2.62 (ddt, *J* = 17.0, 9.2,

4.7 Hz, 3H), 1.70 (dt, *J* = 13.7, 10.3 Hz, 3H); ^{13}C NMR (151 MHz, D_2O) δ 104.34, 103.14, 102.92, 102.90, 97.67 (β-C1), 90.75 (α-C1), 78.36, 78.34, 78.25, 78.21, 75.81, 75.53, 74.66, 74.07, 73.83, 73.55, 73.10, 72.90, 69.50, 67.53, 60.55, 60.37, 60.18, 59.87, 37.41, 37.29, 37.24; $[\alpha]_D^{20}$ +7.58 (c 0.1, H_2O); IR (neat) ν_{max} = 3340, 2927, 1596, 1041 cm^{-1}; m/z ($HRMS^+$) 965.3326 $[M + Na]^+$ ($C_{36}H_{62}O_{28}Na^+$ requires 965.3320).

RP-HPLC of AEAEAE-OH (ELSD trace, Method C, t_R = 16.7, 17.3 min)

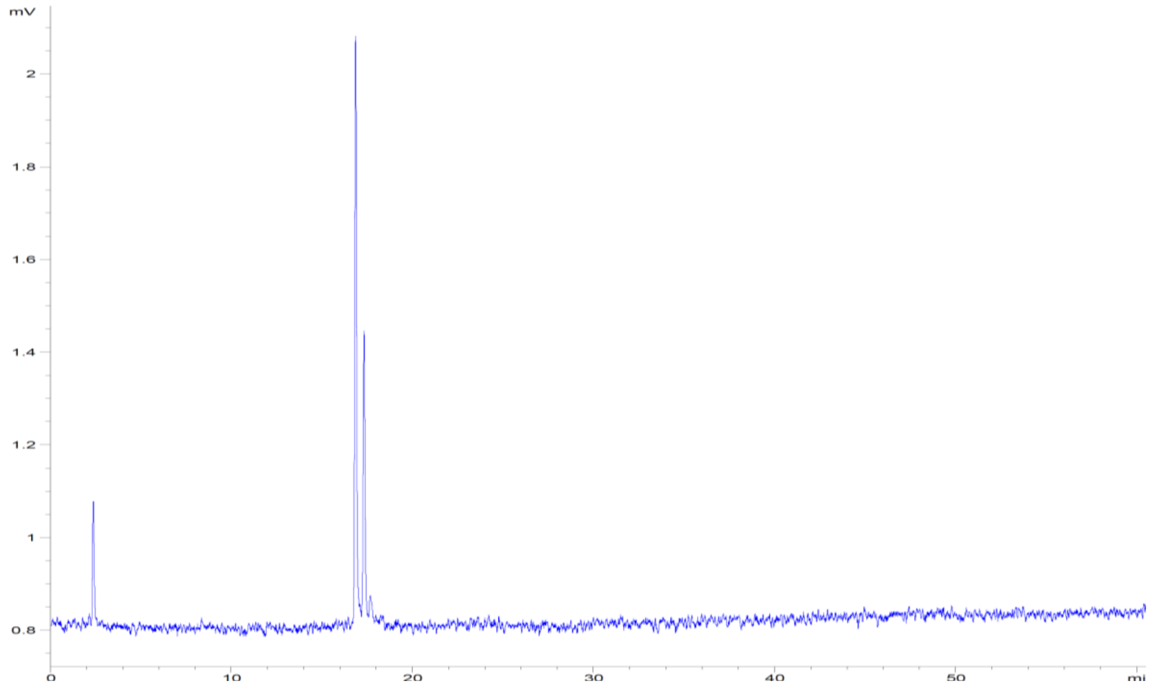

^1H NMR of AEAEAE-OH (600 MHz, D_2O)

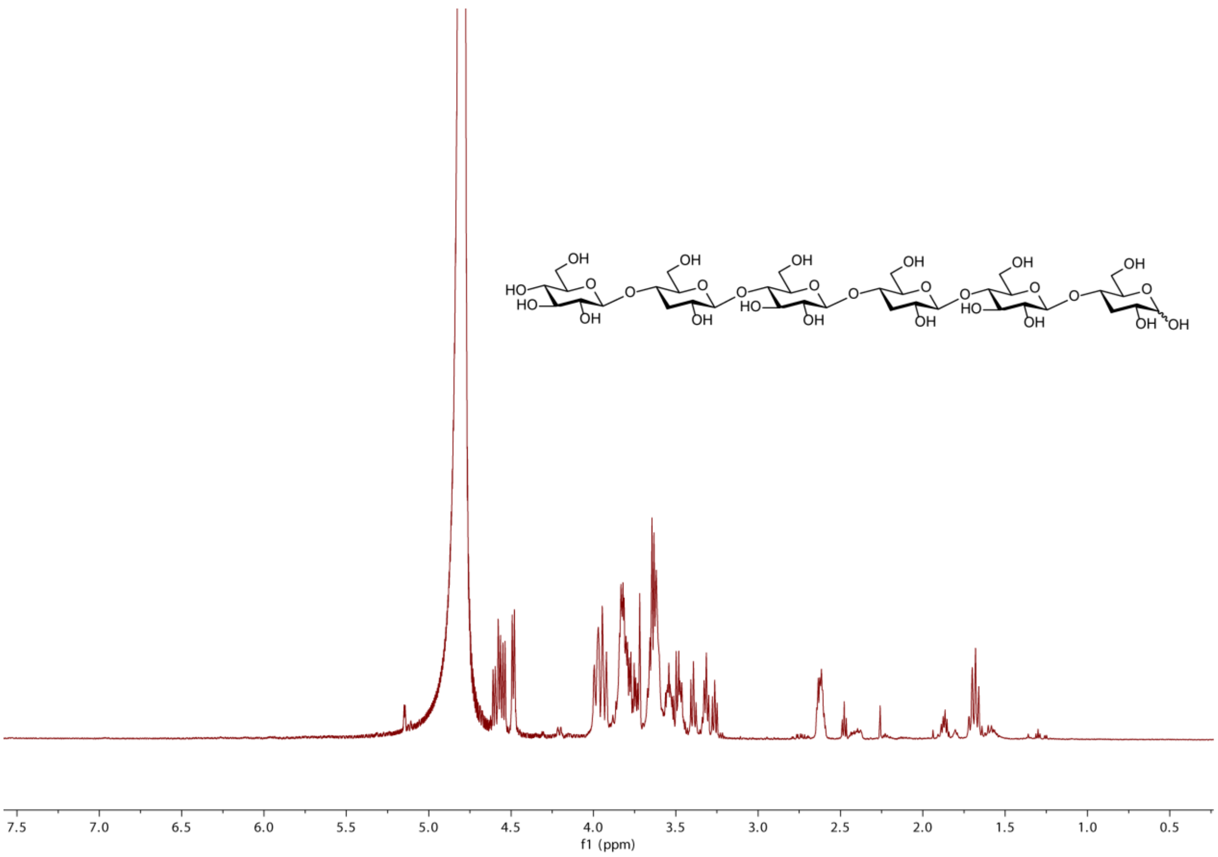

^{13}C NMR of AEAEAE-OH (151 MHz, D_2O)

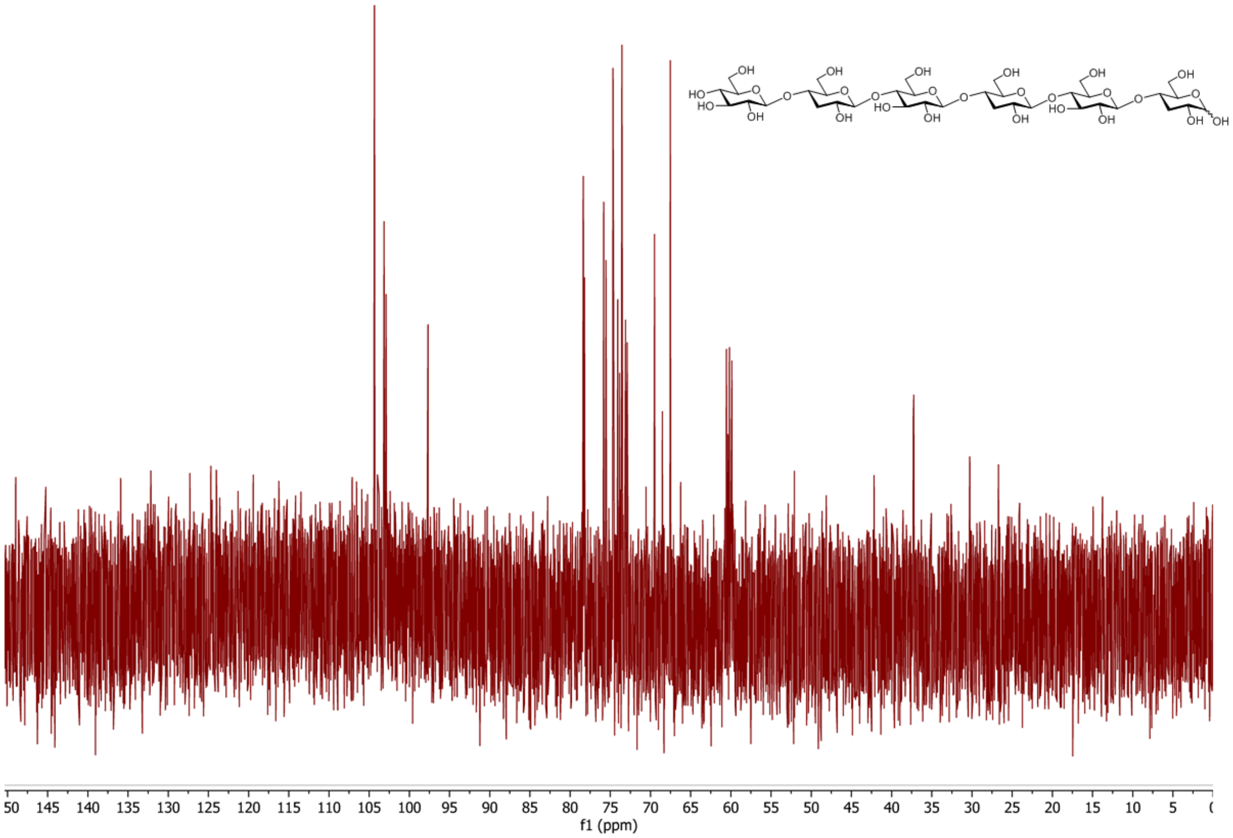

HSQC NMR of AEAEAE-OH (D_2O)

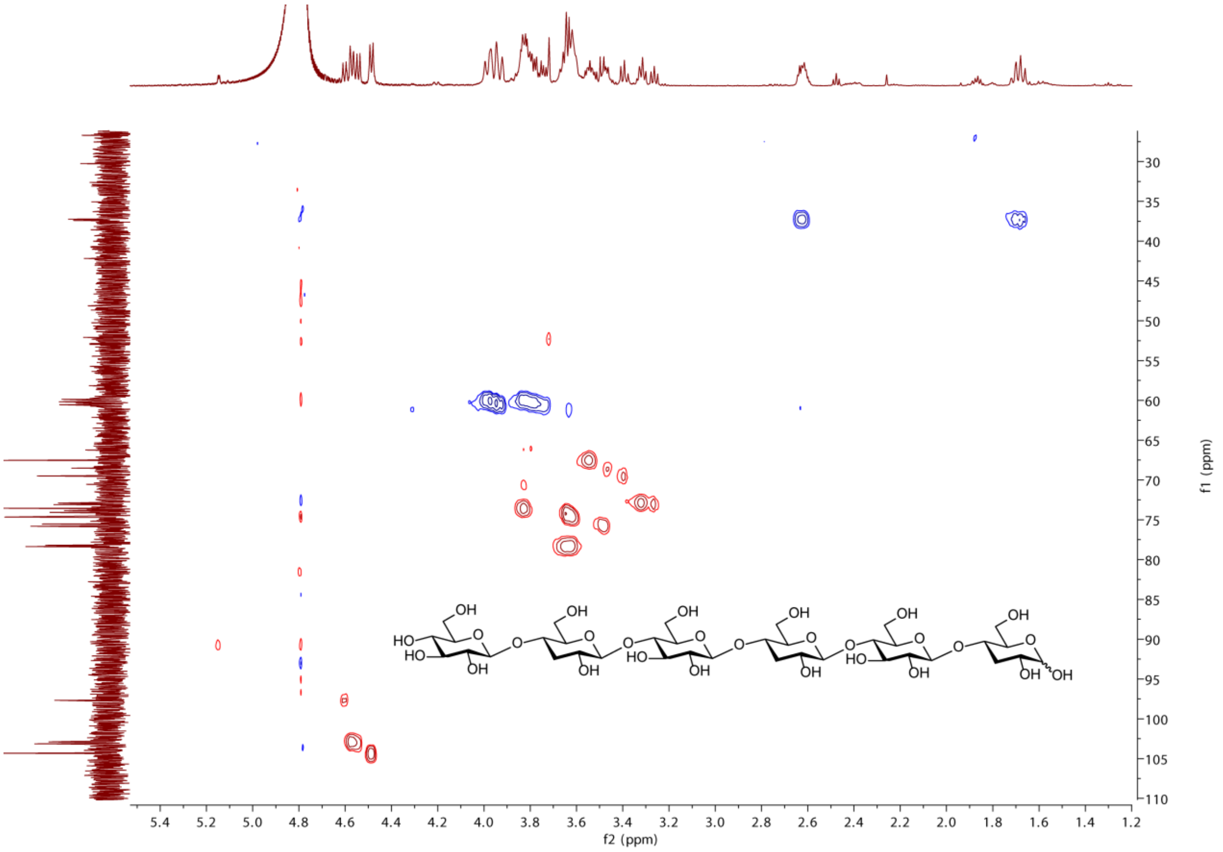

3.5.1.14. *Synthesis of NNNNNN-OH*

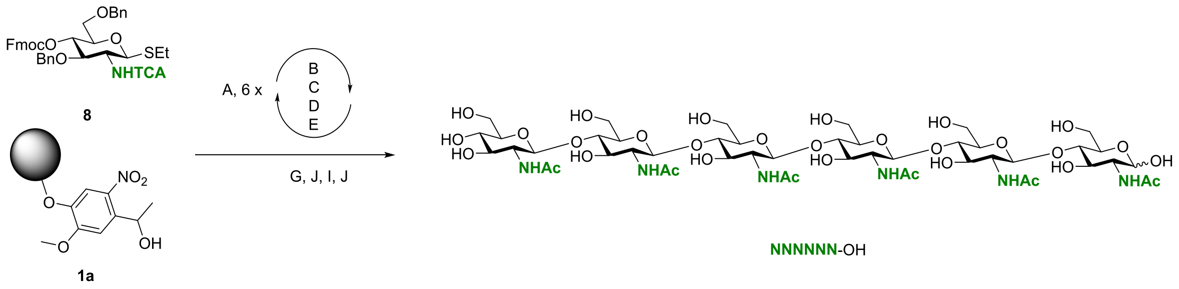

Module		Conditions
	**A**: Resin Preparation for Synthesis	
6	**B**: Acidic Wash with TMSOTf Solution	
	**C**: Thioglycoside Glycosylation	**8**, 8 equiv.(-20°C for 5 min, 0°C for 20 min)
	**D**: Capping	
	**E**: Fmoc Deprotection	
	**G**: Cleavage from Solid Support	
	**J**: Purification	**Method B**, t_R = 32.0 and 34.0 min
	**I**: Hydrogenolysis	
	**J**: Purification	**Method E**, t_R = 29.0 and 30.1 min

Automated synthesis, global deprotection, and purification afforded **NNNNNN-OH** as white solid (1.3 mg, 8% overall yield).

Analytical data for **NNNNNN-OH**: ^{1}H NMR (600 MHz, D_2O) δ 5.40 – 5.38 (m, 1H), 5.21 (d, *J* = 2.8 Hz, 0.55H, α-H1), 4.71 (d, *J* = 8.2 Hz, 0.45H, β-H1), 4.60 (dd, *J* = 8.1, 5.4 Hz, 5H), 3.99 – 3.45 (m, 39H), 2.13 – 2.03 (m, 18H); ^{13}C NMR (151 MHz, D_2O) δ 174.53, 174.50, 174.37, 170.96, 101.37, 101.15, 94.74 (β-C1), 90.36 (α-C1), 79.54, 79.06, 78.83, 75.82, 74.49, 74.45, 74.42, 73.35, 72.04, 71.97, 69.91, 69.61, 69.15, 60.45, 59.92, 59.89, 59.85, 55.49, 54.98, 54.96, 54.96, 54.92, 54.92, 53.56, 22.08, 22.02; $[\alpha]_D^{20}$ +7.61 (c 0.17, H_2O); IR (neat) ν_{max} = 3256, 1649, 1443, 1355, 1311, 1078 cm^{-1};m/z (HRMS+) 1259.476 $[M + Na]^+$ ($C_{48}H_{81}N_6O_{31}Na$ requires 1259.477).

RP-HPLC of NNNNNN-OH (ELSD trace, Method E, t_R = 29.0 and 30.1 min)

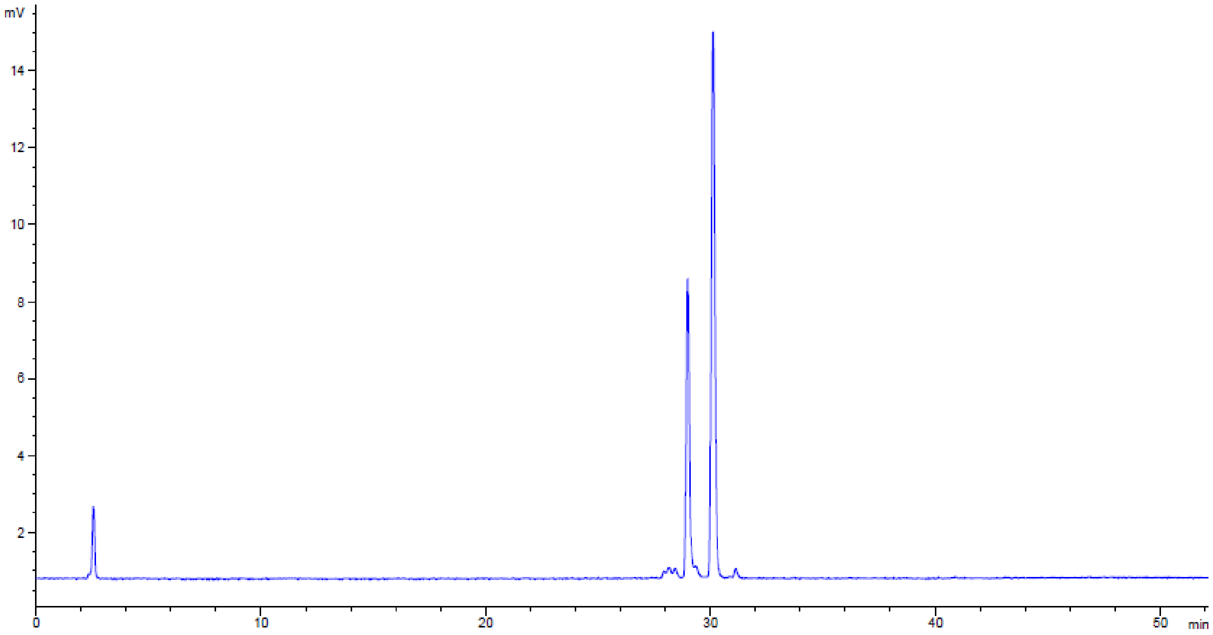

^{1}H NMR of NNNNNN-OH (600 MHz, D_2O)

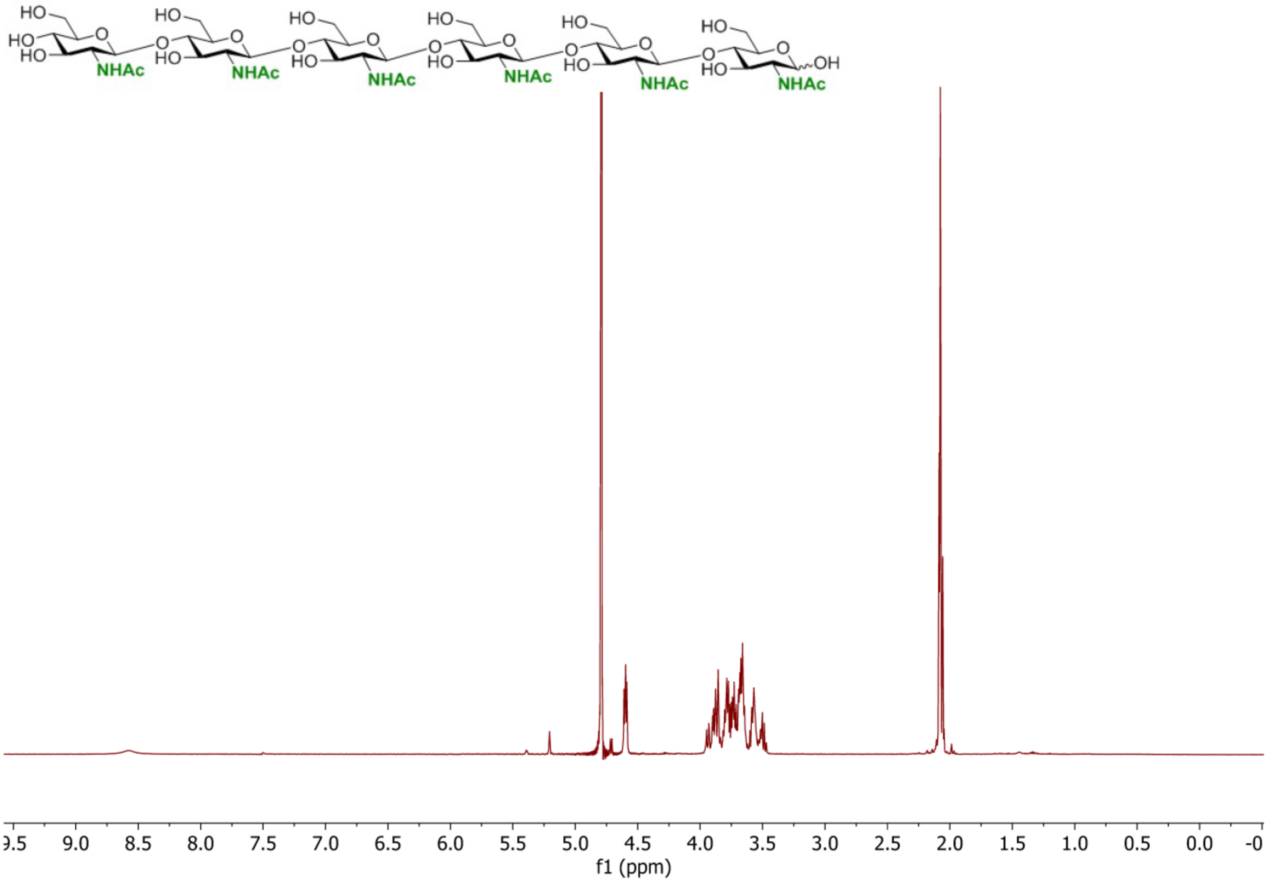

^{13}C NMR of NNNNNN-OH (151 MHz, D_2O)

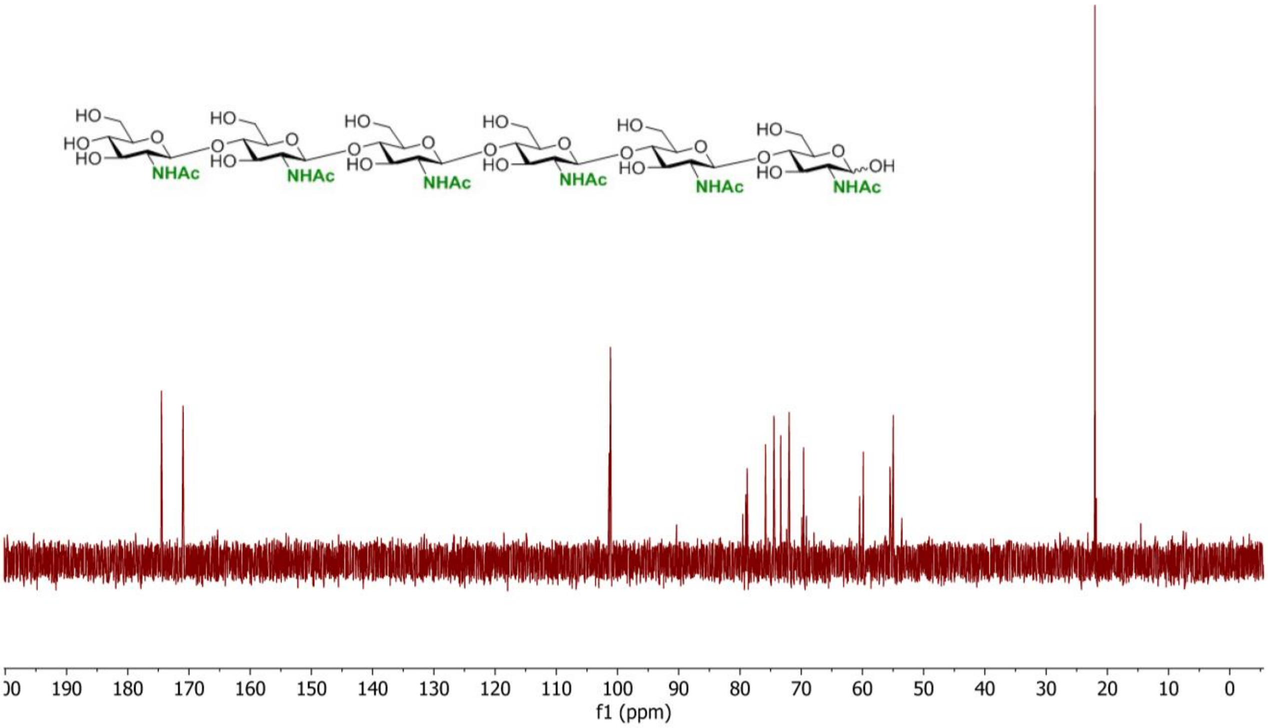

HSQC NMR of NNNNNN-OH (D_2O)

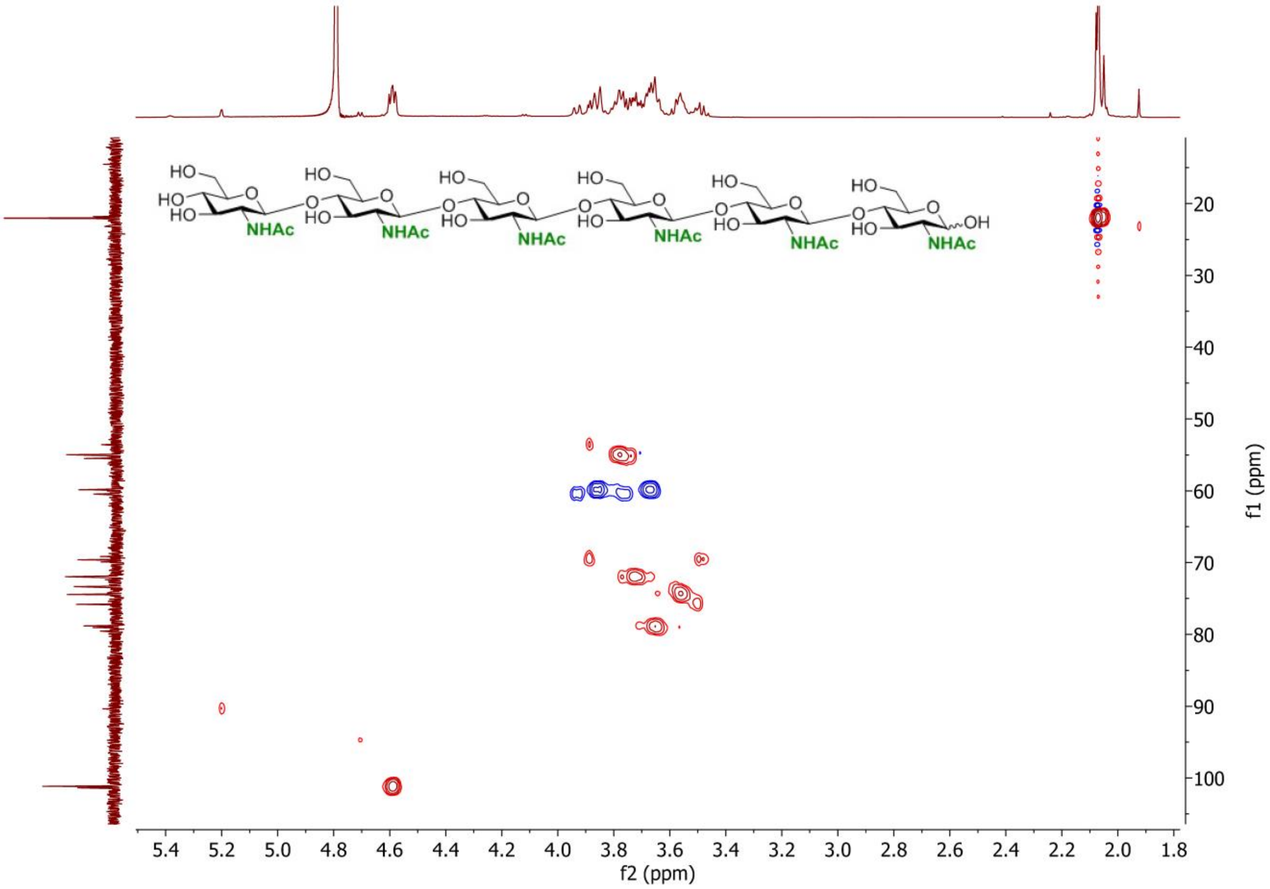

3.5.1.15. *Synthesis of ANAANA-OH*

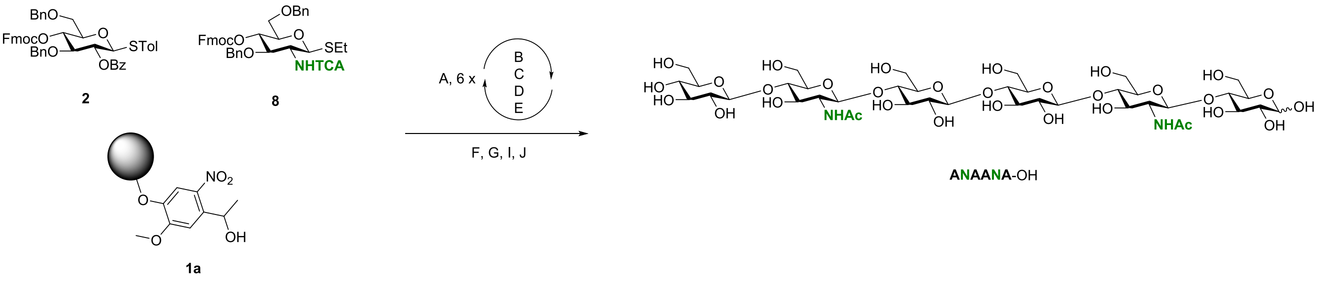

	Module	Conditions
	**A**: Resin Preparation for Synthesis	
2	**B**: Acidic Wash with TMSOTf Solution	
	**C**: Thioglycoside Glycosylation	**2**, 6.5 equiv.(-20°C for 5 min, 0°C for 20 min)
	**D**: Capping	
	**E**: Fmoc Deprotection	
	**B**: Acidic Wash with TMSOTf Solution	
	**C**: Thioglycoside Glycosylation	**8**, 8 equiv.(-20°C for 5 min, 0°C for 20 min)
	**D**: Capping	
	**E**: Fmoc Deprotection	
	**B**: Acidic Wash with TMSOTf Solution	
	**C**: Thioglycoside Glycosylation	**2**, 6.5 equiv.(-20°C for 5 min, 0°C for 20 min)
	**D**: Capping	
	**E**: Fmoc Deprotection	
	**F**: On-resin Methanolysis	
	**G**: Cleavage from Solid Support	
	**I**: Hydrogenolysis	
	**J**: Purification	**Method E**, t_R = 35.8 and 36.2 min

Automated synthesis, global deprotection, and purification afforded **ANAANA-OH** as white solid (3.8 mg, 28% overall yield).

Analytical data for **ANAANA-OH**: ^{1}H NMR (600 MHz, D_2O) δ 5.23 (d, *J* = 3.7 Hz, 0.38H), 4.67 (d, *J* = 8.0 Hz, 0.62H, β-H1), 4.63 – 4.50 (m, 5H), 4.00 (ddd, *J* = 20.1, 12.3, 2.1 Hz, 3H), 3.93 (dd, *J* = 12.4, 2.2 Hz, 1H), 3.90 – 3.49 (m, 28H), 3.43 (dd, *J* = 9.9, 9.1 Hz, 1H), 3.39 – 3.31 (m, 3H), 3.28 (dd, *J* = 9.4, 8.0 Hz, 0.6H), 2.08 (s, 6H); ^{13}C NMR (151 MHz, D_2O) δ 174.50, 170.97, 102.49, 102.29, 102.17, 101.23, 101.22, 101.18, 95.60 (β-C1), 91.55 (α-C1), 79.29, 79.08, 78.88, 78.46, 78.41, 78.38, 78.08, 75.93, 75.40, 74.78, 74.70, 74.49, 74.46, 74.28, 74.05, 74.02, 73.91, 73.66, 73.08, 72.87, 72.70, 71.93, 71.91, 71.34, 69.36, 61.51, 60.68, 60.50, 60.05, 59.93, 59.91, 59.81, 59.78, 59.76, 55.26, 55.21, 22.04; $[\alpha]_D^{20}$ +15.51 (c 0.2, H_2O); IR (neat) ν_{max} = 3347, 2925, 1645, 1564, 1379, 1029 cm^{-1}; m/z (HRMS+) 1095.368 $[M + Na]^+$ ($C_{40}H_{68}N_2O_{31}Na$ requires 1095.368).

RP-HPLC of ANAANA-OH (ELSD trace, Method E, t_R = 35.8 and 36.2 min)

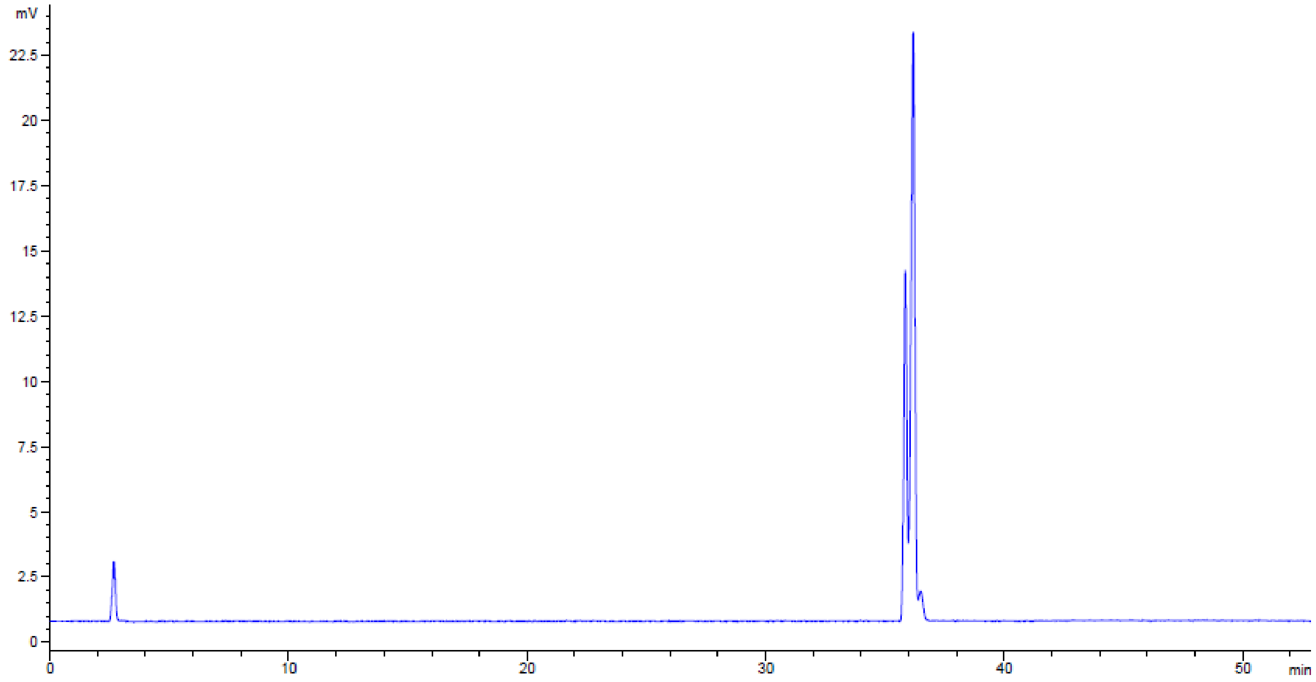

\

^{1}H NMR of ANAANA-OH (600 MHz, D_2O)

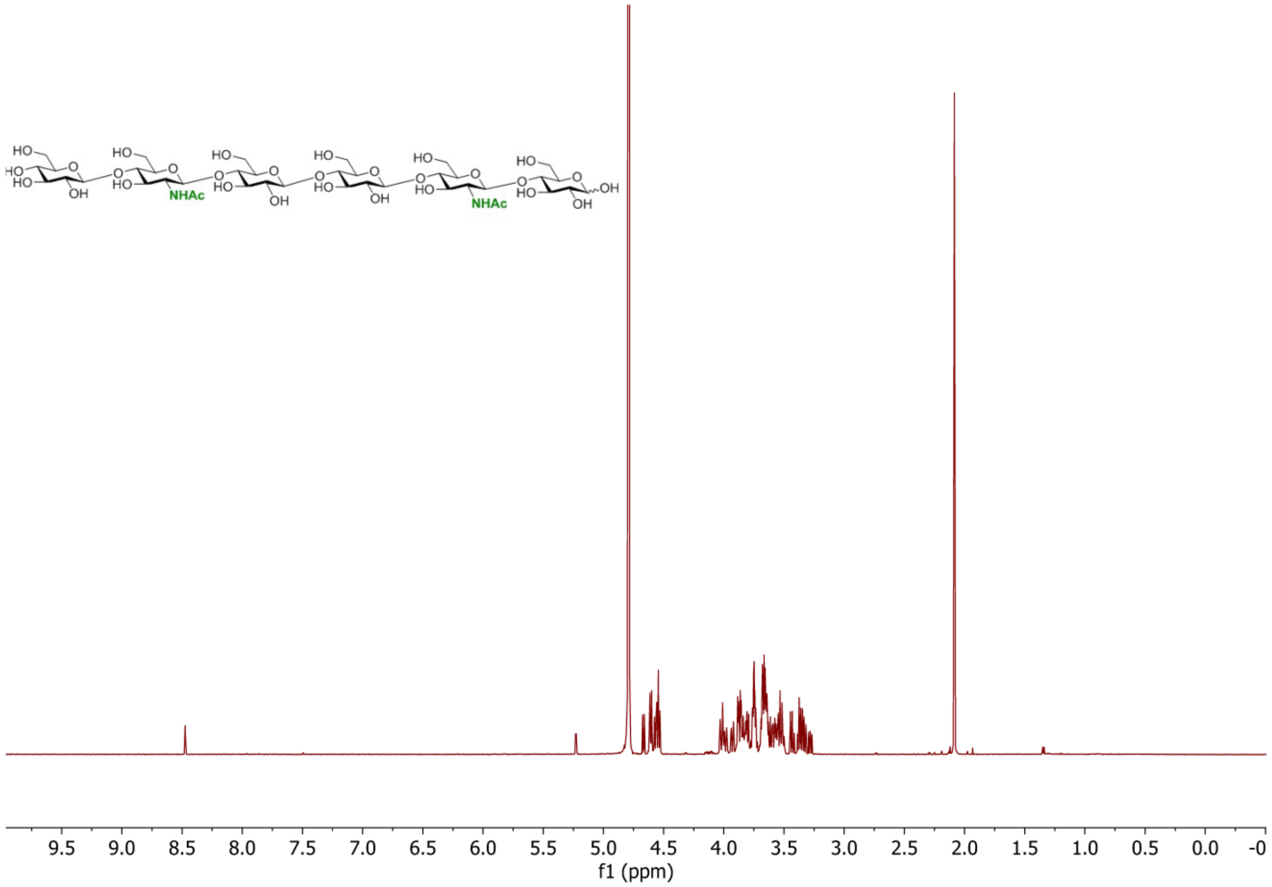

^{13}C NMR of ANAANA-OH (151 MHz, D_2O)

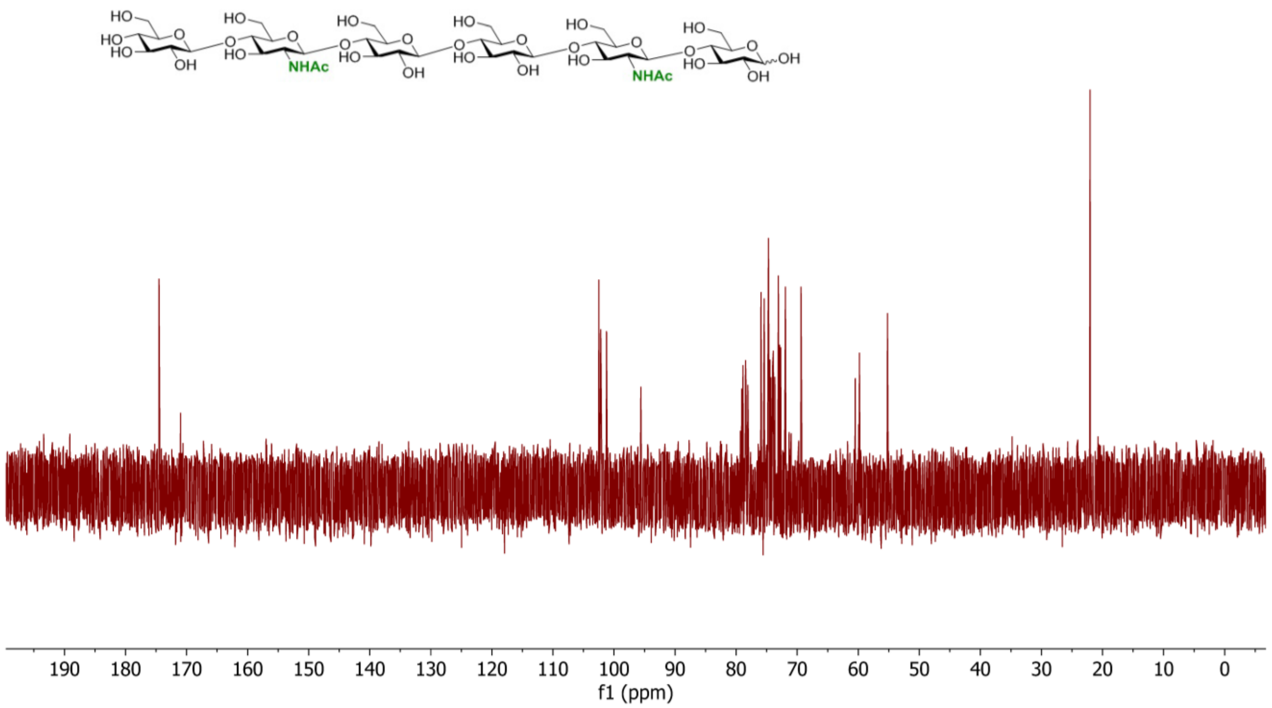

HSQC NMR of ANAANA-OH (D_2O)

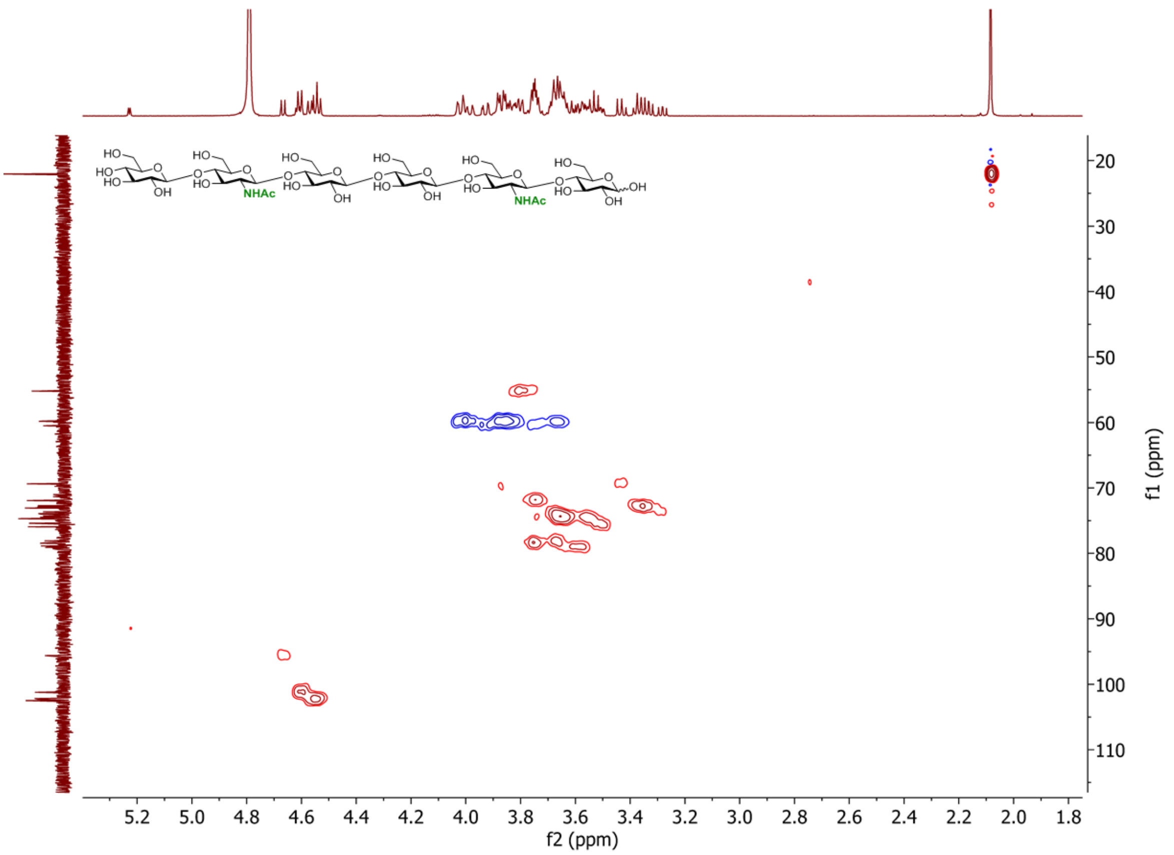

3.5.2. Synthesis of 12-mers

3.5.2.1. ***Synthesis of AAAAAAAAAAAA-OH***

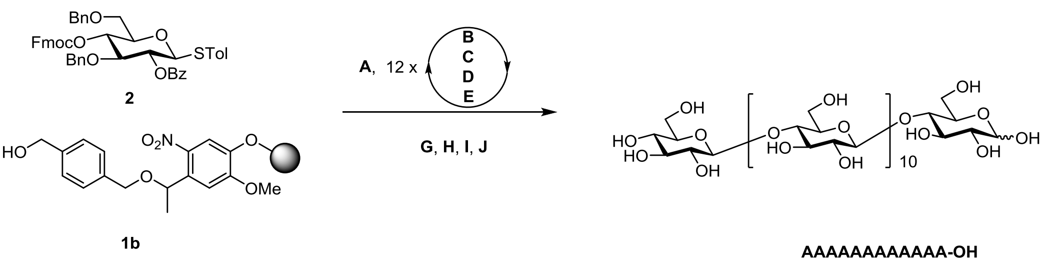

Module	Conditions
**A**: Resin Preparation for Synthesis	
12 { **B**: Acidic Wash with TMSOTf Solution	
**C**: Thioglycoside Glycosylation	**2**, 6.5 equiv.(-20°C for 5 min, 0°C for 20 min)
**D**: Capping	
**E**: Fmoc Deprotection	
**F**: On-resin Methanolysis	
**G**: Cleavage from Solid Support	
**I**: Hydrogenolysis	
**J**: Purification	**Method M**

Automated synthesis, global deprotection, and purification afforded **AAAAAAAAAAAA-OH** as white solid (0.6 mg, 2% overall yield).

Analytical data for **AAAAAAAAAAAA-OH**: ^{1}H NMR (700 MHz, D_2O) δ 5.24 (d, *J* = 3.7 Hz, 0.36 H, α-H1), 4.67 (d, *J* = 7.9 Hz, 0.64 H, β-H1), 4.57 – 4.51 (m, 11H), 3.99 (d, *J* = 11.9 Hz, 8H), 3.95 – 3.90 (m, 4H), 3.88 (d, *J* = 11.6 Hz, 2H), 3.84 (dd, *J* = 12.9, 4.6 Hz, 8H), 3.81 (d, *J* = 9.3 Hz, 3H), 3.79 (d, *J* = 7.6 Hz, 1H), 3.76 – 3.73 (m, 1H), 3.69 (q, *J* = 9.5 Hz, 17H), 3.66 – 3.61 (m, 14H), 3.54 – 4.48 (m, 3H), 3.43 (t, *J* = 9.4 Hz, 1H), 3.37 (t, *J* = 8.5 Hz, 8H), 3.31 (dt, *J* = 22.7, 8.7 Hz, 2H); ^{13}C NMR (176 MHz, D_2O) δ 102.34, 78.52, 78.48, 78.35, 78.27, 78.27, 74.82, 74.54, 74.01, 73.15, 73.04, 72.94, 71.32, 60.57, 59.87; solubility not enough for optical rotation measurement; IR (neat) ν_{max} = 3340, 2893, 1644, 1030 cm^{-1}; m/z (HRMS+) 1985.635 $[M + Na]^+$ ($C_{72}H_{122}NaO_{61}$ requires 1985.634).

RP-HPLC of AAAAAAAAAAAA-OH (ELSD trace, Method C, t_R = 16.9 min)

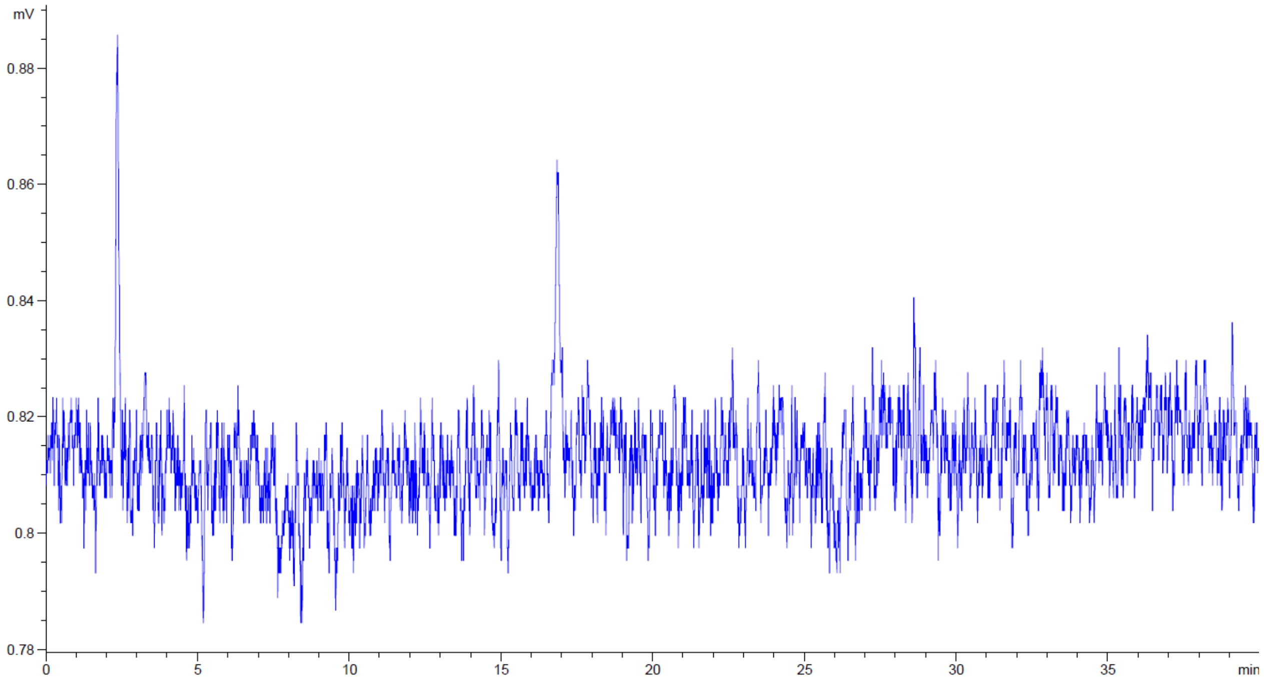

^{1}H NMR of AAAAAAAAAAAA-OH (700 MHz, D_2O)

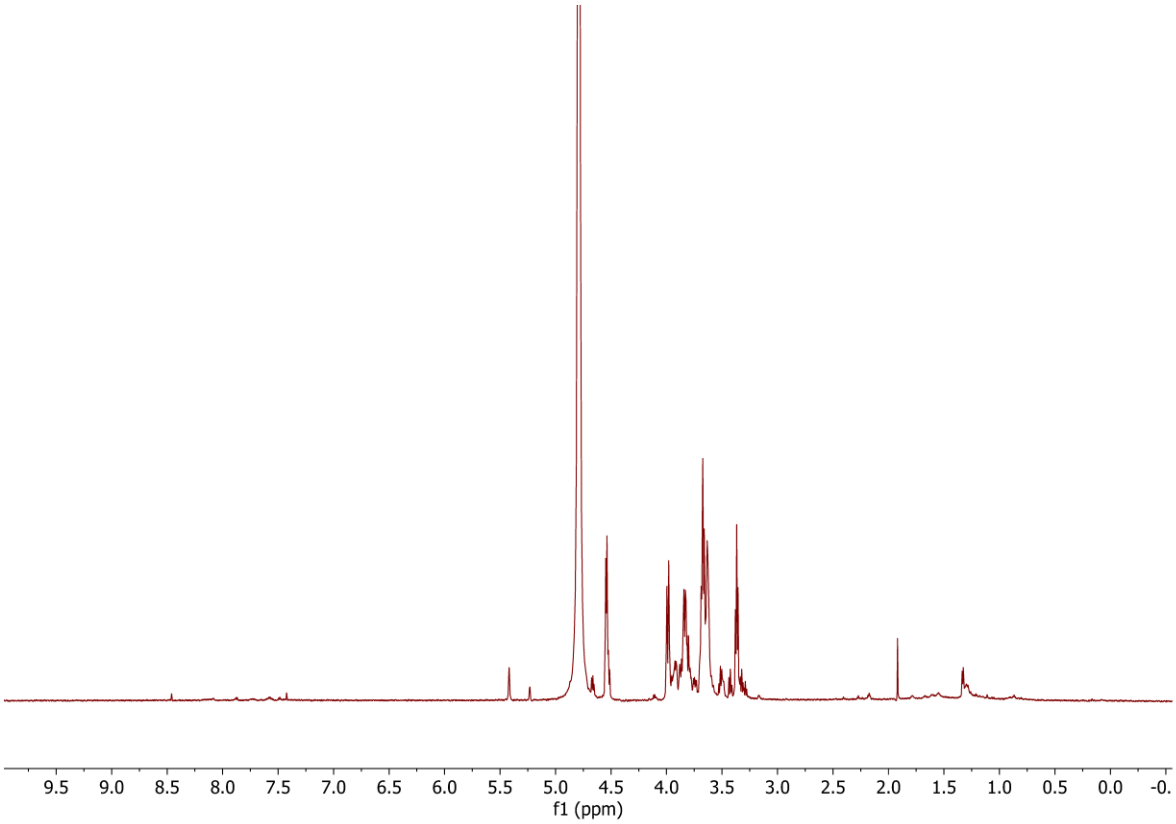

^{13}C NMR of AAAAAAAAAAAA-OH (176 MHz, D_2O)

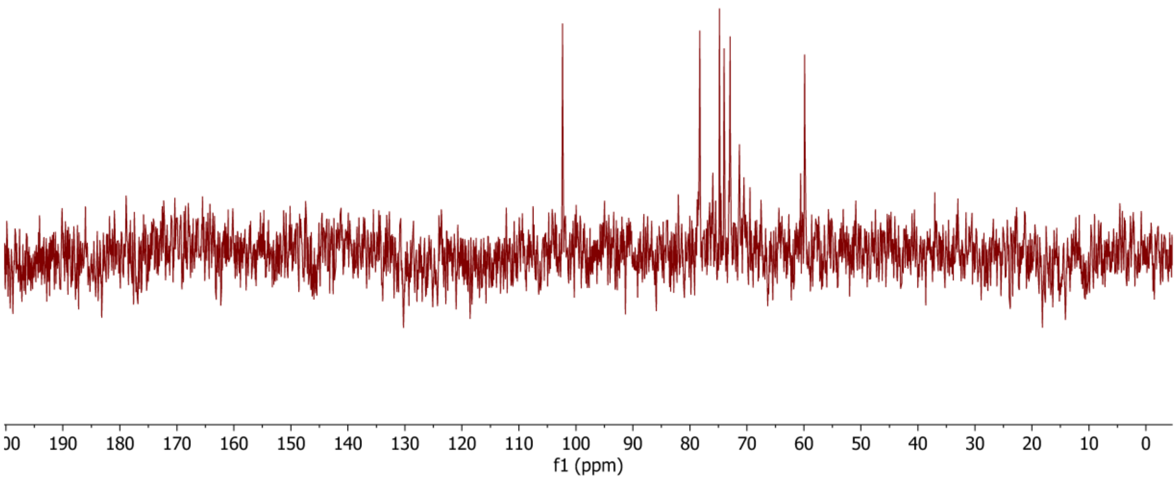

HSQC NMR of AAAAAAAAAAAA-OH (D_2O)

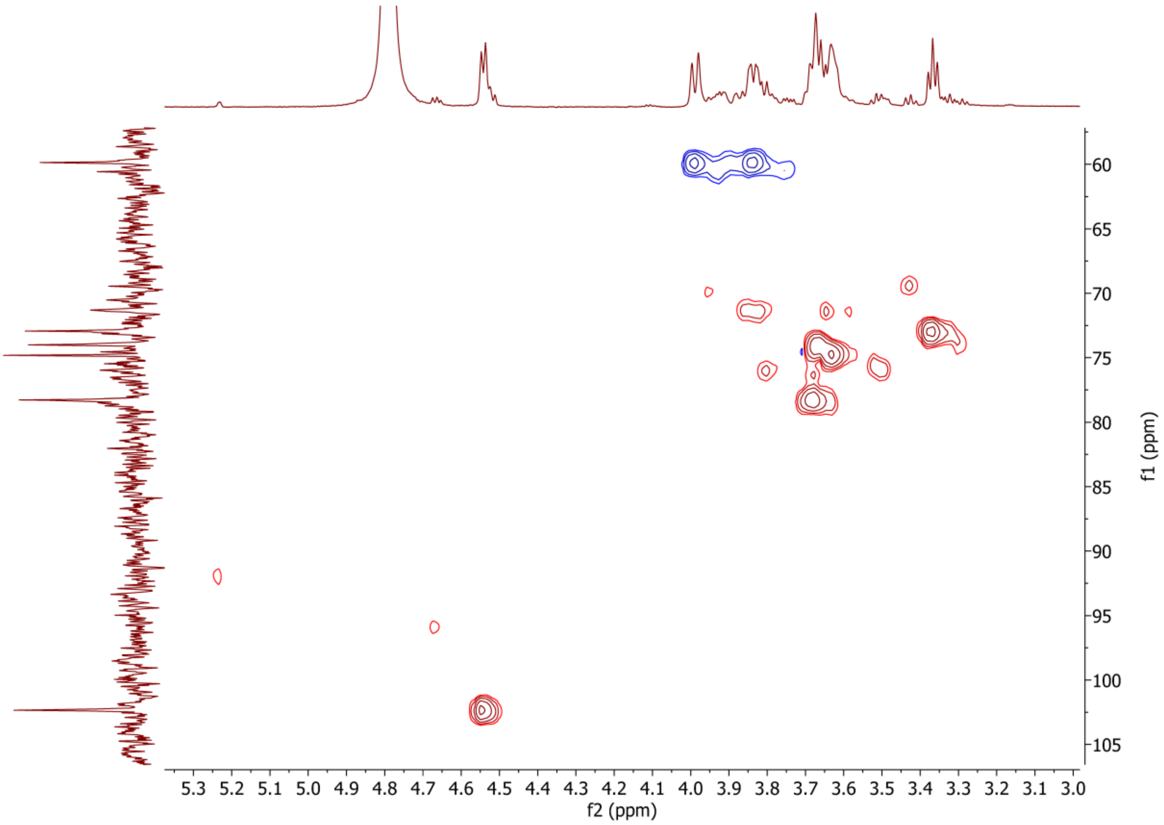

3.5.2.2. *Synthesis of AAABBBAAABBB-OH*

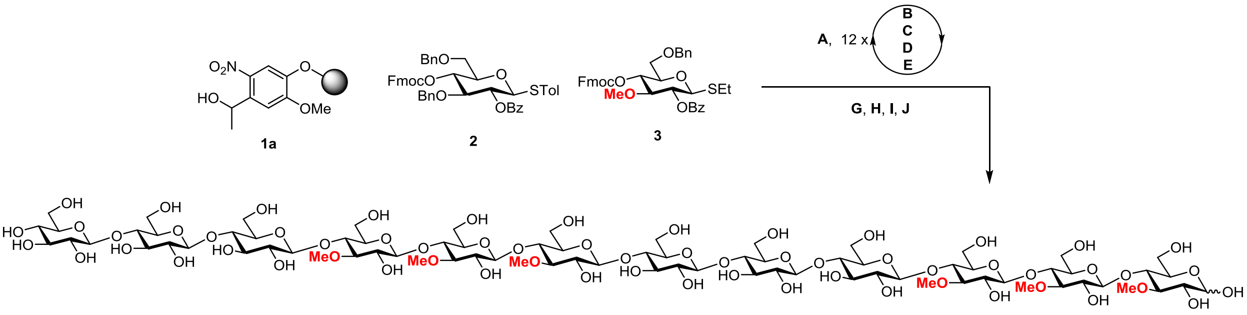

Module	Conditions
**A**: Resin Preparation for Synthesis	
3 × **B**: Acidic Wash with TMSOTf Solution	
**C**: Thioglycoside Glycosylation	**3**, 6.5 equiv.(-20°C for 5 min, 0°C for 20 min)
**D**: Capping	
**E**: Fmoc Deprotection	
3 × **B**: Acidic Wash with TMSOTf Solution	
**C**: Thioglycoside Glycosylation	**2**, 6.5 equiv.(-20°C for 5 min, 0°C for 20 min)
**D**: Capping	
**E**: Fmoc Deprotection	
3 × **B**: Acidic Wash with TMSOTf Solution	
**C**: Thioglycoside Glycosylation	**3**, 6.5 equiv.(-20°C for 5 min, 0°C for 20 min)
**D**: Capping	
**E**: Fmoc Deprotection	
3 × **B**: Acidic Wash with TMSOTf Solution	
**C**: Thioglycoside Glycosylation	**2**, 6.5 equiv.(-20°C for 5 min, 0°C for 20 min)
**D**: Capping	
**E**: Fmoc Deprotection	
**F**: On-resin Methanolysis	
**G**: Cleavage from Solid Support	
**I**: Hydrogenolysis	
**J**: Purification	**Method M**

Automated synthesis, global deprotection, and purification afforded **AAABBBAAABBB-OH** as white solid (1.8 mg, 7% overall yield).

Analytical data for **AAABBBAAABBB-OH**: ^{1}H NMR (600 MHz, D_2O) δ 5.24 (d, *J* = 3.6 Hz, 0.39 H, α-H1), 4.69 (d, *J* = 7.8 Hz, 0.61 H, β-H1), 4.63 – 4.51 (m, 11H), 4.05 – 4.00 (m, 8H), 4.00 – 3.92 (m, 4H), 3.85 (tdd, *J* = 22.6, 12.8, 4.9 Hz, 17H), 3.77 (dd, *J* = 12.6, 5.9 Hz, 2H), 3.73 – 3.65 (m, 13H), 3.65 – 3.62 (m, 18H), 3.59 (td, *J* = 16.7, 14.9, 5.4 Hz, 7H), 3.55 – 3.50 (m, 3H), 3.50 – 3.42 (m, 11H), 3.42 – 3.31 (m,

7H); ^{13}C NMR (151 MHz, D_2O) δ 102.49, 102.28, 102.19, 95.72 (β-C1), 91.75 (α-C1), 83.51, 83.45, 83.39, 83.39, 83.23, 78.35, 78.32, 78.18, 75.91, 75.58, 75.45, 75.43, 75.41, 74.99, 74.77, 74.75, 74.12, 73.98, 73.93, 73.30, 73.18, 73.07, 72.87, 72.82, 72.77, 72.71, 72.70, 72.68, 72.65, 72.27, 70.69, 69.38, 60.50, 59.94, 59.90, 59.89, 59.88, 59.82, 59.79, 59.50, 59.15, 59.12, 59.07, 59.03, 59.00; $[\alpha]_D^{20}$ +10.86 (c 0.2, H_2O); IR (neat) ν_{max} = 3357, 2929, 1647, 1024 cm^{-1}; m/z (HRMS+) 2069.728 $[M + Na]^+$ ($C_{78}H_{134}NaO_{61}$ requires 2069.728).

RP-HPLC of AAABBBAAABBB-OH (ELSD trace, Method C, t_R = 19.8 min)

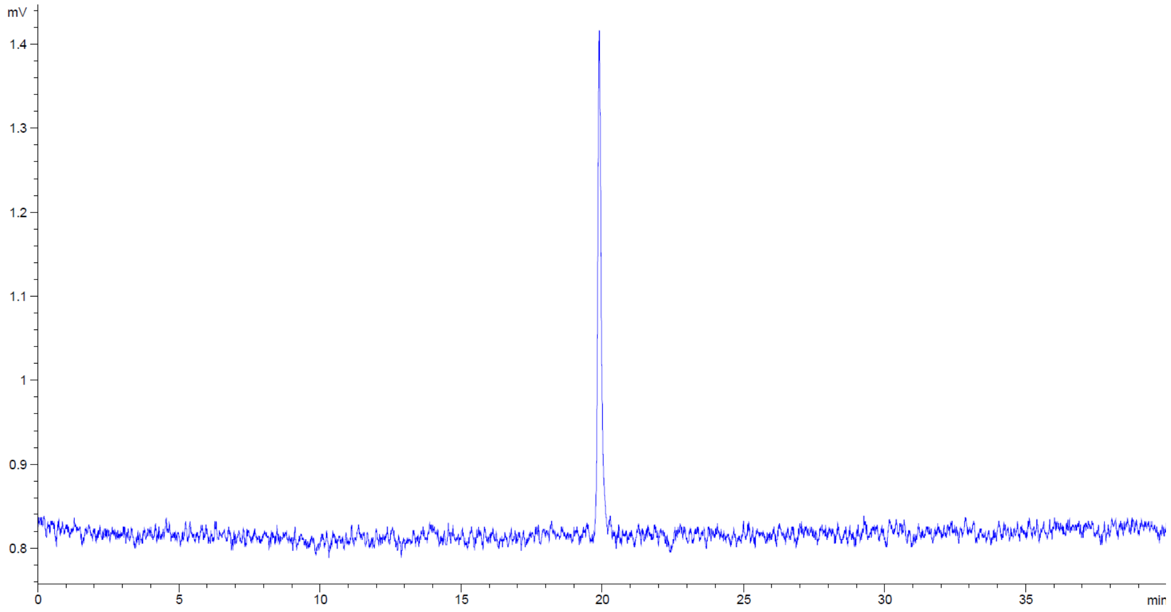

^{1}H NMR of AAABBBAAABBB-OH (600 MHz, D_2O)

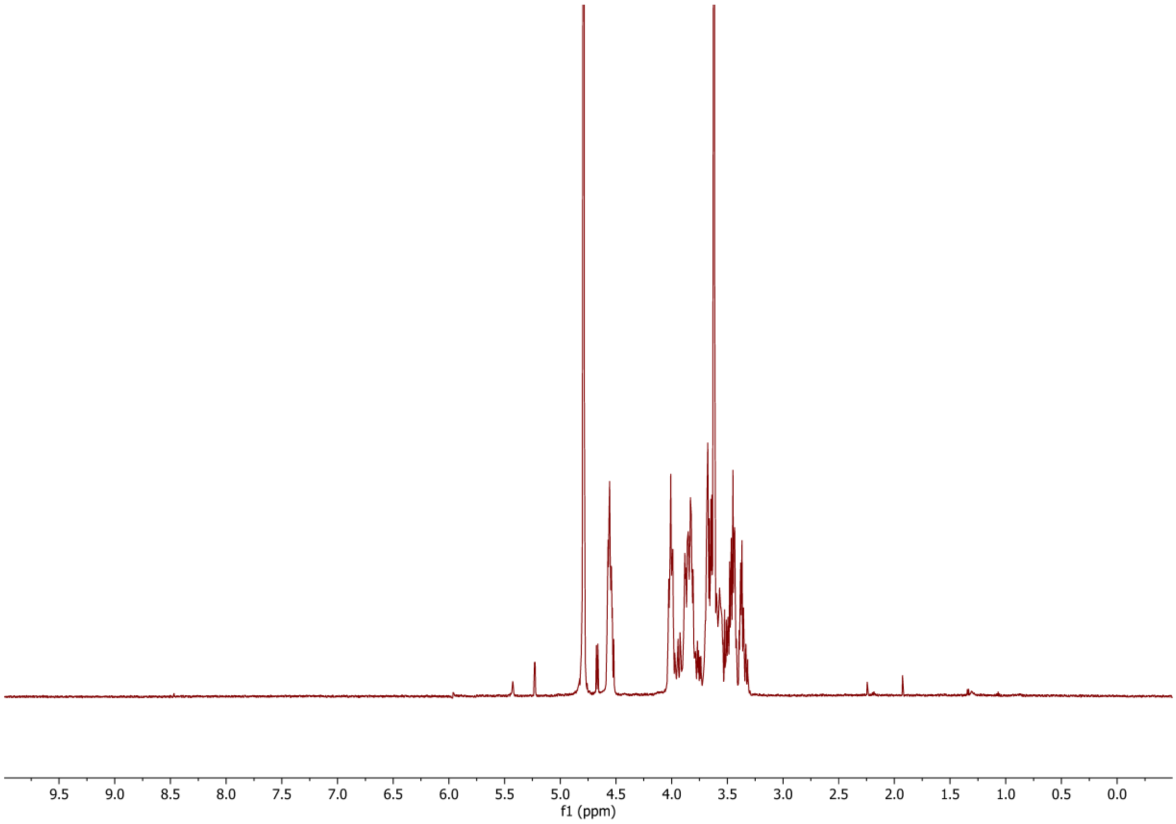

^{13}C NMR AAABBBAAABBB-OH (151 MHz, D_2O)

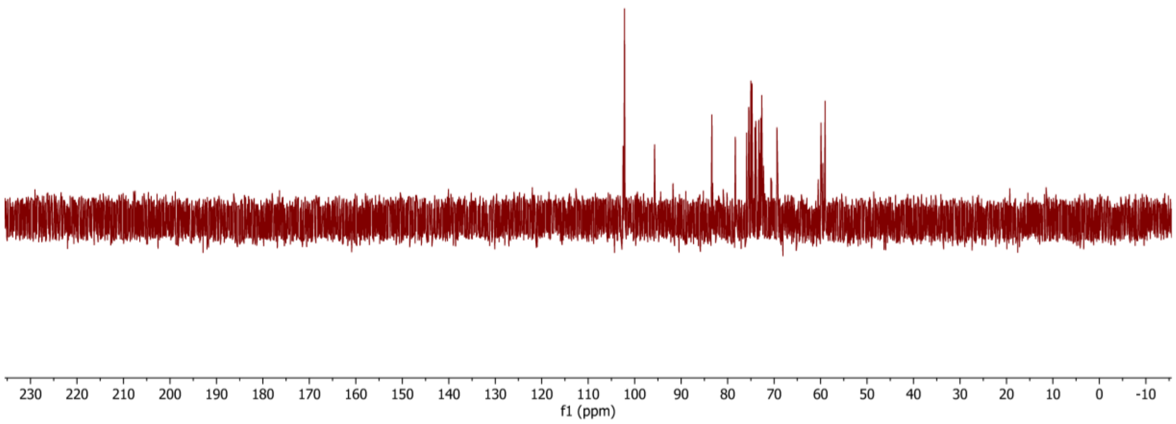

HSQC NMR of AAABBBAAABBB-OH (D_2O)

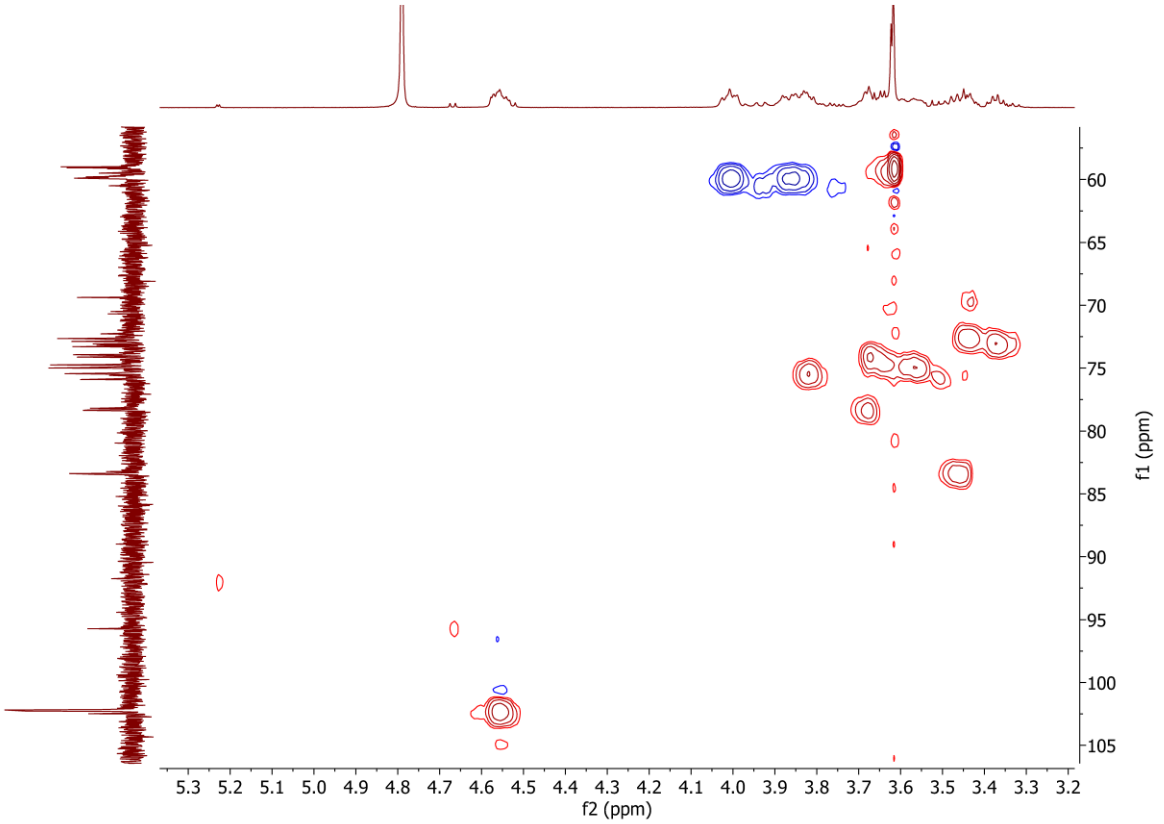

3.5.2.3. *Synthesis of ABAABAAAABBB-OH*

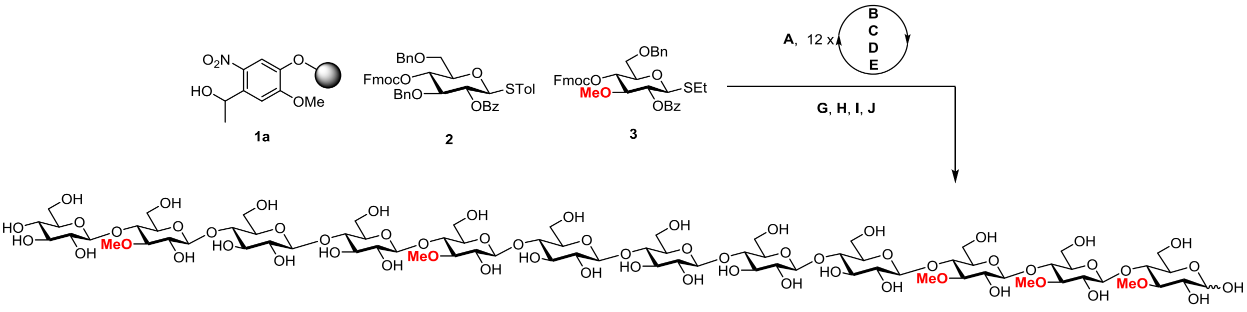

ABAABAAAABBB-OH

	Module	Conditions
	**A**: Resin Preparation for Synthesis	
3	**B**: Acidic Wash with TMSOTf Solution	
	**C**: Thioglycoside Glycosylation	**3**, 6.5 equiv.(-20°C for 5 min, 0°C for 20 min)
	**D**: Capping	

	**E**: Fmoc Deprotection	
3	**B**: Acidic Wash with TMSOTf Solution	
	**C**: Thioglycoside Glycosylation	**2**, 6.5 equiv.(-20°C for 5 min, 0°C for 20 min)
	**D**: Capping	
	**E**: Fmoc Deprotection	
2	**B**: Acidic Wash with TMSOTf Solution	
	**C**: Thioglycoside Glycosylation	**2**, 6.5 equiv.(-20°C for 5 min, 0°C for 20 min)
	**D**: Capping	
	**E**: Fmoc Deprotection	
	**B**: Acidic Wash with TMSOTf Solution	
	**C**: Thioglycoside Glycosylation	**3**, 6.5 equiv.(-20°C for 5 min, 0°C for 20 min)
	**D**: Capping	
	**E**: Fmoc Deprotection	
	**B**: Acidic Wash with TMSOTf Solution	
	**C**: Thioglycoside Glycosylation	**2**, 6.5 equiv.(-20°C for 5 min, 0°C for 20 min)
	**D**: Capping	
	**E**: Fmoc Deprotection	
	**F**: On-resin Methanolysis	
	**G**: Cleavage from Solid Support	
	**I**: Hydrogenolysis	
	**J**: Purification	**Method M**

Automated synthesis, global deprotection, and purification afforded **ABAABAAAABBB-OH** as white solid (3.9 mg, 15% overall yield).

Analytical data for **ABAABAAAABBB-OH**: ^{1}H NMR (600 MHz, D_2O) δ 5.24 (d, *J* = 3.5 Hz, 0.43 H, α-H1), 4.68 (d, *J* = 7.8 Hz, 0.57 H, β-H1), 4.60 – 4.52 (m, 11H), 4.01 (d, *J* = 11.5 Hz, 8H), 3.98 – 3.92 (m, 3H), 3.92 – 3.81 (m, 16H), 3.81 – 3.74 (m, 3H), 3.74 – 3.64 (m, 18H), 3.64 – 3.61 (m, 15H), 3.61 – 3.54 (m, 6H), 3.54 – 3.42 (m, 11H), 3.41 – 3.35 (m, 6H), 3.35 – 3.31 (m, 1H); ^{13}C NMR (151 MHz, d_2o) δ 102.35, 102.33, 102.29, 102.24, 102.23, 102.20, 102.19, 95.72 (β-C1), 91.76(α-C1), 83.52, 83.46, 83.44, 83.43, 83.40, 83.23, 83.18, 83.14, 80.96, 78.36, 78.34, 78.21, 78.21, 75.97, 75.92, 75.91, 75.61, 75.60, 75.48, 75.46, 75.43, 75.42, 75.36, 74.99, 74.76, 74.16, 74.15, 74.13, 73.95, 73.52, 73.31, 73.27, 73.19, 72.87, 72.70, 72.66, 72.31, 60.69, 59.93, 59.92, 59.91, 59.89, 59.82, 59.81, 59.79, 59.50, 59.17, 59.12, 59.08, 59.04, 59.00; $[\alpha]_D^{20}$ +12.74 (c 0.2, H_2O); IR (neat) ν_{max} = 3376, 1019 cm^{-1}; m/z (HRMS+) 2055.714 $[M + Na]^+$ ($C_{77}H_{132}NaO_{61}$ requires 2055.712).

RP-HPLC of ABAABAAAABBB-OH (ELSD trace, Method C, t_R = 18.7, 19.3 min)

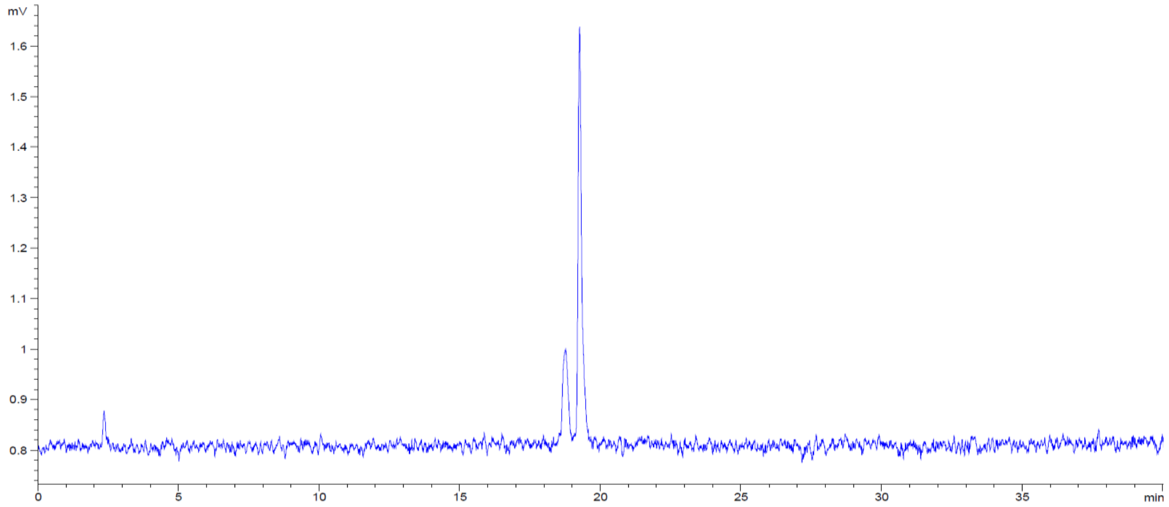

^{1}H NMR of ABAABAAAABBB-OH (600 MHz, D_2O)

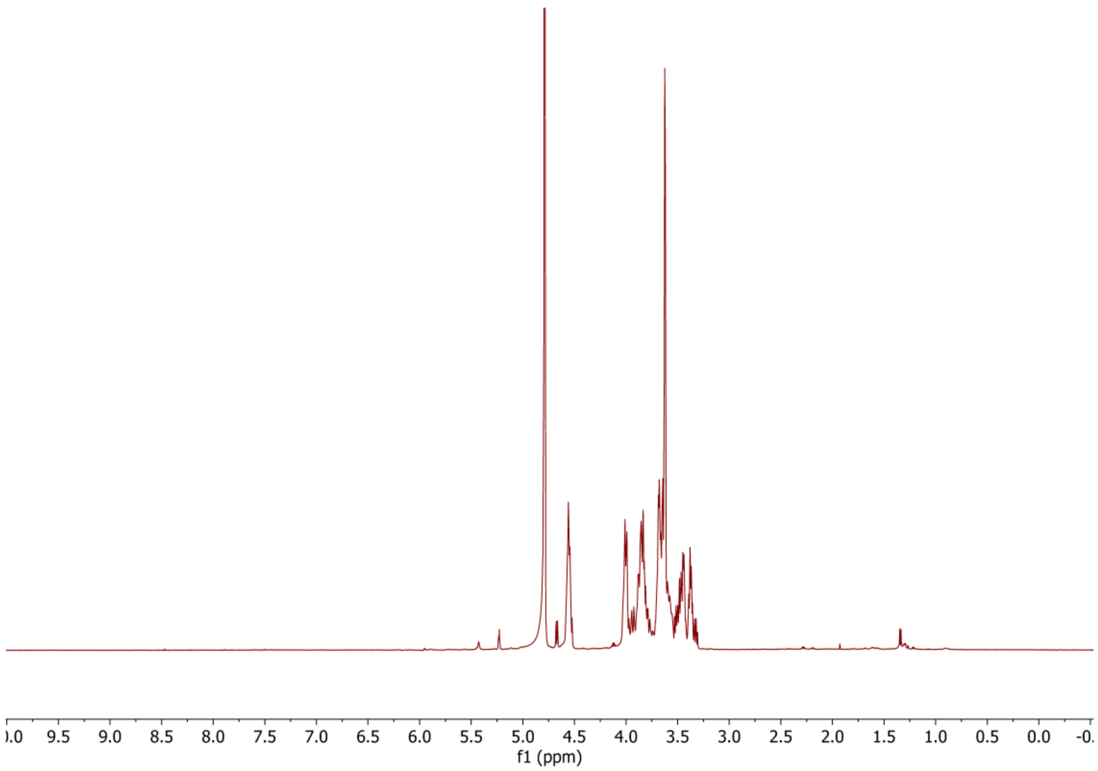

^{13}C NMR of ABAABAAAABBB-OH (151 MHz, D_2O)

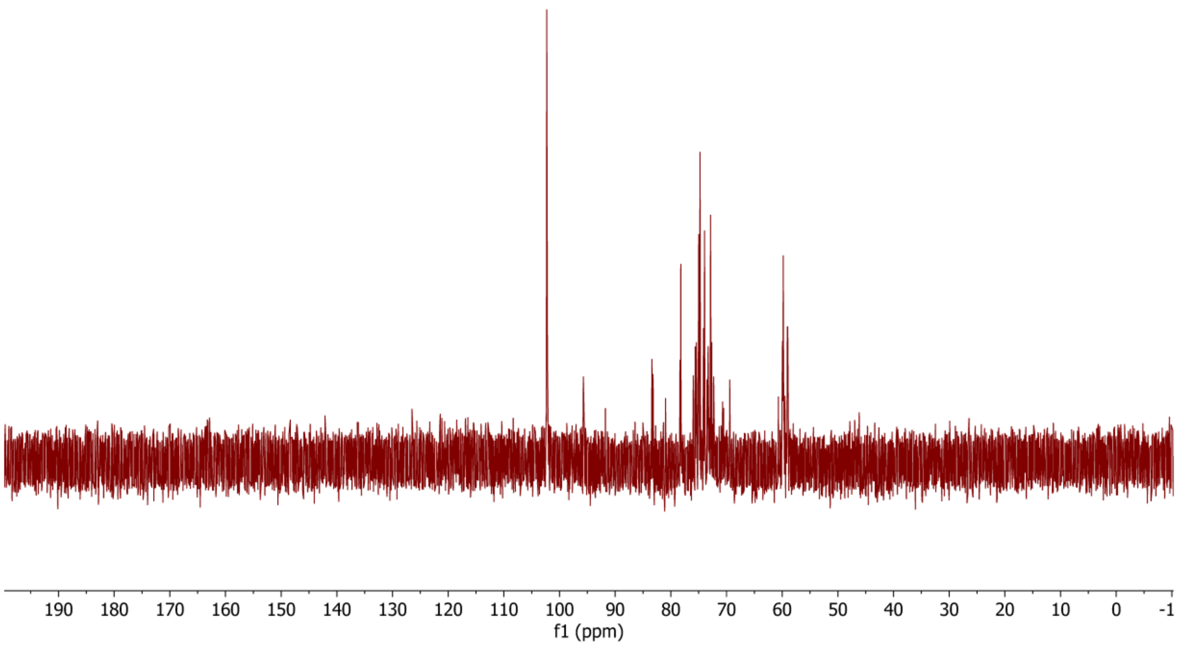

HSQC NMR of ABAABAAAABBB-OH (D_2O)

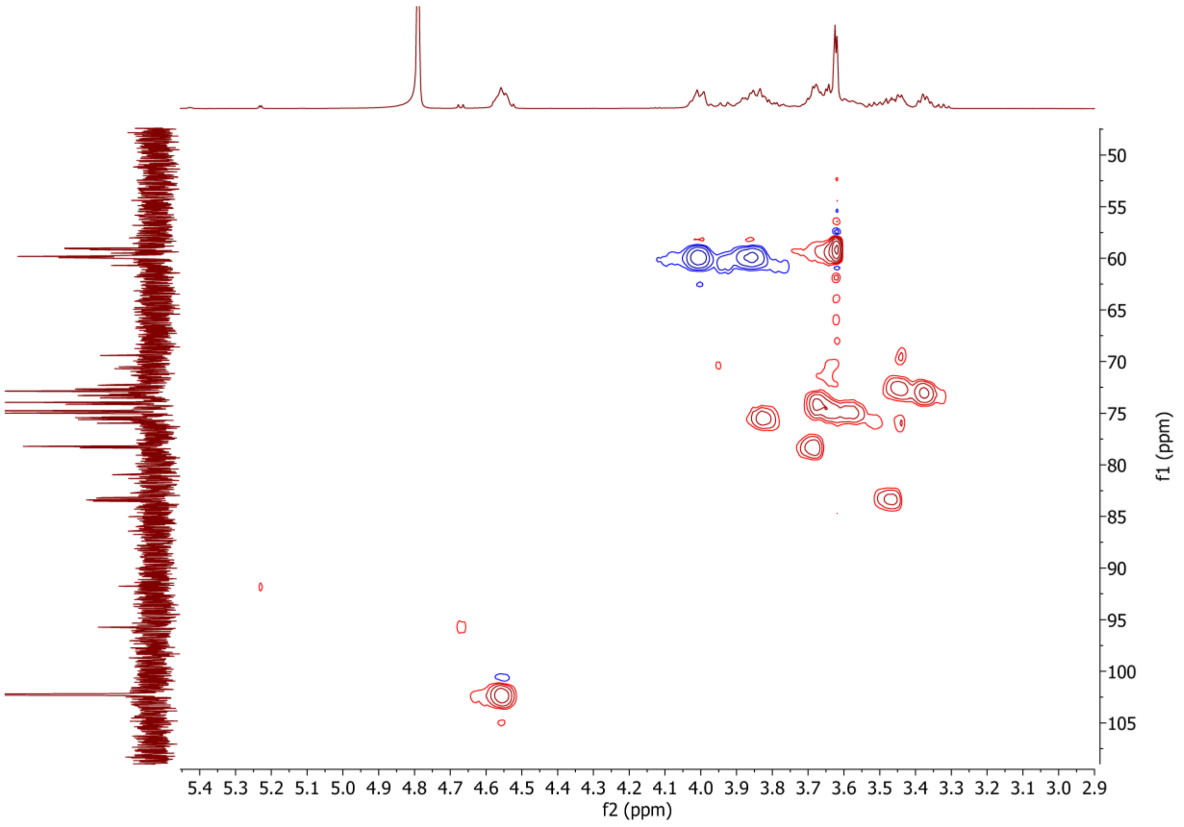

3.5.2.4. *Synthesis of ABAABAABAABA-OH*

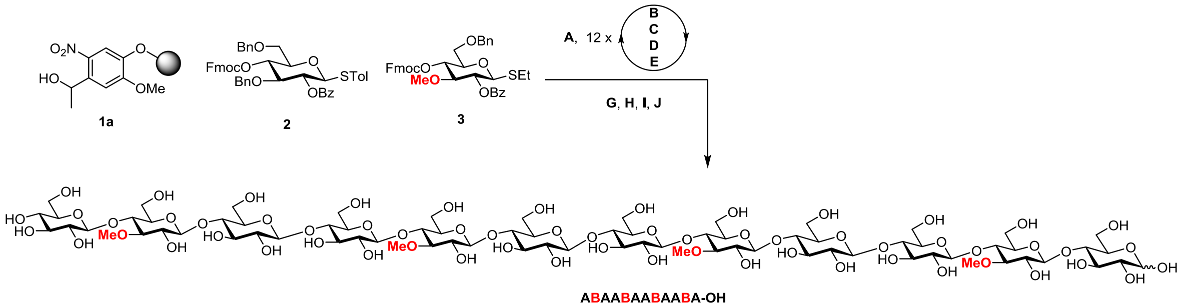

Module	Conditions
**A**: Resin Preparation for Synthesis	
**B**: Acidic Wash with TMSOTf Solution	
**C**: Thioglycoside Glycosylation	**2**, 6.5 equiv.(-20°C for 5 min, 0°C for 20 min)
**D**: Capping	
**E**: Fmoc Deprotection	
**B**: Acidic Wash with TMSOTf Solution	
**C**: Thioglycoside Glycosylation	**3**, 6.5 equiv.(-20°C for 5 min, 0°C for 20 min)
**D**: Capping	
**E**: Fmoc Deprotection	
**B**: Acidic Wash with TMSOTf Solution	
**C**: Thioglycoside Glycosylation	**2**, 6.5 equiv.(-20°C for 5 min, 0°C for 20 min)
**D**: Capping	
**E**: Fmoc Deprotection	
**F**: On-resin Methanolysis	
**G**: Cleavage from Solid Support	
**I**: Hydrogenolysis	
**J**: Purification	**Method M**

(Modules B–E above: 4×)

Automated synthesis, global deprotection, and purification afforded **ABAABAABAABA-OH** as white solid (1.8 mg, 7% overall yield).

Analytical data for **ABAABAABAABA-OH**: ^{1}H NMR (700 MHz, D_2O) δ 5.24 (d, *J* = 3.7 Hz, 0.38 H, α-H1), 4.67 (d, *J* = 8.0 Hz, 0.62 H, β-H1), 4.55 (td, *J* = 10.3, 9.0, 4.3 Hz, 11H), 3.96 (dd, *J* = 11.7, 5.0 Hz, 10H), 3.97 – 3.90 (m, 3H), 3.85 (dq, *J* = 22.9, 8.5, 6.6 Hz, 16H), 3.78 (dd, *J* = 12.6, 4.4 Hz, 1H), 3.67 (tt, *J* = 10.9, 5.3 Hz, 17H), 3.62 (s, 12H), 3.60 – 3.55 (m, 5H), 3.53 – 3.41 (m, 11H), 3.37 (q, *J* = 8.4 Hz, 7H), 3.30 (dt, *J* = 16.7, 8.6 Hz, 2H); ^{13}C NMR (176 MHz, D_2O) δ 102.40, 102.36, 102.27, 95.76 (β-C1), 91.82 (α-C1), 83.30, 83.25, 78.50, 78.41, 78.28, 76.04, 75.68, 75.49, 75.42, 75.09, 75.05, 74.85, 74.81, 74.27, 74.19, 74.03, 73.90, 73.58, 73.34, 72.95, 72.37, 72.32, 71.32, 71.23, 69.49, 60.75, 60.01, 59.90, 59.84, 59.83, 59.23, 59.14, 59.09; $[\alpha]_D^{20}$ +11.41 (c 0.2, H_2O); IR (neat) ν_{max} = 3360, 2926, 1034 cm^{-1}; m/z (HRMS+) 2041.697 $[M + Na]^+$ ($C_{76}H_{130}NaO_{61}$ requires 2041.696).

RP-HPLC of ABAABAABAABA-OH (ELSD trace, Method C, t_R = 18.8 min)

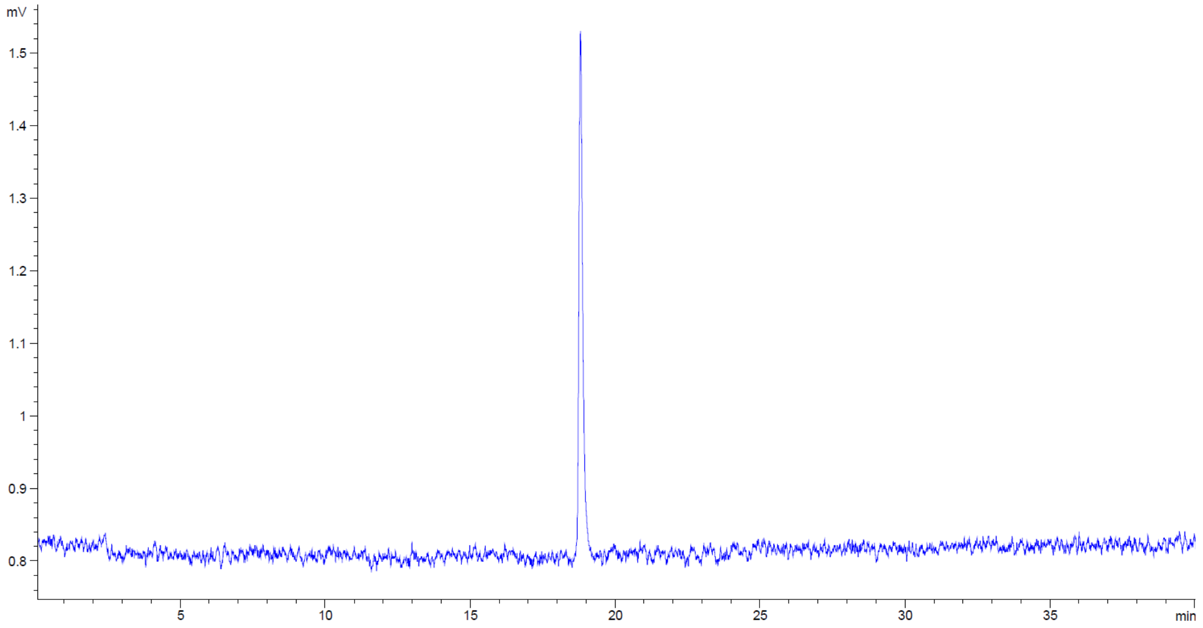

^{1}H NMR of ABAABAABAABA-OH (700 MHz, D_2O)

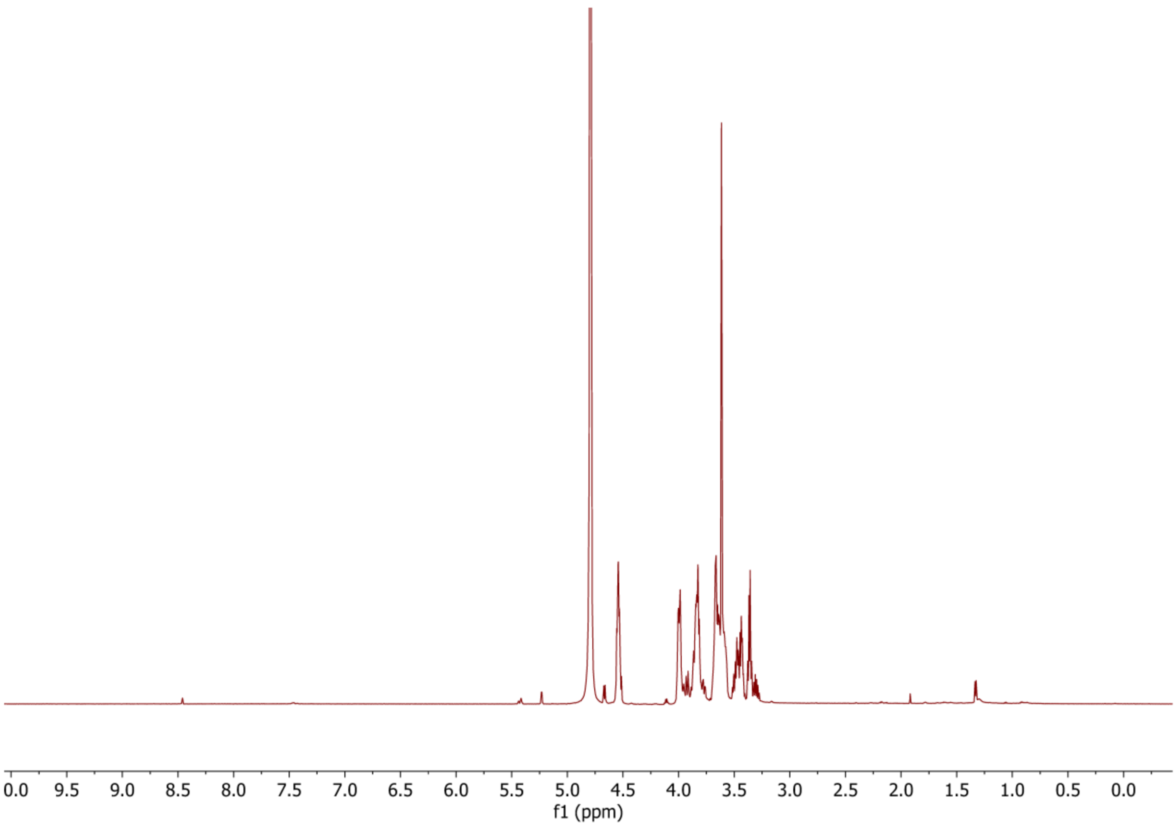

^{13}C NMR of ABAABAABAABA-OH (176 MHz, D_2O)

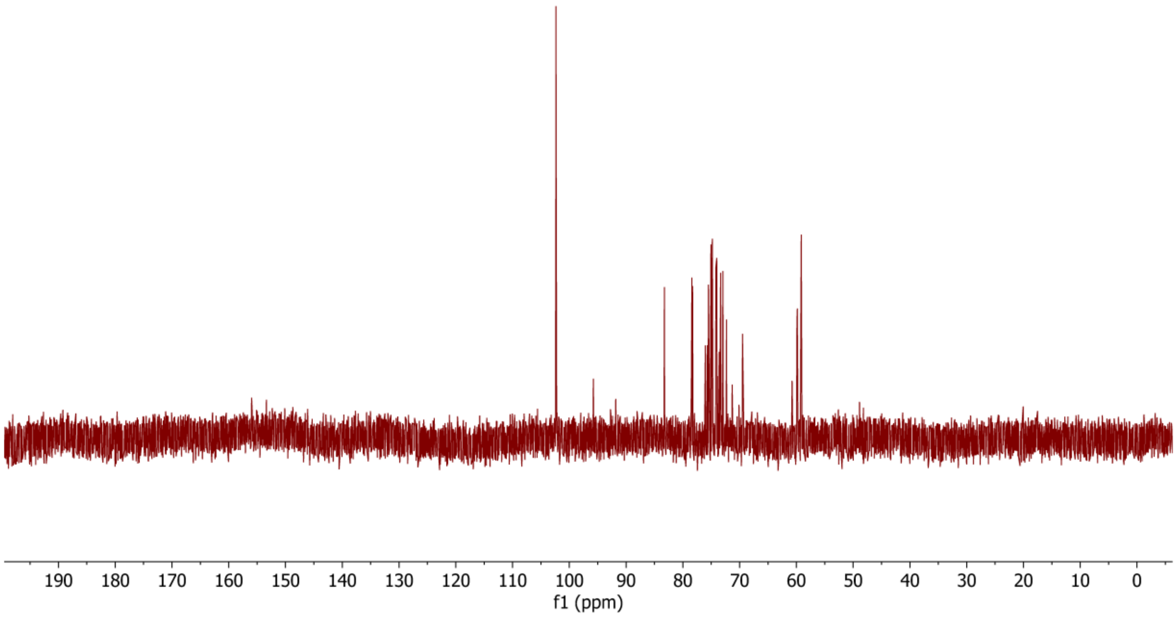

HSQC NMR of ABAABAABAABA-OH (D_2O)

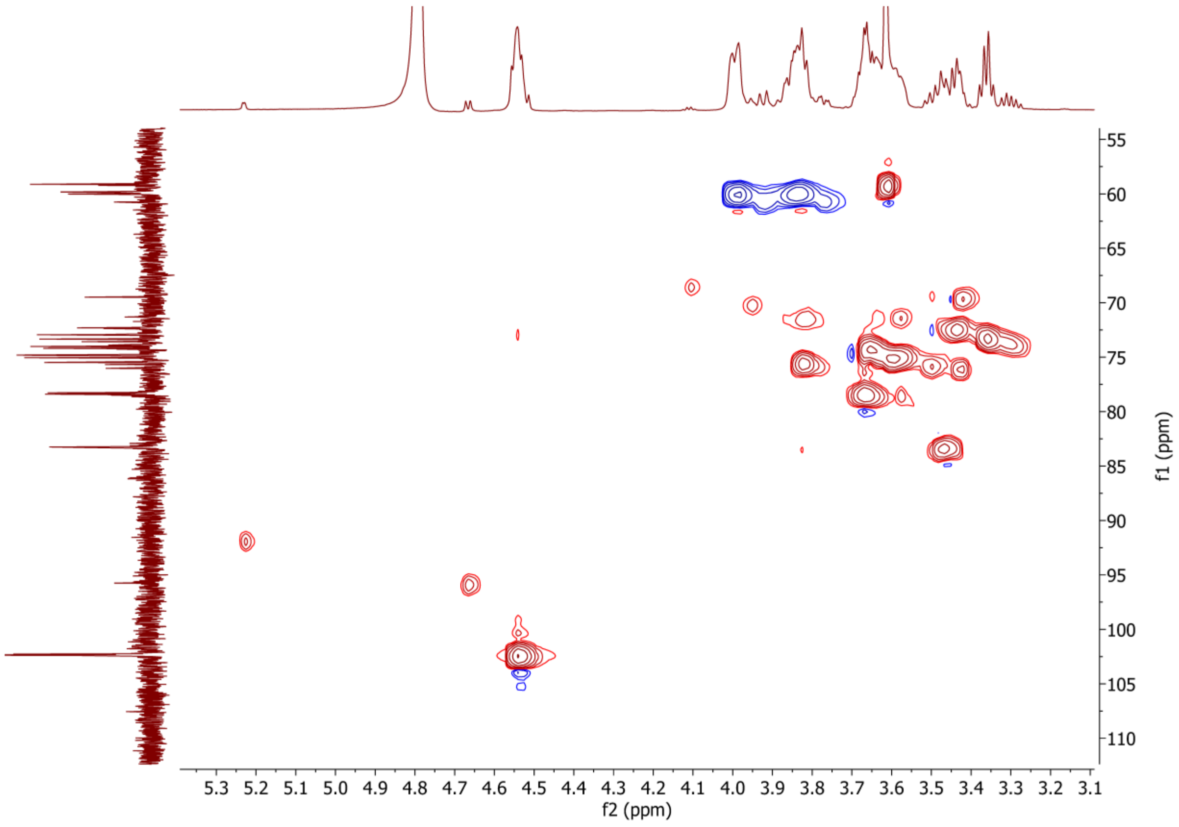

3.5.2.5. *Synthesis of ABACABABACAB-OH*

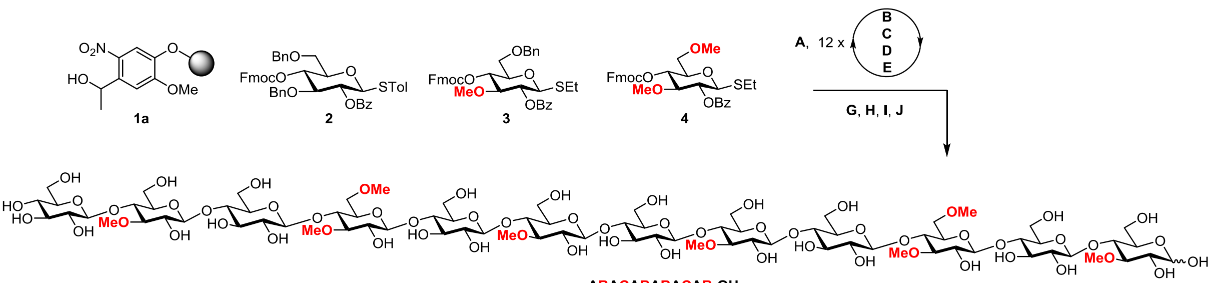

ABACABABACAB-OH

	Module	Conditions
	**A**: Resin Preparation for Synthesis	
	**B**: Acidic Wash with TMSOTf Solution	
	**C**: Thioglycoside Glycosylation	**3**, 6.5 equiv.(-20°C for 5 min, 0°C for 20 min)
	**D**: Capping	
	**E**: Fmoc Deprotection	
	**B**: Acidic Wash with TMSOTf Solution	
	**C**: Thioglycoside Glycosylation	**2**, 6.5 equiv.(-20°C for 5 min, 0°C for 20 min)
	**D**: Capping	
	**E**: Fmoc Deprotection	
	**B**: Acidic Wash with TMSOTf Solution	
	**C**: Thioglycoside Glycosylation	**4**, 6.5 equiv.(-20°C for 5 min, 0°C for 20 min)
	**D**: Capping	
	**E**: Fmoc Deprotection	
	**B**: Acidic Wash with TMSOTf Solution	
	**C**: Thioglycoside Glycosylation	**2**, 6.5 equiv.(-20°C for 5 min, 0°C for 20 min)
**2**	**D**: Capping	
	**E**: Fmoc Deprotection	
	**B**: Acidic Wash with TMSOTf Solution	
	**C**: Thioglycoside Glycosylation	**3**, 6.5 equiv.(-20°C for 5 min, 0°C for 20 min)
	**D**: Capping	
	**E**: Fmoc Deprotection	
	**B**: Acidic Wash with TMSOTf Solution	
	**C**: Thioglycoside Glycosylation	**2**, 6.5 equiv.(-20°C for 5 min, 0°C for 20 min)
	**D**: Capping	
	**E**: Fmoc Deprotection	
	**F**: On-resin Methanolysis	
	**G**: Cleavage from Solid Support	
	**I**: Hydrogenolysis	
	**J**: Purification	**Method M**

Automated synthesis, global deprotection, and purification afforded **ABACABABACAB-OH** as white solid (3.2 mg, 12% overall yield).

Analytical data for **ABACABABACAB-OH**: ^{1}H NMR (600 MHz, D_2O) δ 5.23 (d, *J* = 3.5 Hz, 0.37 H, α-H1), 4.67 (d, *J* = 7.9 Hz, 0.63H, β-H1), 4.59 – 4.51 (m, 9H), 4.46 (d, *J* = 7.9 Hz, 2H), 4.01 (d, *J* = 11.9 Hz, 7H), 3.98 – 3.88 (m, 5H), 3.88 – 3.83 (m, 11H), 3.82 (s, 6H), 3.79 – 3.75 (m, 3H), 3.71 (d, *J* = 12.7 Hz, 3H), 3.67 (t, *J* = 8.8 Hz, 10H), 3.64 – 3.60 (m, 18H), 3.59 (d, *J* = 11.1 Hz, 7H), 3.54 – 3.43 (m, 13H), 3.42 (s, 6H), 3.39 – 3.35 (m, 5H), 3.32 (dd, *J* = 9.4, 7.9 Hz, 2H); ^{13}C NMR (151 MHz, d_2o) δ 102.33, 102.30, 102.27, 102.24, 102.24, 102.21, 102.18, 102.18, 95.72, 83.46, 83.24, 83.19, 83.19, 83.02, 82.51, 78.37, 78.35, 75.96, 75.61, 75.43, 75.37, 75.31, 74.99, 74.81, 74.69, 74.17, 73.51, 73.46, 73.27, 73.24, 73.23, 73.21, 73.16, 72.32, 72.26, 72.11, 69.92, 69.42, 60.00, 59.98, 59.97, 59.78, 59.78, 59.76, 59.44, 59.17, 59.07, 59.03, 58.32; $[\alpha]_D^{20}$ +12.15 (c 0.2, H_2O); IR (neat) ν_{max} = 3378, 2928, 1066 cm^{-1}; m/z (HRMS+) 2097.759 $[M + Na]^+$ ($C_{80}H_{138}NaO_{61}$ requires 2097.759).

RP-HPLC of ABACABABACAB-OH (ELSD trace, Method C, t_R = 21.0, 21.3 min)

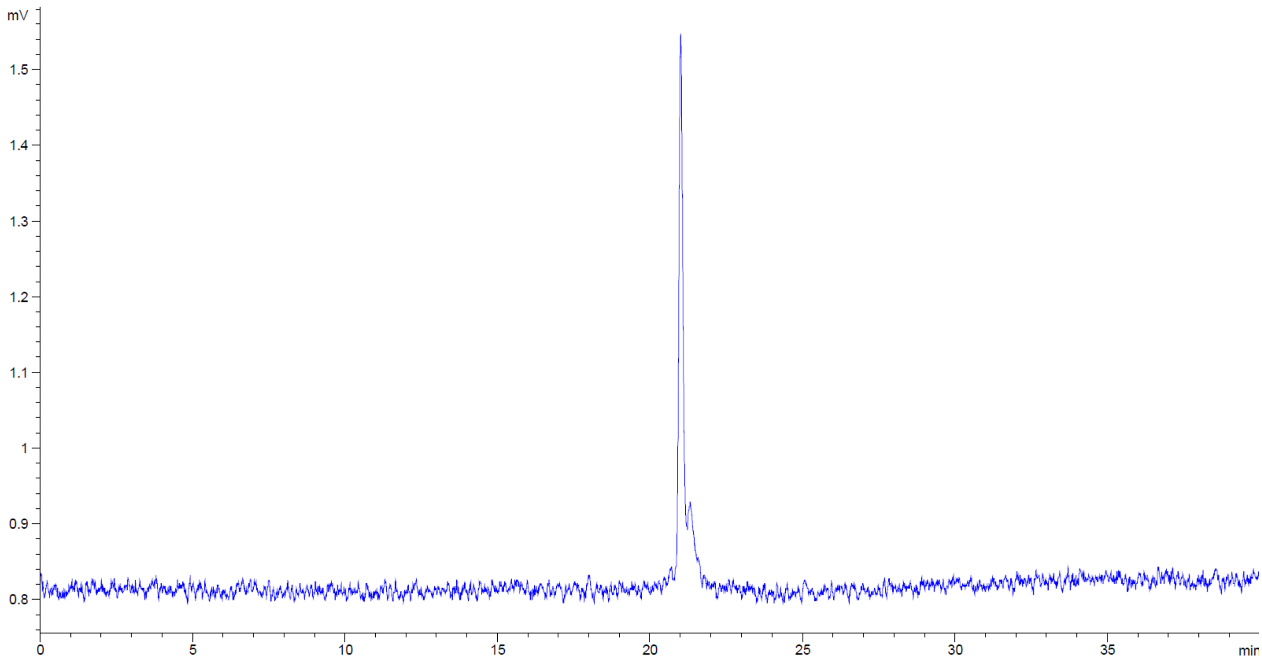

^{1}H NMR of ABACABABACAB-OH (600 MHz, D_2O)

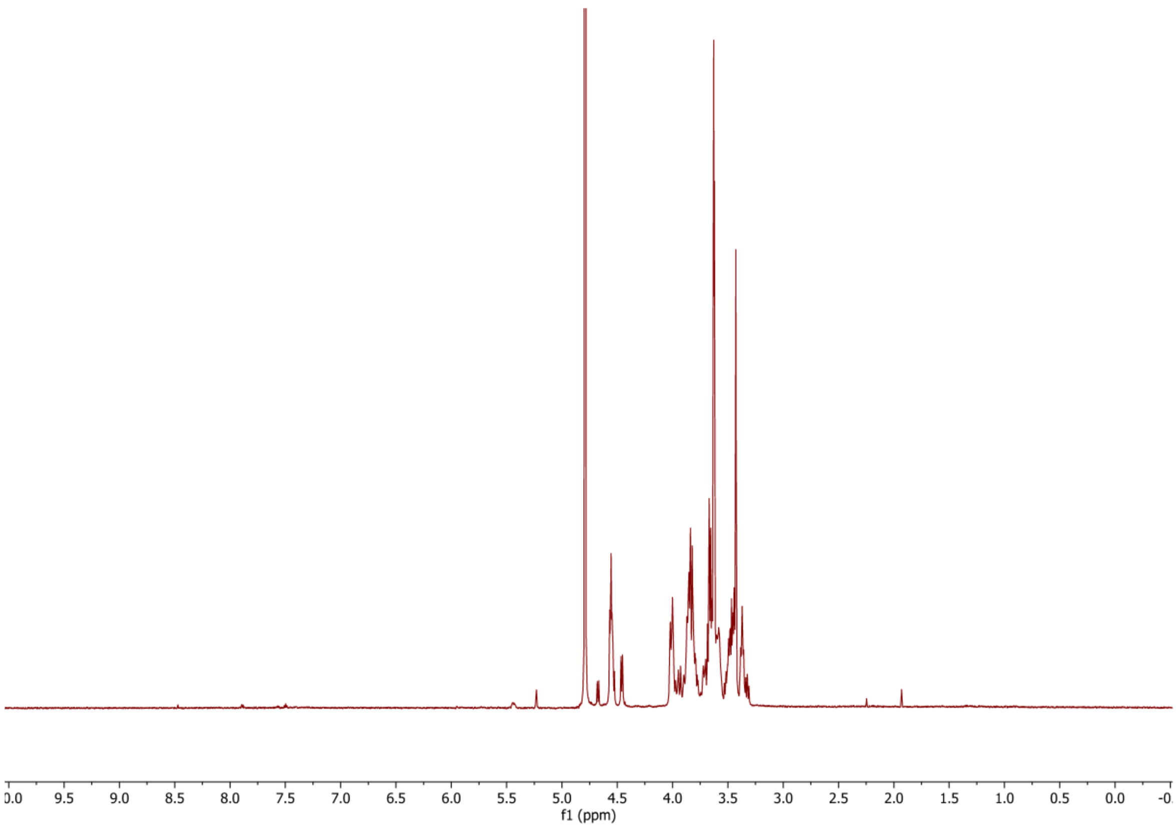

^{13}C NMR of ABACABABACAB-OH (151 MHz, D_2O)

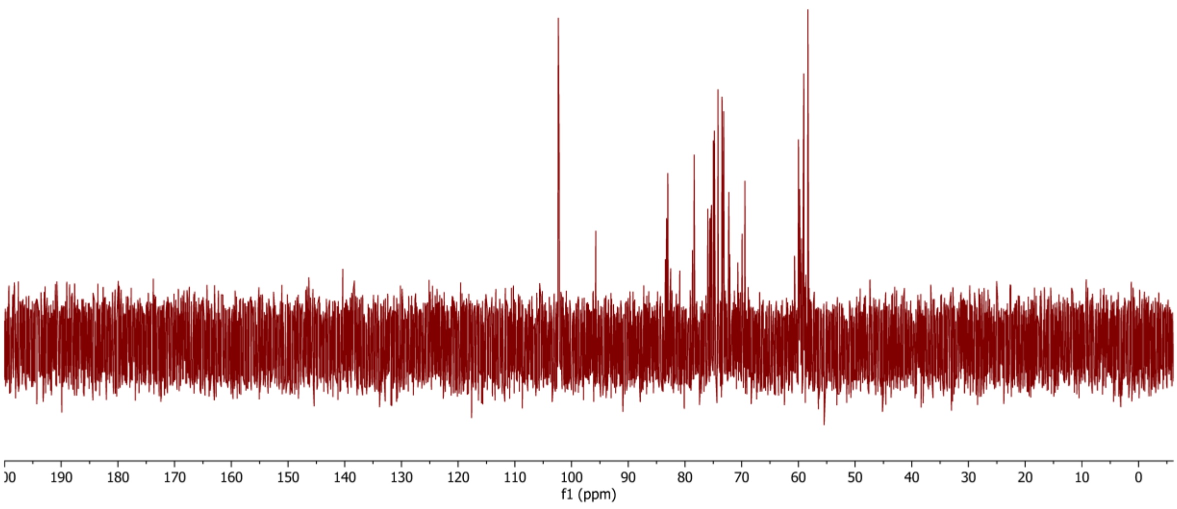

HSQC NMR of ABACABABACAB-OH (D_2O)

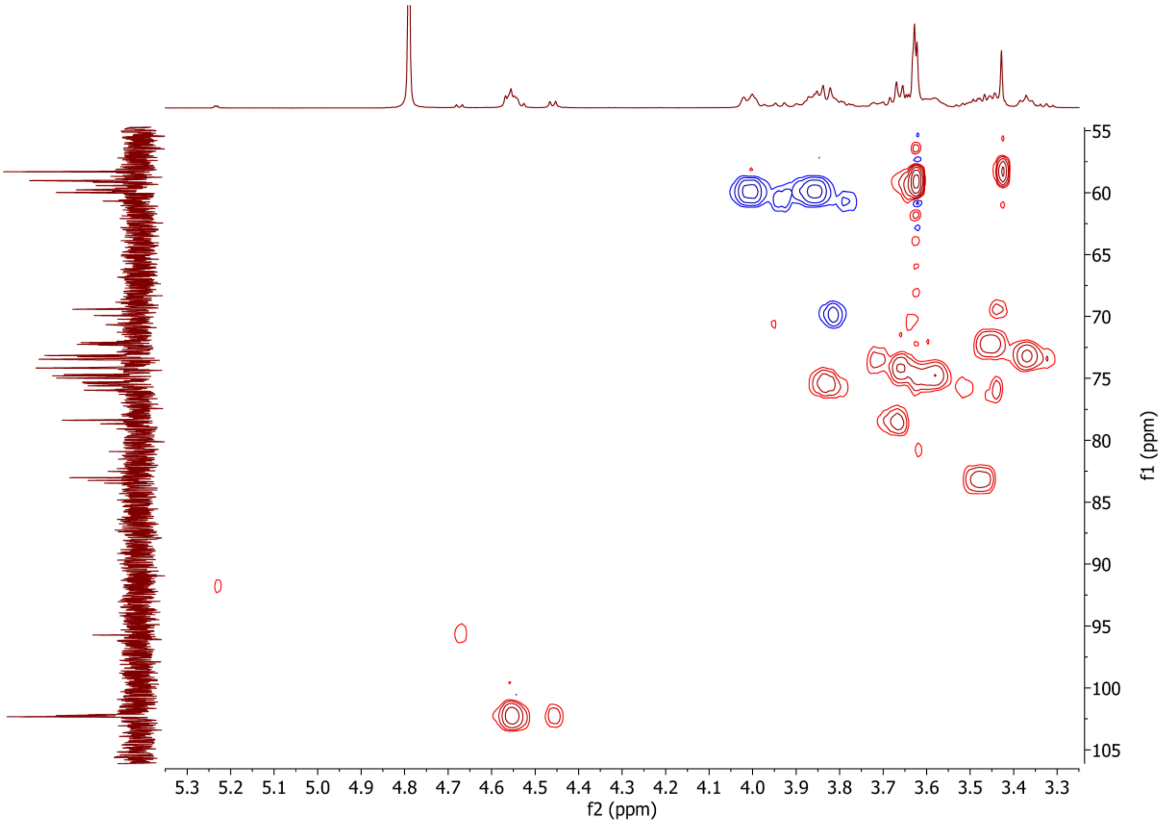

3.5.2.6. *Synthesis of ADAADAADAAD-OH*

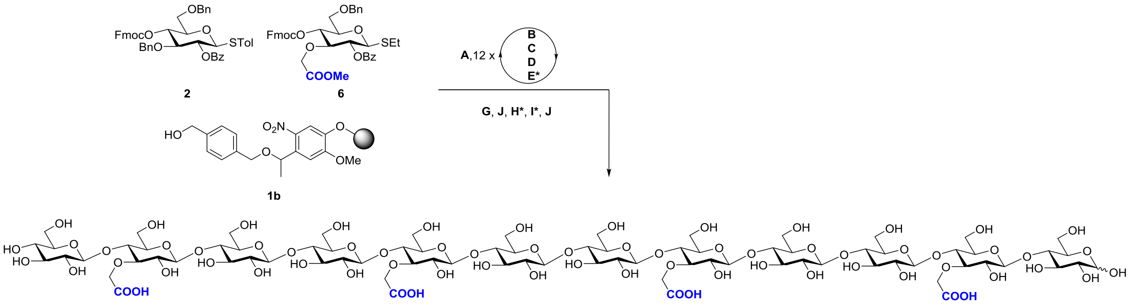

ADAADAADAADA-OH

	Module	Conditions
	**A**: Resin Preparation for Synthesis	
	**B**: Acidic Wash with TMSOTf Solution	
	**C**: Thioglycoside Glycosylation	**2**, 6.5 equiv.(-20°C for 5 min, 0°C for 20 min)
	**D**: Capping	
	**E***: Fmoc Deprotection (TEA)	
	**B**: Acidic Wash with TMSOTf Solution	
	**C**: Thioglycoside Glycosylation	**6**, 6.5 equiv.(-20°C for 5 min, 0°C for 20 min)
**4**	**D**: Capping	
	**E***: Fmoc Deprotection (TEA)	
	**B**: Acidic Wash with TMSOTf Solution	
	**C**: Thioglycoside Glycosylation	**2**, 6.5 equiv.(-20°C for 5 min, 0°C for 20 min)
	**D**: Capping	
	**E***: Fmoc Deprotection (TEA)	
	**G**: Cleavage from Solid Support	
	**J**: Purification	**Method N and Method B**
	**H***: Solution-phase Methanolysis	
	**I***: Hydrogenolysis with balloon	
	**J**: Purification	**Method D**

Automated synthesis, global deprotection, and purification afforded **ADAADAADAADA-OH** as white solid (4.8 mg, 17 % overall yield).

Analytical data for **ADAADAADAADA-OH**: ^{1}H NMR (600 MHz, D_2O) δ 5.24 (d, *J* = 3.7 Hz, 0.31 H, α-H1), 4.68 (d, *J* = 8.0 Hz, 0.69H, β-H1), 4.64 – 4.50 (m, 9H), 4.43 (d, *J* = 16.4 Hz, 4H), 4.07 – 3.93 (m, 12H), 3.93 – 3.77 (m, 19H), 3.76 – 3.54 (m, 30H), 3.53 – 3.45 (m, 6H), 3.45 – 3.25 (m, 11H); ^{13}C NMR (151 MHz, D_2O) δ 175.27, 166.10, 102.34, 102.29, 102.24, 102.14, 95.69 (β-C1), 91.75 (α-C1), 82.92, 82.89, 78.50, 78.43, 78.33, 78.11, 76.13, 76.04, 75.96, 75.48, 74.85, 74.72, 74.16, 74.00, 73.90, 73.81, 73.57, 73.31, 72.87, 72.47, 71.21, 71.15, 70.03, 69.66, 69.49, 60.82, 60.67, 60.08, 59.76, 59.67; $[\alpha]_D^{20}$ +18.95 (c 0.2, H_2O); IR (neat) ν_{max} = 3333, 2927, 1723, 1020 cm^{-1}; m/z (HRMS$^+$) 2217.641 $[M + Na]^+$ ($C_{80}H_{130}O_{69}Na^+$ requires 2217.655).

RP-HPLC of ADAADAADAADA-OH (ELSD trace, Method C, t_R = 18.5 min)

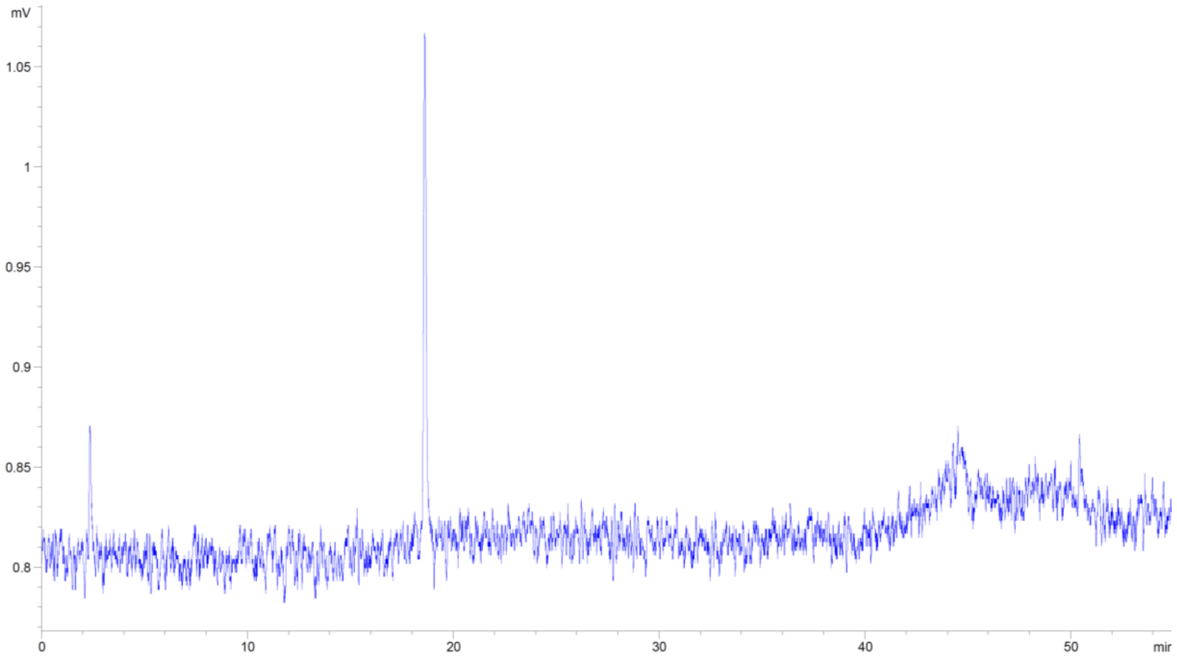

^{1}H NMR of ADAADAADAADA-OH (600 MHz, D_2O)

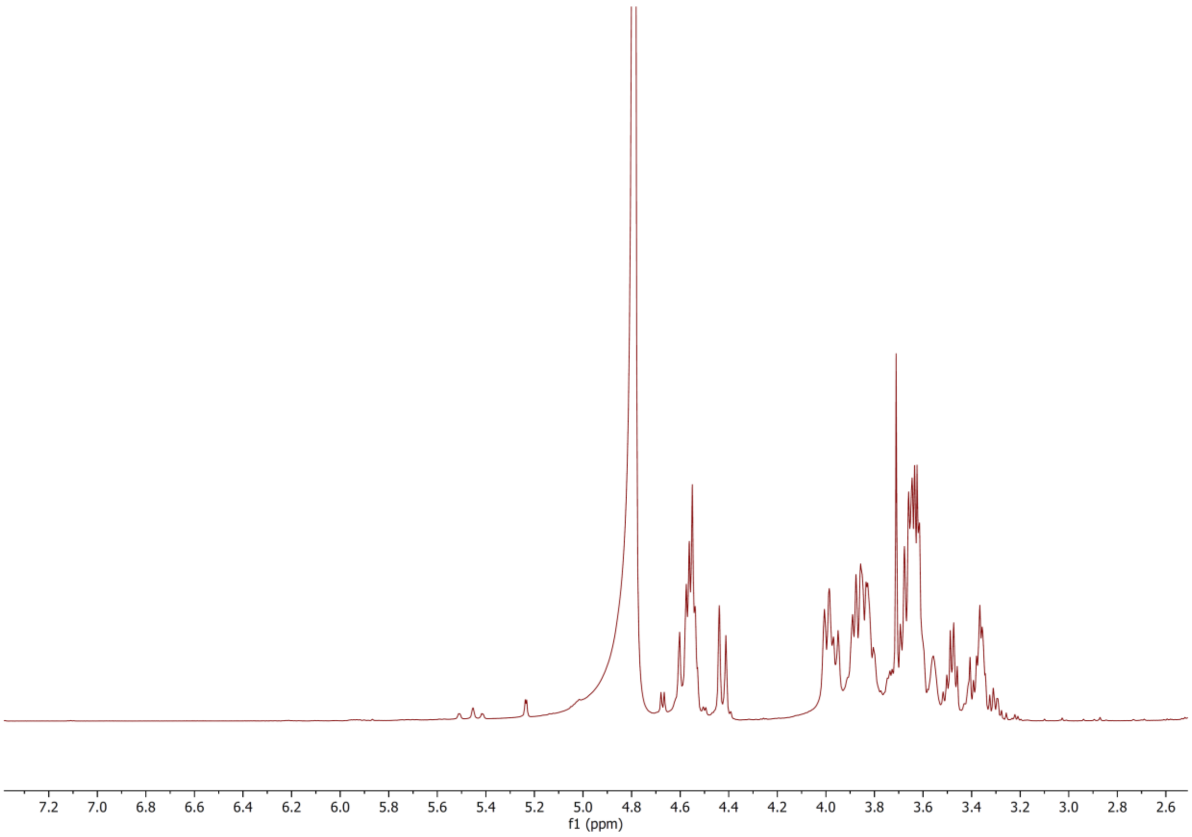

^{13}C NMR of ADAADAADAADA-OH (151 MHz, D_2O)

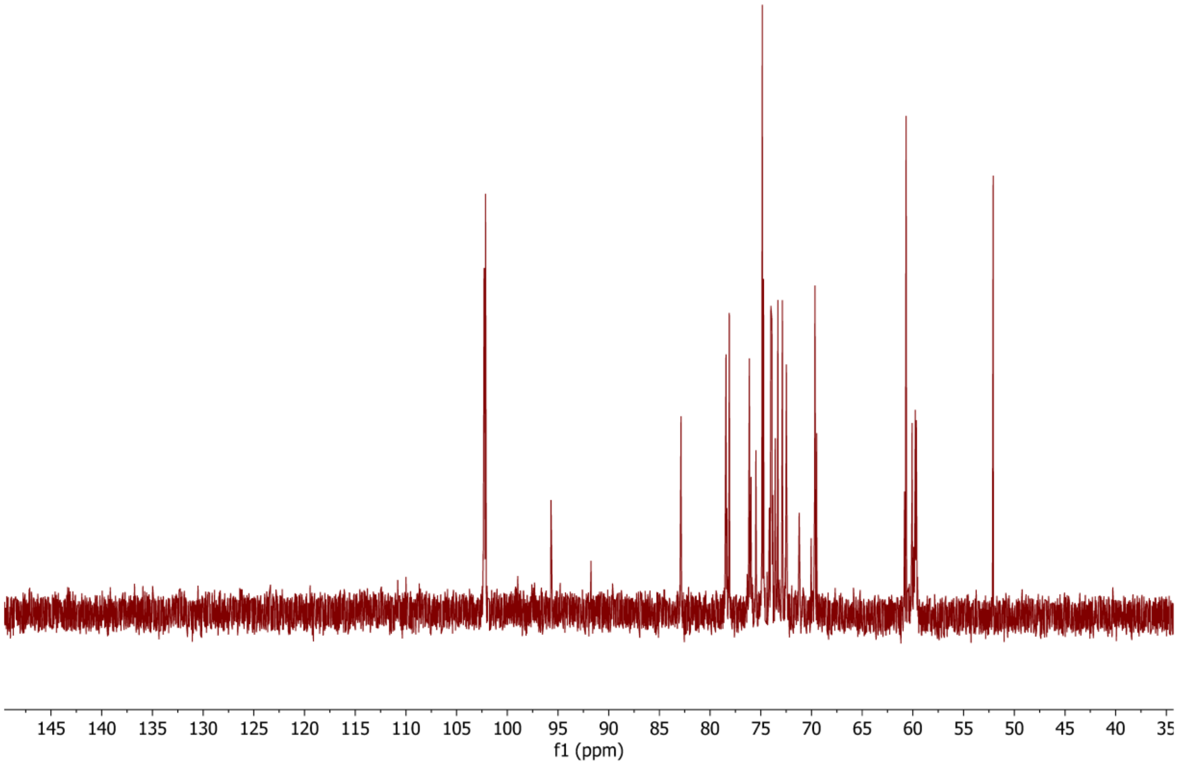

HSQC NMR of ADAADAADAADA-OH (D_2O)

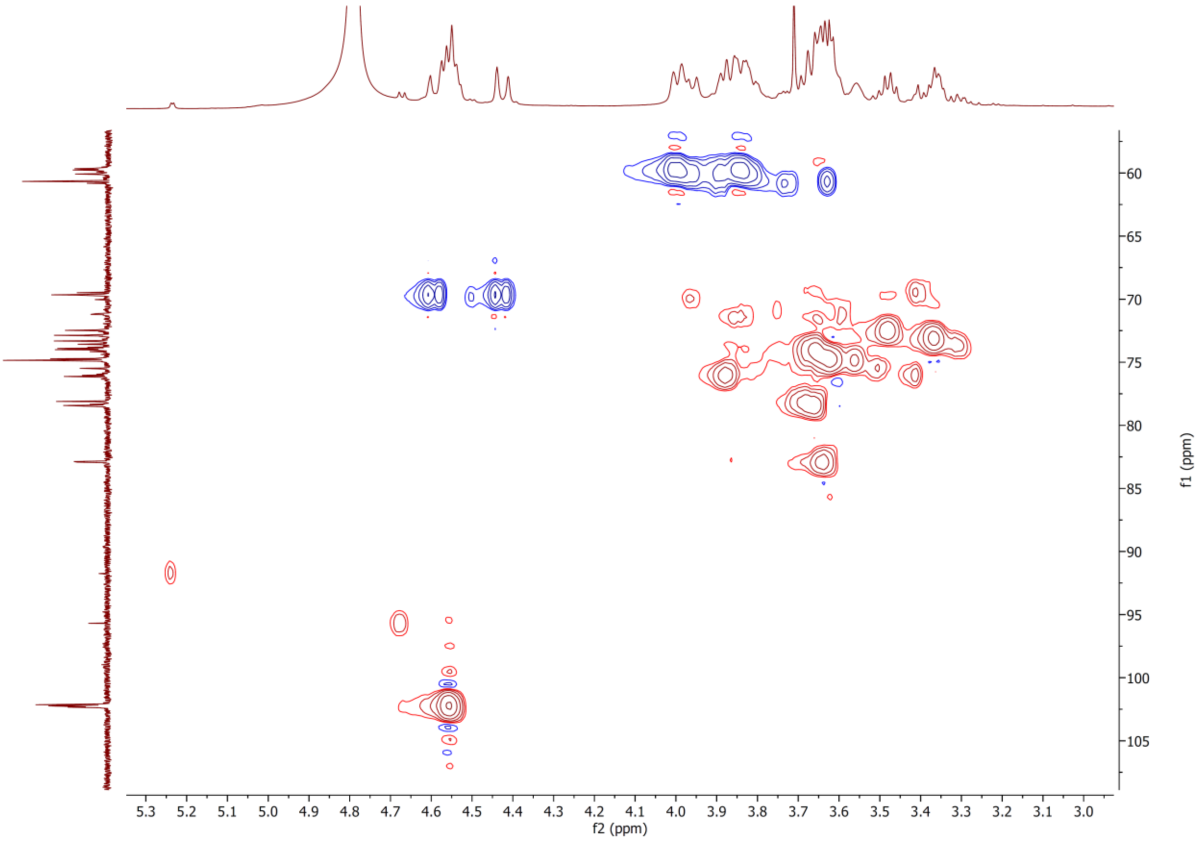

4. Synthesis and NMR analysis of 3-*O*-methyl-D-glucopyranoside S1

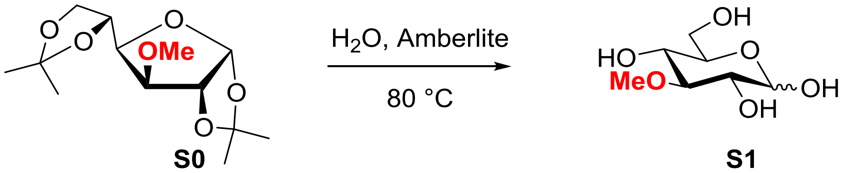

Compound **S0** was prepared according to previous literature.[2]

1,2:5,6-di-*O*-isopropylidene-3-*O*-methyl-α-D-ribohexofuranose **S0** (56 g, 204 mmol) was suspended in 400 mL H_2O. 75 g Amberlite IR-120 (H^+ form) was added. The reaction mixture was heated to 80 °C and stirred vigorously for 16 h. The reaction was cooled to rt and the solid material was filtered off. The reaction was concentrated under reduced pressure. The residue was suspended in ACN and then the solvent was removed under reduced pressure to give **S1** as a pale yellow powder (39.6 g, quantitative). ^{1}H NMR (600 MHz, D_2O) δ 5.23 (d, *J* = 3.7 Hz, 0.4H, α-H1), 4.66 (d, *J* = 8.0 Hz, 0.6H, β-H1), 3.90 (dd, *J* = 12.4, 2.0 Hz, 0.6H, β-H6), 3.84 (dd, *J* = 12.2, 2.1 Hz, 0.4H, α-H6), 3.87 – 3.87 (m, 0.4H, α-H5), 3.77 (dd, *J* = 12.4, 5.4 Hz, 0.4H, α-H6'), 3.73 (dd, *J* = 12.3, 5.4 Hz, 0.4H, β-H6'), 3.64 (s, 3H, OMe), 3.62 – 3.57 (m, 0.4H, β-H5), 3.53 – 3.46 (m, 2H), 3.34 – 3.27 (m, 1H); ^{13}C NMR (151 MHz, d_2o) δ 95.79 (β-C1), 92.01 (α-C1), 85.28, 82.65, 75.73, 73.41, 71.41, 70.92, 69.01, 68.89, 60.55, 60.38, 59.98, 59.71; $[\alpha]_D^{20}$ +95.71 (c 1, $CHCl_3$); IR (neat) νmax = 3415, 1089 cm^{-1}; m/z (HRMS+) 217.0687 $[M + Na]^+$ ($C_7H_{14}NaO_6$ requires 217.0683).

^{1}H NMR of S1 (600 MHz, D_2O)

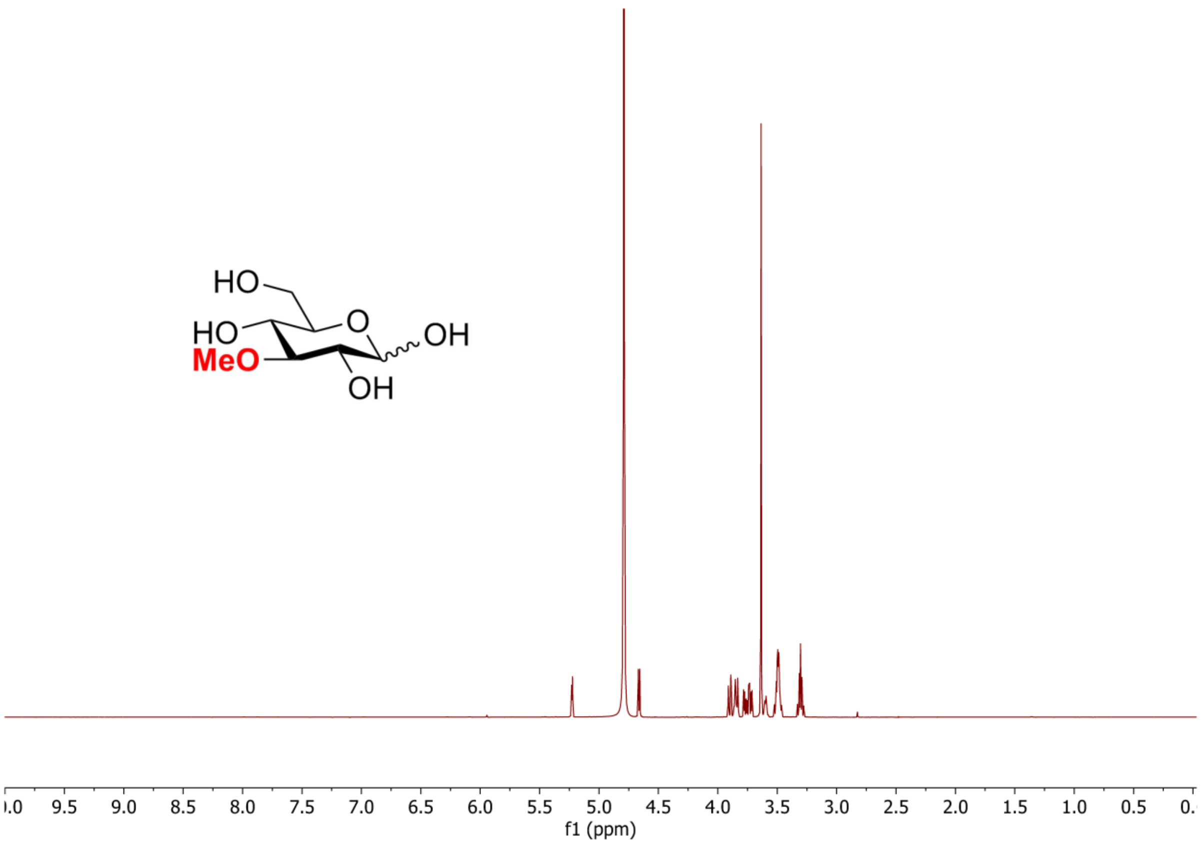

^{13}C NMR of S1 (151 MHz, D_2O)

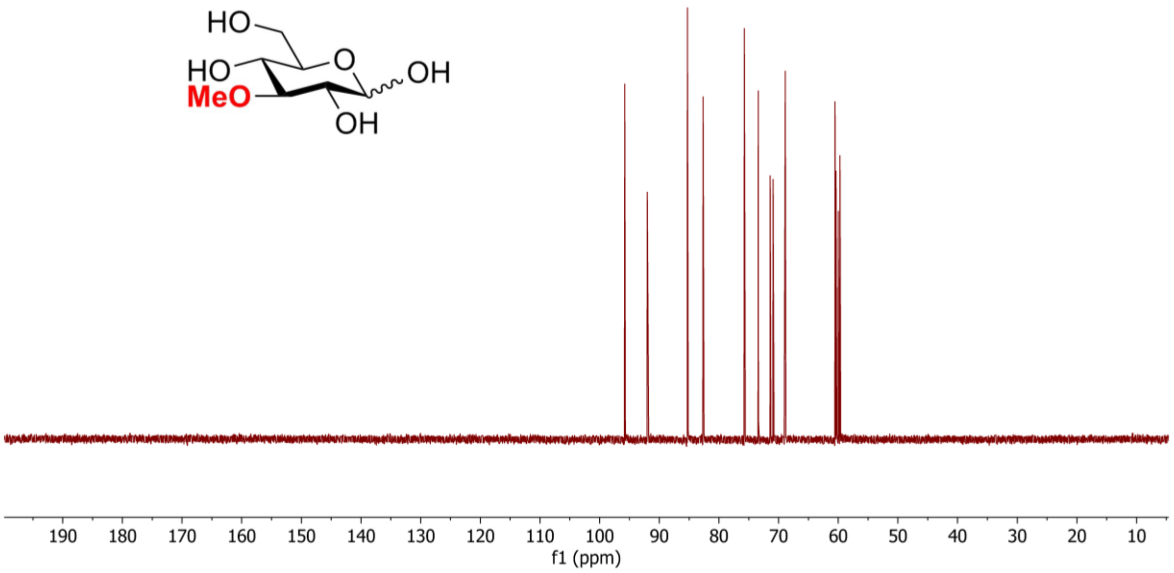

HSQC NMR of S1 (D_2O)

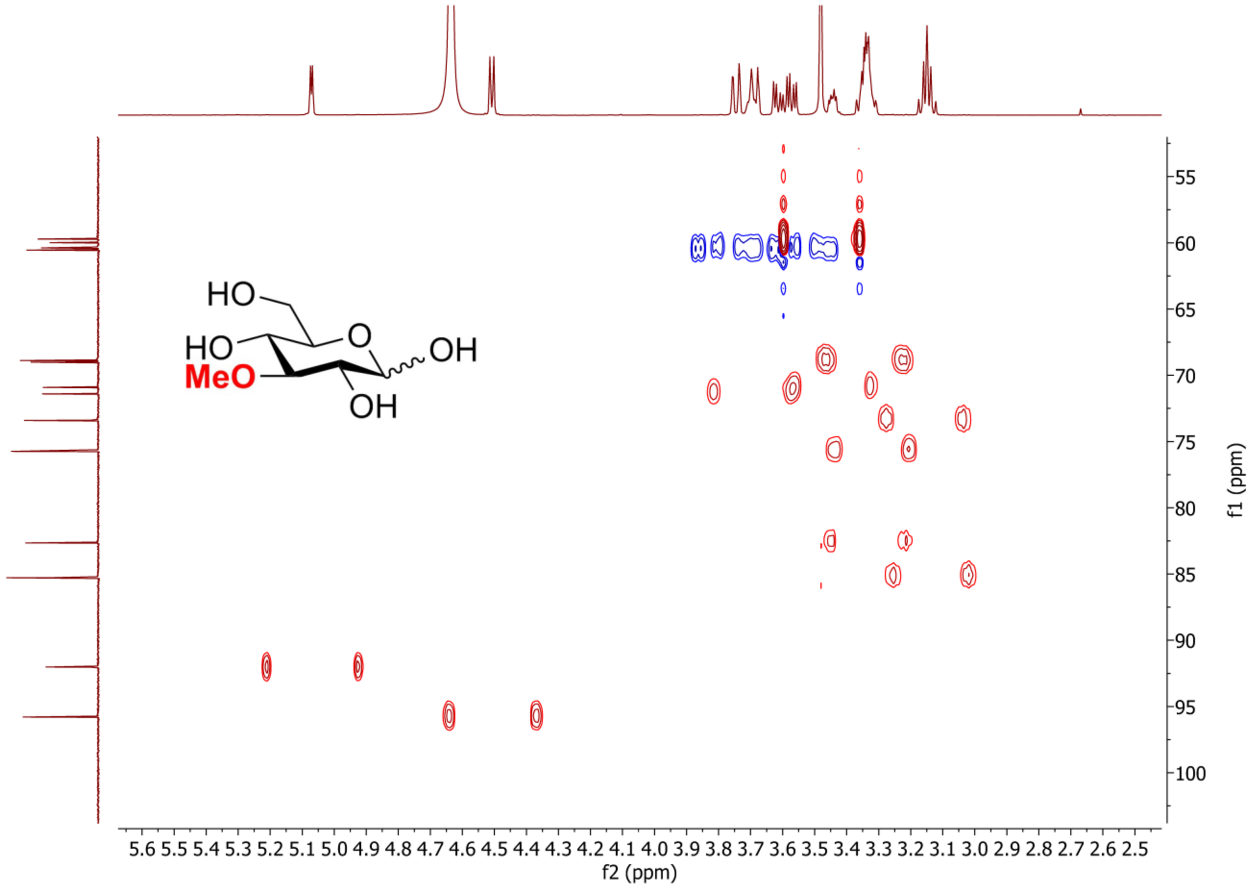

^{1}H NMR of S1 with Coupling Constant (600 MHz, D_2O)

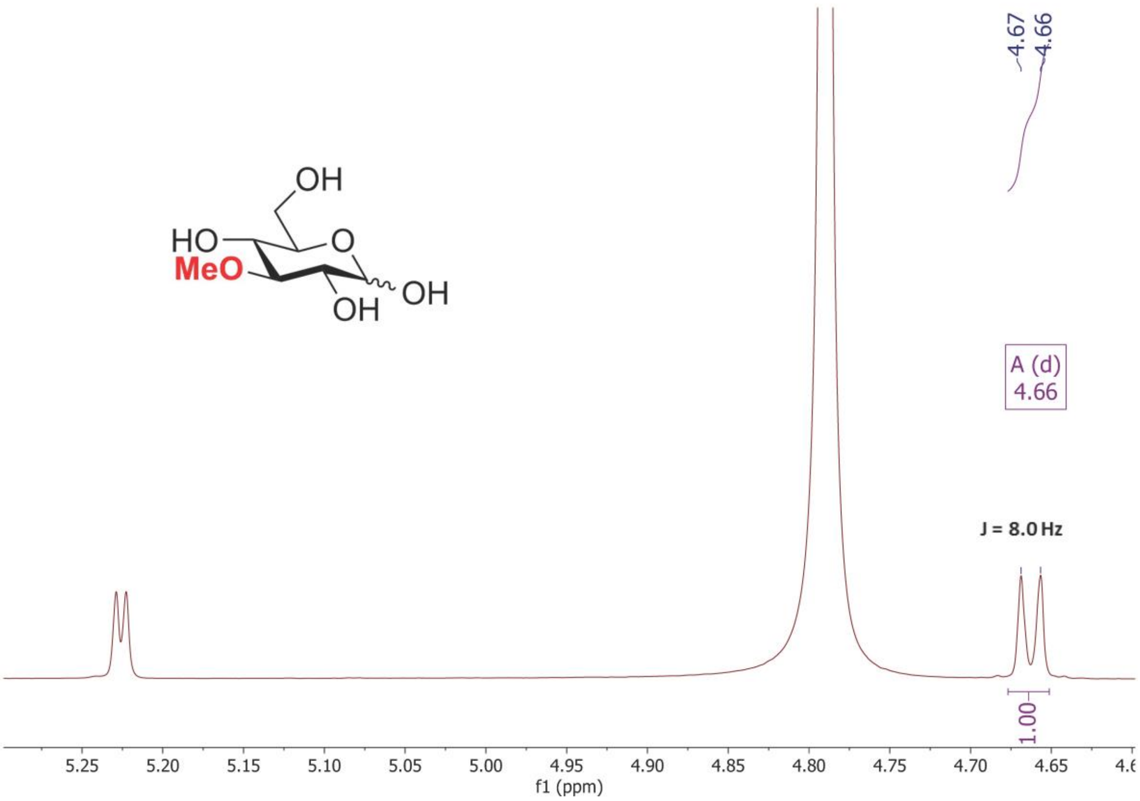

^{1}H NMR of D-glucose with Coupling Constant (600 MHz, D_2O)

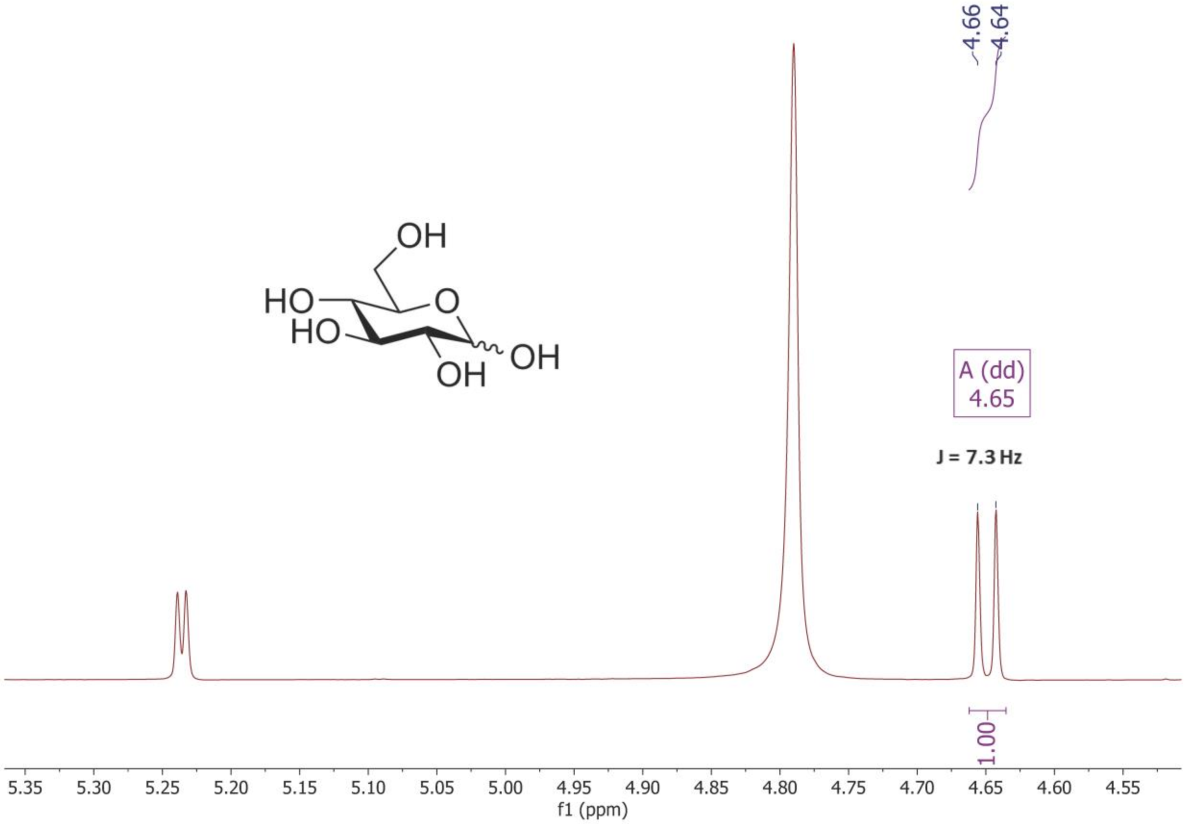

HSQC NMR of S1 with Coupling Constant (D_2O)

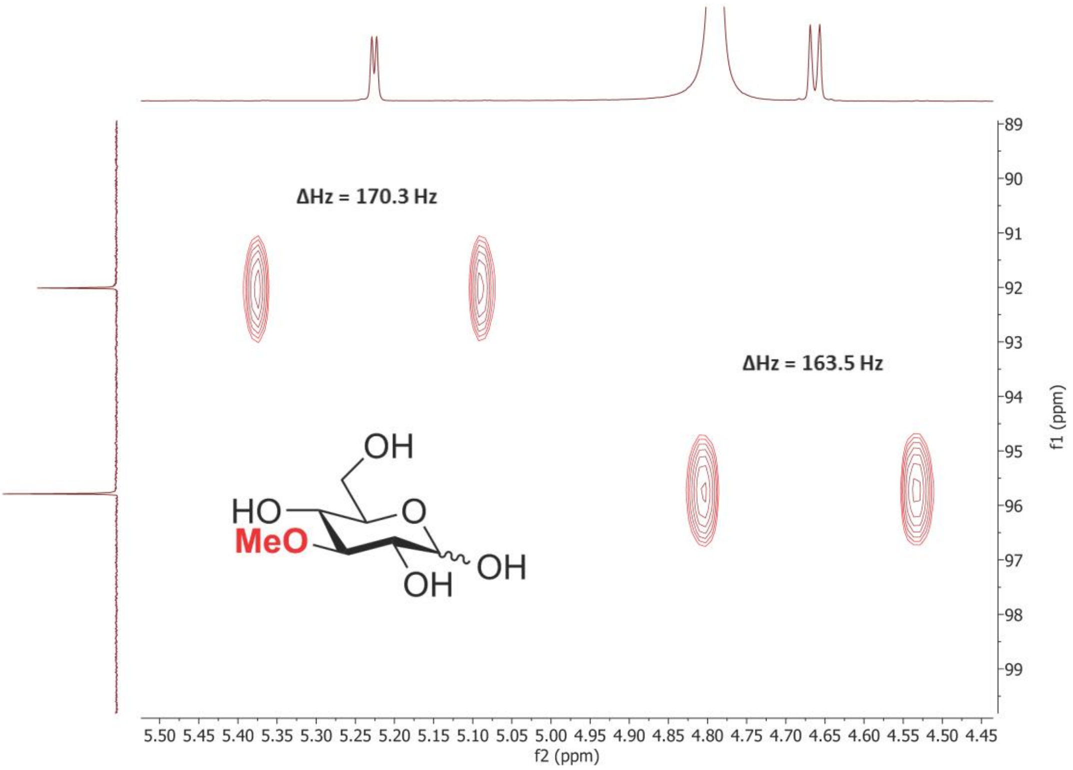

HSQC NMR of D-glucose with Coupling Constant (D_2O)

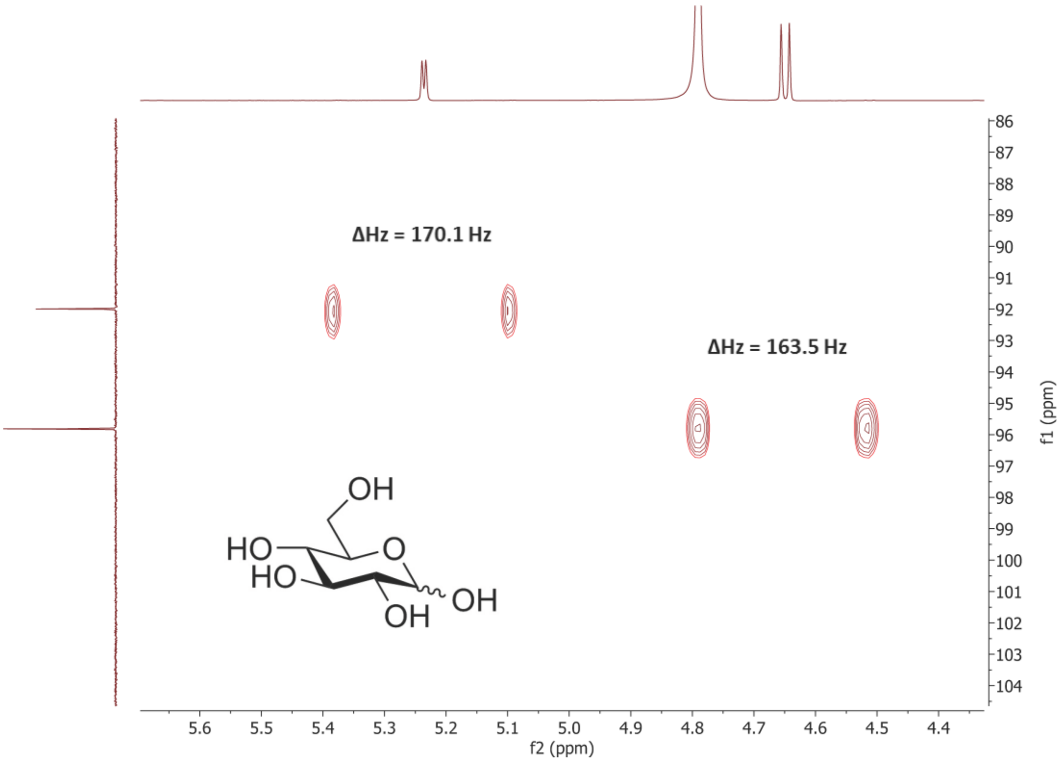

Comparison of the $^{1}J_{C1H1}$ and $^{3}J_{H1H2}$ coupling constants shows no significant difference between D-glucose and the 3-methyl analogue S1, confirming that no "chair flipping" is taking place.

5. Solubility Measurement

The lyophilized oligomer was weighed in a glass vial and water was injected in portions. After each portion, the mixture was bubbled with N_2 through a syringe for 30 seconds. Upon complete disappearance of insoluble matter, the range of solubility was calculated. The water addition was stopped when the solubility was calculated to be less than 1 mg/mL.

Sample	Mass	Last volume before dissolution (μL)	Volume upon dissolution (μL)	Solubility (mg/mL)
A_6	1.0 mg	1000	-	<1
A_3B_3	1.0 mg	-	20	>50
$(ABA)_2$	1.0 mg	-	20	>50
$(AB)_3$	1.0 mg	-	20	>50
ABACAB	1.0 mg	-	20	>50
$(ACA)_2$	1.0 mg	-	20	>50
$(AC)_3$	1.0 mg	-	20	>50
$(AFA)_2$	1.1 mg	-	20	>50
$(FA)_3$	1.0 mg	-	20	>50
$(ADA)_2$	1.0 mg	-	20	>50
$(DA)_3$	1.0 mg	-	20	>50
$(AEA)_2$	1.0 mg	-	20	>50
$(AE)_3$	1.0 mg	-	20	>50
N_6	2.6 mg	150	200	13-17
$(ANA)_2$	1.0 mg	-	20	>50
A_{12}	0.6 mg	600	-	<1
$A_3B_3A_3B_3$	1.0 mg	-	20	>50
$(ABA)_2A_3B_3$	1.0 mg	-	20	>50
$(ABA)_4$	1.0 mg	-	20	>50
$(ABACAB)_2$	1.0 mg	-	20	>50
$(ADA)_4$	1.0 mg	-	20	>50

6. XRD Analysis

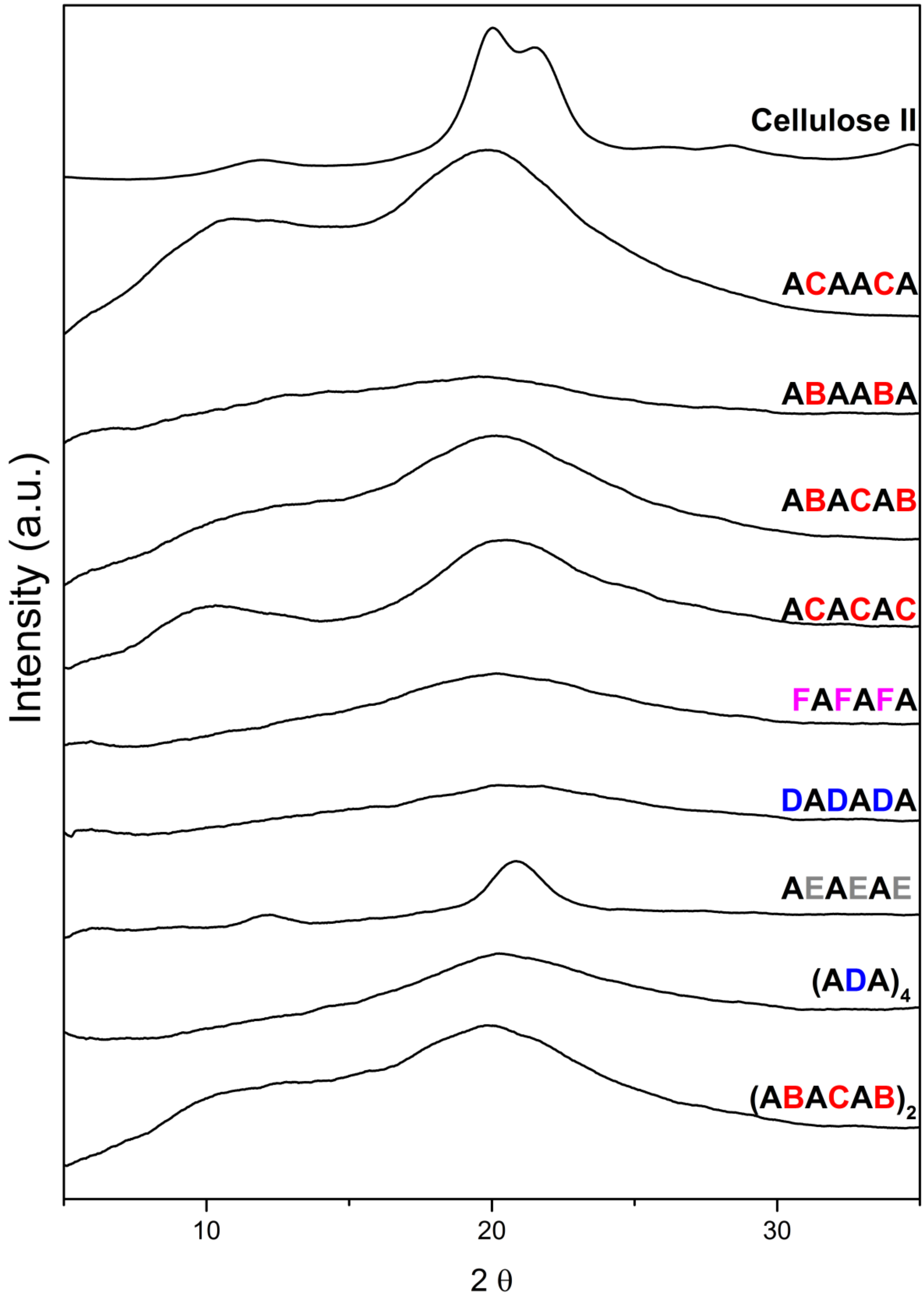

Fig. S1 | XRD data of cellulose analogues.

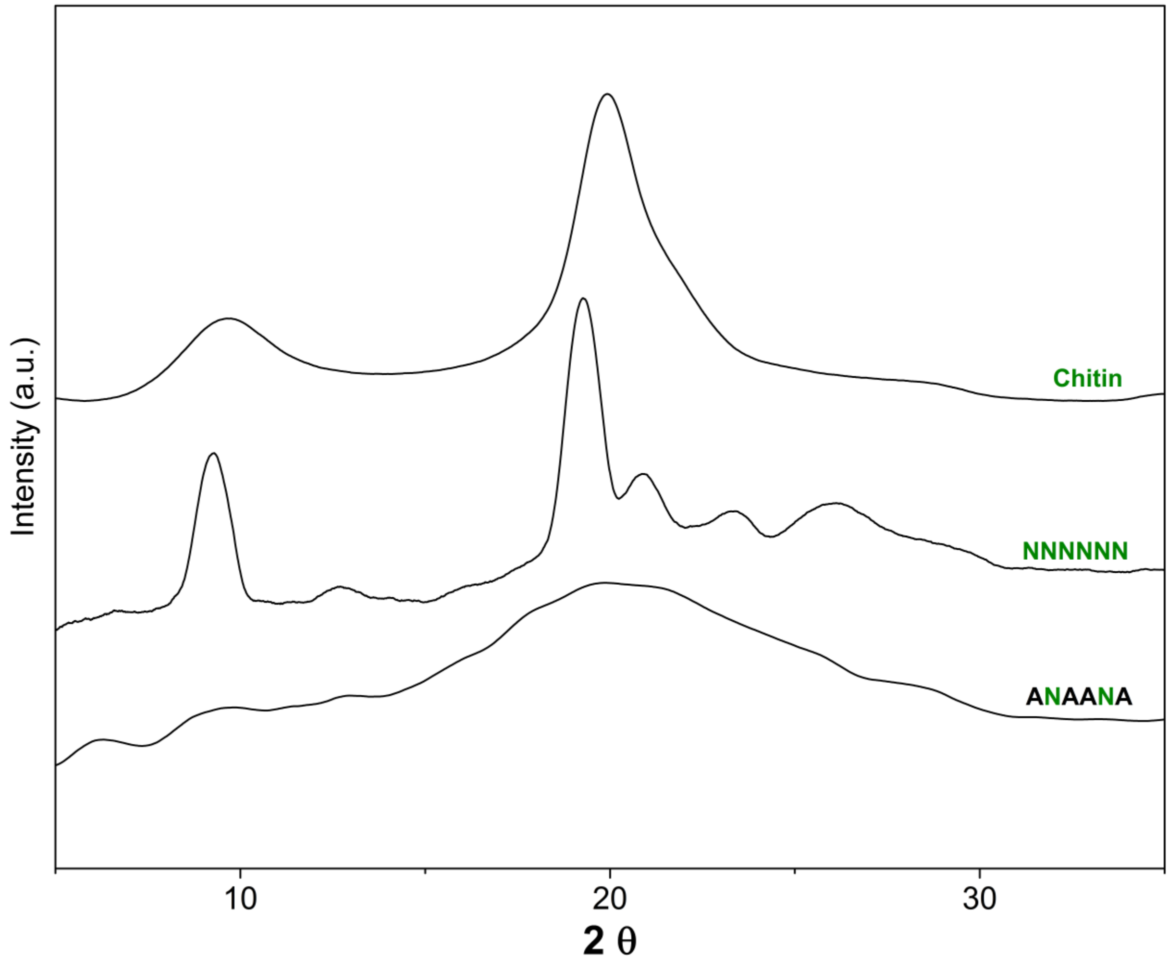

Fig. S2 | Comparison of XRD data of Chitin, NNNNNN, and ANAANA.

7. Molecular Dynamics Simulations

Initial conformations for single hexamer and dodecamer simulations were constructed with the Glycam Carbohydrate builder[8] and tleap. The topology was subsequently converted using the glycam2gmx.plscript[9] and solvated with 2100 TIP5P[10] water molecules using gromacs tools. Concentrated systems were prepared by randomly inserting, 25 hexamer molecules in a simulation box (6 nm x 6 nm x 6 nm) with gmx_insert-molecules. The system was then solvated with 84 TIP5P water molecules per hexamer.

Molecular Dynamics simulations for each system were performed using gromacs 5.1.2.[11] The systems were kept at a constant temperature of 303 K using a Nosé- Hoover thermostat and at constant pressure of 1 bar with the Parrinello-Rahman barostat. Non-bonded interactions were cut-off at 1.4 nm, long range electrostatics were calculated using the particle mesh ewald method.[12] Bonds involving hydrogens were constrained using the LINCS[13] to allow a 2 fs time step algorithm; water molecules were kept rigid with SETTLE.[14]

After energy minimization (steepest descent algorithm) and before the production run, the systems were equilibrated at 300 K for 20 ns in a canonical (NVT) ensemble (constant number of particles, volume and temperature) and subsequently at 300 K and 1 atm for 20 ns in an isothermal-isobaric (NPT) ensemble. A longer equilibration procedure (50ns of npt and 400ns of NVT) was performed for the concentrated experiments.

All hexamers were simulated for 500 ns, all dodecamers for 400ns and concentrated solutions for 1 µs.

For all methylated (A and B) and carboxymethylated (D) monosaccharides dihedral restraints were applied to the dihedral angle C_1-C_2-C_3-C_4, using an equilibrium angle of -47° and a force constant of 20 20 20 (x, y, z ; kJ/(mol rad^2)) , in order to prevent the ”ring flip” artifact.
Radial distribution functions were calculated between the centers of mass of the oligosaccharides by invoking the gmx rdf command with the option (whole_mole_com).

S3

Root-mean-square deviation of atomic positions (**RMSD**)

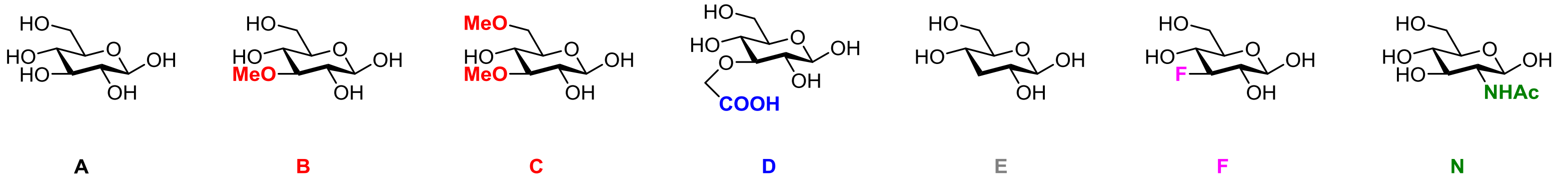

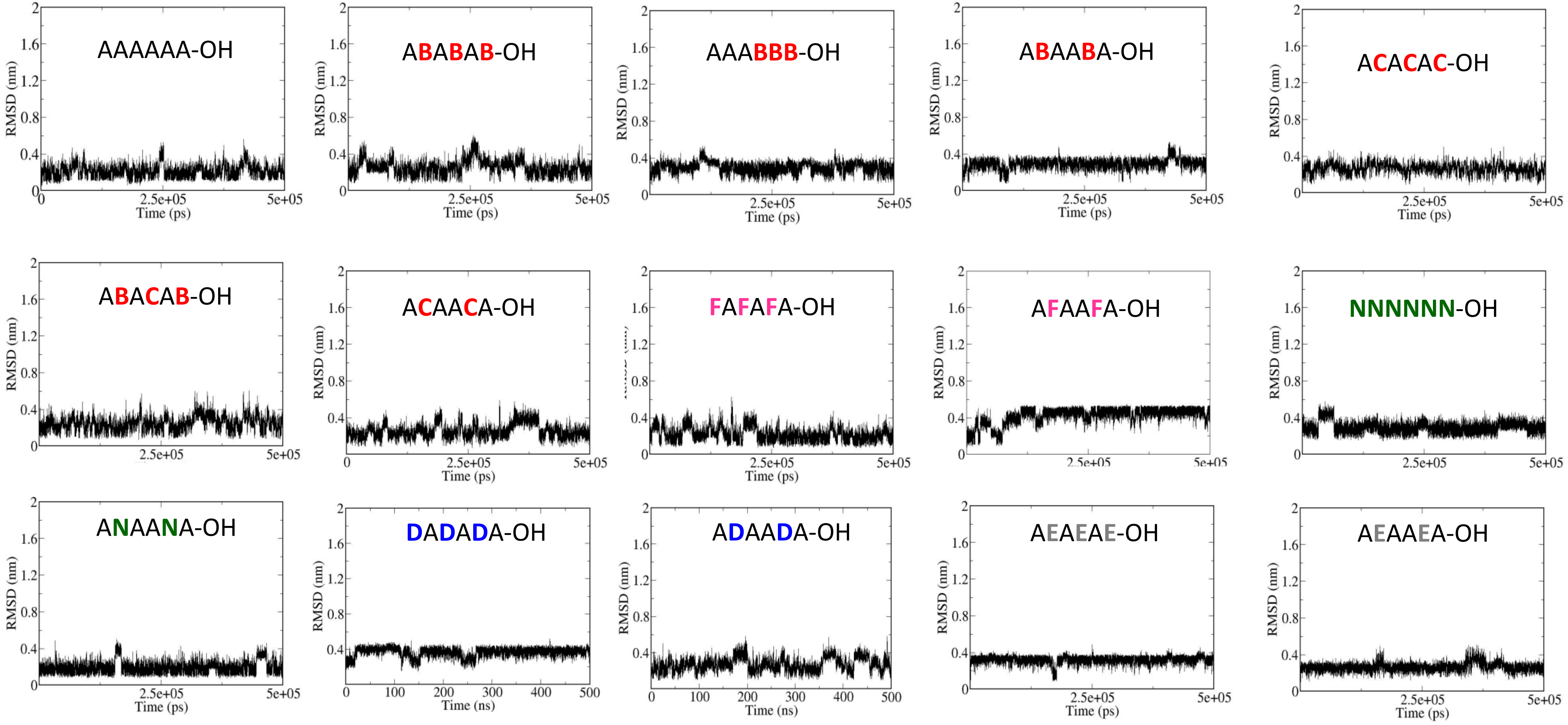

S4

Histograms of radius of gyration (**RoG**)

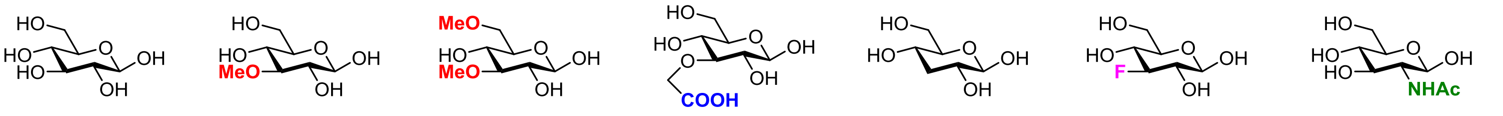

A B C D E F N

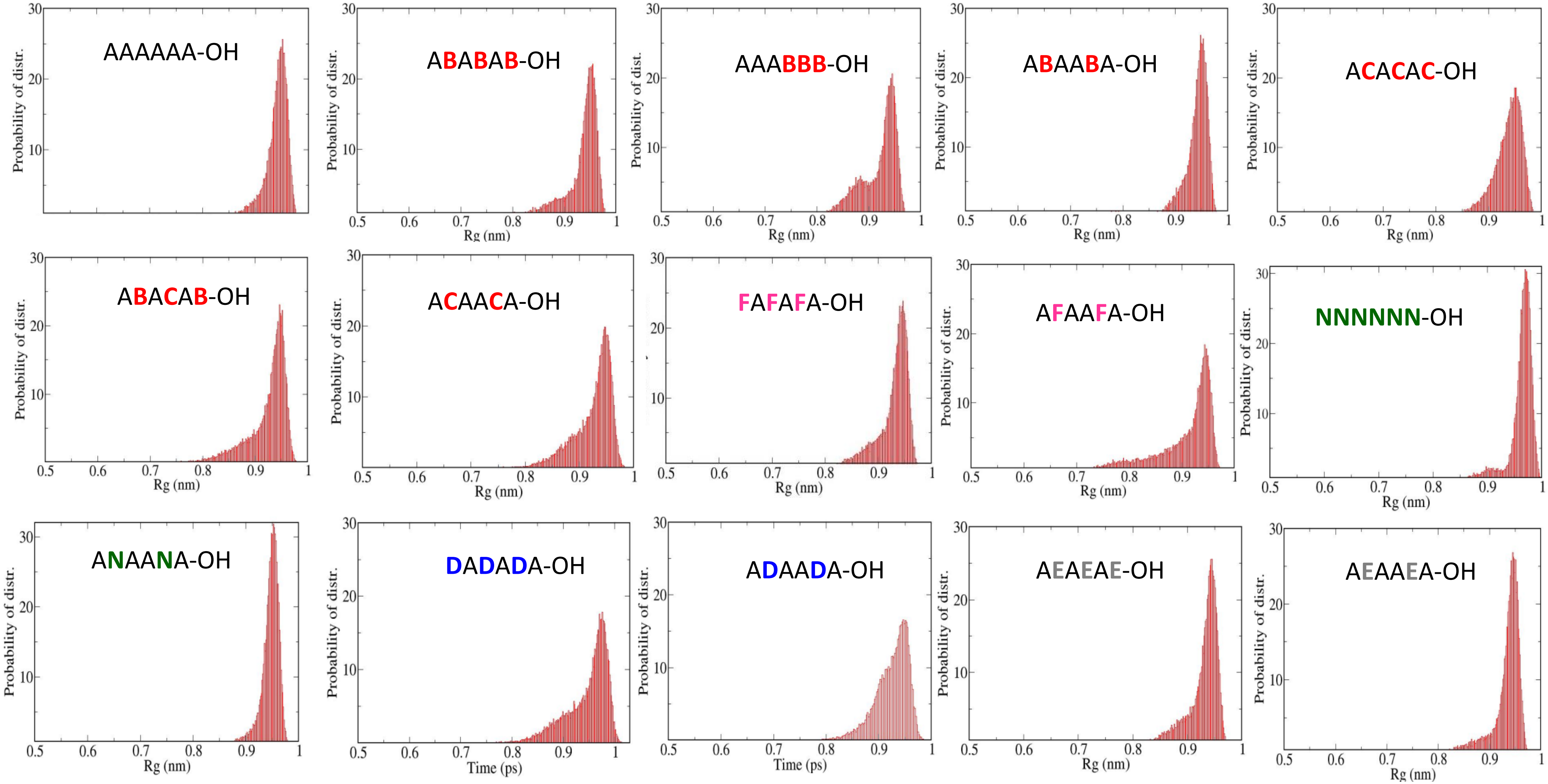

S5

End-to-end distance as a function of time

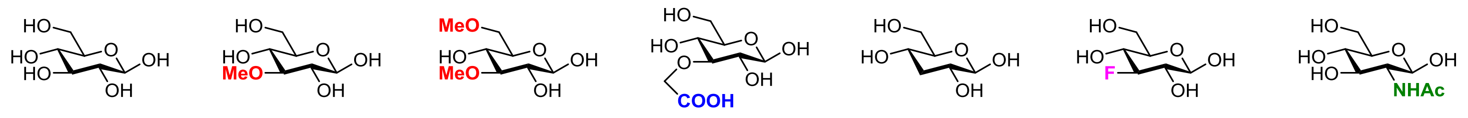

A B C D E F N

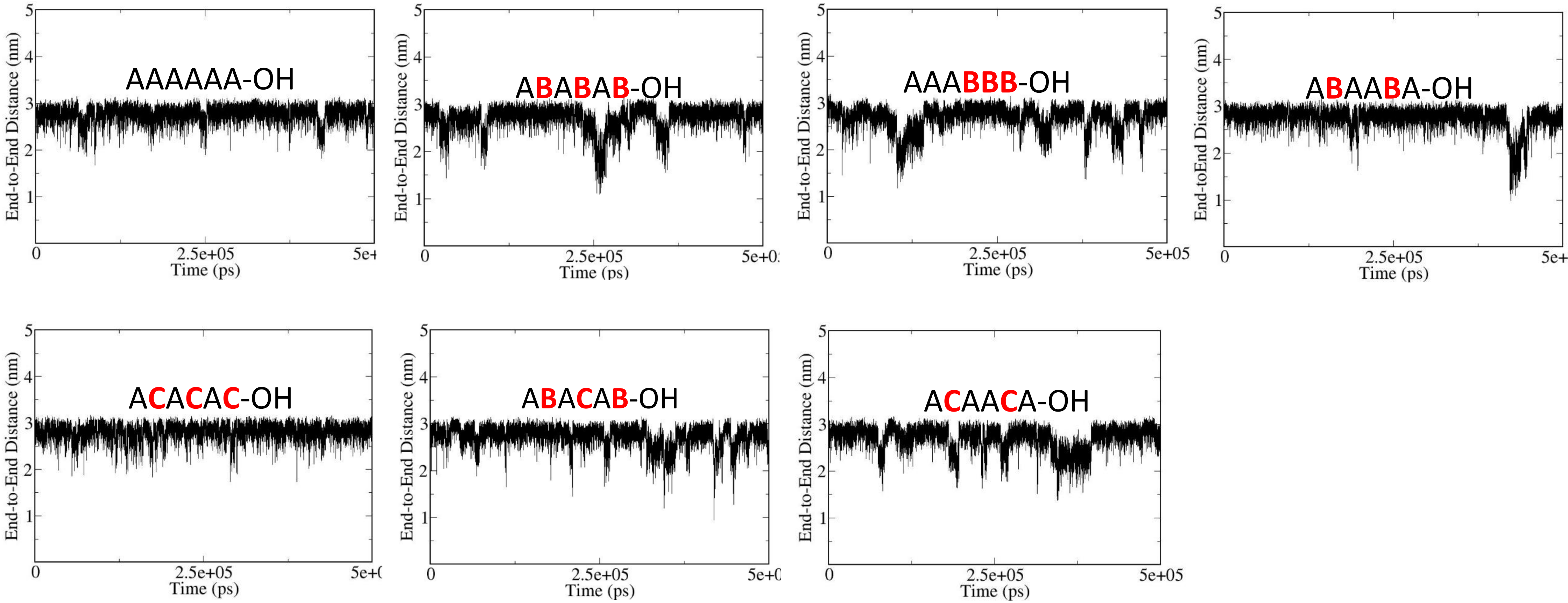

Hexamer	AAAAAA-OH	ABABAB-OH	AAABBB-OH	ABAABA-OH	ACACAC-OH	ABACAB-OH	ACAACA-OH
Average Distance (nm)	2.76	2.71	**2.65**	2.75	2.80	2.73	2.72
Standard Deviation	0.17	0.25	**0.26**	0.24	0.17	0.23	0.25

End-to-end distance as a function of time

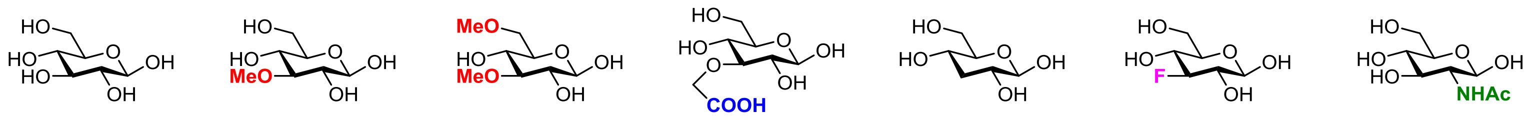

A B C D E F N

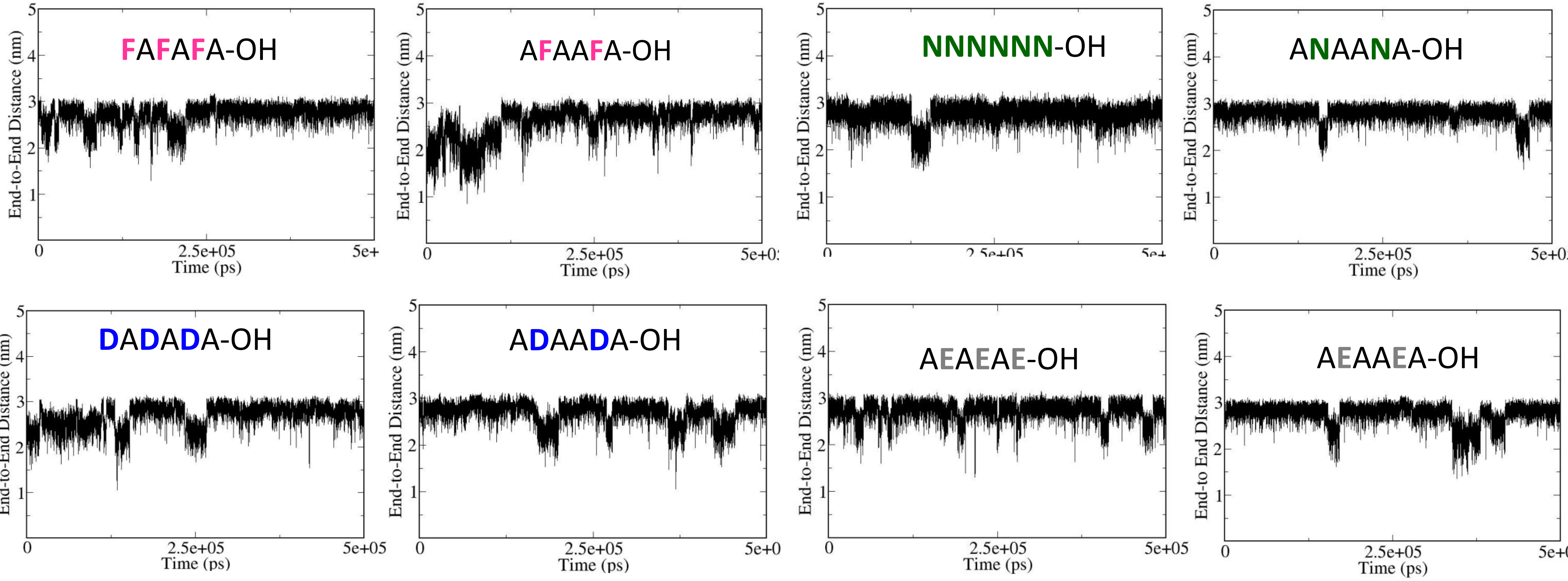

Hexamer	FAFAFA-OH	AFAAFA-OH	NNNNNN-OH	ANAANA-OH	ADAADA-OH	DADADA-OH	AEAEAE-OH	AEAAEA-OH
Average Distance (nm)	2.72	**2.61**	2.76	2.79	2.70	2.69	2.73	2.76
Standard Deviation	0.26	**0.34**	0.22	0.17	0.24	0.26	0.21	0.22

Definition of dihedrals in a glucose disaccharide

ω = O5C5C6O6

Ψ = C1O4C4H4

Φ = H1C1O4C4

(The atoms in red belong to the following residue)

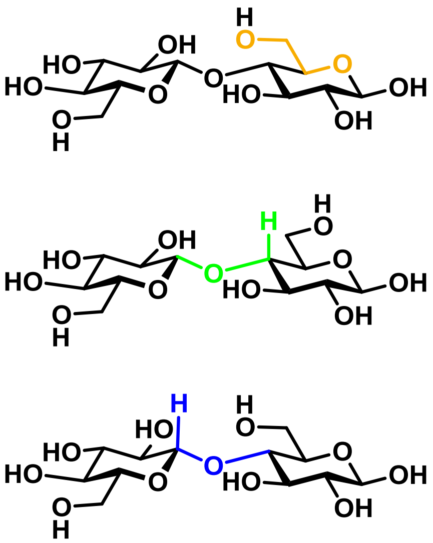

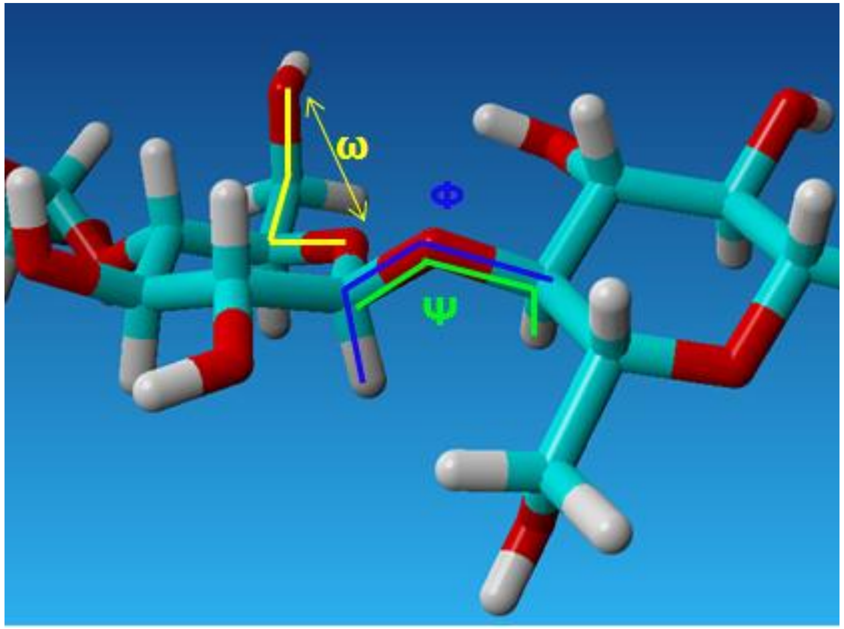

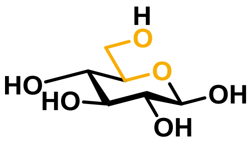

gt (ω = 60°)

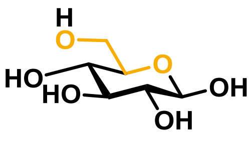

tg (ω = 180°)

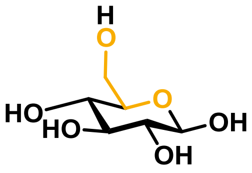

gg (ω = - 60°)

S7

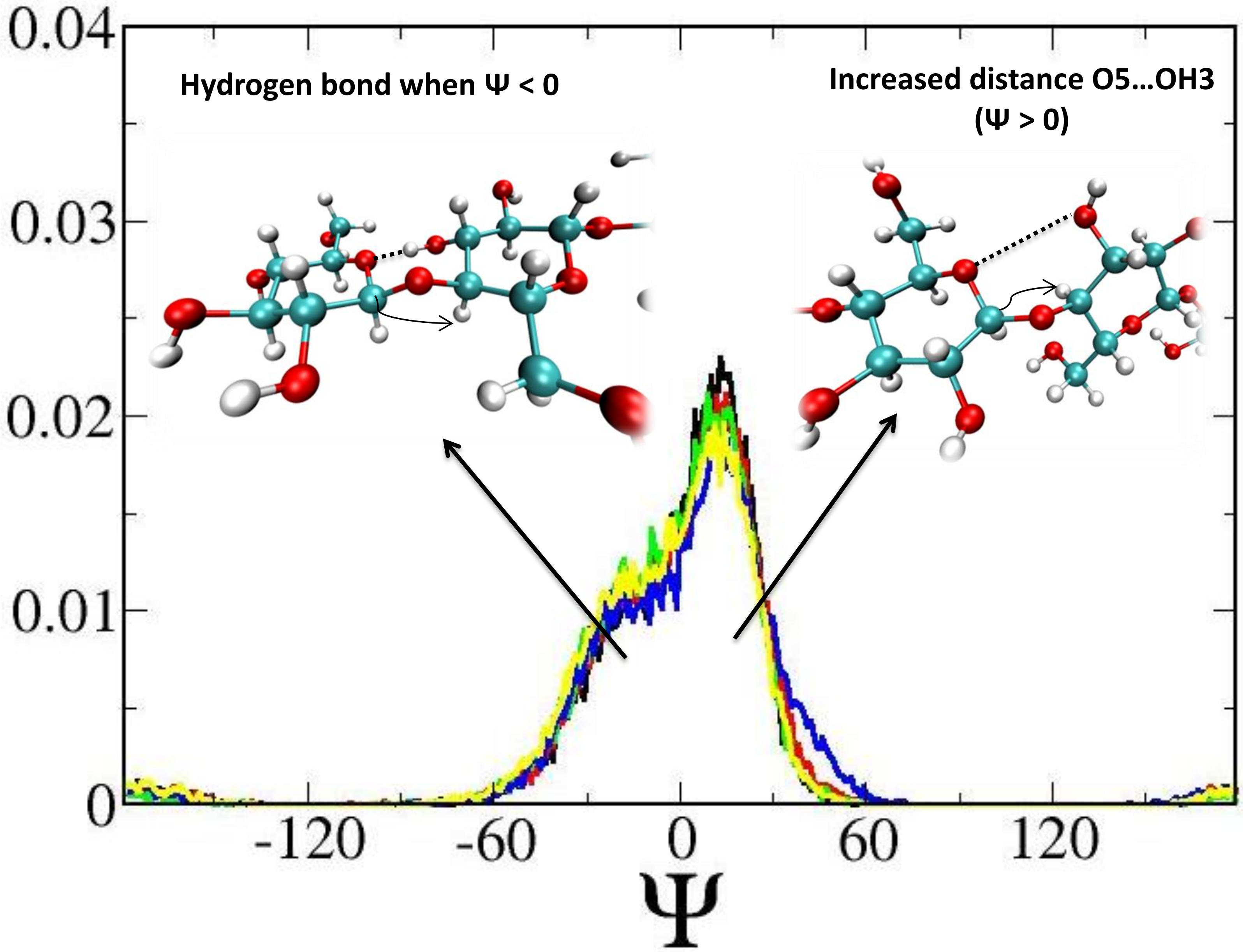

0.04
0.03
0.02
0.01
0
-120
-60
0
60
120
Ψ
Hydrogen bond when Ψ < 0
Increased distance O5…OH3
(Ψ > 0)

S8

Omega (ω) torsion angles.

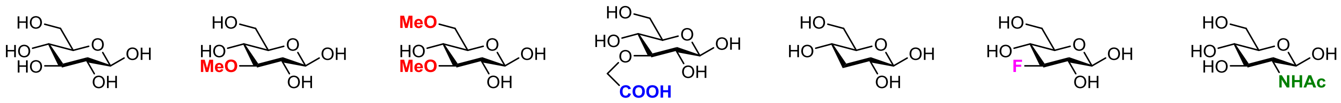

A B C D E F N

Omega (ω) 1		Omega (ω) 2		Omega (ω) 3		Omega (ω) 4		Omega (ω) 5		Omega (ω) 6	

Non-reducing end Reducing end

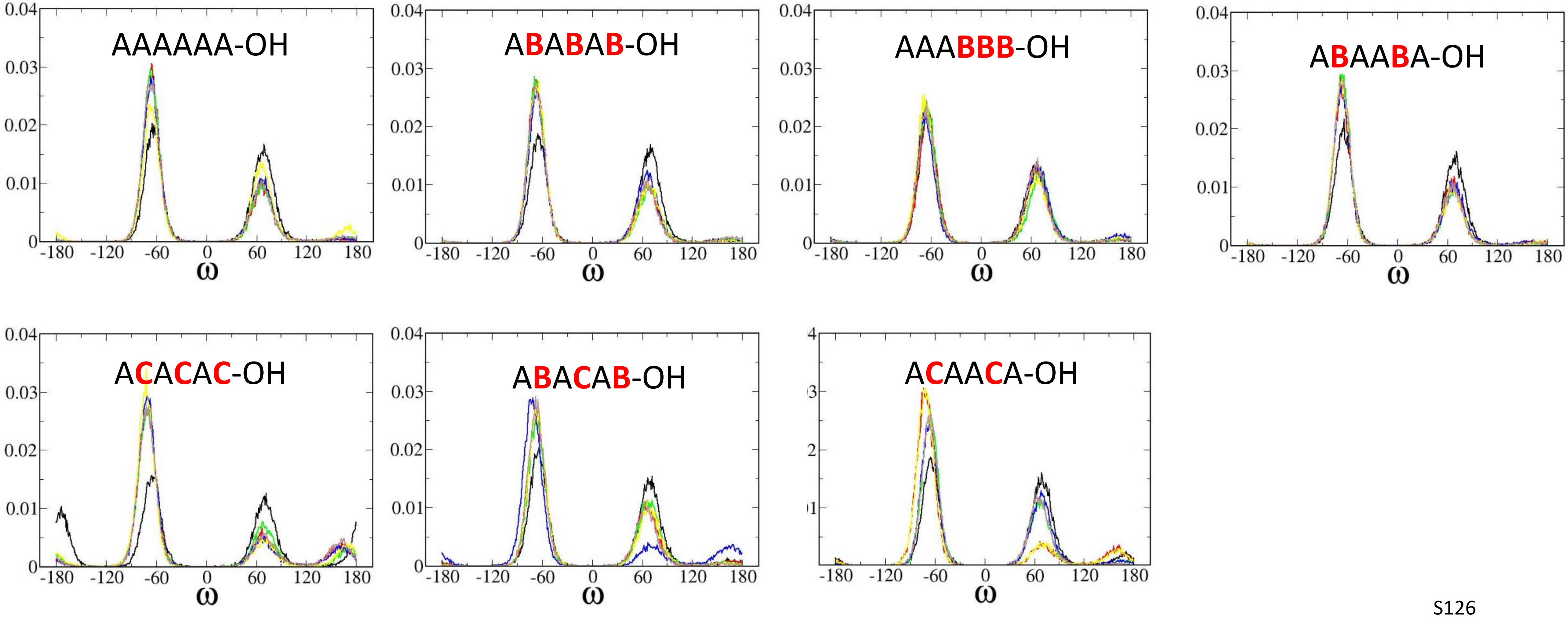

Omega (ω) torsion angles.

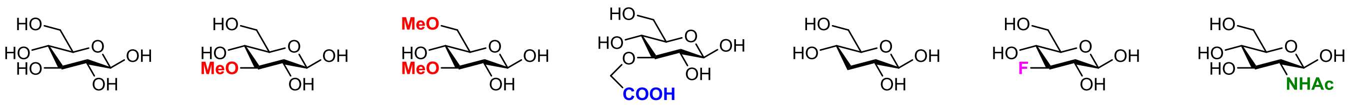

A B C D E F N

Omega (ω) 1		Omega (ω) 2		Omega (ω) 3		Omega (ω) 4		Omega (ω) 5		Omega (ω) 6	

Non-reducing end — Reducing end

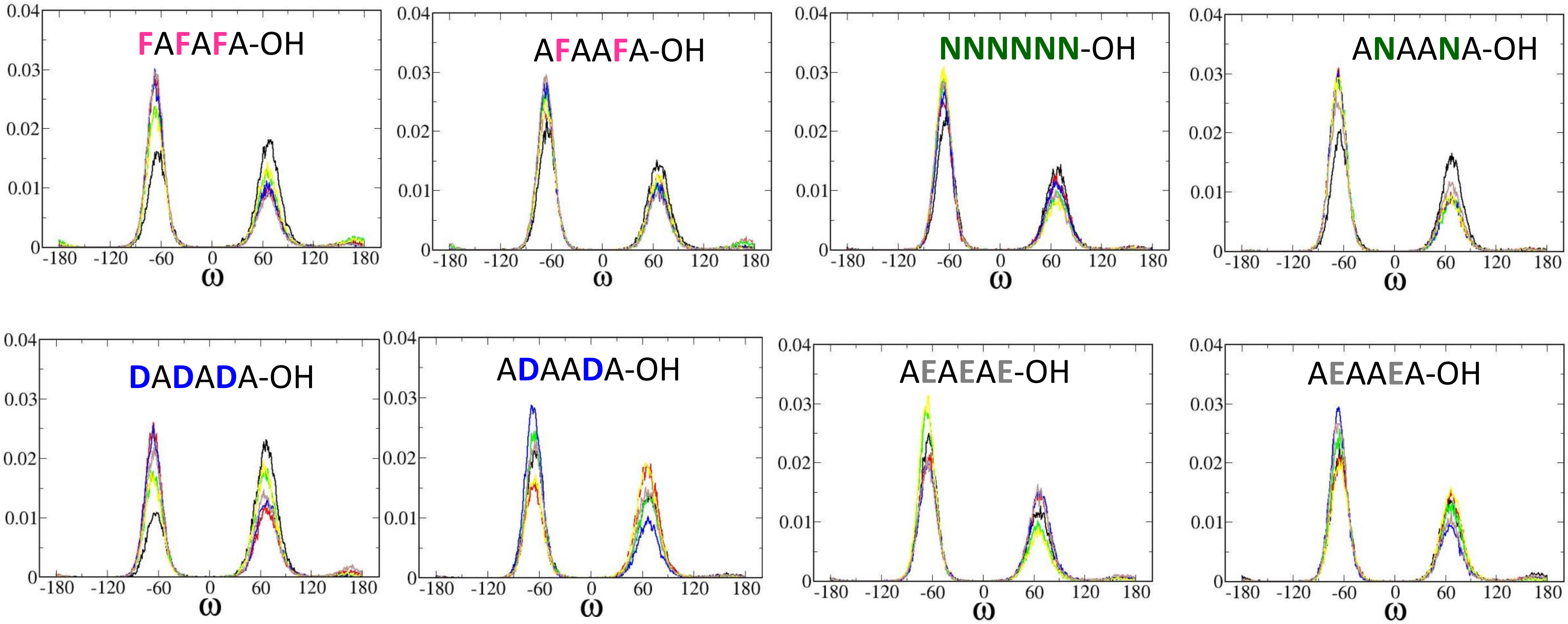

S9

Phi (ϕ) torsion angles.

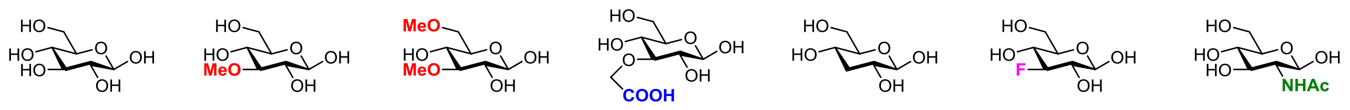

A B C D E F N

Phi (ϕ) 1		Phi (ϕ) 2		Phi (ϕ) 3		Phi (ϕ) 4		Phi (ϕ) 5	

Non-reducing end — Reducing end

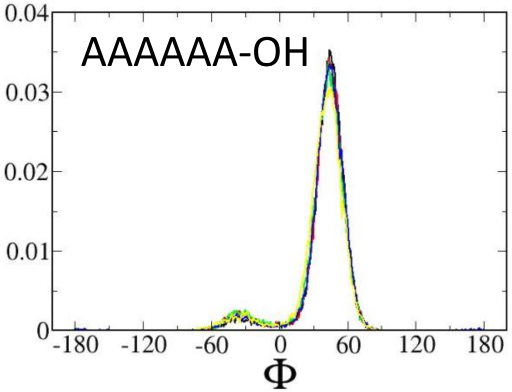

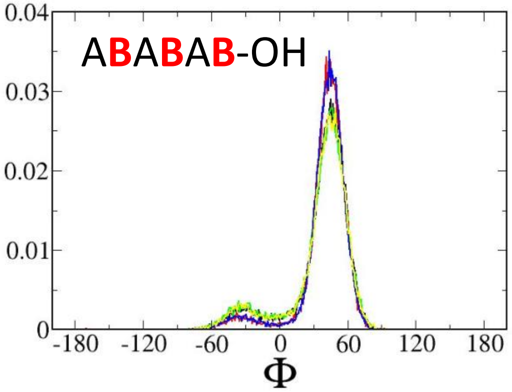

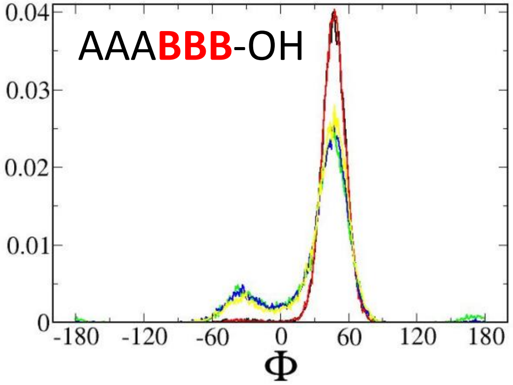

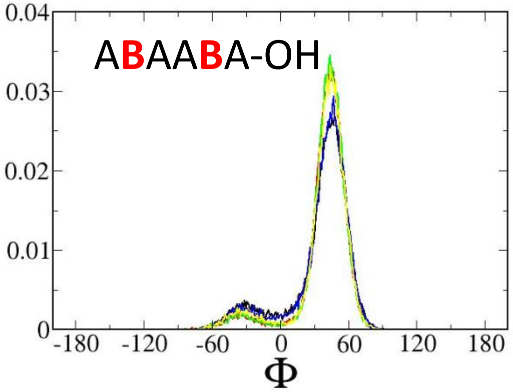

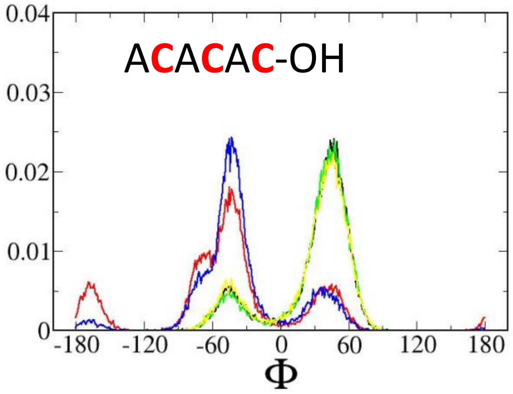

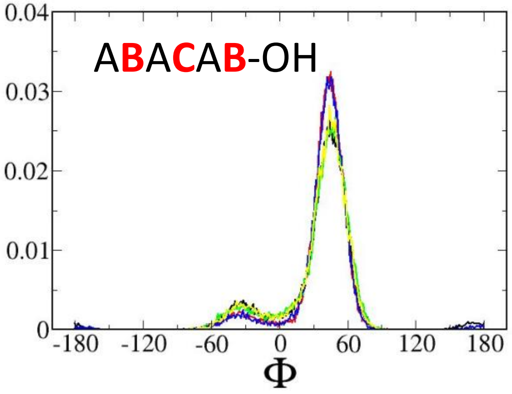

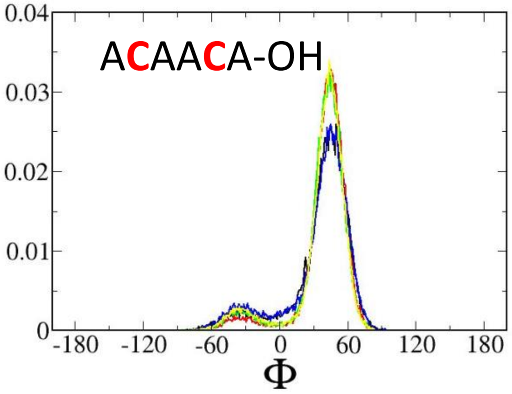

Phi (ϕ) torsion angles.

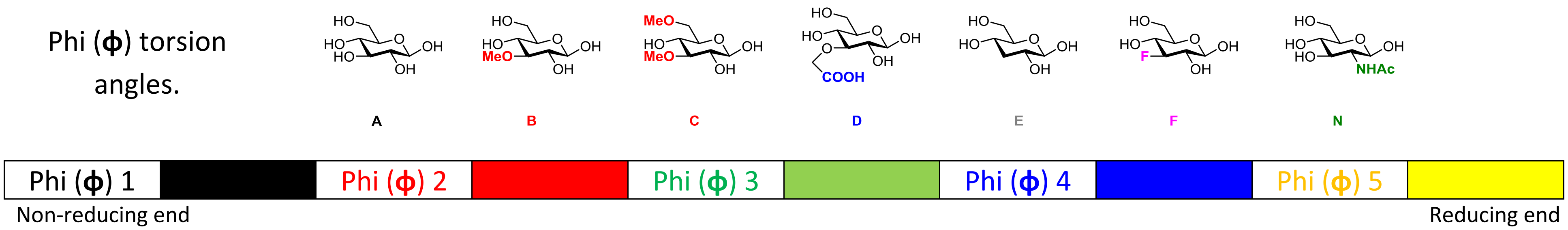

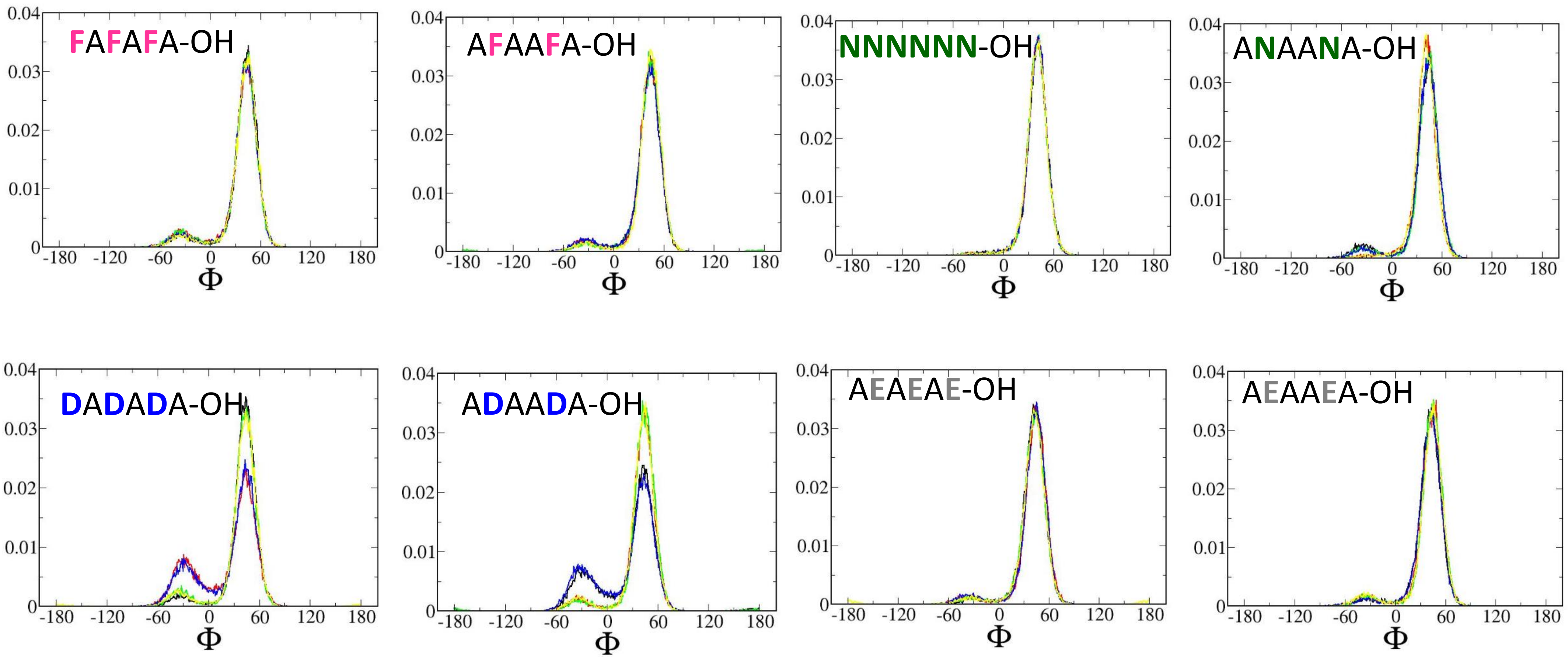

S10

Psi (ψ) torsion angles.

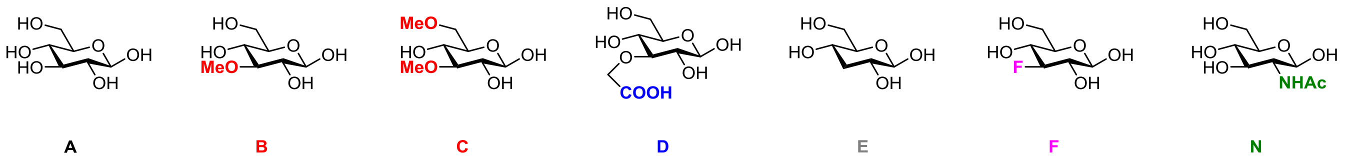

Psi (ψ) 1		Psi (ψ) 2		Psi (ψ) 3		Psi (ψ) 4		Psi (ψ) 5	

Non-reducing end — Reducing end

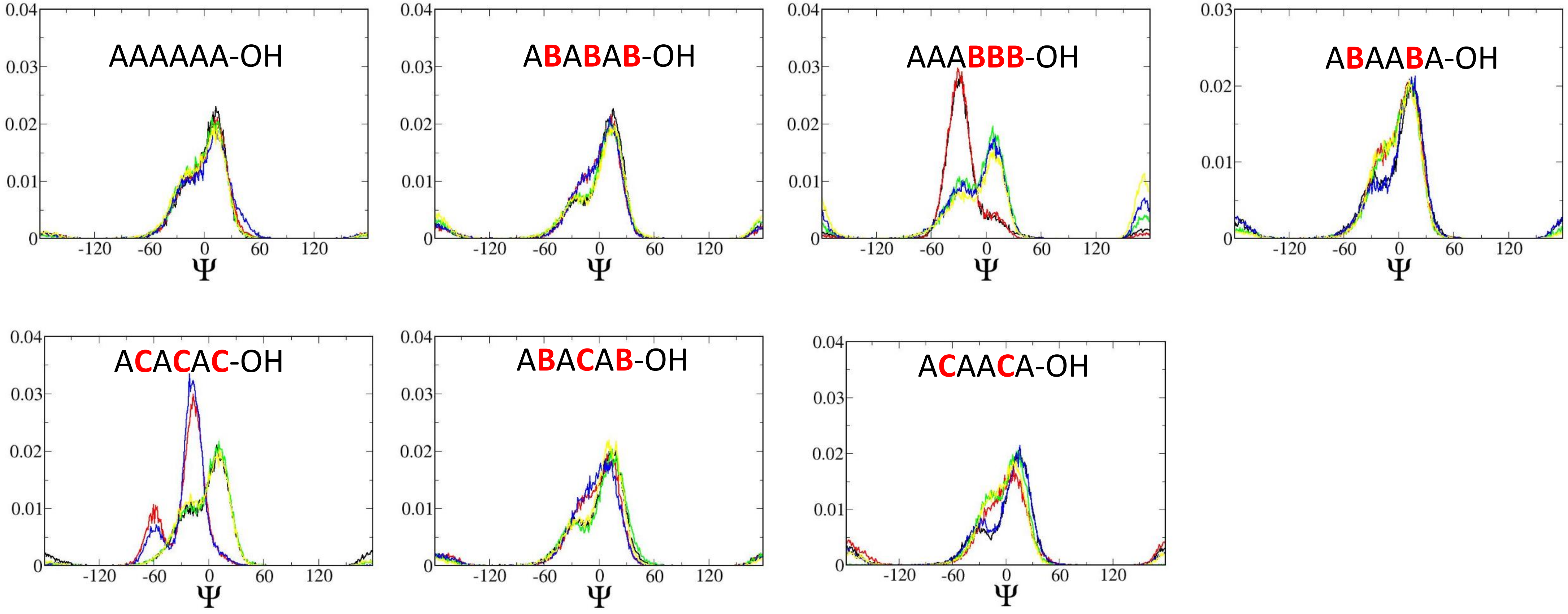

Psi (ψ) torsion angles.

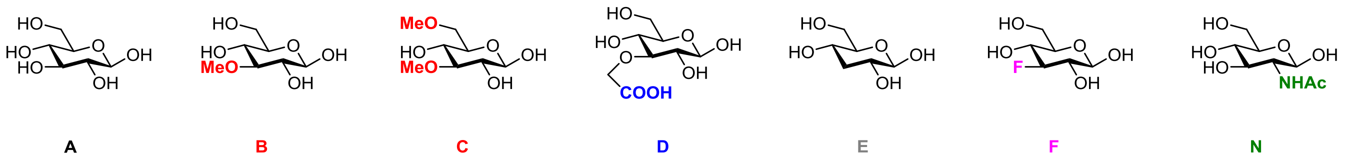

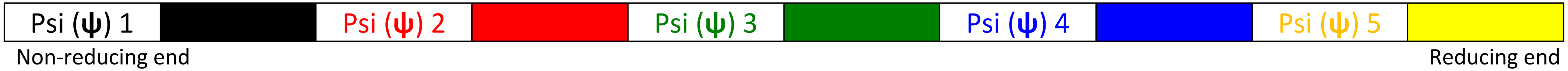

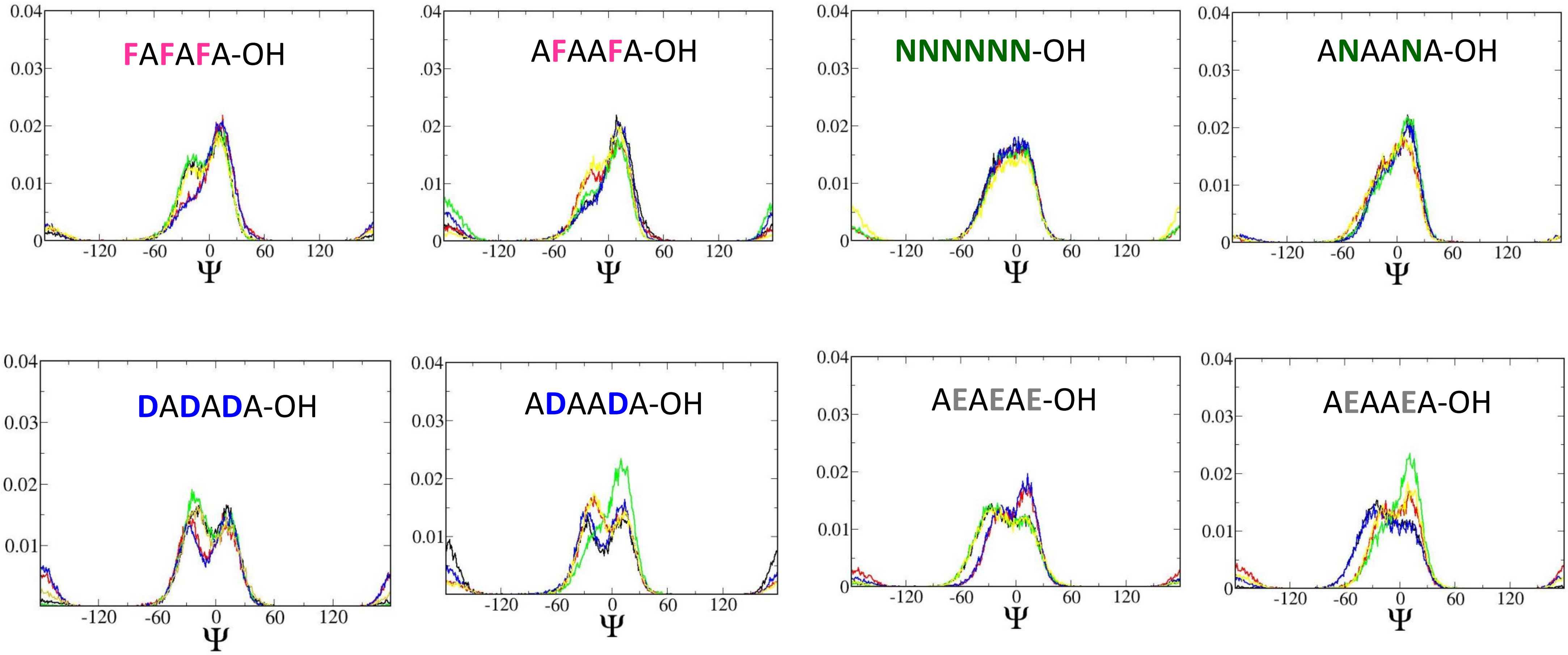

S11 Overimposed puckering theta (θ) values

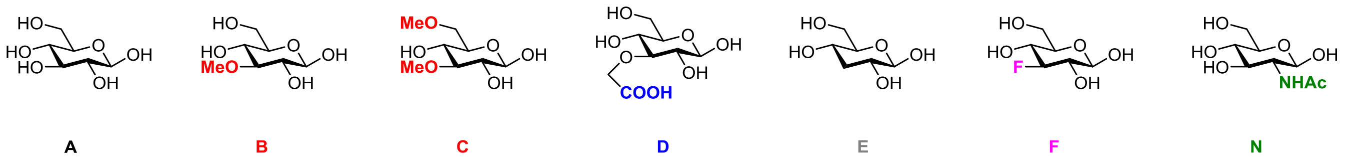

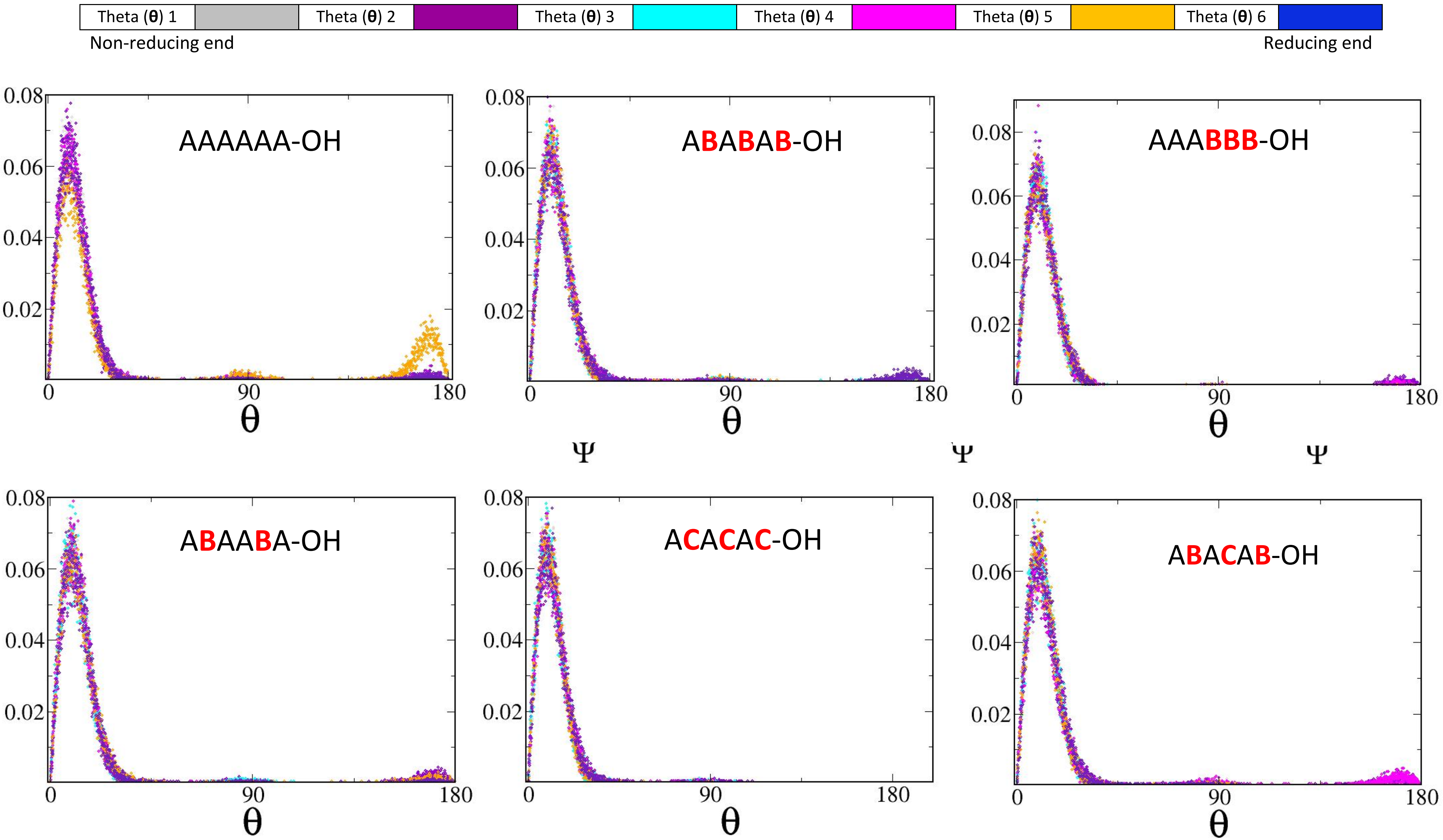

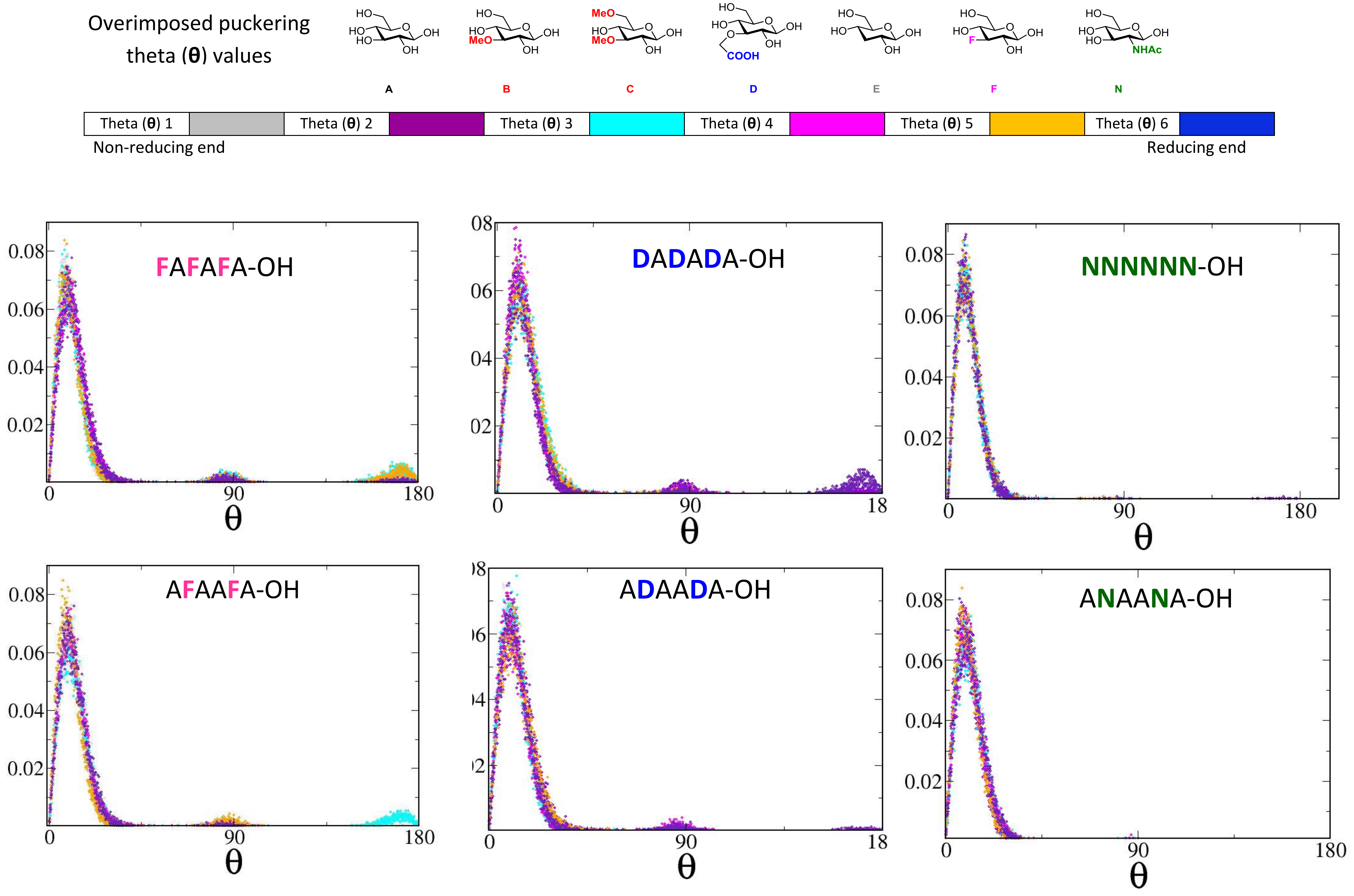

Overimposed puckering theta (θ) values
A
B
C
D
E
F
N
Theta (θ) 1
Theta (θ) 2
Theta (θ) 3
Theta (θ) 4
Theta (θ) 5
Theta (θ) 6
Non-reducing end
Reducing end
FAFAFA-OH
DADADA-OH
NNNNNN-OH
AFAAFA-OH
ADAADA-OH
ANAANA-OH
θ

Overimpose puckering theta (θ) values

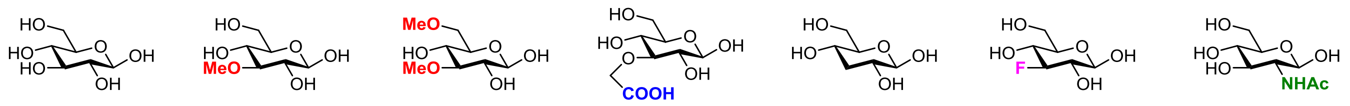

A B C D E F N

Theta (θ) 1		Theta (θ) 2		Theta (θ) 3		Theta (θ) 4		Theta (θ) 5		Theta (θ) 6	

Non-reducing end Reducing end

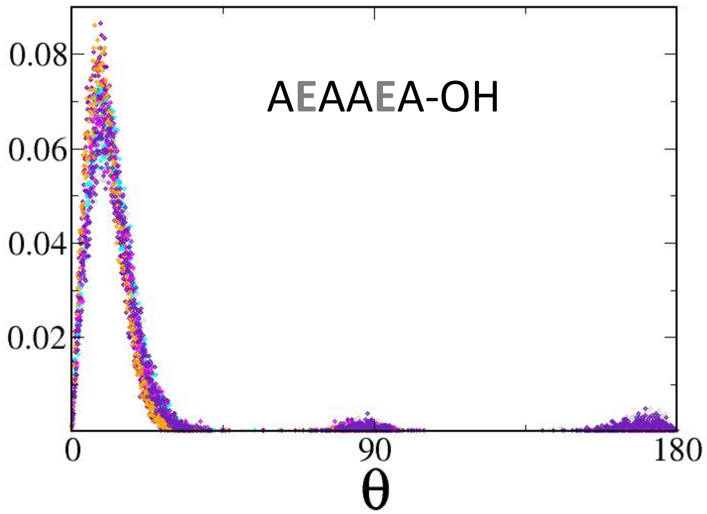

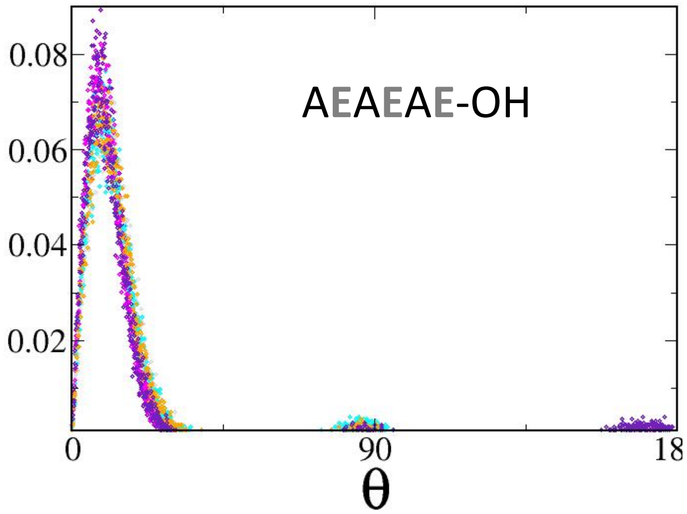

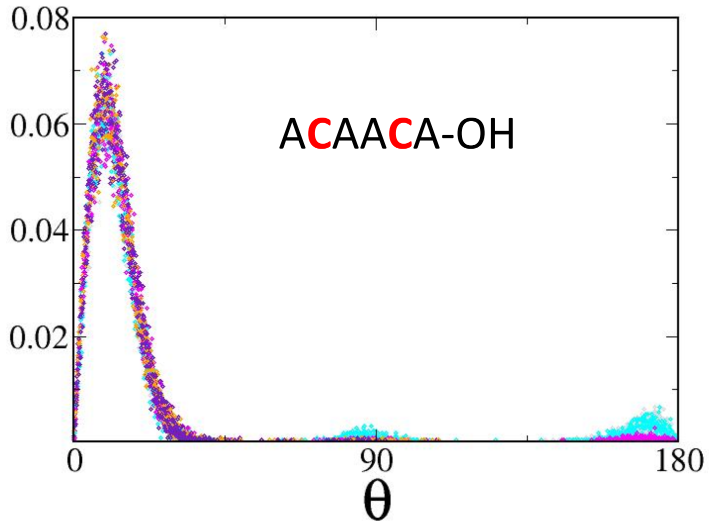

S12

Ramachandran plots

Overimposed phi and psi torsion angles

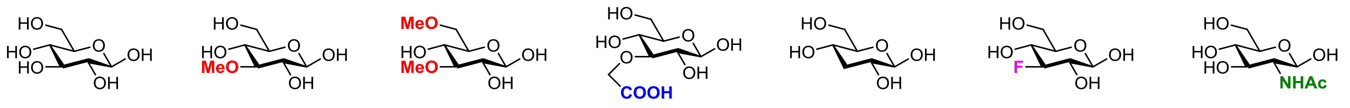

A B C D E F N

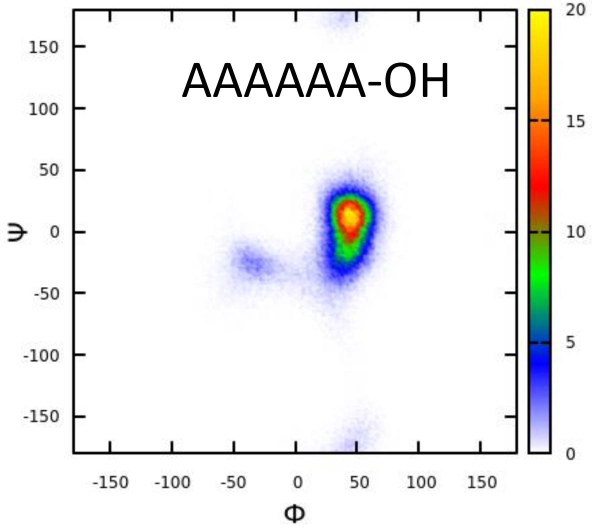

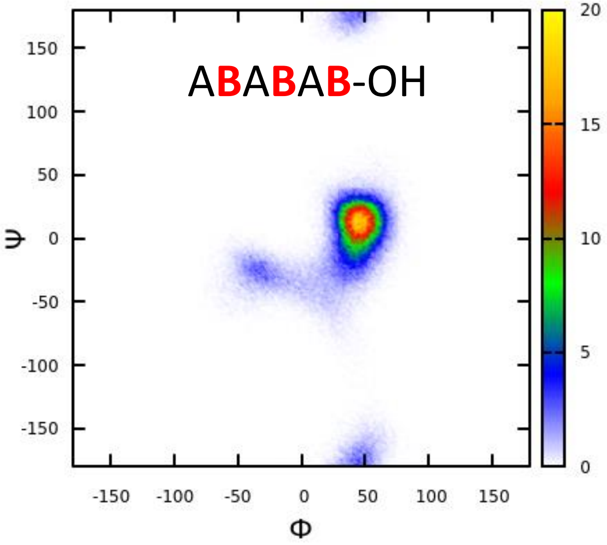

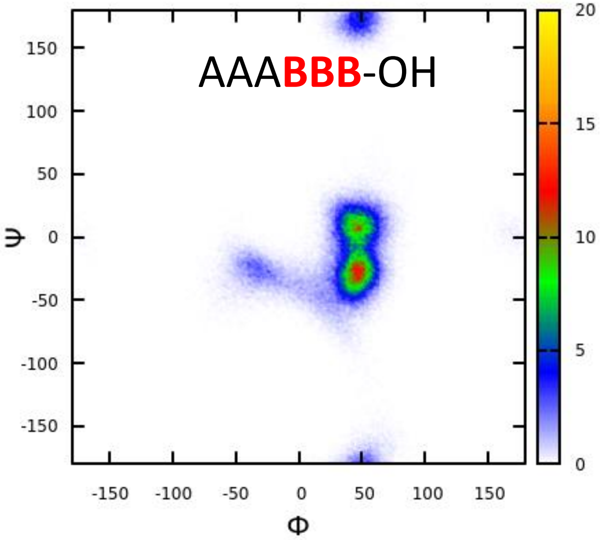

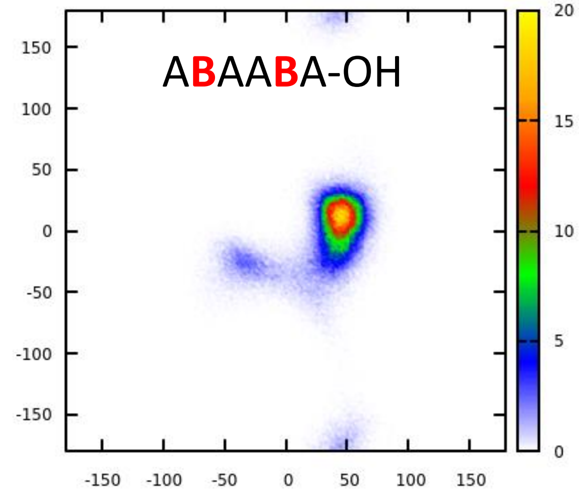

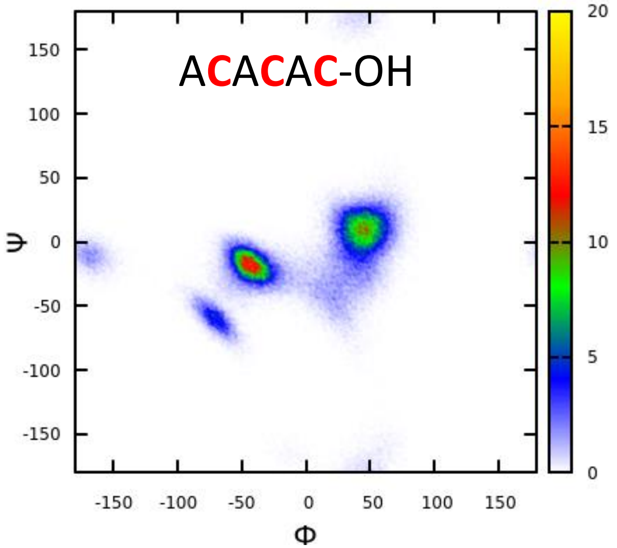

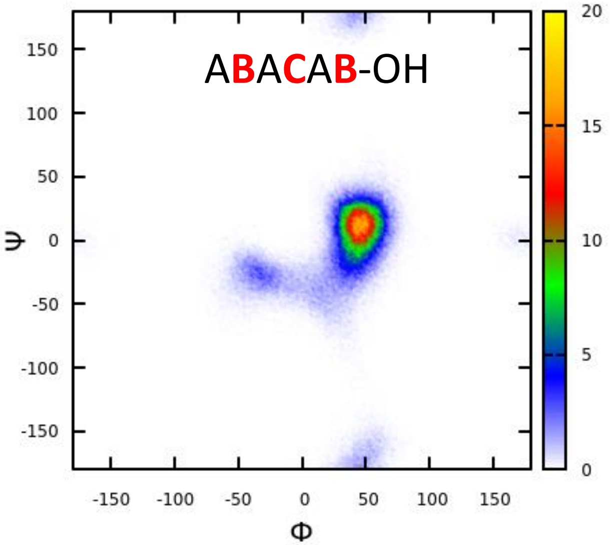

Ramachandran plots

Overimposed phi and psi torsion angles

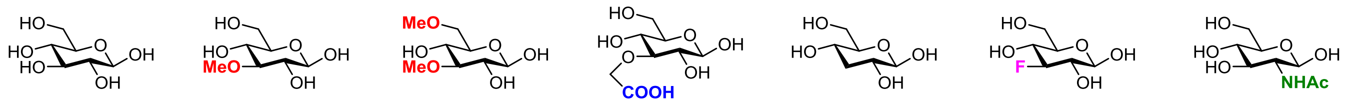

A B C D E F N

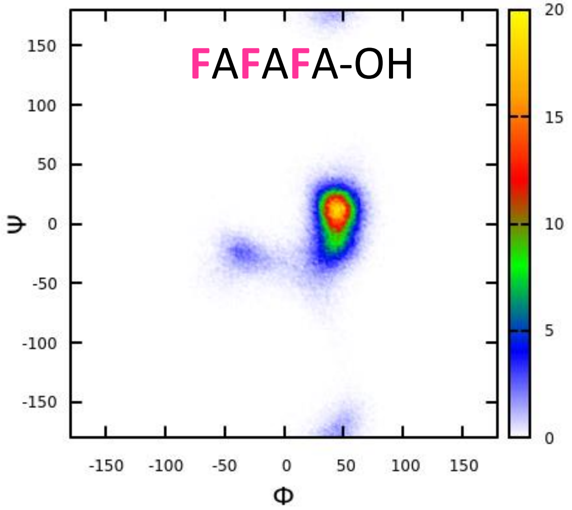

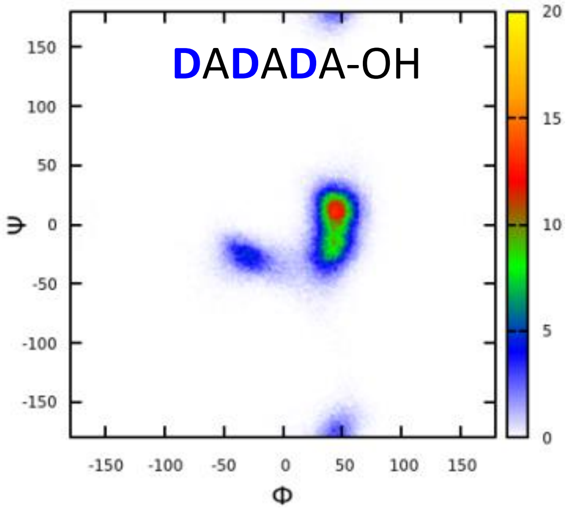

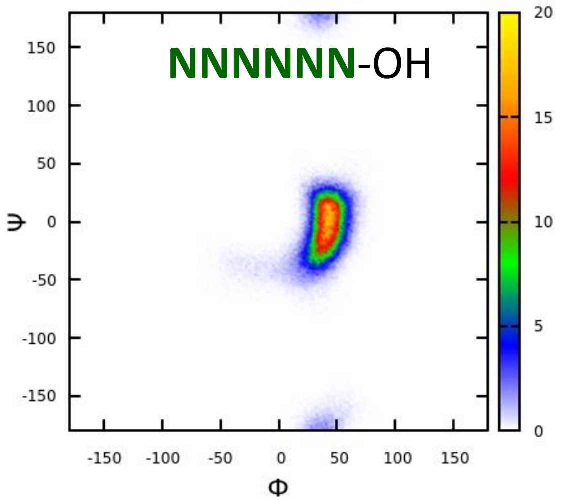

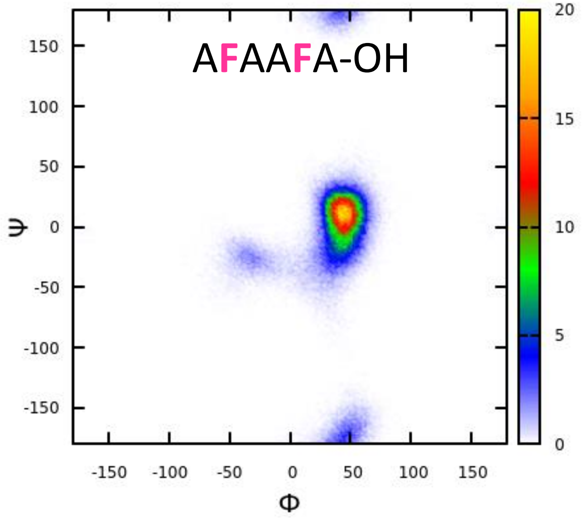

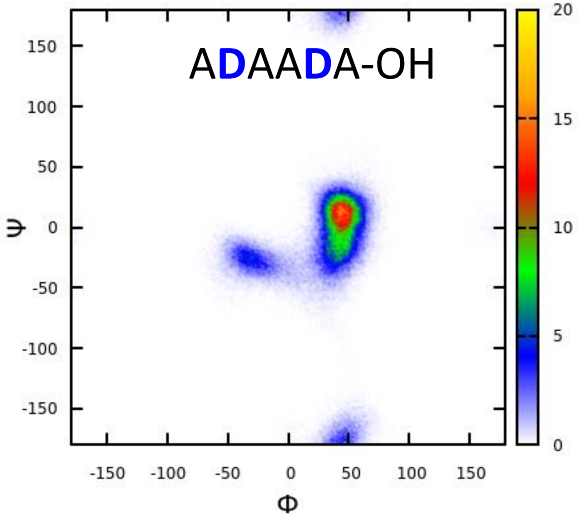

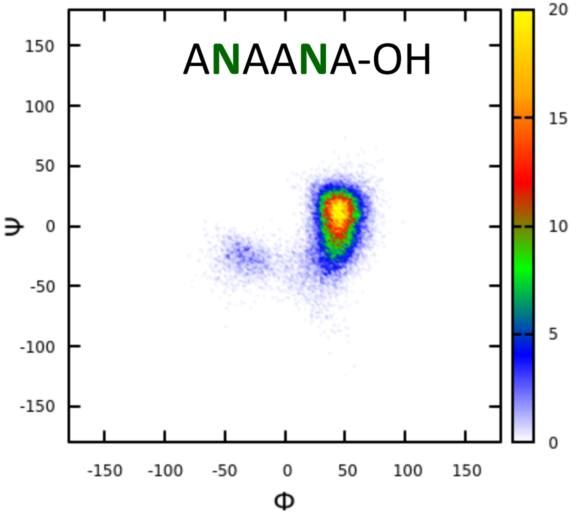

Ramachandran plots

Overimposed phi and psi torsion angles

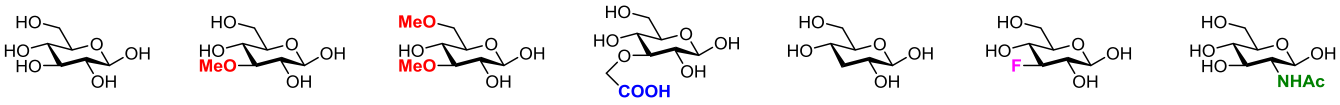

A B C D E F N

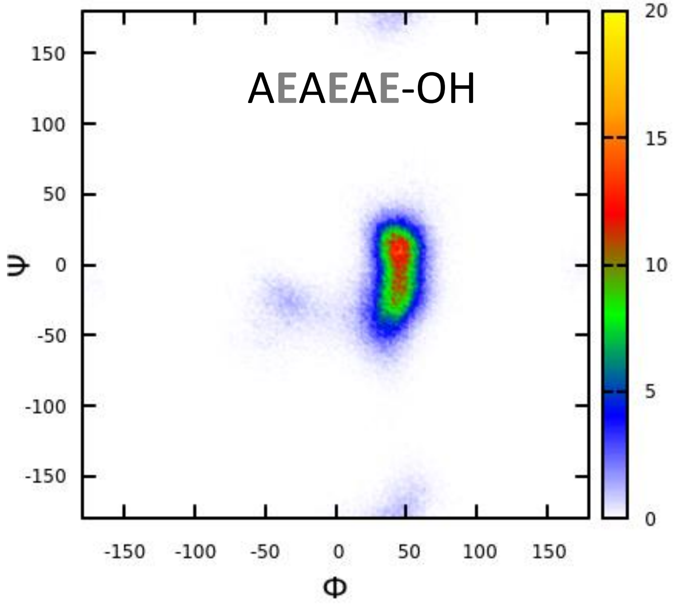

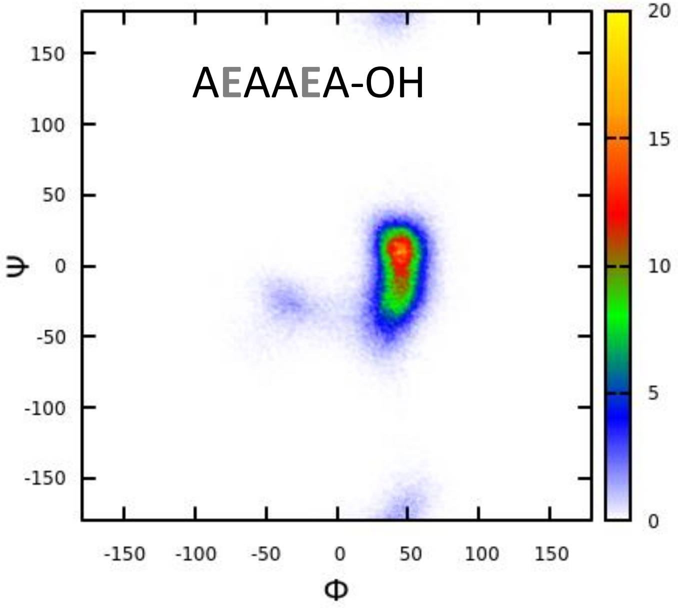

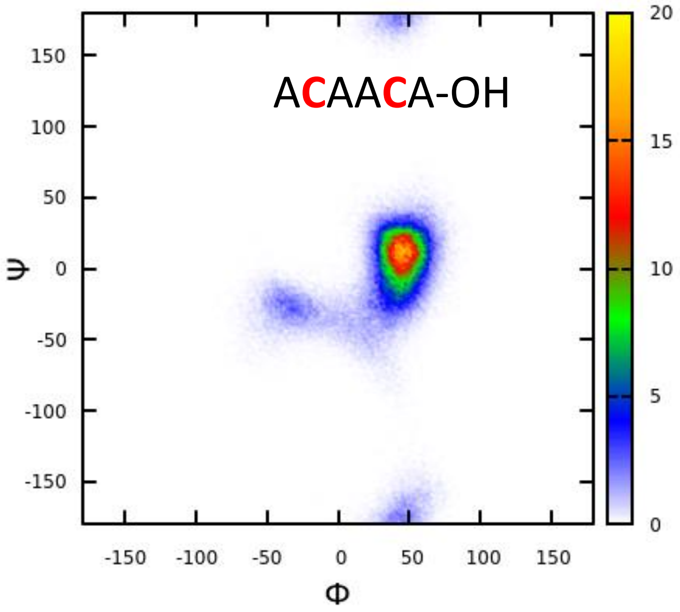

Ramachandran plots

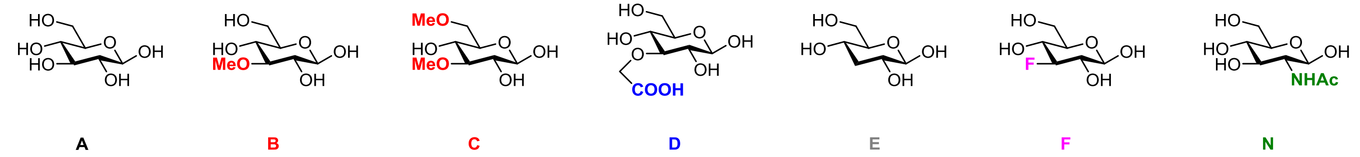

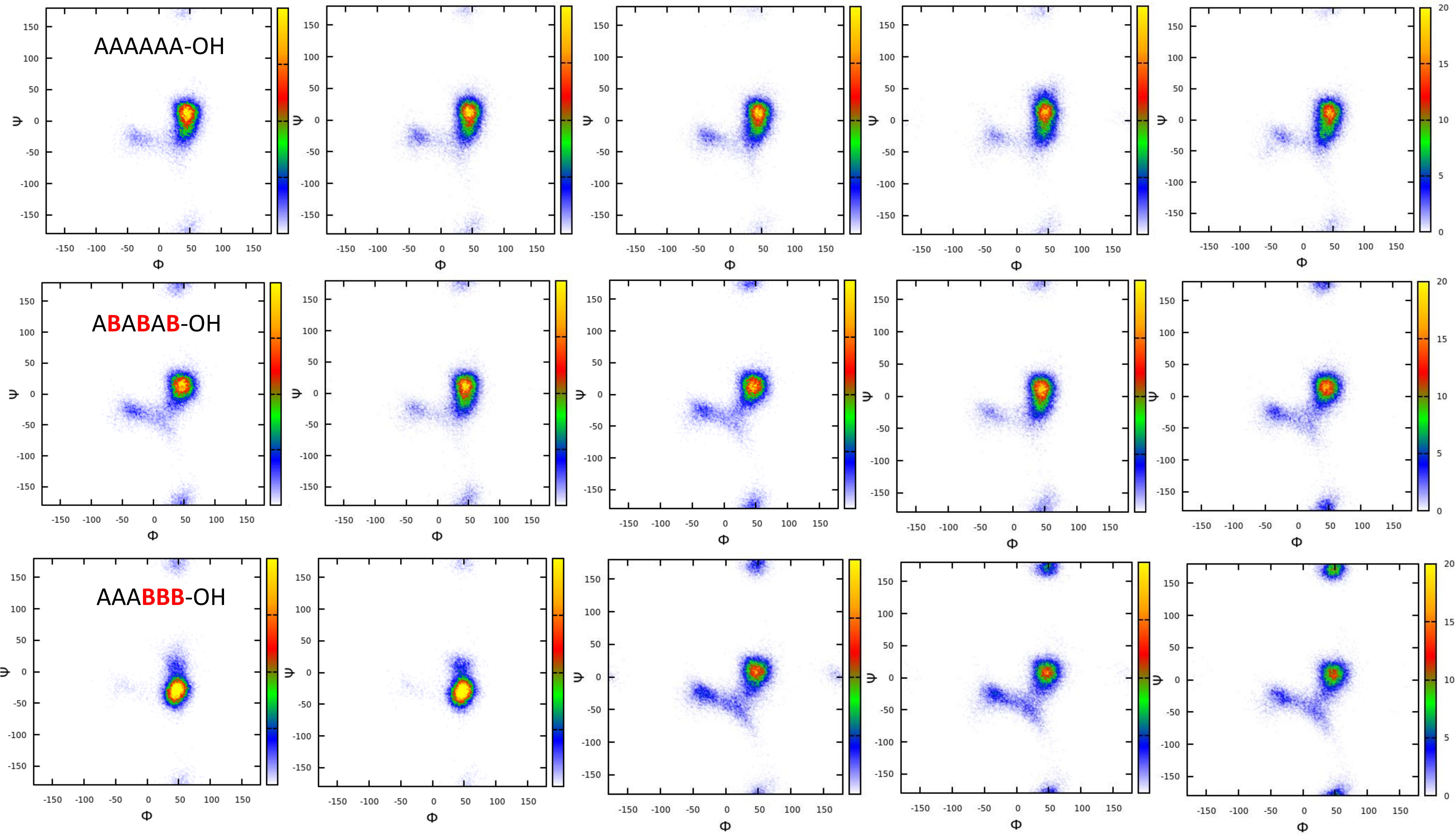

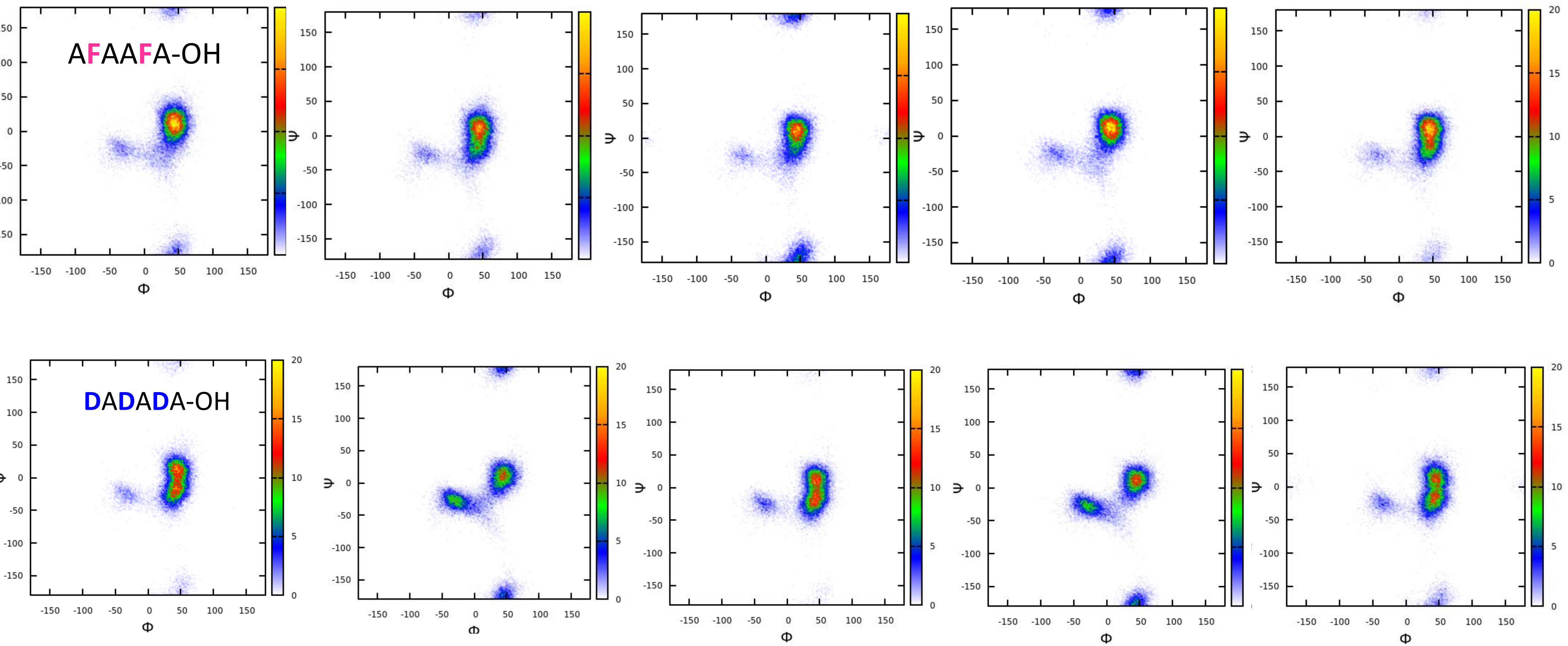
AFAAFA-OH
DADADA-OH

S13

Number of H-bonds detected during simulations

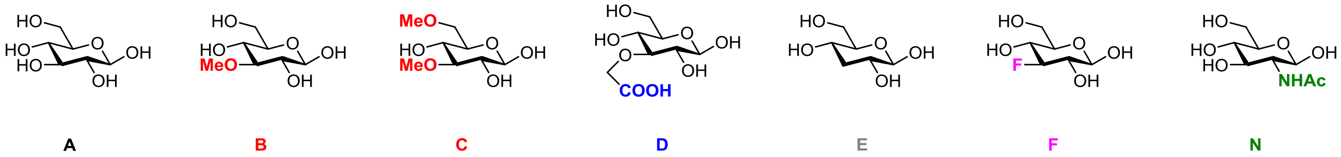

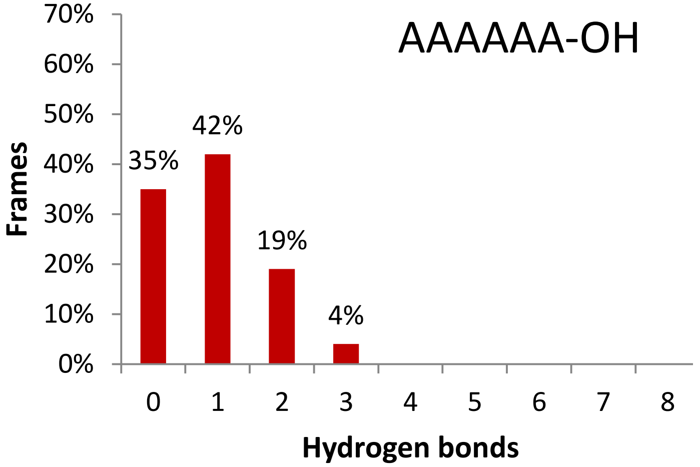

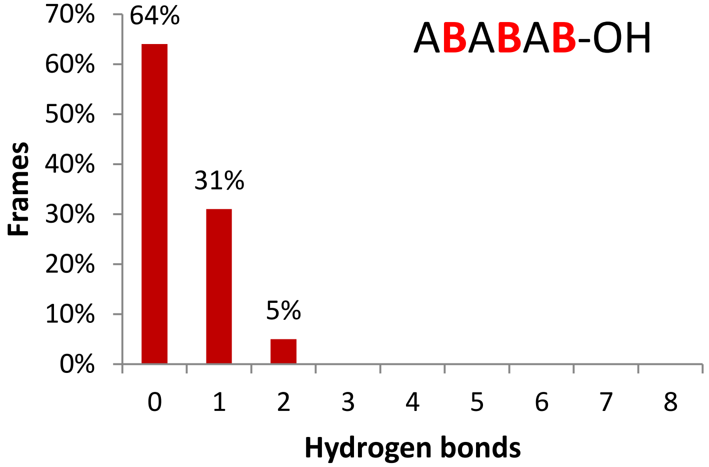

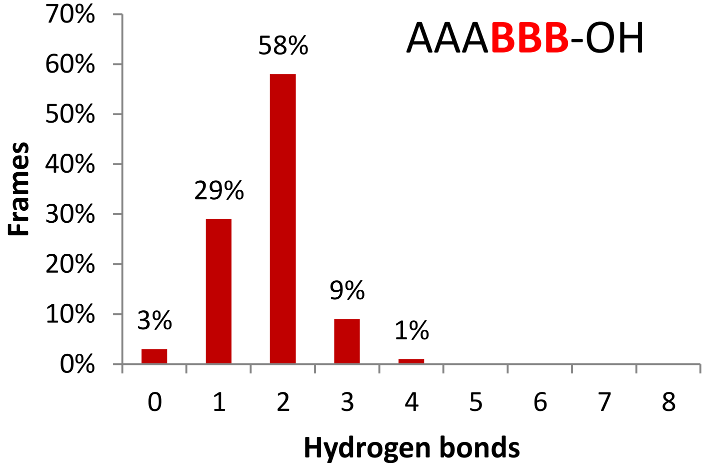

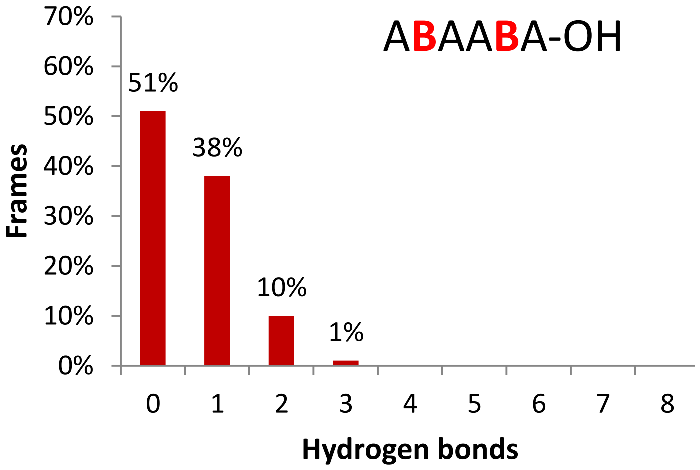

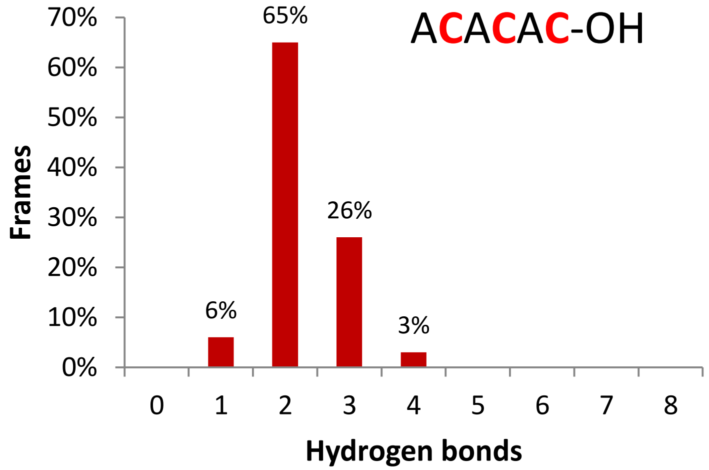

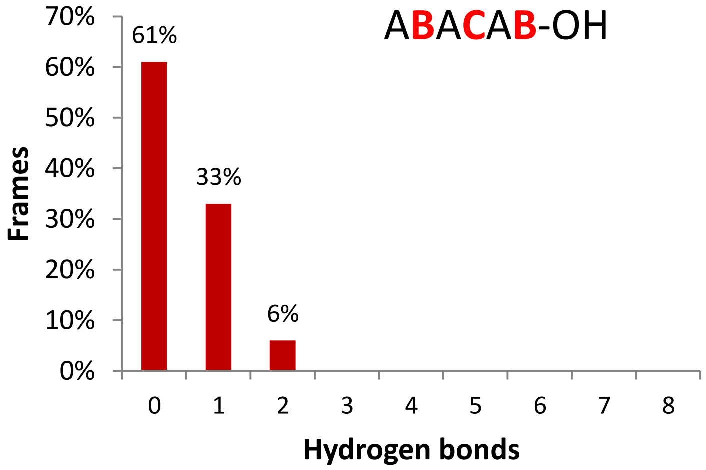

Number of H-bonds detected during simulations

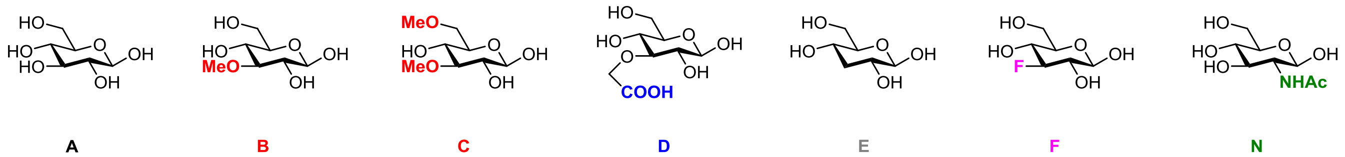

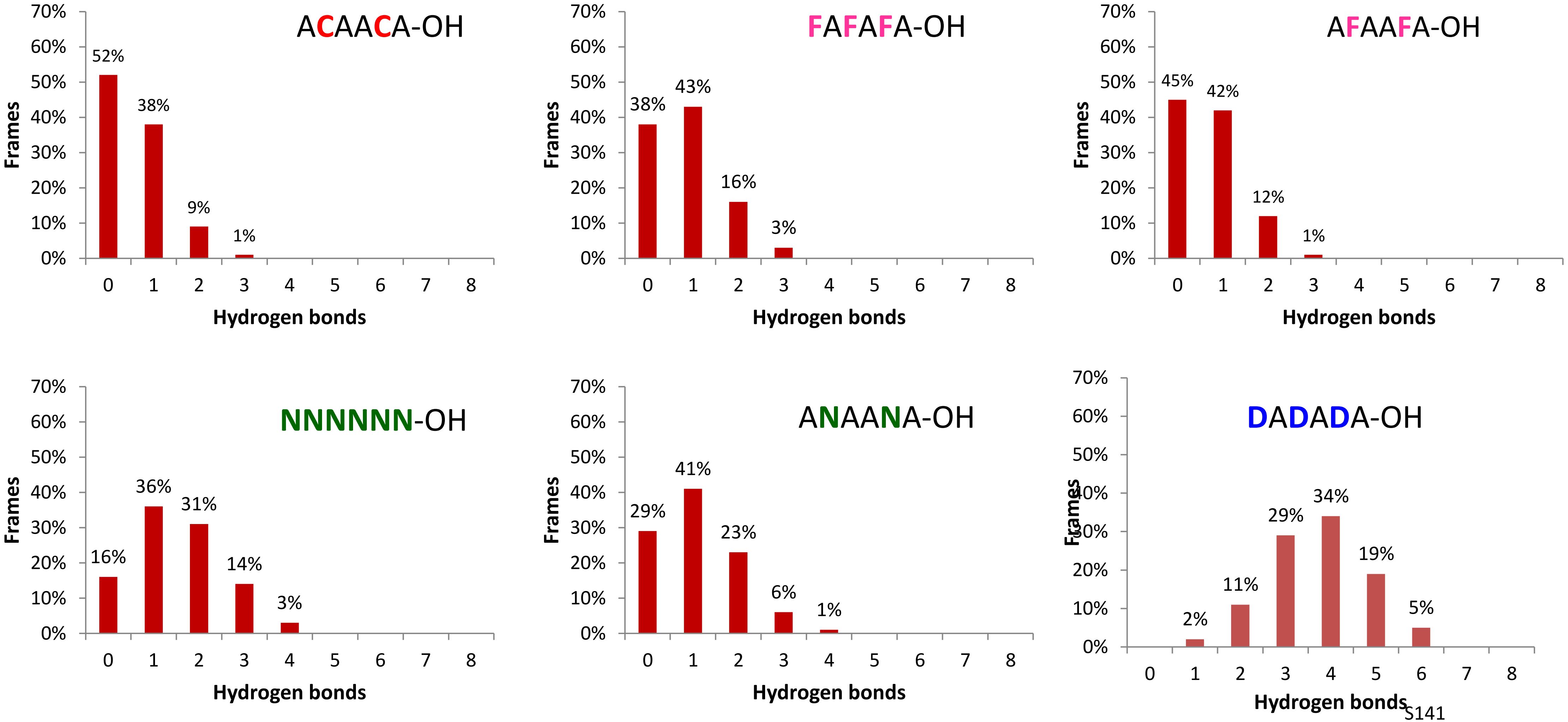

Number of H-bonds detected during simulations

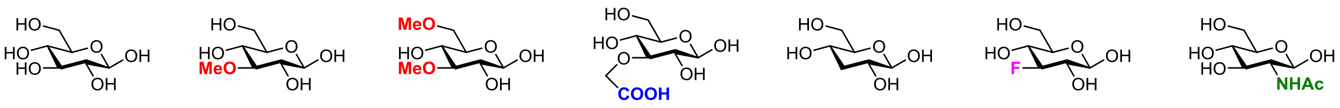

A B C D E F N

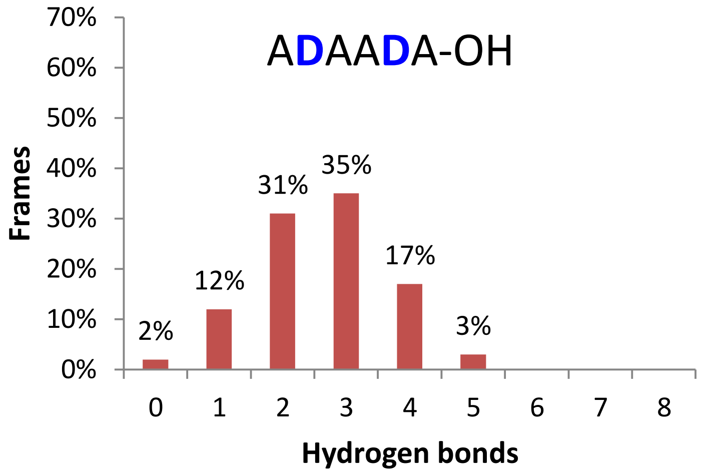

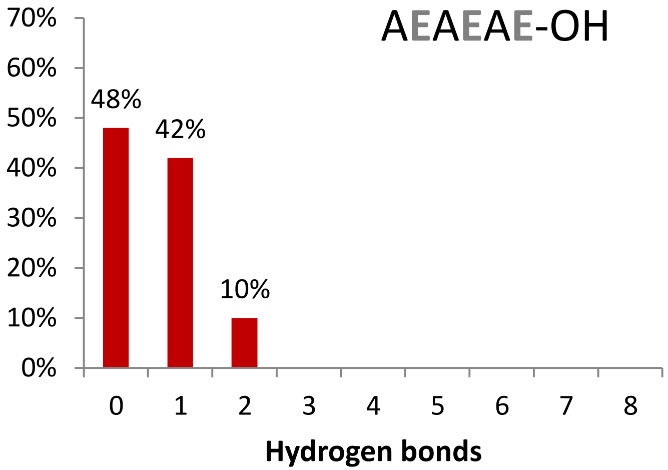

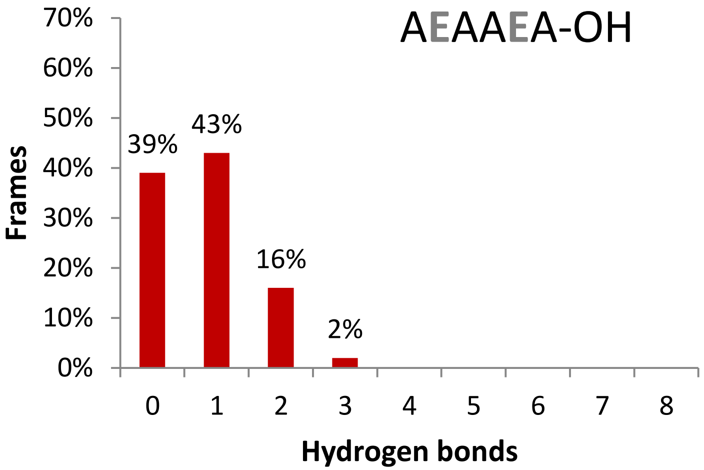

S14

Representative structure of

AAAAAA-OH

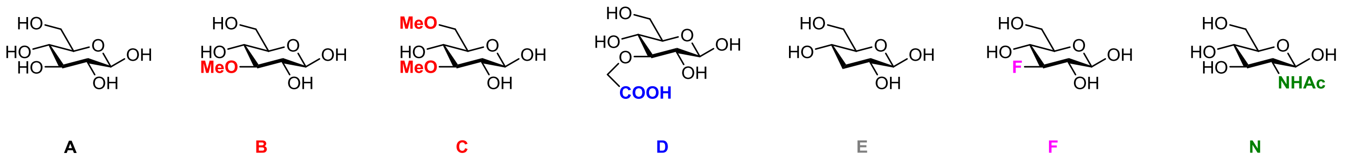

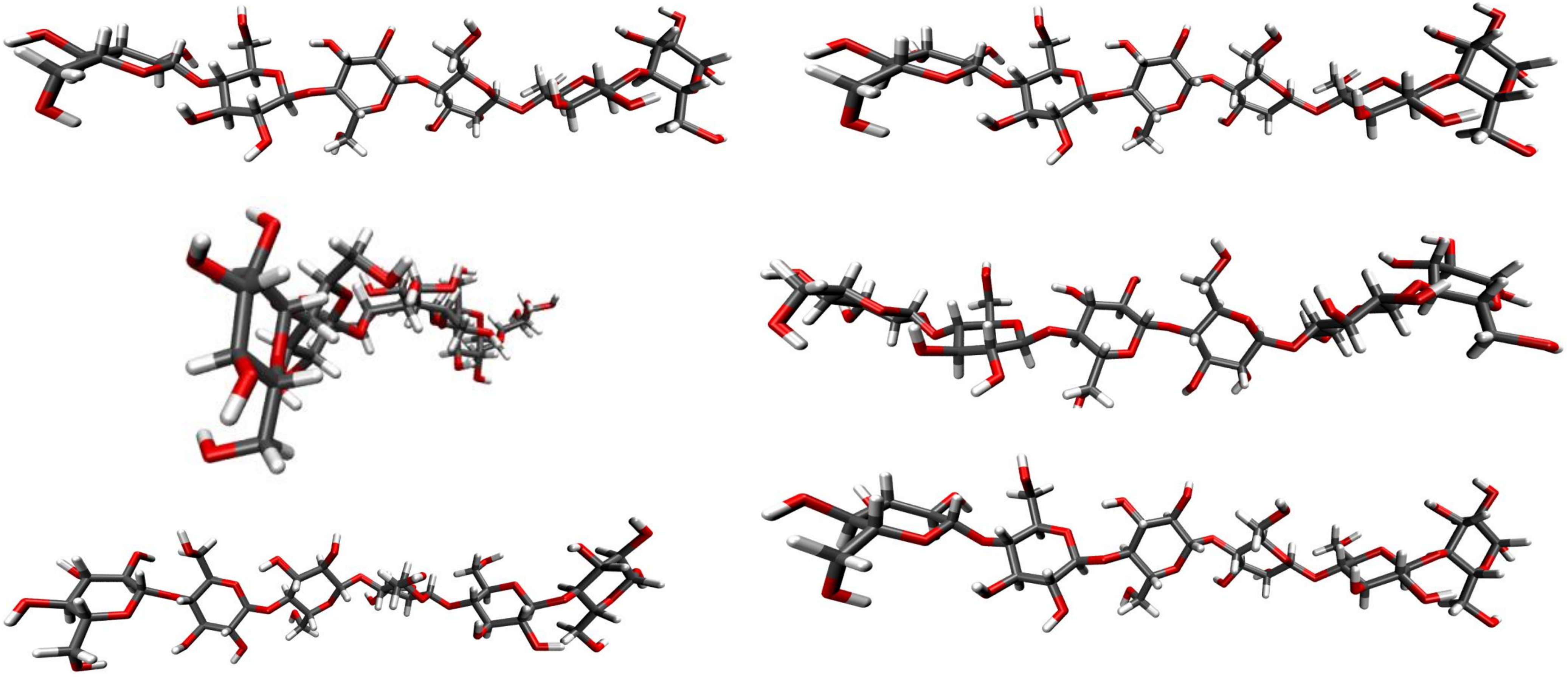

Representative structure of

ABABAB-OH

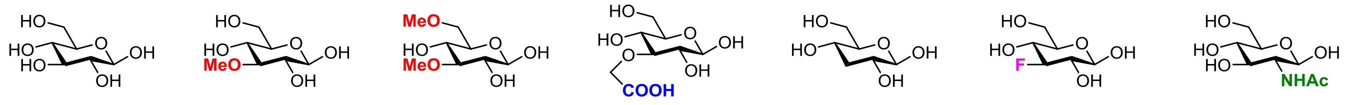

A B C D E F N

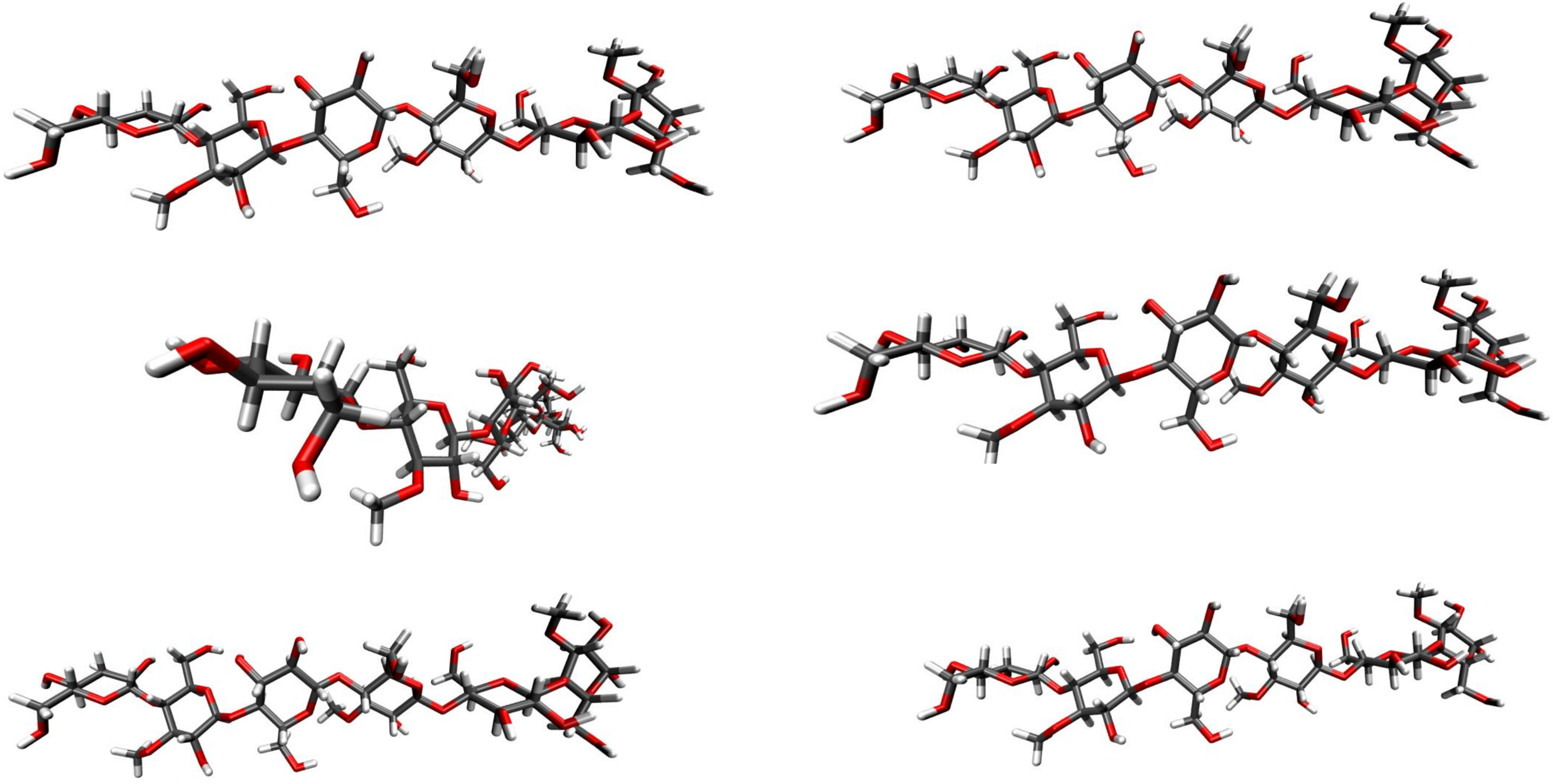

Representative structure of

AAABBB-OH

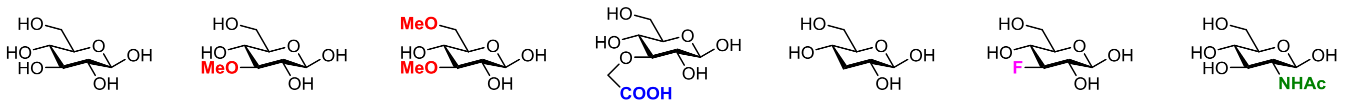

A **B** **C** **D** **E** **F** **N**

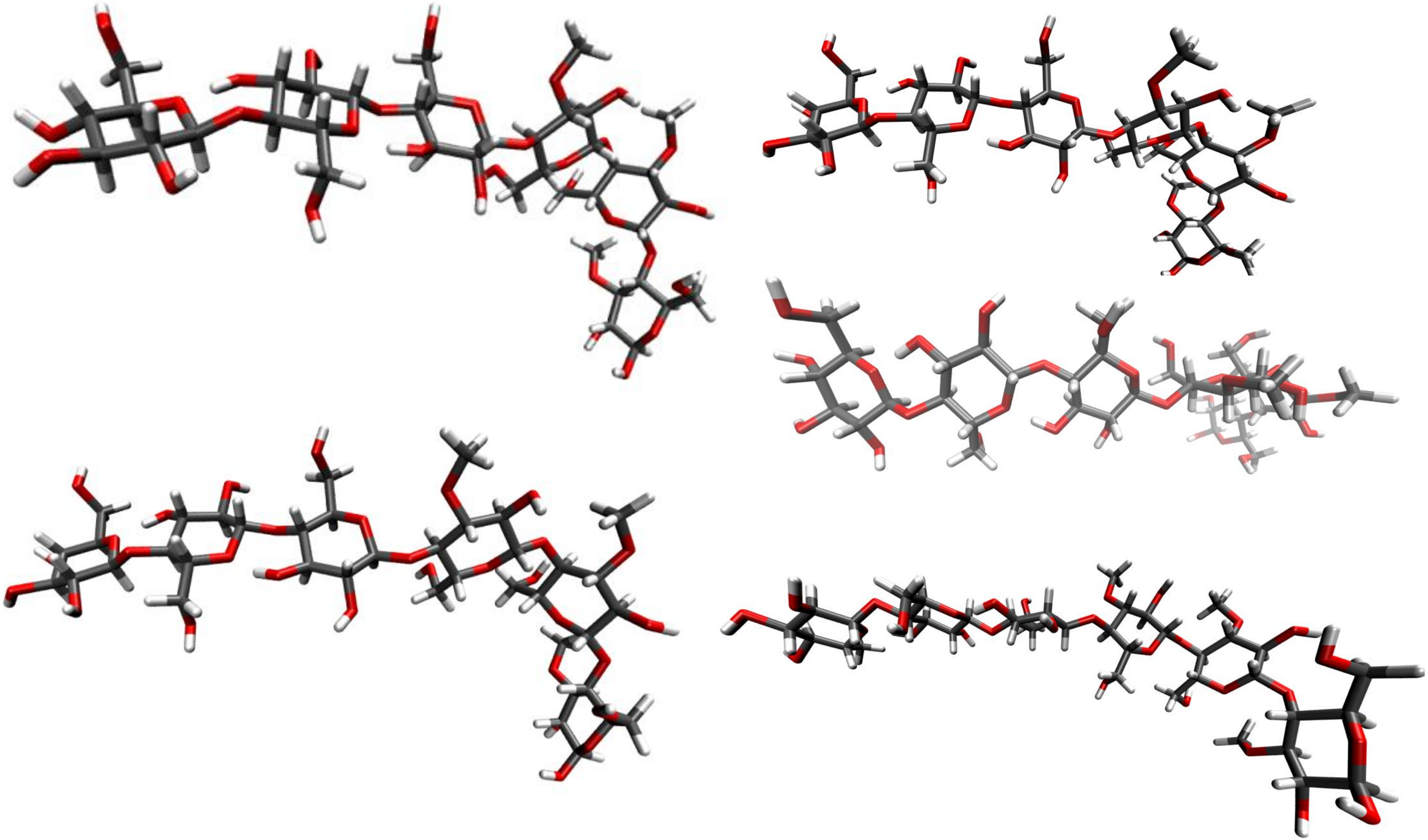

Representative structure of

ABAABA-OH

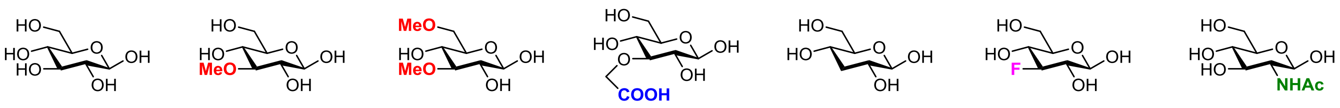

A B C D E F N

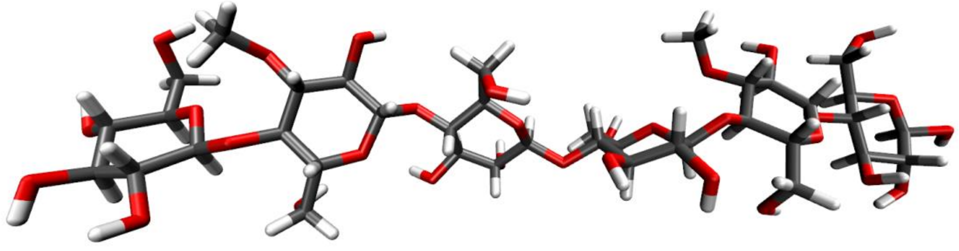

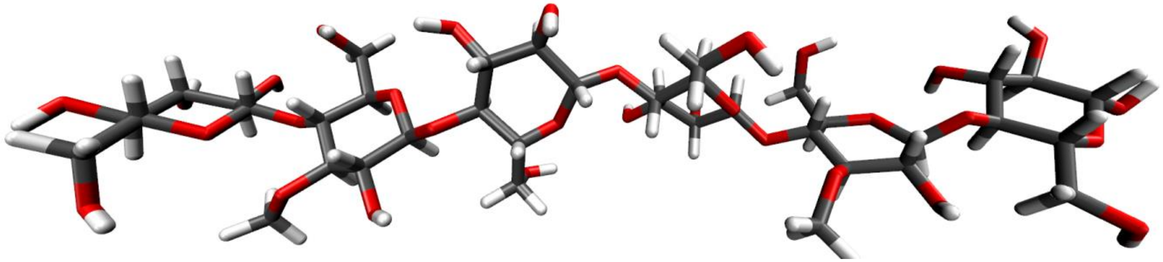

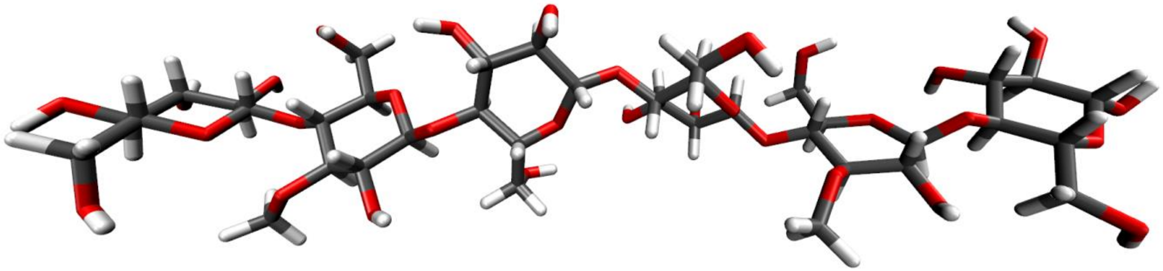

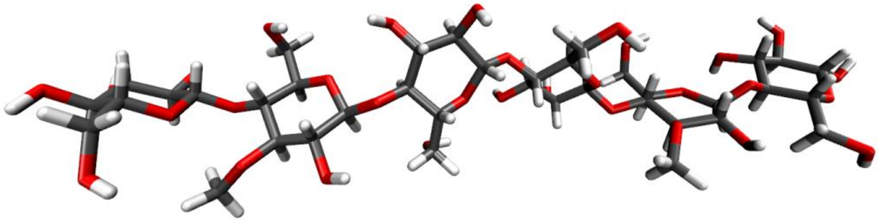

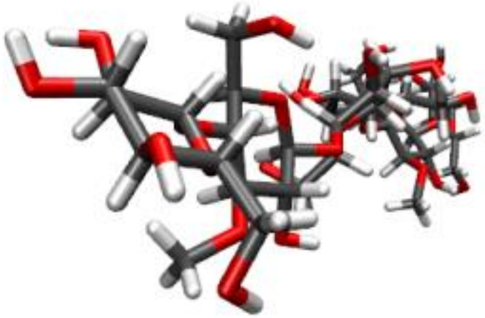

Representative structure of

ACACAC-OH

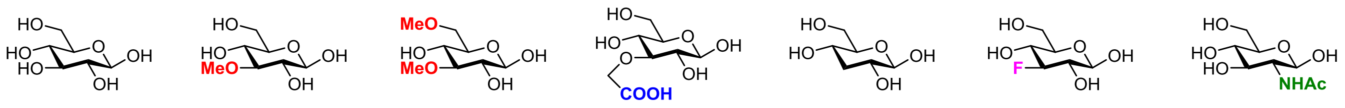

A B C D E F N

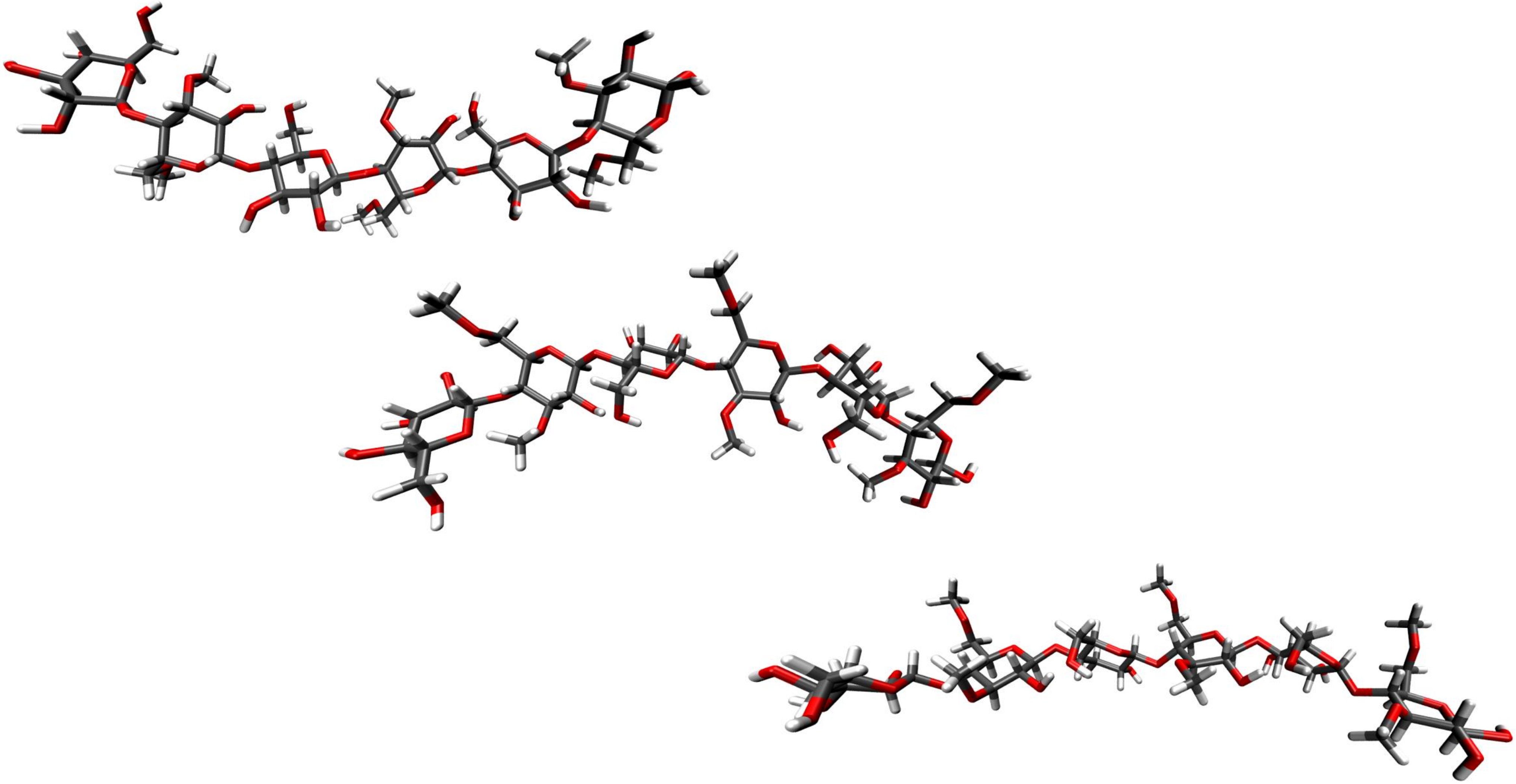

Representative structure of

ABACAB-OH

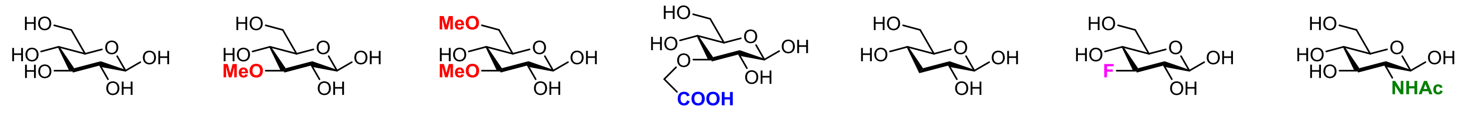

A B C D E F N

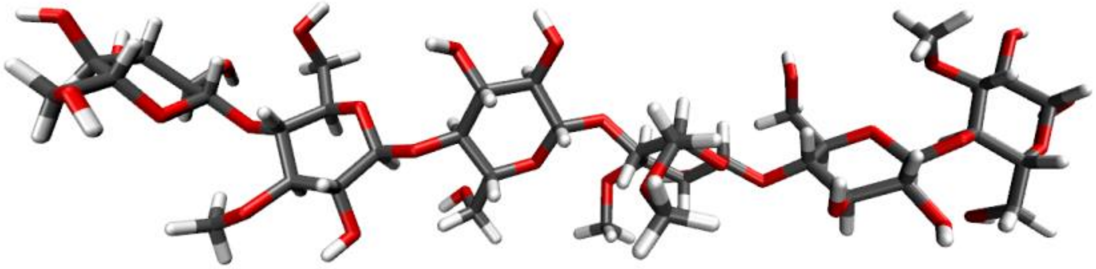

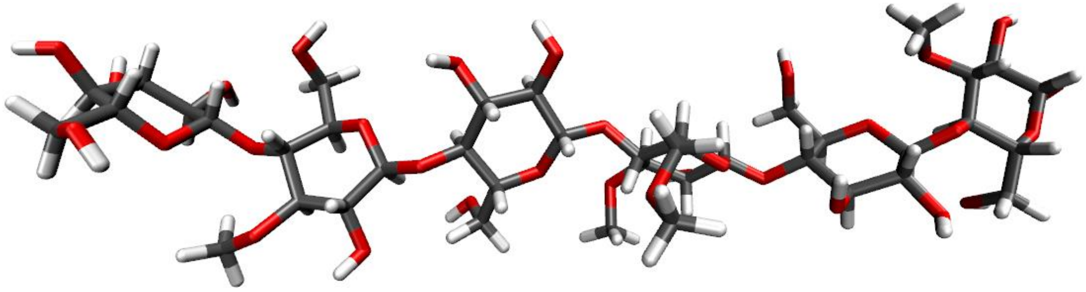

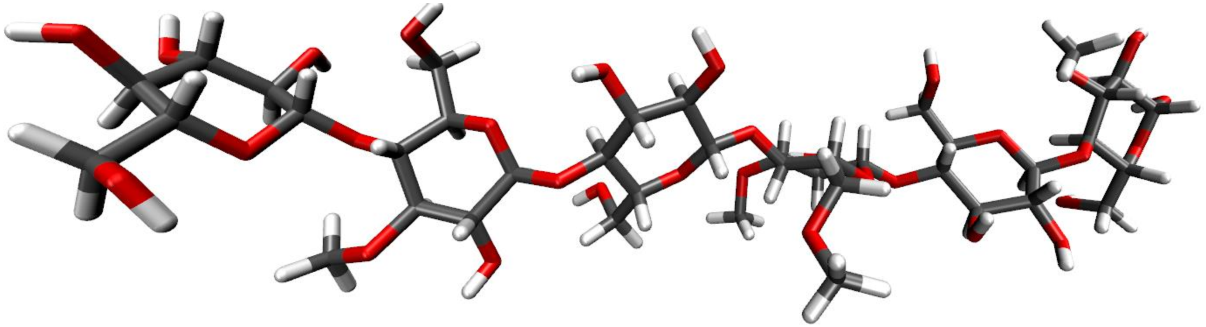

Representative structure of

ACAACA-OH

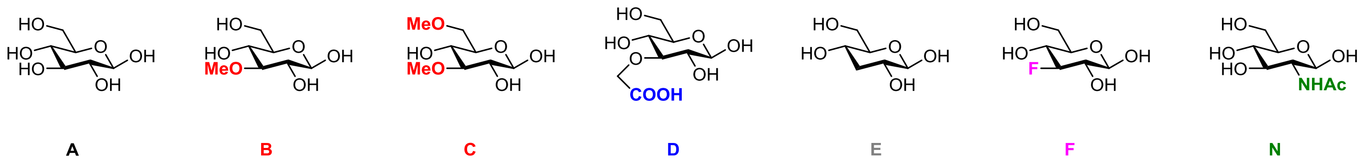

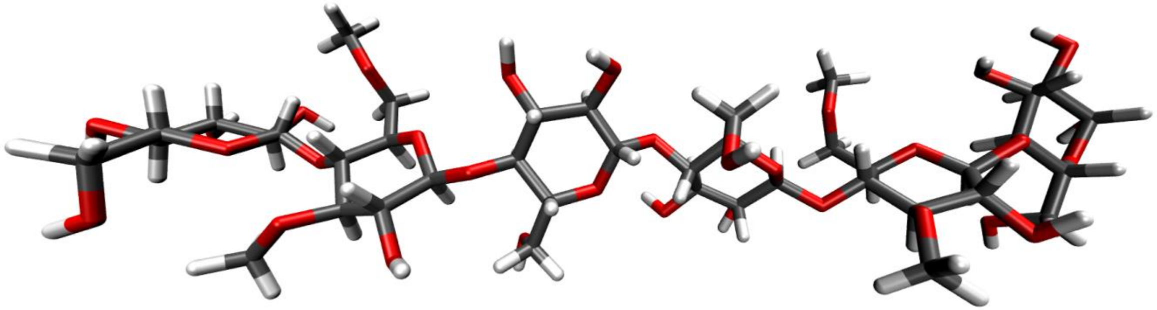

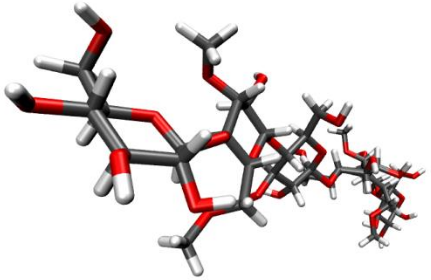

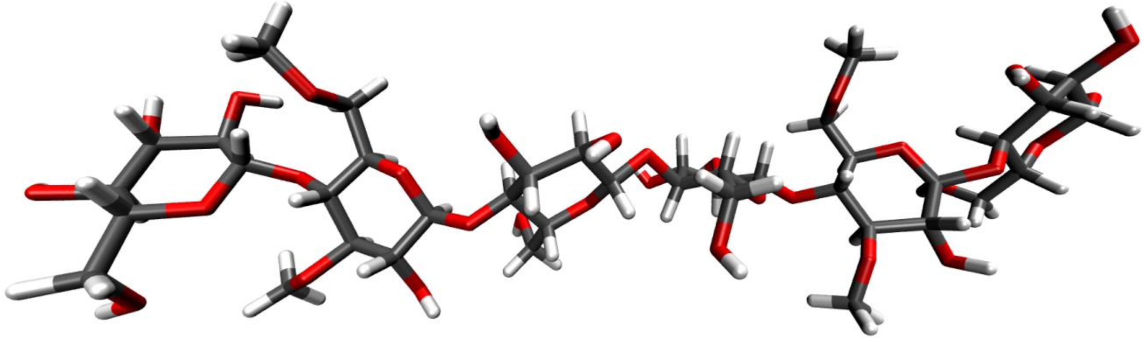

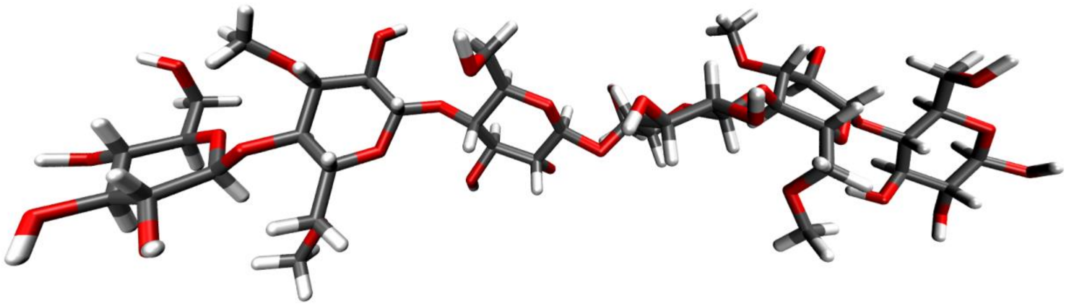

Representative structure of

FAFAFA-OH

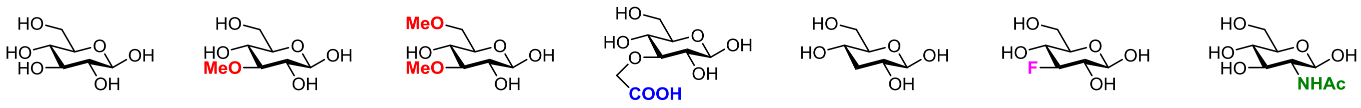

A B C D E F N

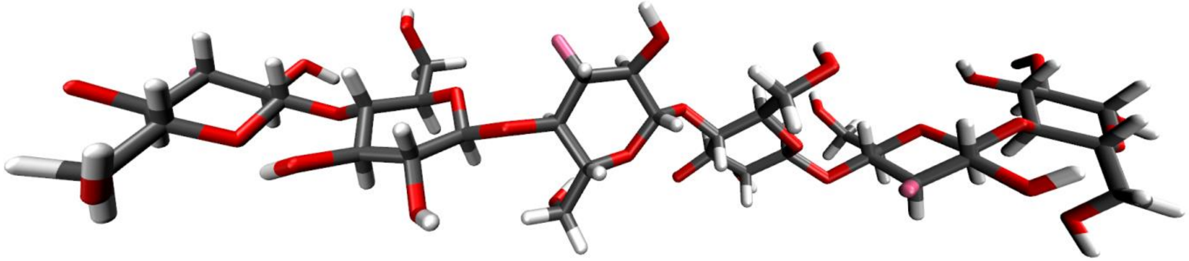

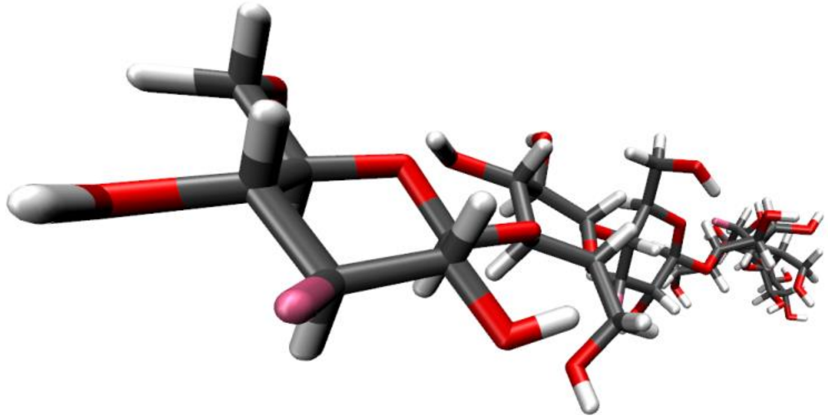

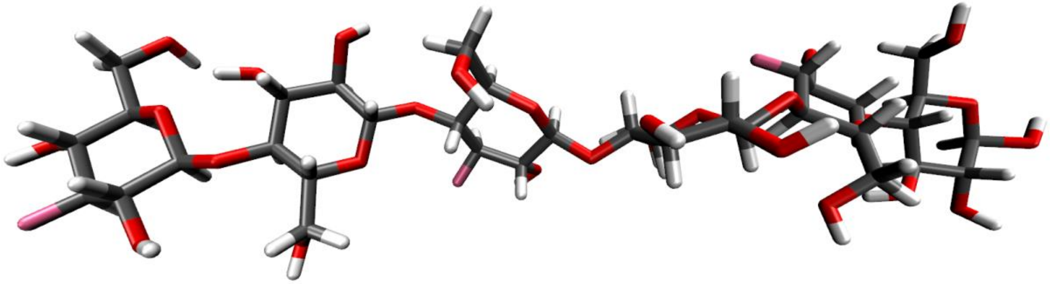

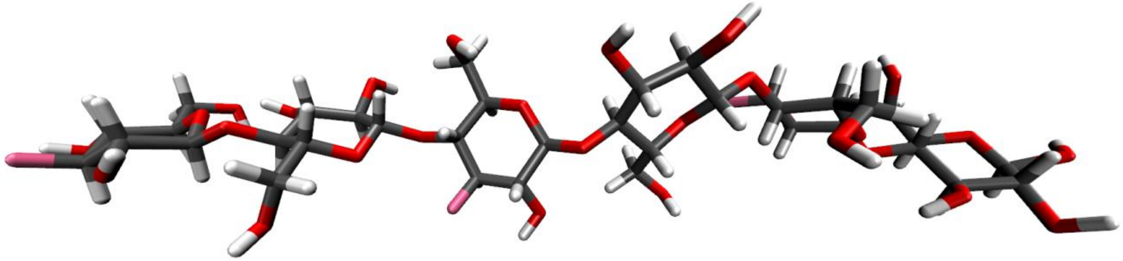

Representative structure of

AFAAFA-OH

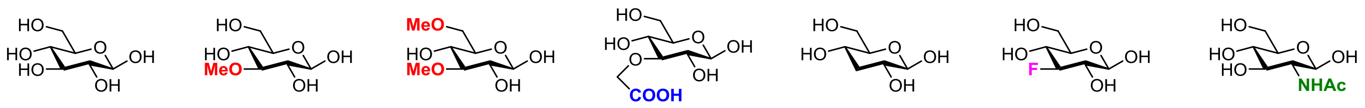

A **B** **C** **D** **E** **F** **N**

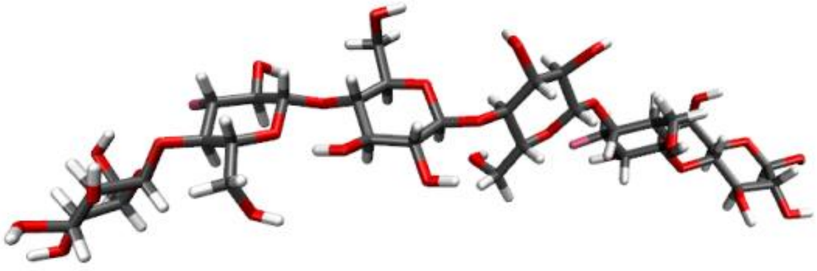

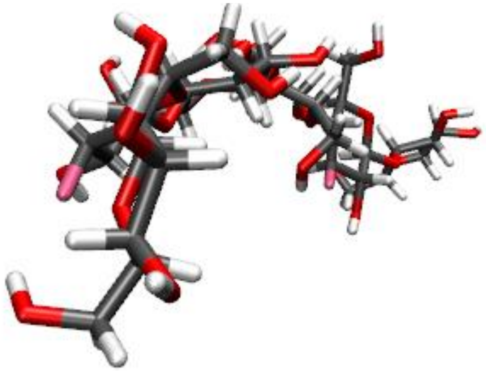

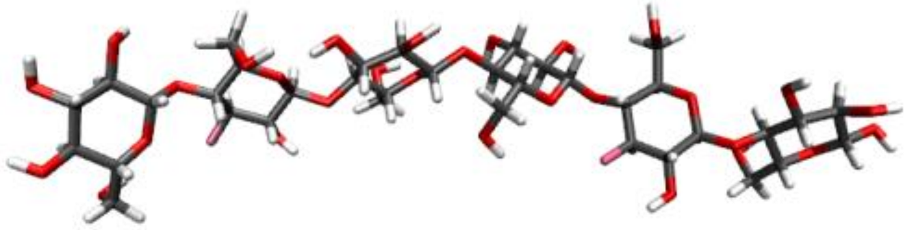

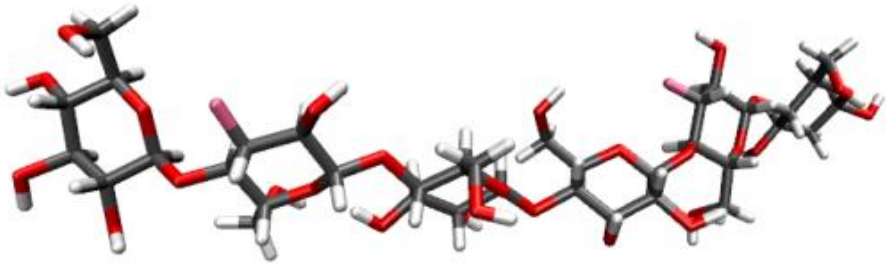

Representative structure of

NNNNNN-OH

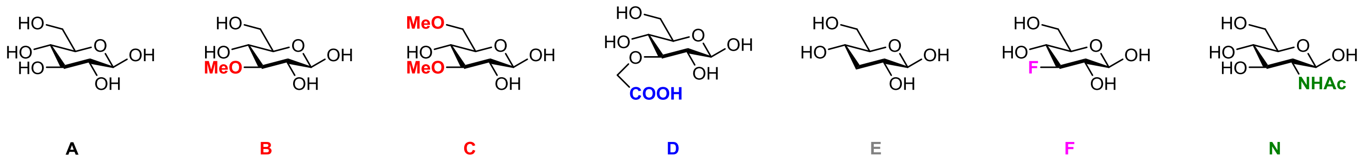

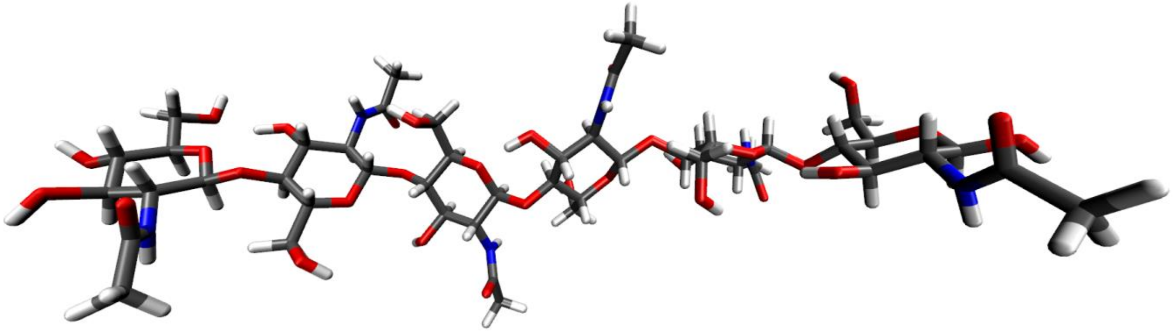

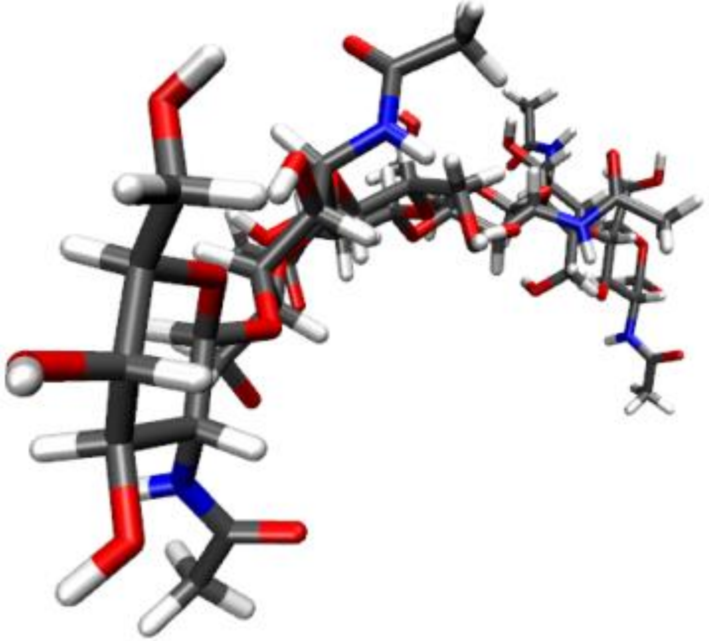

Representative structure of

ANAANA-OH

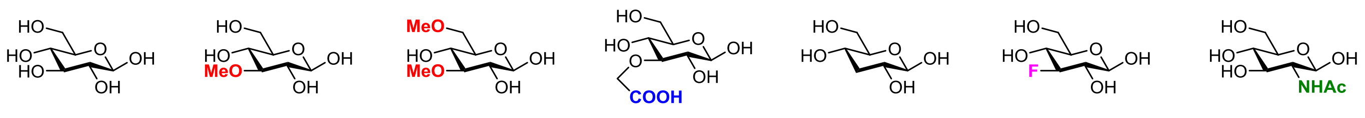

A B C D E F N

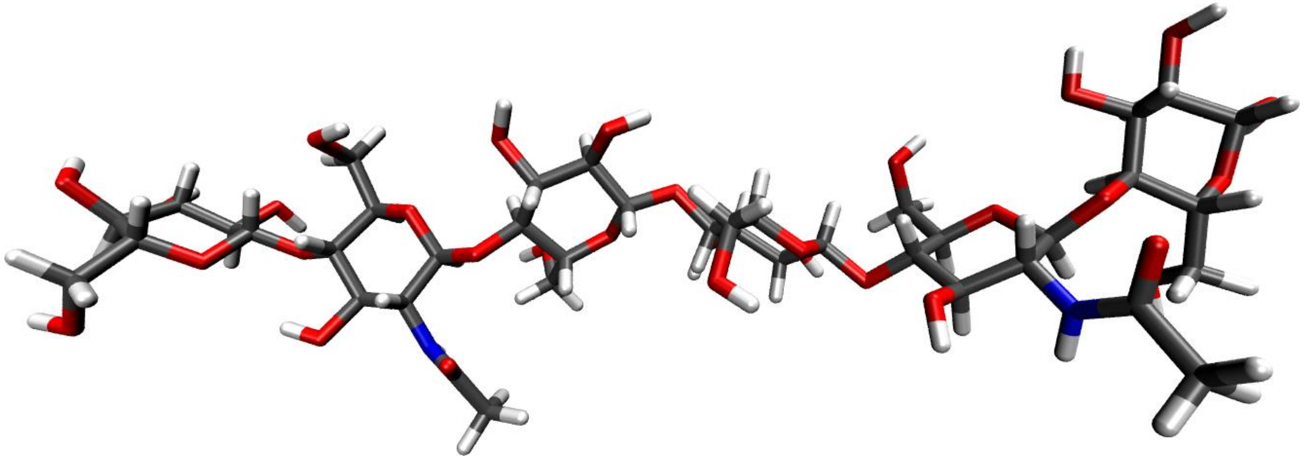

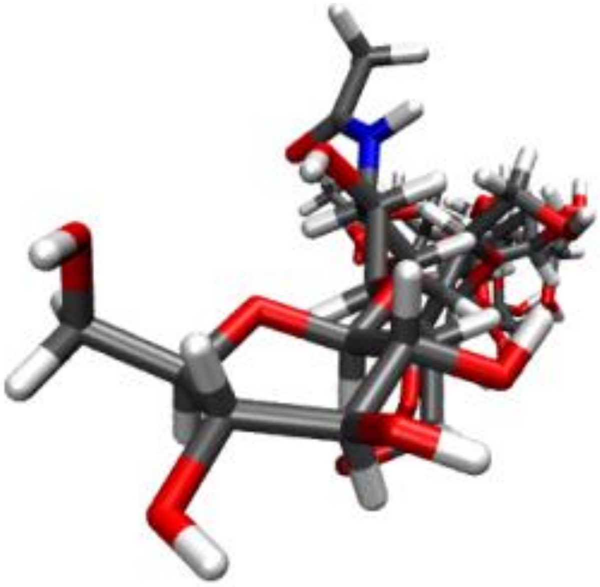

Representative structure of

AEAEAE-OH

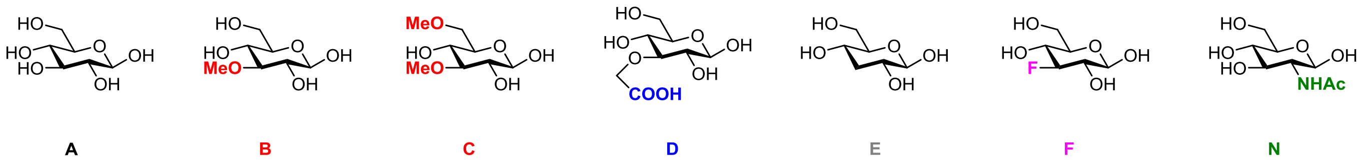

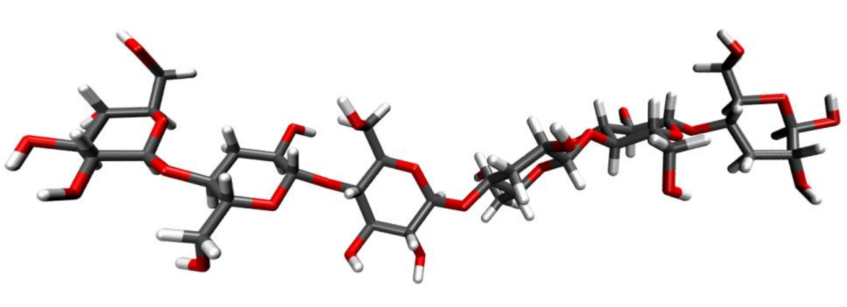

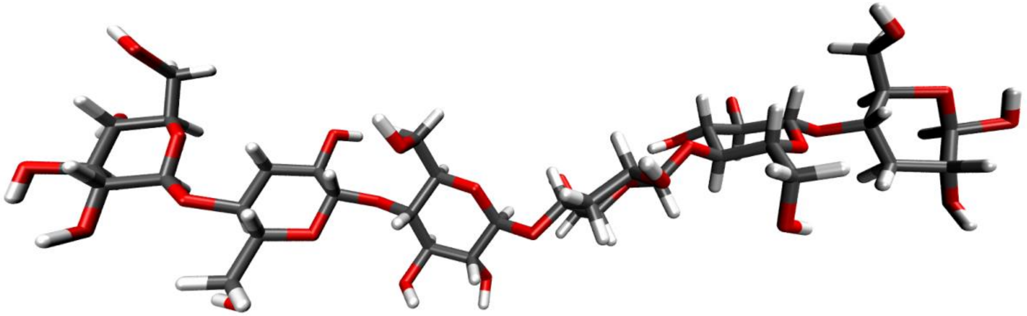

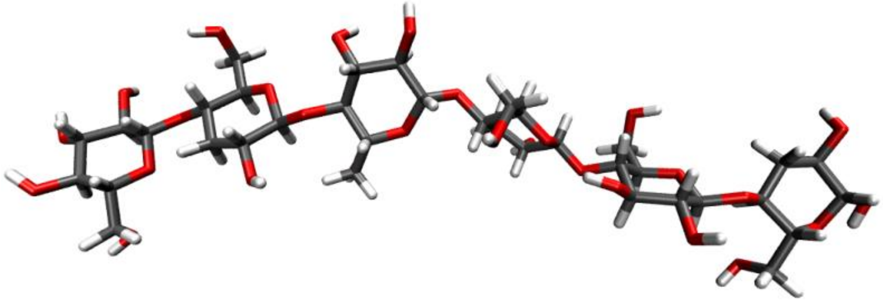

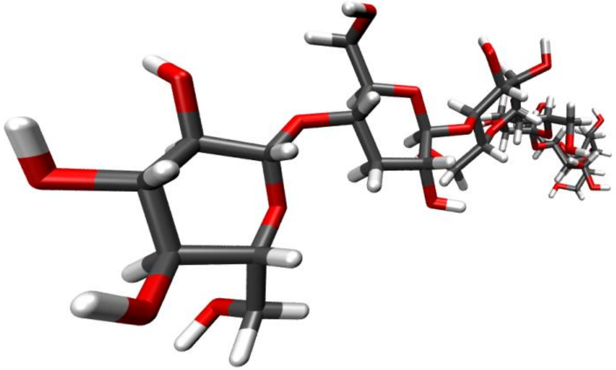

Representative structure of

AEAAEA-OH

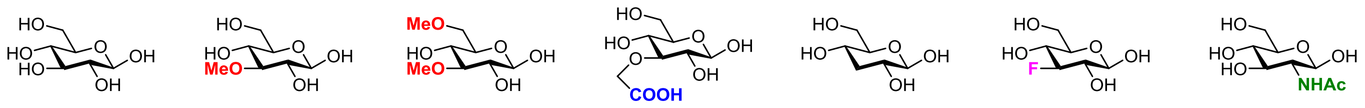

A B C D E F N

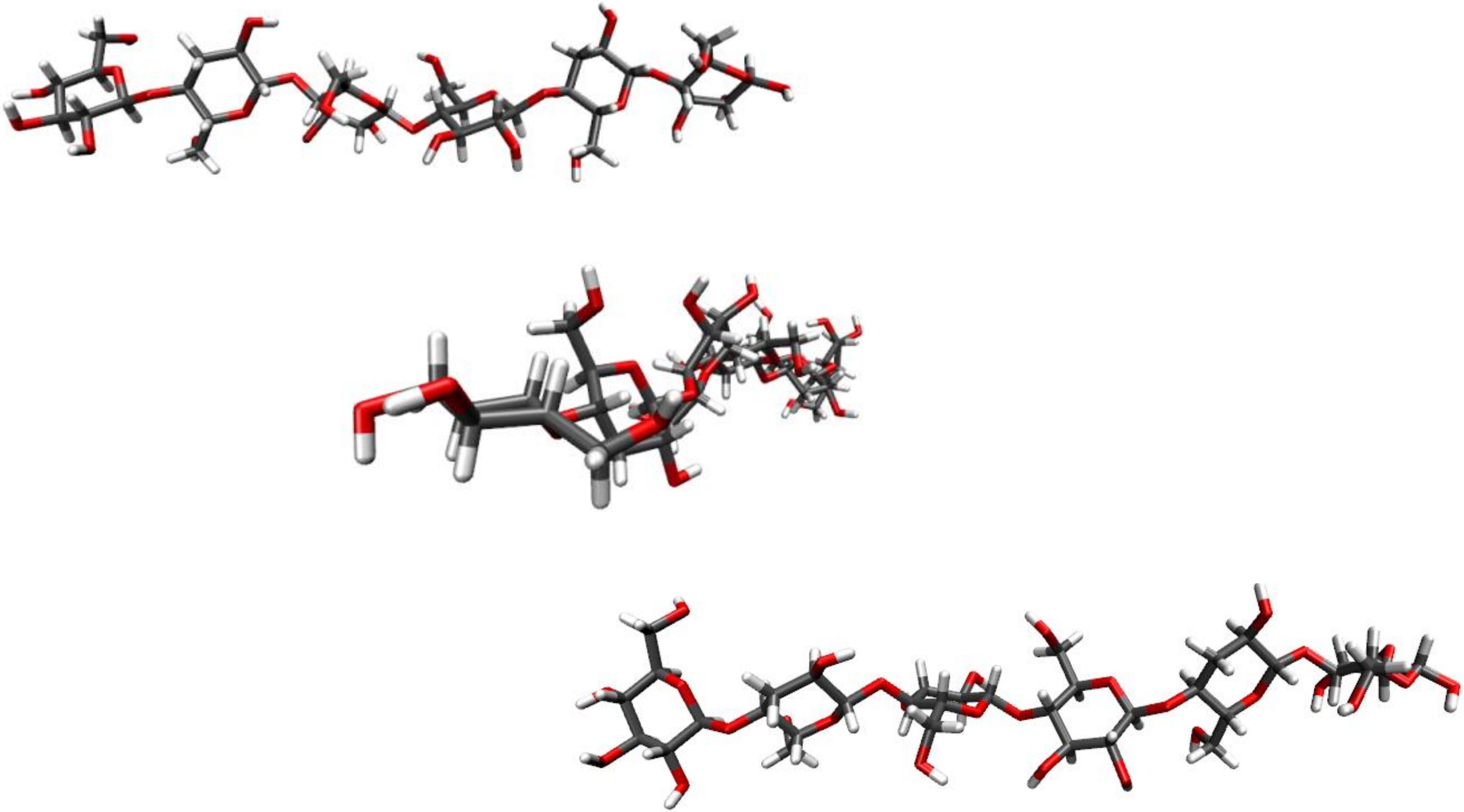

Representative structure of

DADADA-OH

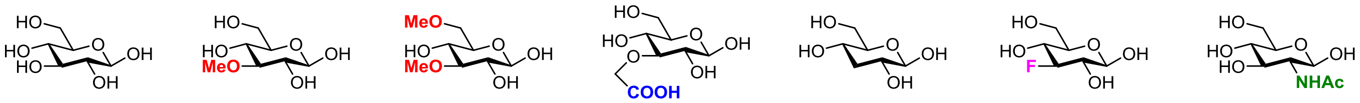

A B C D E F N

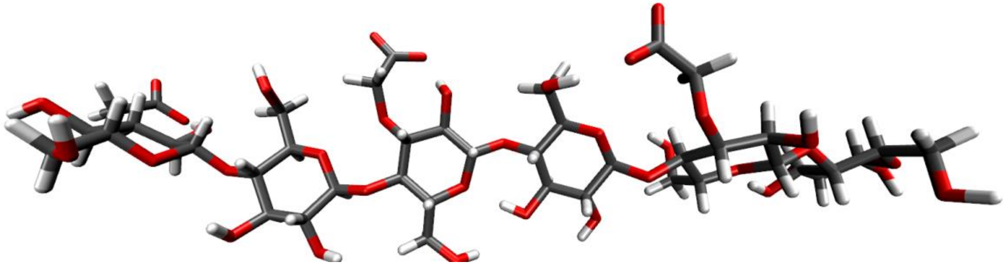

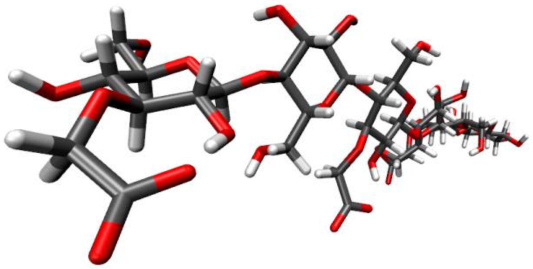

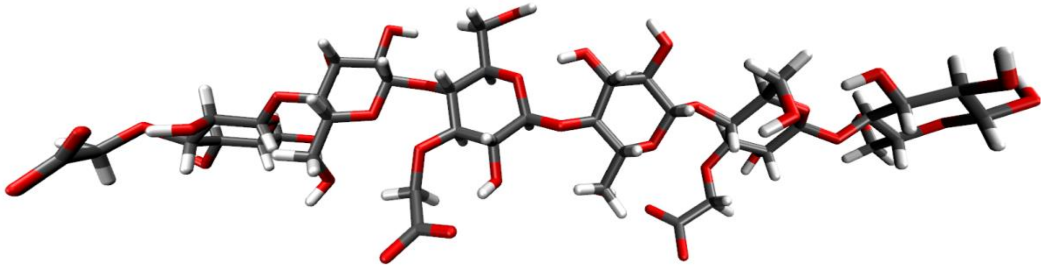

Representative structure of

ADAADA-OH

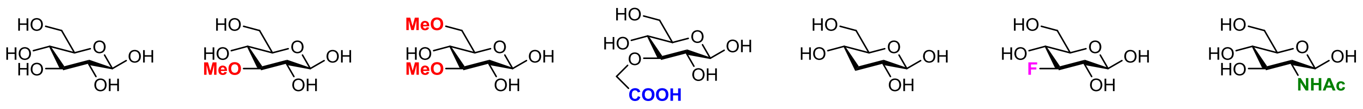

A B C D E F N

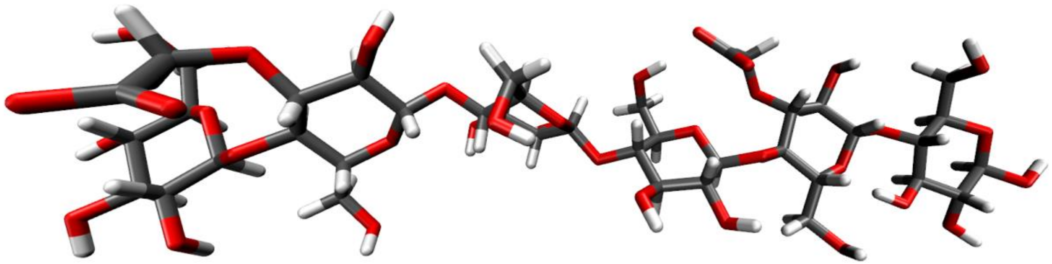

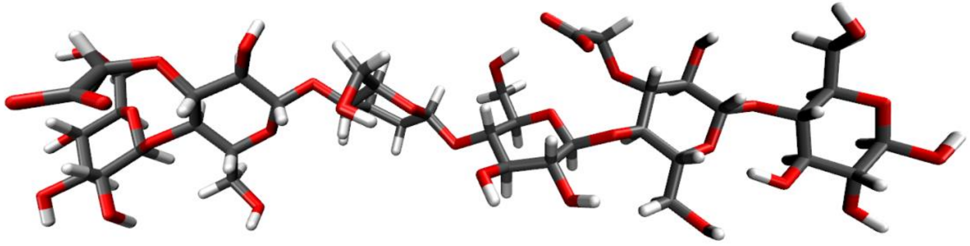

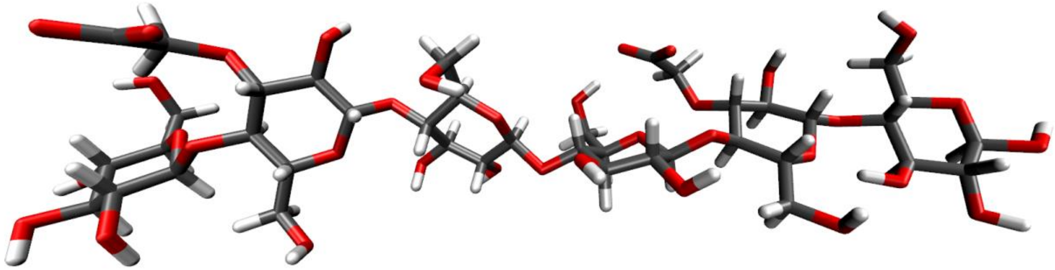

S15 End-to-end distance as a function of time

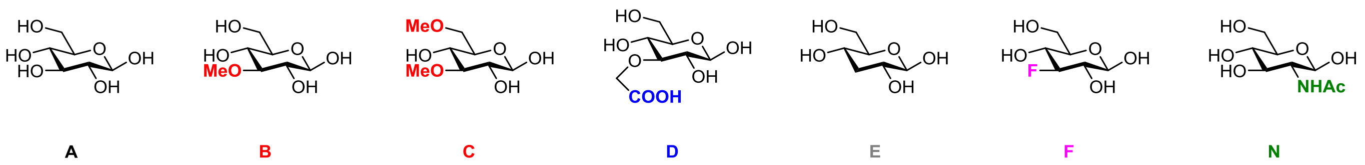

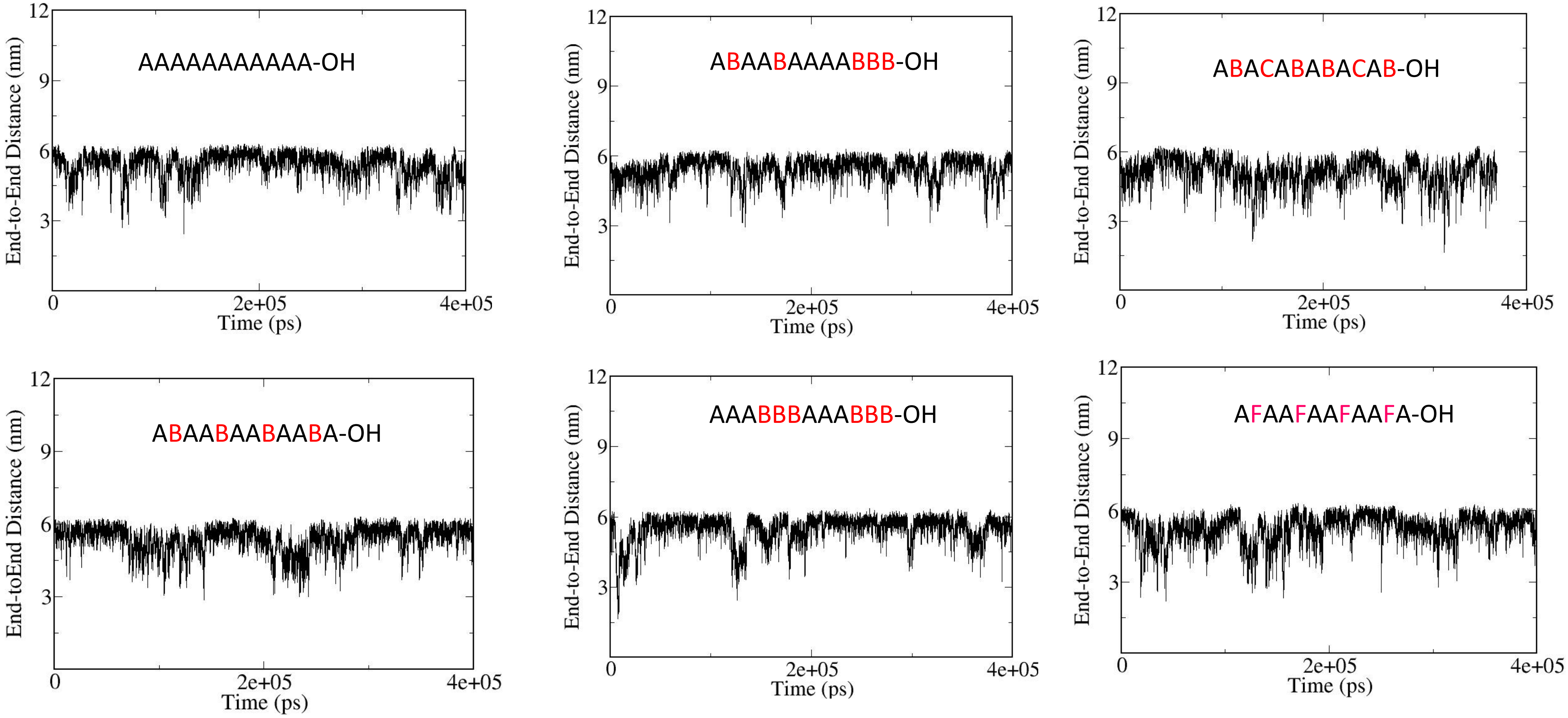

S16

Ramachandran plots

Overimposed phi and psi torsion angles

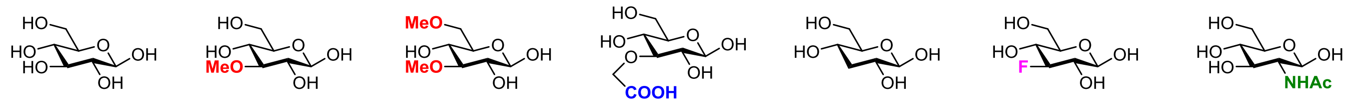

A B C D E F N

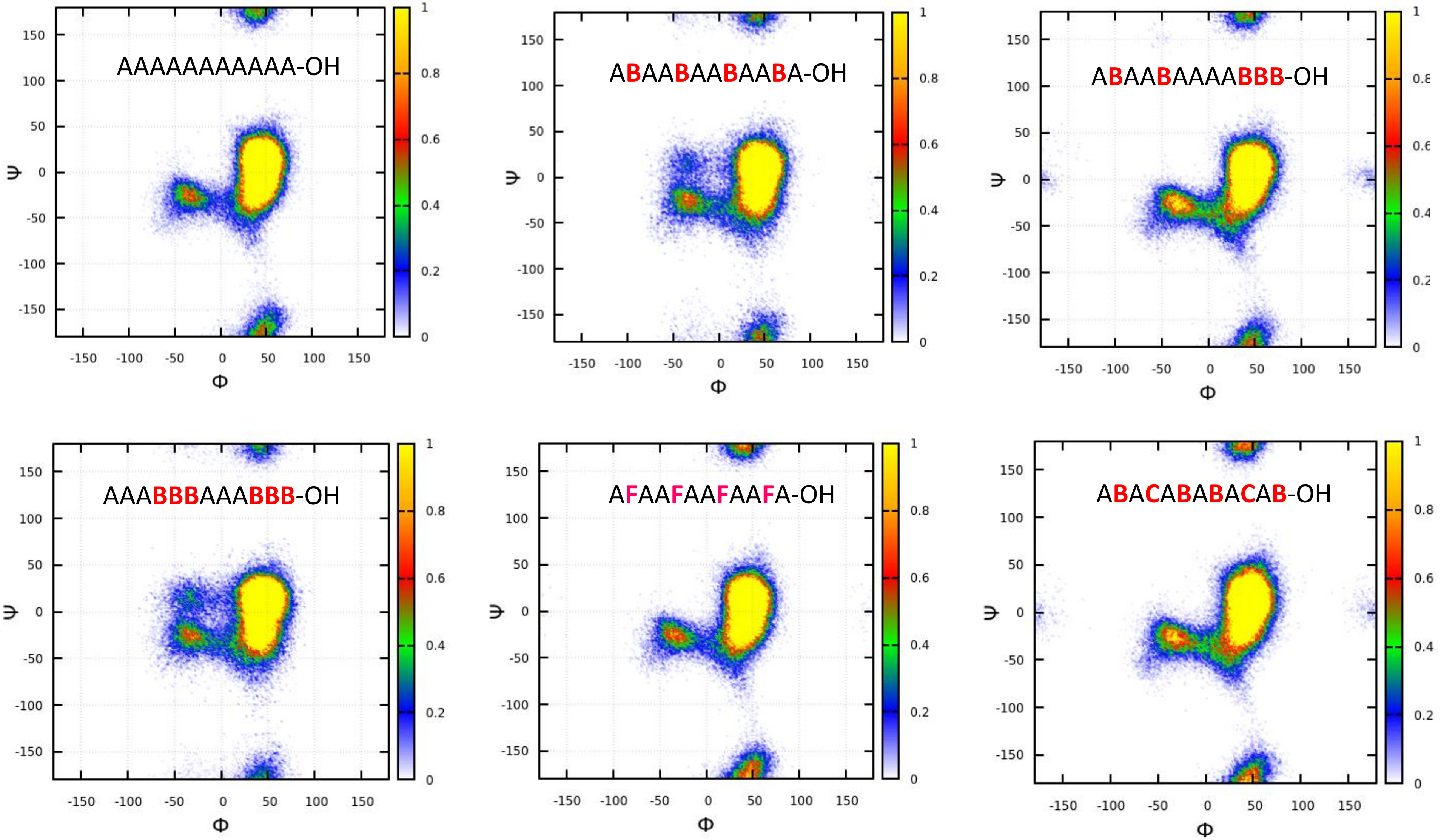

S17 Radial Distribution Functions

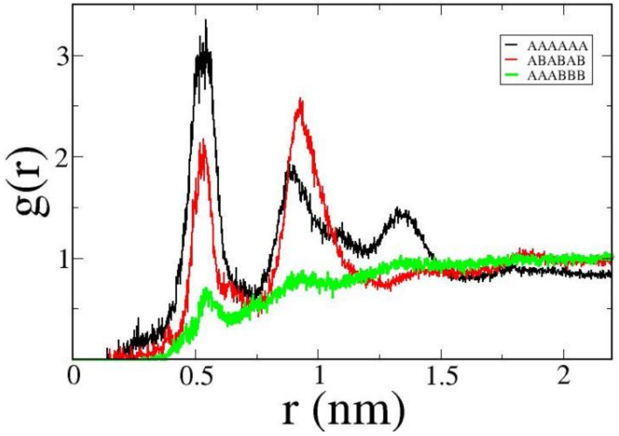

molecule - molecule interactions

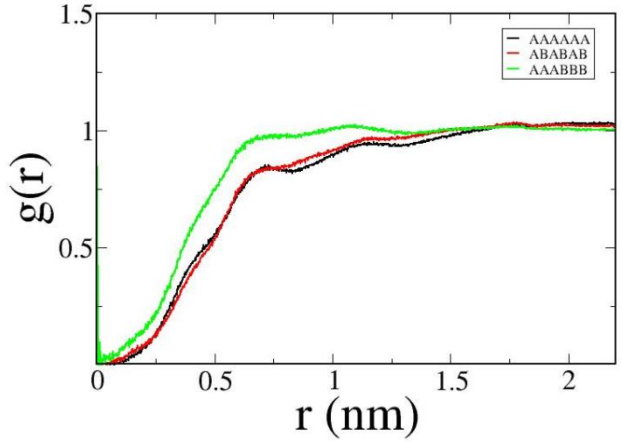

molecule - water interactions

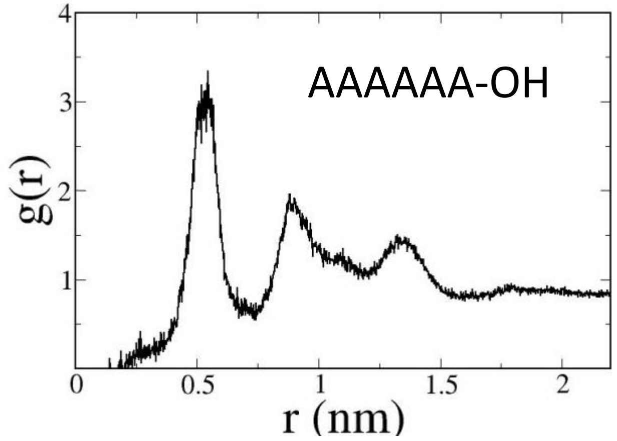

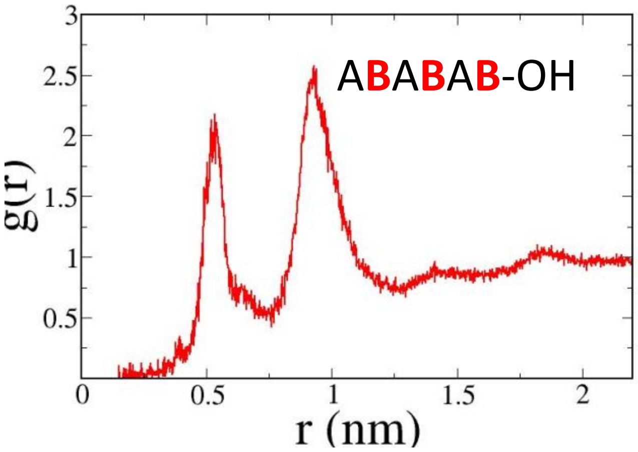

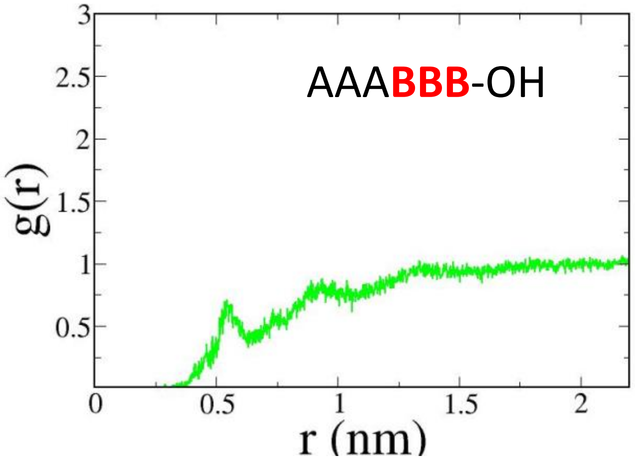

Radial Distribution Functions

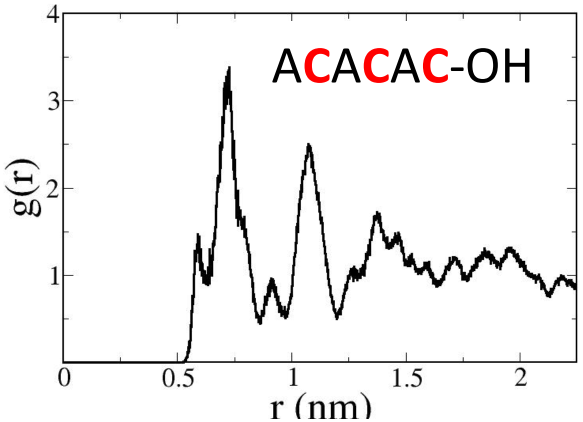

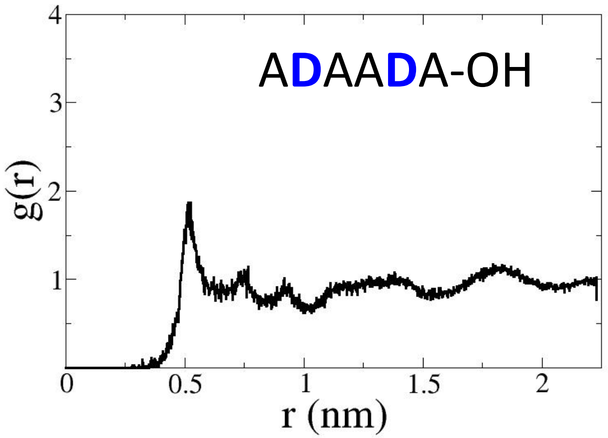

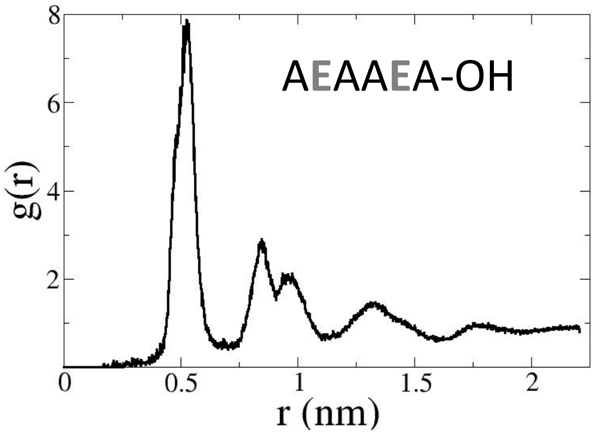

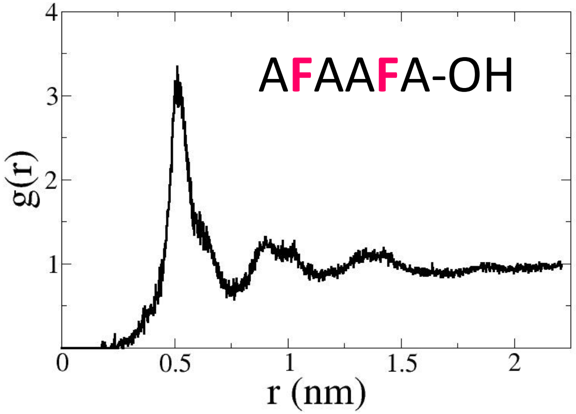

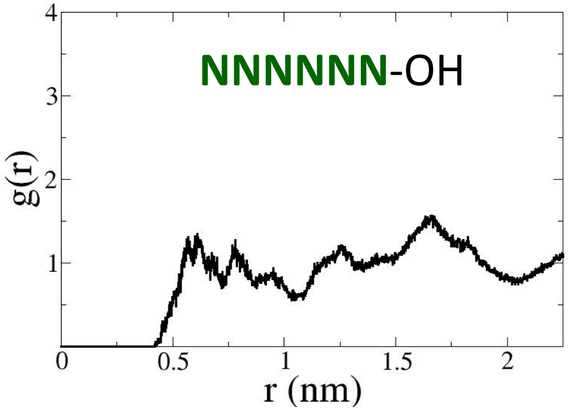

8. References

[1] M. Delbianco, A. Kononov, A. Poveda, Y. Yu, T. Diercks, J. Jiménez-Barbero, P. H. Seeberger, *J. Am. Chem. Soc.* **2018**, *140*, 5421-5426.

[2] Y. Yu, S. Gim, D. Kim, Z. A. Arnon, E. Gazit, P. H. Seeberger, M. Delbianco, *J. Am. Chem. Soc.* **2019**, *141*, 4833-4838.

[3] a) M. Egli, P. S. Pallan, C. R. Allerson, T. P. Prakash, A. Berdeja, J. Yu, S. Lee, A. Watt, H. Gaus, B. Bhat, E. E. Swayze, P. P. Seth, *J. Am. Chem. Soc.* **2011**, *133*, 16642-16649; b) P. Kováč, H. J. C. Yeh, C. P. J. Glaudemans, *Carbohydr. Res.* **1987**, *169*, 23-34.

[4] S. Arungundram, K. Al-Mafraji, J. Asong, F. E. Leach III, I. J. Amster, A. Venot, J. E. Turnbull, G.-J. Boons, *J. Am. Chem. Soc.* **2009**, *131*, 17394-17405.

[5] H. B. Mereyala, G. Pathuri, L. Nagarapu, *Synth. Commun.* **2012**, *42*, 1278-1287.

[6] a) S. G. Withers, M. D. Percival, I. P. Street, *Carbohydr. Res.* **1989**, *187*, 43-66; b) R. Danac, L. Ball, S. J. Gurr, A. J. Fairbanks, *Carbohydr. Res.* **2008**, *343*, 1012-1022.

[7] L. Krock, D. Esposito, B. Castagner, C.-C. Wang, P. Bindschadler, P. H. Seeberger, *Chem. Sci.* **2012**, *3*, 1617-1622.

[8] W. Group, University of Georgia, Athens, GA, **(2005-2018)**.

[9] a) E. J. Sorin, V. S. Pande, *Biophys. J.* **2005**, *88*, 2472-2493; b) M. Wehle, I. Vilotijevic, R. Lipowsky, P. H. Seeberger, D. Varon Silva, M. Santer, *J. Am. Chem. Soc.* **2012**, *134*, 18964-18972.

[10] M. W. Mahoney, W. L. Jorgensen, *J. Chem. Phys.* **2000**, *112*, 8910-8922.

[11] D. Spoel, E. Lindahl, B. Hess, G. Groenhof, A. E. Mark, H. J. C. Berendsen, *J. Comput. Chem.* **2005**, *26*, 1701-1718.

[12] T. Darden, D. York, L. Pedersen, *J. Chem. Phys.* **1993**, *98*, 10089-10092.

[13] B. Hess, H. Bekker, H. J. C. Berendsen, J. G. E. M. Fraaije, *J. Comput. Chem.* **1997**, *18*, 1463-1472.

[14] S. Miyamoto, P. A. Kollman, *J. Comput. Chem.* **1992**, *13*, 952-962.
